# Effect of praziquantel on the differential expression of mouse hepatic genes and parasite ATP binding cassette transporter gene family members during *Schistosoma mansoni* infection

**DOI:** 10.1371/journal.pntd.0005691

**Published:** 2017-06-26

**Authors:** Melissa C. Sanchez, Katina V. Krasnec, Amalia S. Parra, Christian von Cabanlong, Geoffrey N. Gobert, Boris Umylny, Pauline M. Cupit, Charles Cunningham

**Affiliations:** 1 Department of Biology, University of New Mexico, Albuquerque, New Mexico, United States of America; 2 School of Biological Sciences, Queen’s University, Belfast, United Kingdom; 3 National Center for Genome Resources, Santa Fe, New Mexico, United States of America; 4 Skaggs School of Pharmacy and Pharmaceutical Sciences, University of California San Diego, La Jolla, California, United States of America; University of Cambridge, UNITED KINGDOM

## Abstract

Schistosomiasis is a chronic parasitic disease caused by sexually dimorphic blood flukes of the genus *Schistosoma*. Praziquantel (PZQ) is the only drug widely available to treat the disease but does not kill juvenile parasites. Here we report the use of next generation sequencing to study the transcriptional effect of PZQ on murine hepatic inflammatory, immune and fibrotic responses to *Schistosoma mansoni* worms and eggs. An initial T helper cell 1 (Th1) response is induced against schistosomes in mice treated with drug vehicle (Vh) around the time egg laying begins, followed by a T helper cell 2 (Th2) response and the induction of genes whose action leads to granuloma formation and fibrosis. When PZQ is administered at this time, there is a significant reduction in egg burden yet the hepatic Th1, Th2 and fibrotic responses are still observed in the absence of granuloma formation suggesting some degree of gene regulation may be induced by antigens released from the dying adult worms. Quantitative real-time PCR was used to examine the relative expression of 16 juvenile and adult *S*. *mansoni* genes during infection and their response to Vh and PZQ treatment *in vivo*. While the response of stress genes in adult parasites suggests the worms were alive immediately following exposure to PZQ, they were unable to induce transcription of any of the 9 genes encoding ATP-binding cassette (ABC) transporters tested. In contrast, juvenile schistosomes were able to significantly induce the activities of ABCB, C and G family members, underscoring the possibility that these efflux systems play a major role in drug resistance.

## Author summary

Schistosomiasis is a disease caused by parasitic worms that significantly impacts the lives of approximately 260 million people. During infection, female parasites can release thousands of eggs, a significant number of which become trapped in the liver. Entrapment of eggs triggers immune and inflammatory responses that in turn causes many of the health problems associated with the disease. In this study, we analyzed gene expression profiles of livers from mice infected with the human parasite *Schistosoma mansoni* over multiple time points, beginning when the parasites start to lay eggs. In mice treated with a lethal dose of the anti-schistosomal drug praziquantel, we identified a number of different genes and pathways central to immune and inflammatory responses that are active even in the absence of egg deposition. Praziquantel is the only drug available to treat schistosomiasis, however, it displays bimodal activity in that it is ineffective against juvenile parasites during the early stages of mammalian infection. We also show that development of drug resistance may be due to the improved efficacy of the juvenile parasite to actively excrete the drug. These results provide insights into the effect of praziquantel on the host response to infection as well as the ability of juvenile parasites to overcome the drug’s lethal effect.

## Introduction

Schistosomiasis is a chronic neglected tropical disease caused by digenetic parasitic flatworms of the genus *Schistosoma*. In 2014, 259 million people were treated for the disease with 91.4% of those individuals living in Africa where *Schistosoma mansoni* and *S*. *haematobium* are the major causative agents [1]. Of those infected globally, an estimated 123 million were school-aged children [1]. The human cost of schistosomiasis in 2010 was calculated at approximately 3 million years lived with disability [2], however, accurate numbers are difficult to pin down for a number of reasons, not least of which is the contribution of co-infection with other helminths as well as HIV [3].

The complex life cycle of *Schistosoma spp*. requires that free-living cercariae released from infected fresh water snails burrow through human skin and enter the venous circulation as schistosomulae [4]. Within a week, parasites migrate to the lungs and reach the hepatoportal circulation approximately 14 days after penetration of the skin. In experimental mouse disease models, schistosomes do not develop synchronously though most can be considered as sexually immature juveniles between 14 and 28 days post infection, with sexual maturation occurring from day 28 onward. In the case of *S*. *mansoni*, sexually mature adult male and female worms reside in the hepatic portal and mesenteric venous systems of the host with the female releasing increasing numbers of fertilized eggs, of which approximately half will migrate through the bowel wall to be excreted in the host stool. The remaining eggs often become trapped in the liver where they trigger host inflammatory responses. Hepatic fibrosis and granuloma formation caused by the deposition of collagen and extracellular matrix components around the eggs leads to the occlusion of the hepatic portal veins, which in turn causes portal hypertension, enlargement of the spleen, ascites, and gastrointestinal bleeding [4,5].

Numerous studies have tracked the expression of immune and inflammatory hepatic genes during infection, inflammation and granuloma formation. In the early stages of infection, a T-helper cell type 1 response (Th1) against the parasite has been recognized through increased production of pro-inflammatory cytokines (TNFα, IL1α, IL1β and IL6) as well as Signal Transducers and Activators of Transcription 1 (STAT1) and IFNγ [5–7], while elevated Th17 cell numbers have been suggested to mediate bladder pathology during *S*. *haematobium* infection [8]. As parasite egg deposition commences, a contemporaneous Th2 response characterized by a surplus of chemokine expression as well as IL4, IL5, IL10, IL13 and IL33 production also gets underway [5–7, 9]. In addition, production of IL10 by regulatory T cells (Treg) may help to control pathology along with IL10 independent naturally occurring CD4+ Foxp3+ regulatory T cells [10, 11]. Chuah and colleagues used microarrays to study neutrophil mediated changes in gene expression during granuloma formation in *S*. *japonicum* infected mice and found a significant up-regulation of Th1, Th2 and Th17 immune genes, as well as inflammatory genes within the granuloma that are spatially and temporally separated [12].

While a number of drugs have been used to treat schistosomiasis only one, praziquantel (PZQ), is widely employed as it is relatively cheap, easy to use, and effective against all schistosome species that infect humans [13]. Its use in the past 10 years has increased significantly as the number of patients treated has grown from approximately 12 million in 2006 to 61.6 million in 2014 when 20.7% of affected individuals received the drug [1]. This has largely been due to the implementation of mass treatment campaigns with, for example, an increase of 52.3% in those receiving treatment in the African region when comparing 2014 with the previous year [1].

Although PZQ brings relief to those treated, it does not provide a cure as juvenile schistosomes are relatively resistant to the anthelmintic effects of the drug [14–17]. When available, administration of PZQ is often limited to a single dose per year and treated individuals, frequently children, will quickly become reinfected through continued and unavoidable exposure to the parasite. Additionally, any juvenile parasites that escape elimination during treatment subsequently mature and begin to release eggs. The frequent exposure and survival of resistant juveniles to PZQ also gives concern that under this ineffective pressure, drug resistance could emerge [18].

In the absence of an anti-schistosomal vaccine, one route to improving treatment of patients is to enhance the efficacy of PZQ by increasing the sensitivity of juvenile parasites to the drug. This approach requires an understanding of the molecular basis of juvenile resistance. While it is possible resistance may be driven by a reduction in expression of the drug target or an increased rate of drug metabolism, we and others have suggested that ATP Binding Cassette (ABC) multi-drug transporters play an important role [19–23]. These trans-membrane proteins work by hydrolyzing ATP and using the energy liberated to move compounds, including drugs, across membranes. Drug resistance results from the amplification, over-expression or modification of some members of this transporter family [24].

In the current study we use next generation sequencing (RNA-Seq) to quantitate the effect of PZQ on the mouse hepatic transcriptomic response to *S*. *mansoni* infection during the immediate two-week period following the transition of the parasite from sexually immature juvenile to mature adult. We observe that PZQ has little effect on immune and inflammatory gene regulation in the period immediately following drug treatment, but do note a significant reduction in these responses in the absence of egg deposition two weeks after treatment commenced. In addition, we investigate the response of juvenile and adult schistosomes to treatment with PZQ *in vivo* by quantitative real-time PCR and demonstrate that juvenile schistosomes may protect themselves from the lethal effects of the drug through up-regulation of a number of ABC transporter genes.

## Materials and methods

### Ethics statement

This study was performed in accordance with the Guide for the Care and Use of Laboratory Animals of the U.S. National Institutes of Health. Animal use procedures were reviewed and approved by the University of New Mexico Institutional Animal Care and Use Committee.

### Mice, parasites and experimental procedures

For all infection experiments, eight to ten-week-old female Swiss Webster outbred mice (Charles River, Kingston, NY) were each infected percutaneously with approximately 150 *S*. *mansoni* Puerto Rican 1 (PR1) cercariae. Treatment with PZQ (Sigma-Aldrich, St Louis, MO) was administered by gavage at a dose of 250 mg/kg/day in vehicle (Vh) (Cremophor EL, Sigma-Aldrich) for four consecutive days. Control mice were either infected or left uninfected and administered an equivalent volume/kg/day of Vh for four consecutive days.

To determine the effect of PZQ or Vh on the number of parasites present in the livers of mice during and after treatment, PZQ or Vh was administered to two groups of 15 mice infected with *S*. *mansoni* beginning on day 32 post infection (from this point onwards all data points will be defined by the time post cercarial challenge i.e., days 32, 35, 39 or 46). After infection, mice were randomly distributed into the treatment or control group and five mice from each group sacrificed 3 h after the initial treatment on day 32, after the final treatment on day 35 and 14 days after treatment commenced (day 46). After euthanasia with sodium pentobarbital, livers were collected, gently shredded and parasites counted (n = 5 per group).

The number of eggs present in livers of infected mice receiving PZQ or Vh for four consecutive days starting on day 32 was also assessed. Mice were sacrificed (n = 4 per group) on days 32 and 35 (3 h after treatment) and on days 39 and 46. Livers were digested at 37°C overnight in 4% potassium hydroxide and the number of eggs per gram of liver calculated [25].

To determine changes in expression of hepatic and schistosomal genes during infection and PZQ treatment, two groups of 16 mice were infected with *S*. *mansoni* and treated with PZQ or Vh for 4 consecutive days beginning on day 32. Mice were sacrificed 3 h after treatment (n = 4 per group) on days 32 and 35 and on days 39 and 46. A third group of 16 uninfected mice were treated with a weight related dose of Vh (S1 Fig). Three of the four whole livers were randomly selected and placed in RNA*later* (ThermoFisher Scientific, Waltham, MA) for RNA isolation. The remaining liver was placed in 10% formalin for histological analysis. Livers from four uninfected Vh treated mice at each time interval were used as controls to provide gene expression and histological baseline data.

### Isolation of total RNA

Livers were removed from RNA*later*, weighed then placed in RNeasy lysis buffer with 1% 2-mercaptoethanol (Sigma-Aldrich) and homogenized. Total RNA was isolated using an RNeasy Maxi Kit (Qiagen) and digestion with RNase-free DNase (Qiagen, Redwood City, CA) according to the manufacturer’s instructions. Total RNA was quantified using a ND-1000 spectrophotometer and the quality verified using an Agilent 2100 Bioanalyzer (Agilent, Santa Clara, CA). RNA was stored at -80°C. Representative bioanalyzer traces from each treatment group are shown in S2 Fig and a minimal RNA integrity number (RIN) of 6 was the threshold for samples used in this study.

### Next generation sequencing: cDNA library construction, sequencing and data processing

RNA sequencing (RNA-Seq) was conducted on three separate biological replicates representing each type of treatment and time-point using the Illumina Next Generation Sequencing (NGS) platform (Illumina, San Diego, CA) and was performed at the National Center for Genome Resources (NCGR, Santa Fe, NM). First strand cDNA libraries were prepared from 500 ng of polyA^+^ heat-fragmented RNA using Superscript II (Invitrogen, Carlsbad, CA) and random hexamer primers followed by second strand cDNA synthesis with Second Strand Master Mix (Illumina). Universal and bar coded TruSeq Adapters were ligated to cDNA ends and the resulting adapted cDNA libraries were PCR amplified and further purified. Samples were normalized to a concentration of 10 nM and pooled prior to sequencing. Sequencing was performed on a HiSeq 2000 instrument (Illumina) to generate 50 base-pair single-end reads. The raw sequence reads for each sample were checked for quality control using FastQC (http://www.bioinformatics.bbsrc.ac.uk/projects/fastqc) and mapped to the *S*. *mansoni* (annotation: Ensembl release 75) and *Mus musculus* (assembly: GRCm38, annotation: Ensembl release 75) genomes using TopHat2 [26]. The resulting alignment files were analyzed with Cufflinks [27] and HTSeq-Count [28] to generate a transcriptome assembly for each data set [29]. Differentially expressed genes (DEG) were identified in PZQ and Vh treated infected transcriptomes after normalization against Vh treated uninfected transcriptomes using three separate analysis tools: Cuffdiff [30], DESeq [31] and edgeR [32] with cutoffs for all programs set at an adjusted p ≤0.05 with log_2_ fold change either <-1 (down-regulated genes) or >1 (up-regulated genes). DEG lists for each sample from each program were displayed using a Venn diagram and only genes common in multiple bioinformatics approaches were retained for further analysis. The online Lumenogix platform (api.lumenogix.com) was used to perform all bioinformatics analysis [33]. Two-dimensional principal component analysis (PCA) to characterize inter-variable relationships was performed using R package DESeq2. All high-throughput sequence data sets generated for this study were deposited with Gene Expression Omnibus and can be accessed through series GSE19432.

### Pathway analysis

Final DEG lists from RNA-Seq analysis were analyzed with Ingenuity Pathway Analysis (IPA) tool version 6 (Qiagen). The IPA Knowledge Base incorporates in-house curating and public databases to formulate and update signaling pathways. Overrepresented pathways are measured as the probability of association between experimental gene sets (DEG) compared to a reference gene set for specific processes or pathways. A right-tailed Fisher’s Exact Test resulting in a p <0.05 indicates a statistically significant non-random association.

### Gene ontology analysis

Protein Annotation Through Evolutionary Relationship (PANTHER) was used to classify differentially expressed genes with ontology terms to identify biological functions present in the final DEG lists based on the statistical overrepresentation test [34].

### Quantitative real-time PCR

Mouse RNA used for quantitative real-time PCR (qRT-PCR) originated from the corresponding liver samples used for Illumina sequencing. qRT-PCR was performed on six mouse genes to validate the expression patterns observed with RNA-Seq analysis. These genes were chosen for validation based on their differentially expressed profile in the Illumina data sets, i.e., having > 20 reads per sample and the qRT-PCR primers passing efficiency tests. One microgram of total RNA was reverse transcribed in a 20 μL reaction using the iScript cDNA Synthesis Kit (Bio-Rad Laboratories, Hercules, CA) according to the manufacturer’s instructions. Predesigned PrimeTime qRT-PCR gene specific assays (Integrated DNA Technologies; primer sequences are shown in S1 Table) were used for RNA-Seq validation. qRT-PCR was carried out in 20 μL reactions containing 100 ng cDNA, 0.5 μM primer and SsoAdvanced Universal SYBR Green Supermix (Bio-Rad Laboratories) and performed in biological triplicates with technical duplicates using a C1000 96 Touch Thermo Cycler (Bio-Rad Laboratories). PCR cycling conditions for RNA-Seq validation were 95°C for 2 min followed by 40 cycles at 95°C for 5 s, 60°C for 30 s and 65°C for 15 s. Relative expression (2^-ΔΔCt^) was performed using CFX Manager Software v3.1 (Bio-Rad Laboratories) to calculate the fold change relative to the reference *Mus musculus* gene glyceraldehyde-3-phosphate dehydrogenase (PrimePCR SYBR Green Assay: GAPDH, Mouse, Bio-Rad Laboratories). Expression of this gene did not vary significantly between treatment groups. Correlation between qRT-PCR and RNA-Seq data was assessed using a Spearman’s Rho correlation as the data were not normally distributed. Data analysis and statistical comparisons were performed using GraphPad Prism (GraphPad Software Inc., La Jolla, CA).

The expression of 16 juvenile and adult *S*. *mansoni* genes were analyzed by qRT-PCR. These represented genes we previously reported to be differentially regulated as a result of PZQ treatment *in vitro* [22] or those identified in the recent literature as being affected by PZQ including ABC multi-drug transporter, calcium regulatory and stress-related family members.

To investigate the effect of drug treatment on juvenile *S*. *mansoni* gene expression *in vivo*, groups of infected mice were treated with Vh or PZQ on four consecutive days beginning on day 25 as described above. Mice were sacrificed 3 h after treatment (n = 4 per group) on days 25 and 28 and the livers placed in RNA*later* for total RNA isolation. For qRT-PCR analysis, mRNA was reverse transcribed and parasite gene specific cDNA amplified. Similarly, mouse liver RNA samples used for Illumina sequencing that contained infecting adult *S*. *mansoni* mRNA were reverse transcribed and parasite gene specific cDNA amplified. The general conditions for *S*. *mansoni* qRT-PCR were as described above. *S*. *mansoni* GAPDH was used as a housekeeping gene to calculate relative fold change as its expression did not vary significantly between treatment groups (S2 Table). qRT-PCR cycling conditions for *S*. *mansoni* gene expression were 95°C for 10 min followed by 40 cycles at 95°C for 15 s, 60°C for 1 min and 60°C for 20 s. PCR primers for *S*. *mansoni* genes (S3 Table) were designed using the Integrated DNA Technologies OligoAnalyzer tool (www.idtdna.com). Fold change was calculated as described above. One-way analysis of variance (ANOVA) was performed to calculate statistical significance of normalized gene expression between treatment groups and where significance was detected the Fisher’s least significant difference post hoc test for multiple comparisons was used with p <0.05 considered statistically significant.

## Results and discussion

### Effect of praziquantel on parasitic infection

The initial research aim was to identify a suitable four-day treatment window when the majority of infecting *S*. *mansoni* would be mature enough to be PZQ sensitive, but young enough that egg accumulation and resultant tissue damage in the liver would be negligible. This would allow a comparison of the host hepatic transcriptome giving insights into development of both the immune and inflammatory response to PZQ treated worms as well as to the ongoing infection in those treated with Vh. In addition, it was intended initially that the worm transcriptomic response to both PZQ and mouse immune and inflammatory reaction to infection might also be acquired.

**Fig 1:**
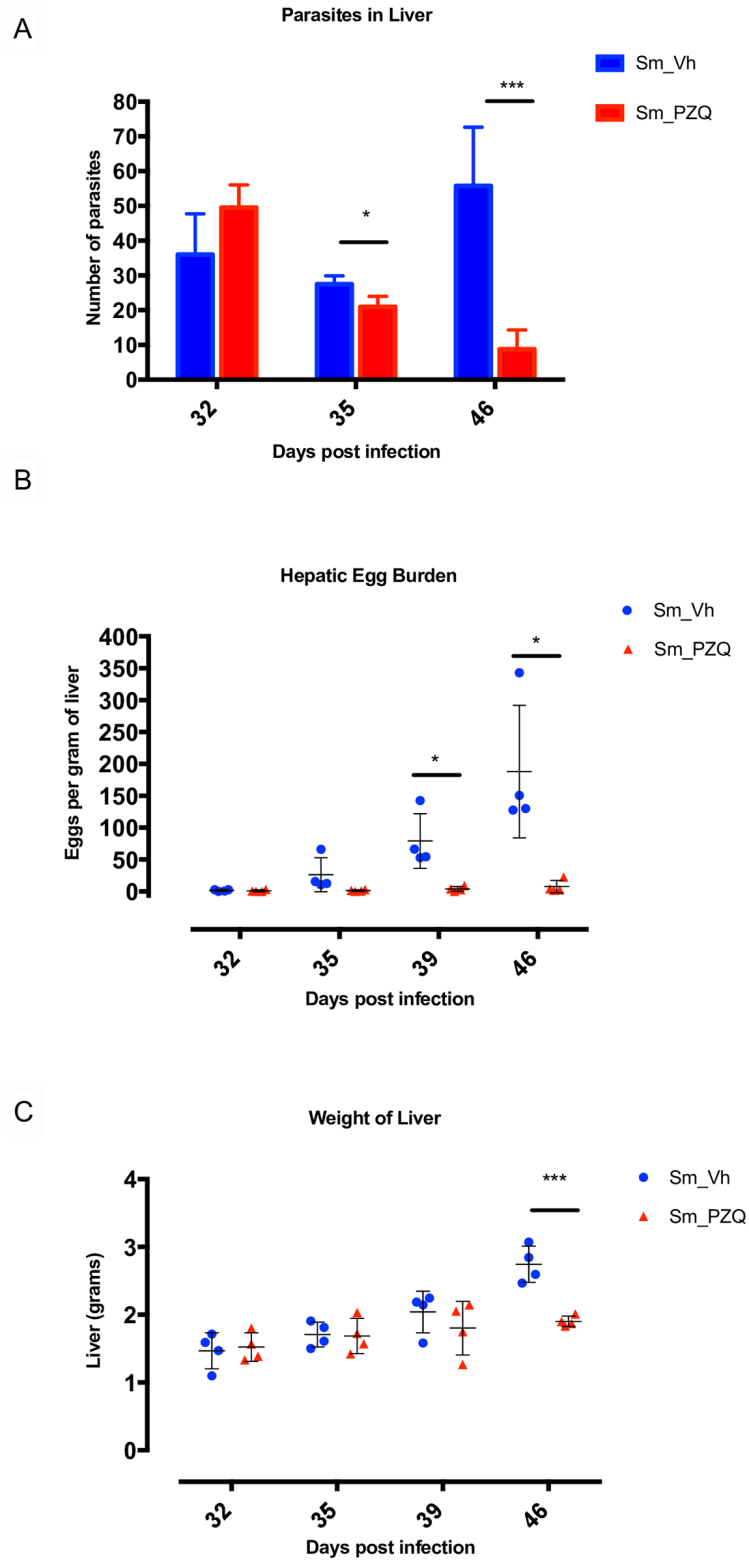
The effects of PZQ on infected host liver. (A) Effect of treatment with Vh and PZQ on the number of parasites present in host livers at days 32, 35 and 46 post infection, n = 5 per treatment group. (B) Number of parasite eggs per gram of liver at days 32, 35, 39 and 46 post infection after treatment with Vh and PZQ (n = 4). (C) Weight of liver at days 32, 35, 39 and 46 post infection after treatment with Vh and PZQ (n = 4). Error bars represent mean with standard deviation. * p <0.05 and *** p < 0.001.

For these studies mice were infected with approximately 150 *S*. *mansoni* cercariae. This level of infection will usually result in around 40–60 worms reaching maturity with the host becoming debilitated on or around days 49 to 56 post infection. Preliminary studies were conducted that identified the 32^nd^ day after infection as the optimal time-point to begin PZQ treatment. At this stage, infection was still relatively benign as few if any eggs had been deposited in the host liver with minimal signs of animal distress or hepatic damage, yet the majority of worms had matured sufficiently to have acquired sensitivity to the drug. Administering 250 mg/kg PZQ on day 32 resulted in no immediate effect on the number of live schistosomes retrieved by dissection from the liver 3 h after treatment compared with Vh treated infected mice (Fig 1A). After four consecutive daily doses totaling 1000 mg/kg there was a small but significant (p <0.05) drop in the number of live worms retrieved from PZQ treated (22.4 ± 2.8) compared to Vh treated (29.1 ± 1.8) mice. This difference was greater still (p <0.001) when mice were sacrificed 14 days (day 46) after the initial treatment when limited numbers of *S*. *mansoni* were retrieved from the liver of PZQ (8.3 ± 5.2) compared with Vh (54.3 ± 17.4) treated mice. The hepatic egg burden was negligible in controls and drug treated mice at days 32 and 35 but had increased significantly (p <0.05) in Vh compared with PZQ treated mice on days 39 and 46 (Fig 1B). Treatment with PZQ resulted in little accumulation of eggs in the liver during the course of the experiment, however, the number of eggs deposited in the liver of Vh treated mice reached its maximum on day 46 coinciding with a significant (p <0.001) increase in liver weight compared to PZQ treated mice (Fig 1C).

### Transcriptome sequencing, assembly and analysis

To compare changes in the hepatic transcriptome, cDNA libraries were prepared from the livers of 24 *S*. *mansoni* infected mice treated with PZQ or Vh, as well as 12 uninfected mice treated with a weight related dose of Vh (S1 Fig). After the removal of adaptor sequences and any ambiguous or low quality reads (Q <20), a total of approximately 920 million 50 base pair single-end reads were obtained (S4 Table). The mean number of reads in each of the 12 treatment groups (n = 3 per group) varied between 23.48 and 27.78 million. Sequence reads were assigned to the *Mus musculus* genome assembly and 88.2–91.0% of individual library reads with means of 88.9–90.3% for each group were successfully mapped. The number of aligned reads per sample was more than sufficient for differential expression analysis with treatment groups each containing three biological replicates [35]. In contrast, only a mean of between 0.01% (uninfected liver from Vh treated mice on days 39 and 46) and 0.13% (infected liver from Vh treated mice on days 32 and 35) of reads mapped to the *S*. *mansoni* genome. While the former number likely represents transcripts of high homology between mouse and parasite as no *S*. *mansoni* transcripts should be present, the latter was not sufficiently robust to allow for inferences to be made regarding the expression of *S*. *mansoni* genes during Vh or PZQ treatment.

**Fig 2:**
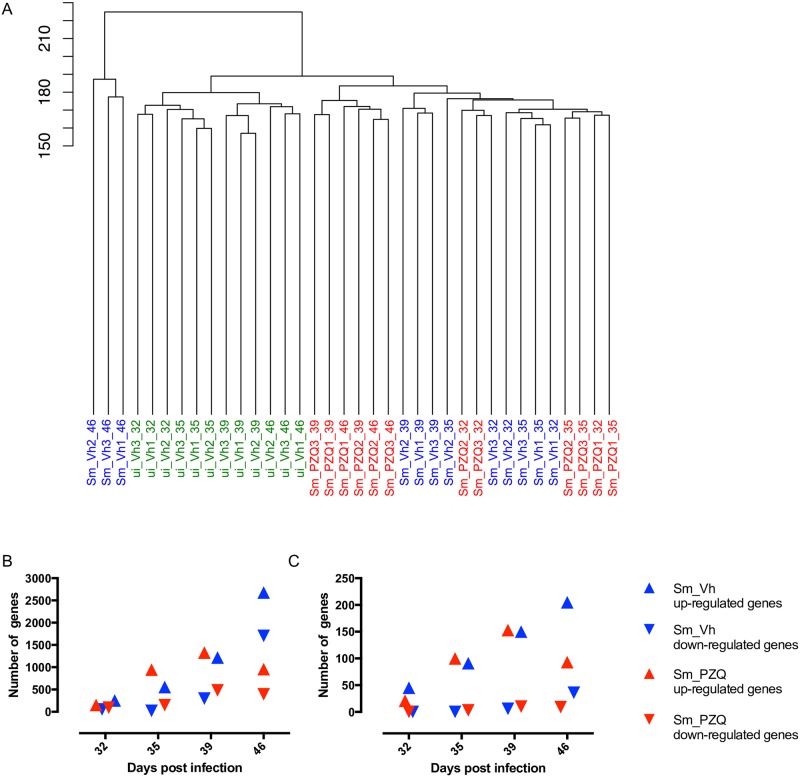
Hierarchical clustering of gene expression between different treatment groups and differentially expressed genes. (A) Dendrogram showing global similarities between hepatic transcriptomic replicates (n = 3) of uninfected Vh treated (ui_Vh), infected Vh treated (Sm_Vh) and infected PZQ treated (Sm_PZQ) mice at days 32, 35, 39 and 46 post infection. (B) The total number of up- and down-regulated hepatic genes in Vh and PZQ treated mice compared to uninfected Vh treated control samples at days 32, 35, 39 and 46 post infection. (C) The total number of up- and down-regulated immune genes identified by GO analysis in Vh and PZQ treated mice compared with uninfected Vh treated control samples at days 32, 35, 39 and 46 post infection.

When the transcriptomes of the 36 individual mouse livers were compared globally, they segregated into three distinct clusters with the 12 uninfected Vh samples grouped together (Fig 2A). Of the infected individuals, the day 32, 35, 39 and 46 transcriptomes from PZQ treated mice grouped together with the day 32, 35 and 39 transcriptomes from Vh treated mice with a high degree of sub-clustering generally coincident with the time and type of treatment. The six day 39 and 46 PZQ treated mice formed a separate sub-cluster indicating their transcriptomes were distinct from the others within this larger subset. The three day 46 Vh samples formed a distinct third cluster and were more similar to each other than any other samples, likely reflecting an expanded gene response to increasing egg deposition in these mice and the ensuing hepatic damage. All three biological replicates associated with each individual treatment and time grouped closely together, suggesting consistency between samples and none were omitted from further analyses. PCA was used to further characterize the relationship between the treatment groups (S3 Fig) and confirmed the internal structure of the dendrogram (Fig 2A) and that conditions for the day 46 Vh samples accounted for most of the variation.

As it has already been established that PZQ has no significant effect on the uninfected mouse liver transcriptome beyond 3 weeks post drug delivery [36] the transcriptomes of the two infected treatment groups at each time point were normalized against the corresponding uninfected Vh treated samples and differentially regulated genes were then identified. While there is no discernible difference in the numbers of differentially regulated genes between the treatment groups at days 32, 35 and 39, day 46 revealed a substantial increase in the number of both up- and down-regulated genes in Vh compared with PZQ treated mice (Fig 2B and S5 Table). Gene Ontology (GO) analysis revealed that while the number of differentially regulated genes increased with each time point regardless of treatment, the proportion of each biological process did not alter significantly (S4 Fig). The identity of all differentiated genes at each time point and treatment as well as their fold change is shown in S6 Table. The number of up-regulated genes characterized as ‘immune response related’ by GO analysis increased over the first three time points irrespective of treatment while the number of down-regulated genes was consistent (Fig 2C and S5 Table). At day 46, however, the number of up-regulated genes in the PZQ samples decreased in comparison to day 39. In contrast, the number of up- and down-regulated immune genes in the Vh samples increased. These results suggest that when viewed globally, PZQ treatment has little impact on the host’s immune response to infection until after day 39 when egg accumulation became more pronounced in Vh treated animals.

### Expression analysis of immune and fibrotic transcripts

**Fig 3:**
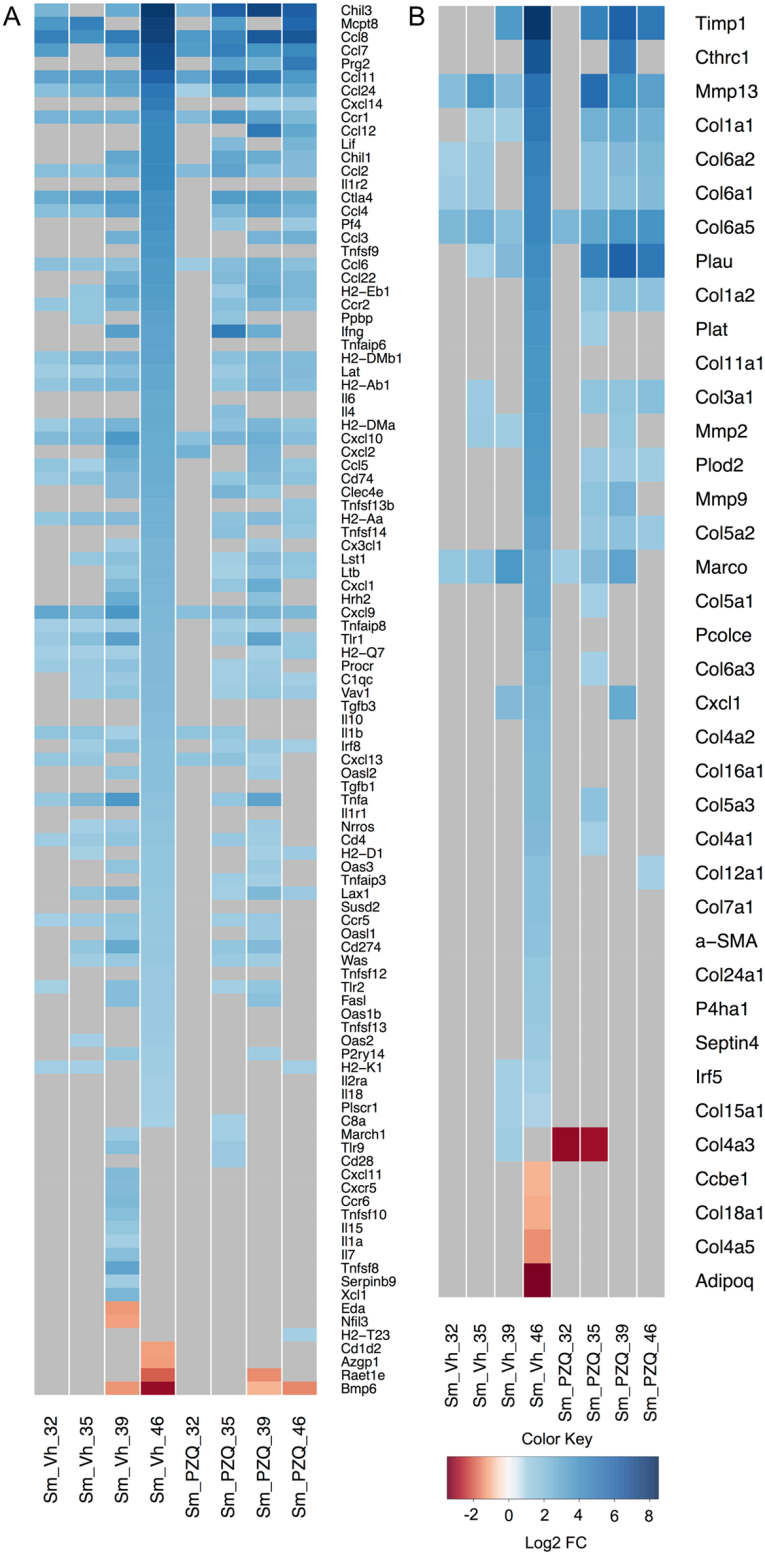
Temporal expression of mouse hepatic genes during infection with *S*. *mansoni*. Heat maps of (A) immune and (B) fibrotic gene markers depicting differentially expressed genes in infected Vh and PZQ treated mice relative to uninfected mice. Gray map sections represent genes not expressed at that time point or in that treatment group. Regions of blue and red indicate, relative to uninfected Vh treated controls, increased and decreased gene expression respectively. The color scale indicates log_2_ fold change (FC) and the profile of each group is the average of three biological replicates. Gene names associated with the figure differ slightly from those used in the text. Relevant differences include: Ccl (rather than CCL as it appears in the main body of text), Cxc (CXC), Cxcl (CXCL), Il1b (IL1β), Tnfa (TNFα), Tgfb (TGFβ), Ifng (IFNγ), Cd (CD) and Timp (TIMP).

To identify the response of individual genes across treatment and time in greater detail, heat maps were constructed for differentially regulated immune/inflammatory and fibrotic genes (Fig 3). Of the classic inflammatory cytokines, IL1β was up-regulated at days 32 through 46 in Vh samples but was only induced at days 32 and 35 with PZQ treatment. IL6 was only found to be up-regulated in day 46 Vh samples and TNFα was up-regulated at days 32 through 46 with a peak of activity at day 39 in Vh samples, while treatment with PZQ resulted in peak at day 39 but this abruptly disappeared at day 46. (Fig 3A).

Previous studies of the immune response to chronic schistosome infection have indicated that a Th1 response against migrating schistosomulae as well as maturing and mature schistosomes during the first 28–42 days is followed by a Th2 response driven by egg deposition and antigen release from the maturing miracidium contained therein [reviewed by 5]. In this study, Th1 related transcripts, including STAT1, STAT4 and Tbet, generally increased their expression from day 32 onwards compared to uninfected controls after Vh treatment (S5A Fig). Of the classical Th1 markers, only IFNγ was increased significantly and only at days 39 and 46 (Fig 3A and S5A Fig). In contrast, PZQ treatment led to an increase in expression of all four Th1 markers at days 35 and 39 though again only IFNγ changed significantly and at both time points with all falling back to initial expression levels at day 46 (Fig 3A and S5B Fig). Th2 related cytokine and chemokines IL4, CCL12 and CCL22 were elevated most significantly at day 46 in Vh samples but their activities peaked earlier after PZQ treatment (Fig 3A and S5C and S5D Fig). TGFβ, a negative immune regulator, was only elevated in Vh samples and only at day 46 (Fig 3A).

Burke and colleagues examined the temporal expression of immune (and fibrotic) genes in *S*. *japonicum* infected mice [7]. These animals were more lightly infected than reported here and gene expression profiles were assessed using microarrays on days 28, 42 and 49 post infection. While granulomas formed as a result of *S*. *japonicum* infection have been described as more severe and neutrophilic than those associated with *S*. *mansoni* [37], which are more eosinophilic [38], we observed many of the same transcriptional patterns during the initiation and establishment of immune responses. Burke and colleagues noted the early onset of Th1 associated gene expression (i.e., IFNγ, STAT1 as well as CXCL9) peaked at days 28–42 with increases in expression of Th2 chemokines (i.e. CCL7 and CCL24) peaking at day 42; a similar pattern of expression was observed for these genes in this study (Fig 3A). Indeed, a number of CC and CXC chemokine transcripts were observed in both studies to be elevated at most time points sampled. For example, the early and sustained elevation of T cell attractants CCL8 and CXCL9 and 10 in both treatment groups as well as the concomitant induction of CD4 agrees with observations in *S*. *japonicum* [7] and supports the notion that Th recruitment begins independently of egg deposition. Induction of the B cell cytokine CXCL13 was also early and sustained, but no change in the B cell marker CD19 was observed, suggesting no significant recruitment of B cells into the liver as a result of worm infection or egg deposition.

**Fig 4:**
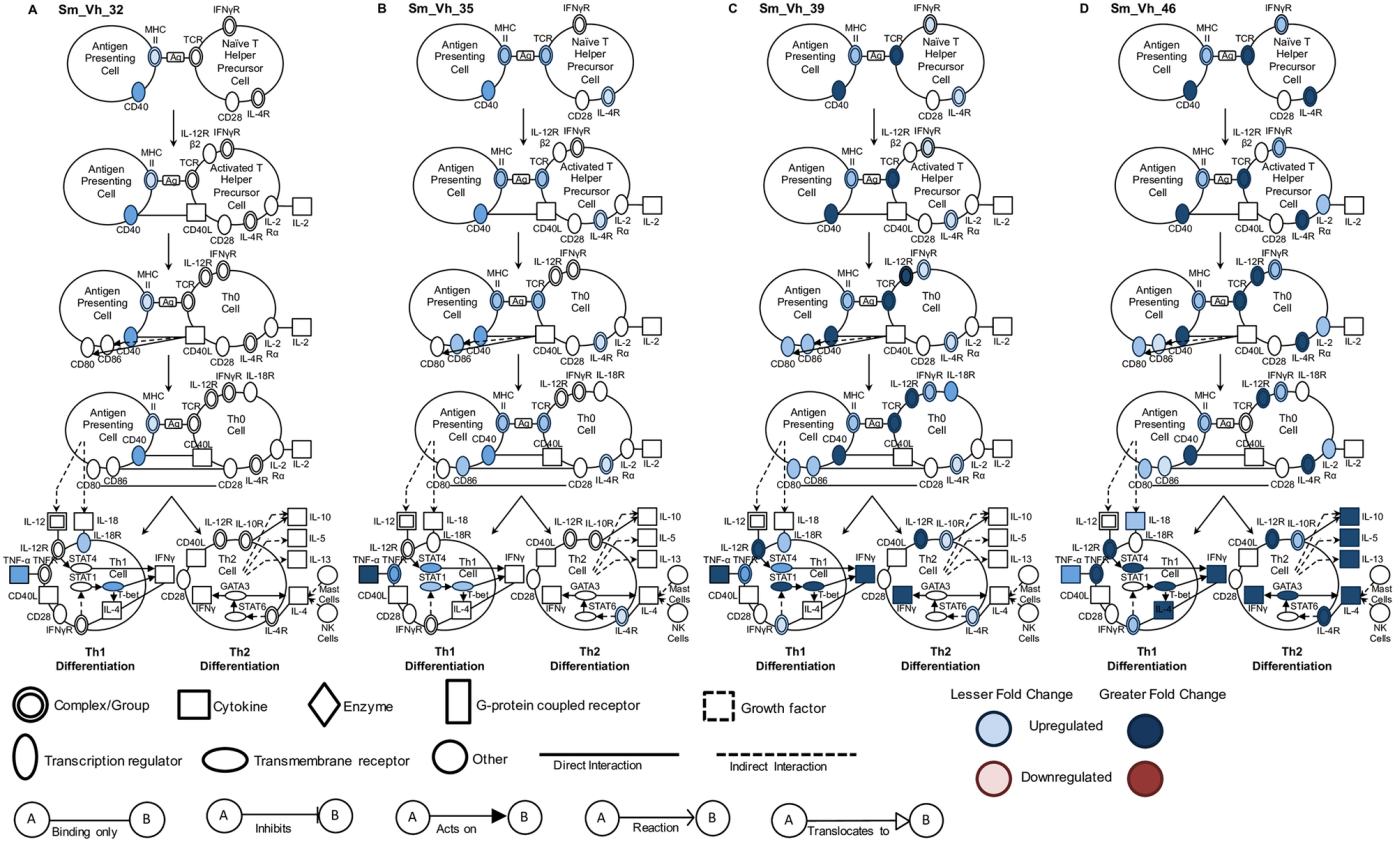
Canonical pathway analysis of T helper cell maturation and differentiation in vehicle treated infected mice. Signaling events in the T cell maturation and differentiation pathway at (A) 32, (B) 35, (C) 39 and (D) 46 days in infected Vh treated (Sm_Vh) mice. Increasing expression in infected mice relative to uninfected mice is indicated by deeper blue shading. None of the genes indicated were down-regulated. Non-expression and non-differential expression is indicated by a lack of shading.

**Fig 5:**
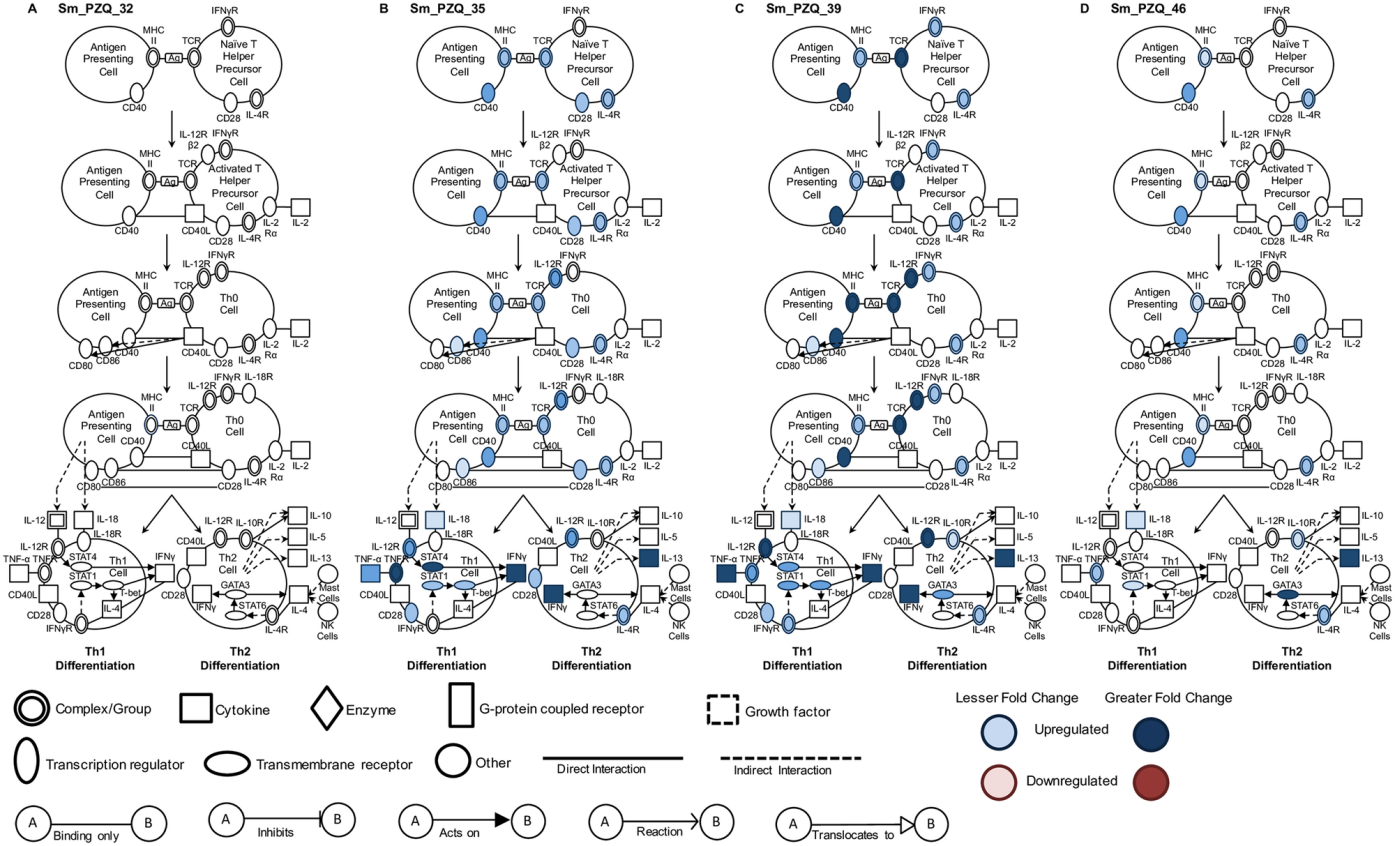
Canonical pathway analysis of T helper cell maturation and differentiation in PZQ treated infected mice. Signaling events in the T cell maturation and differentiation pathway at (A) 32, (B) 35, (C) 39 and (D) 46 days in infected PZQ (Sm_PZQ) treated mice. Increasing expression in infected mice relative to uninfected mice is indicated by deeper blue shading. None of the genes indicated were down-regulated. Non-expression and non-differential expression is indicated by a lack of shading.

Although this approach gives insight into the differential expression of key individual genes, it does not provide a global overview of the effect of infection, treatment and time on T cell gene networks controlling maturation and differentiation. We used Ingenuity Pathway Analysis to visualize this complexity and two pathways that best encapsulate this data, representing T cell maturation/differentiation and fibrosis, are discussed. Vh treatment results in T cell maturation pathway activity that was likely initiated before day 32 and increased progressively until day 46 (Fig 4A–4D). While a number of key genes involved in Th1 differentiation are progressively up-regulated between days 32 and 46, gene regulation leading to Th2 differentiation is only apparent at days 39 and 46. Administration of PZQ did not result in any maturation or differentiation gene activity at day 32, but after completion of treatment, multiple genes in both the maturation and Th1 and Th2 differentiation pathways were substantially up-regulated at day 35 and peaking at day 39 (Fig 5A–5D). At day 46, however, the breadth and intensity of these responses declined precipitously.

Burke and colleagues noted the early and sustained up-regulation of the eosinophil chemo-attractants CCL11 and CCL24 [7] and we also observed this pattern in both treatment groups. It is noteworthy that in the absence of PZQ treatment, CCL11 and CCL24 expression was especially pronounced on day 46, whereas in the presence of the drug, expression of both cytokines peaked earlier (Fig 3A). As noted above, eosinophils have a significant presence in *S*. *mansoni* induced granulomas [38] and our data confirms they play a role in both the initial anthelmintic response as well as establishment of fibrosis. As discussed above, treatment with Vh resulted in a significant accumulation of parasite eggs in the livers of infected animals at day 46. Egg accumulation in turn led to development of granulomas with significant fibrotic pathology (comparing S6A and S6C Fig). For hepatic fibrotic markers there was again substantial differential regulation of individual genes in day 46 Vh samples (Fig 3B). Many of this subset were members of the collagen gene family including *Col1a1* and *Col3a1* that encode type I and III collagens respectively. Histological examination of Vh day 46 liver sections using Picrosirius Red highlighted the deposition of type I and type III collagen in the surrounding matrix of the granulomas (comparing S6B and S6D Fig). Other genes with peak expression coinciding with the fibrotic response in Vh treated animals included those encoding matrix metalloproteinases (Mmp) 2, 9 and 13 as well as tissue inhibitors of matrix metalloproteinases (TIMP) 1 but not TIMP 2. These transcripts have previously been shown to be abundant in *S*. *mansoni* and *S*. *japonicum* induced fibrosis [6, 39, 40]. A number of genes including *Col1a1*, *Col3a1*, *Mmp2*, *9* and *13* as well as *TIMP1* were induced after treatment with PZQ and in the absence of egg deposition or gross fibrosis suggesting that their expression is also driven by the breakdown of *S*. *mansoni* after drug treatment.

**Fig 6:**
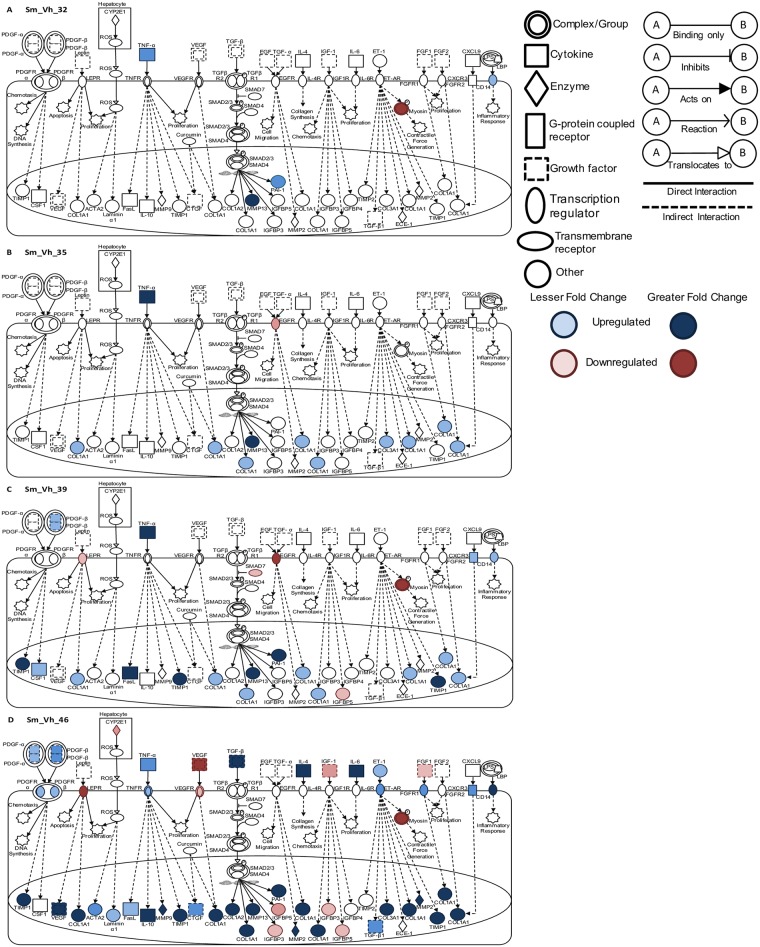
Canonical pathway analysis of hepatic fibrosis and stellate cell activation in vehicle treated infected mice. Signaling events in the fibrotic and stellate cell activation pathway at (A) 32, (B) 35, (C) 39 and (D) 46 days post infection in infected Vh treated (Sm_Vh) mice. Increasing expression in infected mice relative to uninfected mice is indicated by deeper blue shading. Decreased expression is shown in dark red. Non-expression and non-differential expression is indicated by a lack of shading.

**Fig 7:**
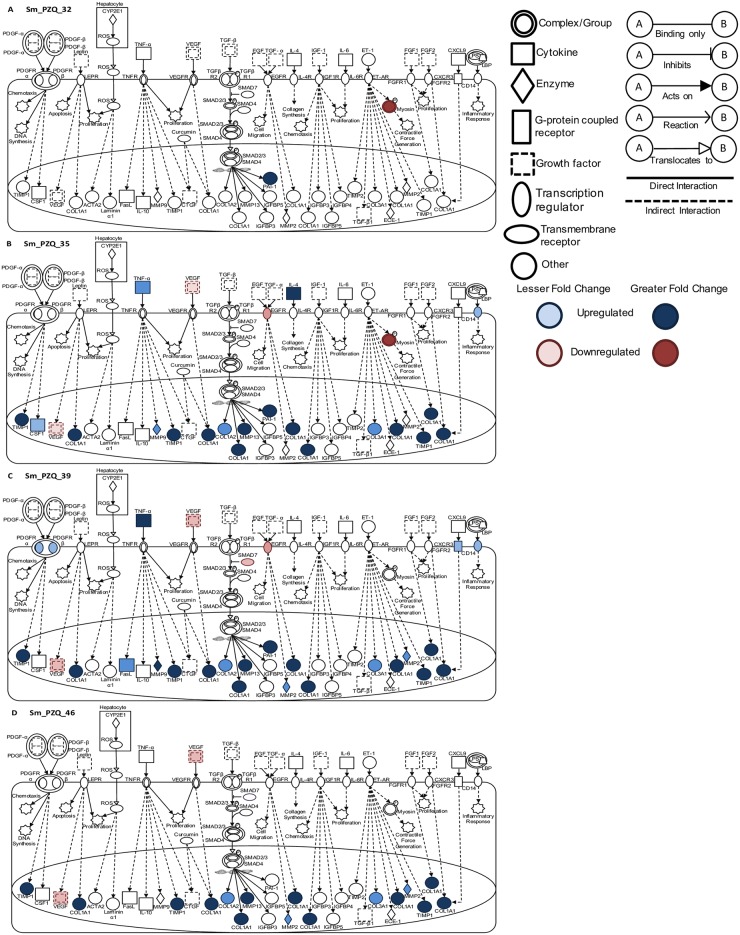
Canonical pathway analysis of hepatic fibrosis and stellate cell activation in PZQ treated infected mice. Signaling events in the fibrotic and stellate cell activation pathway at (A) 32, (B) 35, (C) 39 and (D) 46 days post infection in infected PZQ (Sm_PZQ) treated mice. Increasing expression in infected mice relative to uninfected mice is indicated by deeper blue shading. Decreased expression is shown in dark red. Non-expression and non-differential expression is indicated by a lack of shading.

Pathway analysis was employed to provide a global overview of the differential expression of genes previously identified as being involved in early signaling in hepatic stellate cells, the major cell type involved in liver fibrosis [41]. Thirty-two days after infection and three hours after treatment with PZQ or Vh, there was no substantial differential expression of genes involved in fibrotic pathways (Figs 6A and 7A). Thereafter, treatment with Vh was associated with a gradual increase in differential expression of numerous genes across fibrotic networks (Fig 6B–6D). In contrast, treatment with PZQ led to an earlier more pronounced increase in fibrotic markers at day 35 on cessation of treatment and this level of activity was maintained until day 46 (Fig 7B–7D). Examination of these pathways at 46 days post infection revealed a 10-fold expansion in the number of genes across multiple pathways that are differentially regulated irrespective of treatment. A comparative analysis of different treatments at this time point revealed that Vh treatment led to a greater diversity and a more pronounced expression of genes across multiple fibrotic signaling pathways. These observations suggest that treatment with PZQ coincident with female parasite sexual maturation and egg release dampens but does not eliminate the occurrence of hepatic fibrotic events.

**Fig 8:**
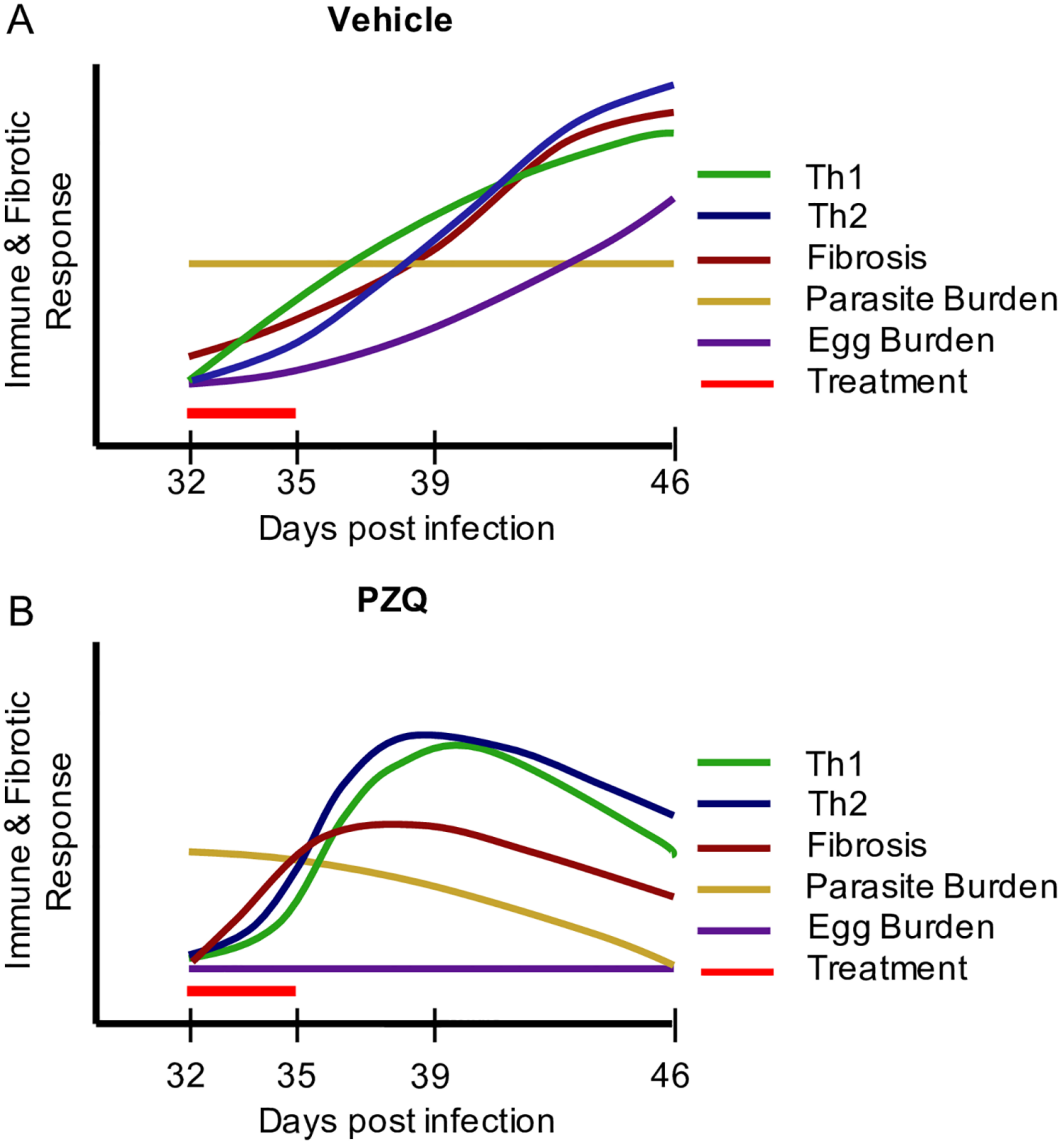
Model of Th1, Th2 and fibrotic responses during *S*. *mansoni* infection and treatment. A global representation of Th1, Th2 and fibrotic gene responses during *S*. *mansoni* infection at 32, 35, 39 and 46 days post infection in (A) Vehicle and (B) PZQ treated mice.

Fig 8A and 8B summarizes Th1, Th2 and fibrotic gene responses in relation to worm presence and/or elimination and egg burden during and after Vh or PZQ treatment. As anticipated, markers of Th1 and Th2 immune function and hepatic fibrosis rise with time in response to increasing hepatic egg burden and perhaps continued parasite presence (Fig 8A). With PZQ treatment, expression of T cell networks peaks earlier as drug treatment eliminates the majority of parasite burden (Fig 8B). Surprisingly, some fibrotic markers also rise in the absence of egg burden, perhaps in response to mechanical breakdown of parasites in the liver although no visible fibrosis was present.

Results obtained from NGS analysis were validated by qRT-PCR of six transcripts (CCL7, Col1a1, Col6a5, IFNγ, IL1β and Krt4) over each time point for samples from both Vh and PZQ treated animals (S7 Fig). Patterns observed in the qRT-PCR data mirrored those obtained by RNA-Seq and showed a significant correlation (S8 Fig) between the two across both treatments (Vh: r = 0.70, p <0.0001; PZQ; r = 0.61, p <0.0001).

### *S*. *mansoni* gene expression *in vivo*

It had been our intention originally to investigate changes in the *S*. *mansoni* transcriptome using the RNA-Seq data generated from infected mouse livers. As outlined above and in S4 Table, too few reads were generated to provide a robust data set and we decided to use the *S*. *mansoni* RNA contained within the hepatic samples as template for qRT-PCR analysis of individual genes of interest, especially those encoding ABC transporters. In a review of the ABC transporter family in *S*. *mansoni* and *S*. *japonicum*, Greenberg reported the presence of 21 and 19 family members in each species respectively [42]. He also matched existing transcripts in the schistosome and NCBI gene and transcript databases with their human homologs and we have relied on an expansion of that nomenclature to identify the transcripts quantified to avoid confusion (S3 Table).

**Fig 9:**
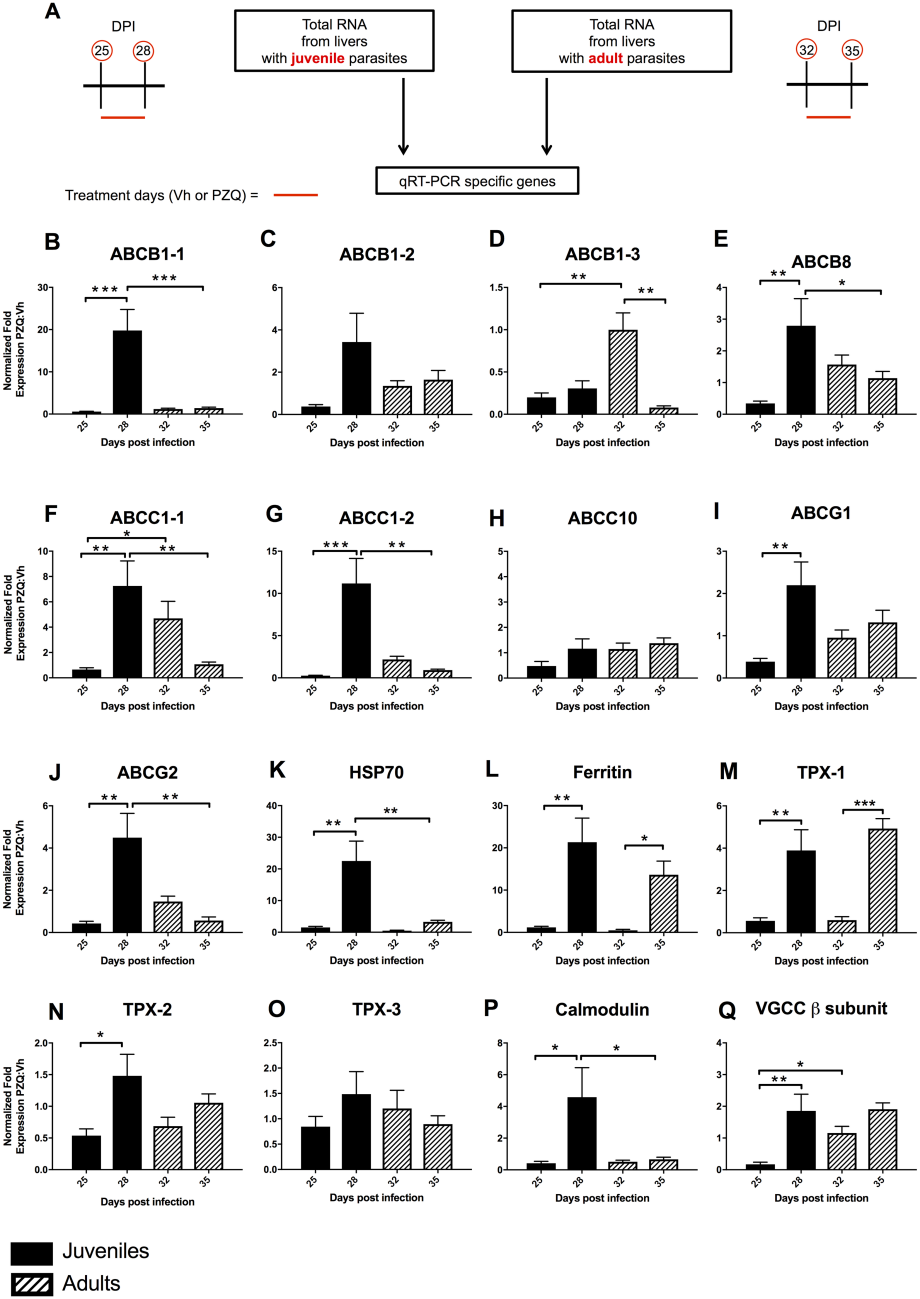
Quantitative real-time PCR analysis of *S*. *mansoni* gene expression. Results are normalized to *S*. *mansoni* GAPDH and fold change in gene expression in PZQ treated schistosomes is shown relative to Vh treated groups. (A) Schematic diagram explaining the source of *S*. *mansoni* RNA for qRT-PCR analysis. (DPI = days post infection). B-Q. Gene expression in juvenile schistosomes at days 25 and 28 is shown with shaded bars and adults at days 32 and 35 are shown hatched. (B-D) and (F-H) show the response of full ABC drug transporters while (E), and (I, J) are half transporters each with homology to a member of the human B, C and G families. (K-O) show the response of stress genes HSP70, ferritin and thioredoxin peroxidase (TPX-1, 2 & 3), (P) the response of calmodulin and (Q) response of voltage-gated Ca^2+^ channel β subunit respectively. Statistical differences in fold change between adult and juvenile *S*. *mansoni* were assessed using one-way ANOVA followed by Fisher’s least significant difference post hoc test for multiple comparisons. Error bars represent +1 standard error of the mean. * p <0.05, ** p <0.01, *** p <0.001.

While we and others have examined the effect of PZQ on a limited number of ABC transporters in *S*. *mansoni* treated *ex vivo*, this, as far as we are aware, is the first study to examine a suite of transporters during drug treatment *in vivo*. As there is also the potential that differences in ABC transporter expression may underpin juvenile resistance to PZQ, we extended our experiments to parasite RNA extracted from livers derived from mice treated with PZQ and Vh on days 25–28 of infection (Fig 9A). Fig 9B–9J shows the differential expression of nine transporters in juvenile and adult *S*. *mansoni* 3 h after the first and fourth doses of PZQ or Vh. After four consecutive days of PZQ treatment there was a significant increase in expression of ABCB1-1, B8, C1-1, C1-2, G1 and G2 transcripts in juvenile schistosomes. No ABC transporter gene showed a reduction in the level of transcription. In contrast, no ABC transporter gene showed an increase in transcription in adult worms exposed to PZQ for 4 consecutive days while ABCB1-3 showed a significant fall. ABCB1-3 and C1-1 showed a significantly higher level of activity in adult compared to juvenile worms on the first day of treatment with no juvenile genes showing greater activity. In contrast, when comparing gene expression between juvenile and adult worms on the fourth day of treatment, genes encoding ABCB1-1, B8, C1-1, C1-2 and G2 all show significantly greater levels of activity in juveniles with no gene activity significantly greater in adult than juvenile worms. Essentially, our data indicates that treatment with a ‘lethal’ dose of PZQ over a 4 day period leads to a significant increase in transcription associated with several ABC transporter genes belonging to the ABCB, C and G families in juvenile but not adult parasites. These families are especially noteworthy as some members (ABCB1, C1 and G2) are strongly associated with the transport of xenobiotics and multi-drug resistance in cancer treatment [43]. Our data is not in complete agreement with several published studies that have investigated the response of individual *S*. *mansoni* transporters to PZQ. Schistosome ABCB1-1 transporter (also named SMDR2) and ABCC1-1 (also named SmMRP1) transcript and protein levels have been shown to be transiently increased in adult schistosomes following exposure to a sub-lethal dose of PZQ *in vitro* [19, 20] while the drug has also been found to be a substrate of ABCB1-1 when this transporter is expressed in CHO cells [21]. Kasinathan and colleagues also demonstrated that *S*. *mansoni* ABCB1-1 and C1-1 were expressed at higher levels in juveniles compared with adult worms and that adult males, but not females show a significant increase in the level of ABCC1-1 on exposure to a sub-lethal dose of PZQ [20]. They suggested this data was ‘..consistent with the hypothesis that increases in levels of schistosome multidrug transporters may be involved in development or maintenance or reduced susceptibility to PZQ’. This hypothesis was further supported by additional data reported by Kasinathan and colleagues in which juvenile and adult *S*. *mansoni* ABC transporter activities were inhibited pharmacologically in the presence of sub-lethal PZQ concentrations *ex vivo* [23]. With adult worms, these conditions resulted in an exacerbation of the effects of PZQ including a loss of motility and tegument disruption, while juveniles became paralyzed when PZQ doses that they would normally prove refractory to were applied. In contrast, adult worms in which expression of five ABC transporters including ABCB1-1 and C1-1 were knocked down showed increased responsiveness to PZQ. This *ex vivo* data taken together with the results reported here, lends further support to the hypothesis that ABC transporter family members play a significant role in modulating the response of juvenile and adult schistosomes to PZQ and underpin juvenile resistance.

In addition to nine ABC transporters, the expression of seven other *S*. *mansoni* genes was examined. Three of these (HSP70, ferritin and thioredoxin peroxidase 1) were selected (Fig 9K–9M) as we observed previously that exposure of *S*. *mansoni* to PZQ results in induction of these stress response genes [17, 22]. This observation was broadly repeated in this study, with all 3 genes being induced significantly in juveniles with ferritin and thioredoxin peroxidase 1 being induced significantly in adult after four days of treatment suggesting that both juveniles and adults were still alive at this time. The expression of two other potential *S*. *mansoni* thioredoxin peroxidase homologs (TPX-2 and TPX-3) were also quantified but only juvenile TPX-2 was found to be differentially expressed after treatment (Fig 9N and 9O). Finally, as schistosomes exposed to PZQ have been demonstrated to undergo a Ca^2+^—dependent contraction that appears to be mediated by a voltage gated Ca^2+^ channel [44], we also measured expression of a schistosomal calmodulin and voltage gated Ca^2+^ channel β subunit transcripts. Binding of Ca^2+^ is essential for the activation of calmodulin which functions as a regulator of many different proteins and pathways. Calmodulin and Ca^2+^ channel β subunit transcript expression was induced significantly in juveniles after 4 days of treatment (Fig 9P and 9Q), however, neither were differentially regulated in adults.

In conclusion, the analysis of livers from mice infected with *S*. *mansoni* and treated with PZQ or its Vh enabled a comprehensive study of the effect of the drug on anthelmintic transcriptomic responses in the presence and absence of developing granulomas. While the immune and fibrotic responses in the absence of the drug are significant leading to a severe pathology that will, in time, prove fatal for the animals, many of the same genes and gene networks are active in PZQ treated mice. Furthermore, we have demonstrated that several ABC transporters, especially members of the ABCB, C and G families, are transcriptionally enriched in juvenile compared to adult worms after exposure to PZQ. This lends further support to the hypothesis that differential expression of these transporters underpins the resistance of juveniles to the drug.

## Supporting information

S1 Fig Schematic diagram of mouse treatments to generate RNA-Seq data. Three distinct groups of 16 mice contributed to the RNa-Seq experiment. These included (A) uninfected mice treated with PZQ vehicle (Cremaphor EL) on days 32–35 post infection with 4 mice each being sacrificed on days 32, 35, 39 and 46 post infection; (B) *S*. *mansoni* infected mice treated with PZQ vehicle (Cremaphor EL) on days 32–35 post infection with 4 mice each being sacrificed on days 32, 35, 39 and 46 post infection and (C) *S*. *mansoni* infected mice treated with PZQ on days 32–35 post infection with 4 mice each being sacrificed on days 32, 35, 39 and 46 post infection.
(TIFF)

S2 Fig Representative Bioanalyzer traces of total RNA from murine livers with the corresponding RNA integrity number (RIN) values from (A) an uninfected vehicle treated, mouse; (B) a *S*. *mansoni* infected vehicle treated mouse and (C) a *S*. *mansoni* infected PZQ treated mouse.
(TIFF)

S3 Fig Two dimensional principal component analysis (PCA). Visualization of the clustering or scattering of hepatic transcriptome replicates.
(TIFF)

S4 Fig Biological process GO terms associated with differentially regulated genes during *S*. *mansoni* infection. Pie charts of the enriched biological processes for genes significantly up- and down-regulated at days 32, 35, 39 and 46 in infected PZQ and Vh treated mice. Data were generated using gene ontology (GO) analysis with a Bonferroni-adjusted p value <0.05.
(TIFF)

S5 Fig Change in expression of Th1 and Th2 markers as a result of Vh or PZQ treatment. (A) and (B) show the change in IFNγ, STAT1, STAT4 and TBET expression during and after treatment with Vh (Sm_Vh) and PZQ (Sm_PZQ) respectively. (C) and (D) show the change in expression of IL4, IL5, GATA3, CCL12, CCL17 and CCL22 expression during and after treatment with Vh (Sm_Vh) and PZQ (Sm_PZQ) respectively.
(TIFF)

S6 Fig Granuloma formation in the murine liver during *S*. *mansoni* infection. Picrosirius staining (PolySciences Inc., Washington, PA) was performed according to the manufacturer’s protocol to determine hepatic fibrosis progression. (A) Bright red stain around the two schistosome eggs in the center field indicates picrosirius dye binding to collagen fibrils within the granuloma. The section was taken from the liver of an infected mouse treated with Vh 45 days after infection. (B) The same field of view shown in (A) but under polarizing light. Yellow-orange birefringence indicates type I collagen fibers while green birefringence indicates type III. (C) and (D). Section from PZQ treated mouse liver 45 days after *S*. *mansoni* infection. No granuloma or collagen fibrils were evident. Slides were visualized on a Zeiss Axio Scope.A1 using a 20x objective and images were acquired with a Nikon D5200 Camera fitted with a MM-SLR Adapter. Scale bar = 130 μm.
(TIFF)

S7 Fig Validation of RNA-Seq gene expression data by quantitative real-time PCR (qRT-PCR). Log_2_ changes in expression of genes encoding chemokine Ccl7, collagen type I pro-α chain (col1a1), collagen type VI α5 chain (Col6a5), interferon γ (IFNγ) interleukin 1β (IL1β) and keratin 4 (Krt4) in Vh and PZQ treated infected mice at days 32, 35, 39 and 46 post infection. Gene expression was assessed by qRT-PCR (A, C)) and RNA-Seq (B, D) after Vh and PZQ treatment respectively. The gene expression profile at each point is the average of three biological replicates. For both RNA-Seq and qRT-PCR data, regions of red and green indicate gene expression has increased and decreased respectively.
(TIFF)

S8 Fig Correlation between RNA-Seq and quantitative real-time PCR (qRT-PCR). Relationship between RNA-Seq and qRT-PCR data for six genes over each time point and for both treatments was established using Spearman’s Rho correlation.
(TIFF)

S1 Table Primers used in *Mus musculus* quantitative real-time PCR reactions. GAPDH (ENSMUSG00000057666) primer set is part of the PrimePCR Probe Assay (BIORAD qMmuCED0027497).
(PDF)

S2 Table Real time PCR cycle threshold (Cq) raw data for *S*. *mansoni* reference gene GAPDH. (PDF)

S3 Table Primers used in *S*. *mansoni* quantitative real-time PCR reactions. (PDF)

S4 Table Summary of Illumina read counts for each sequenced sample. (PDF)

S5 Table Number of differentially regulated genes in Vh and PZQ treated samples. (PDF)

S6 Table Identities of all differentially expressed hepatic genes at each time point and treatment. (PDF)
