# Supplementary figures and images for: Effect of praziquantel on the differential expression of mouse hepatic genes and parasite ATP binding cassette transporter gene family members during Schistosoma mansoni infection
Source: PLoS Negl Trop Dis. 2017 Jun 26;11(6):e0005691. doi: 10.1371/journal.pntd.0005691 (PMC5501684; doi:10.1371/journal.pntd.0005691)

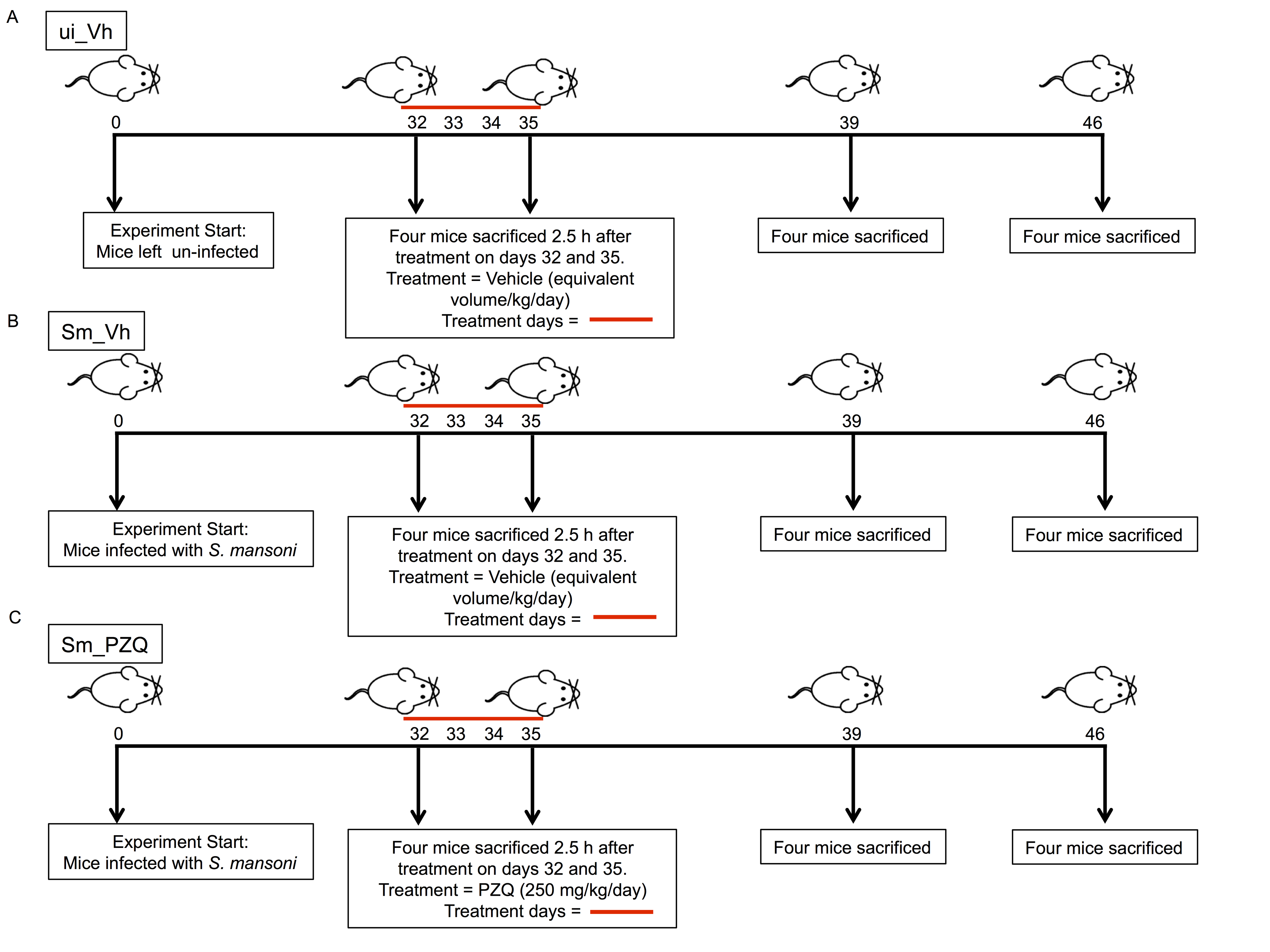

Supplement: S1 Fig — Three distinct groups of 16 mice contributed to the RNa-Seq experiment. These included (A) uninfected mice treated with PZQ vehicle (Cremaphor EL) on days 32–35 post infection with 4 mice each being sacrificed on days 32, 35, 39 and 46 post infection; (B) S. mansoni infected mice treated with PZQ vehicle (Cremaphor EL) on days 32–35 post infection with 4 mice each being sacrificed on days 32, 35, 39 and 46 post infection and (C) S. mansoni infected mice treated with PZQ on days 32–35 post infection with 4 mice each being sacrificed on days 32, 35, 39 and 46 post infection. (TIFF) [file pntd.0005691.s001.tiff]

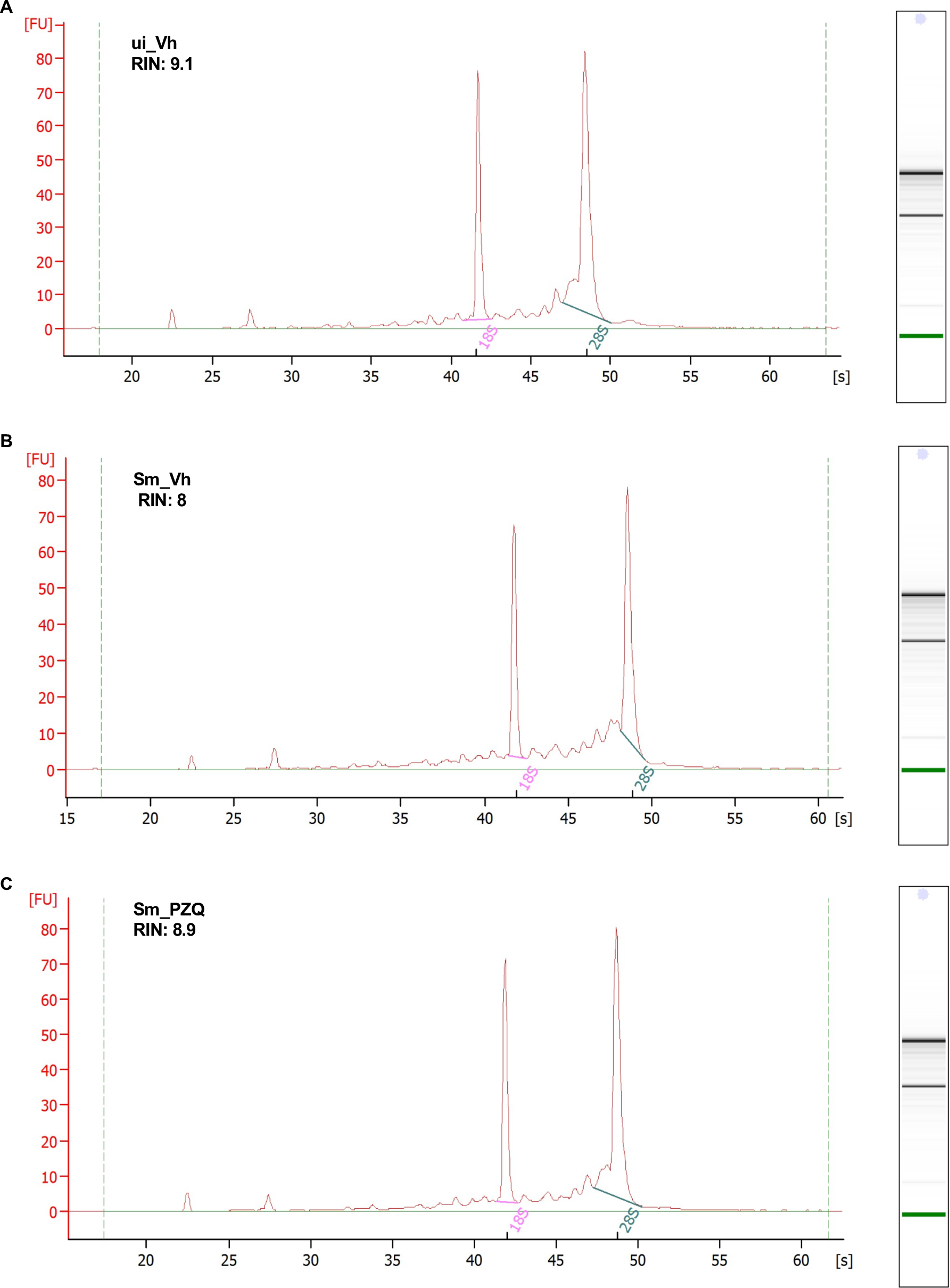

Supplement: S2 Fig — Representative Bioanalyzer traces of total RNA from murine livers with the corresponding RNA integrity number (RIN) values from (A) an uninfected vehicle treated, mouse; (B) a S. mansoni infected vehicle treated mouse and (C) a S. mansoni infected PZQ treated mouse. (TIFF) [file pntd.0005691.s002.tiff]

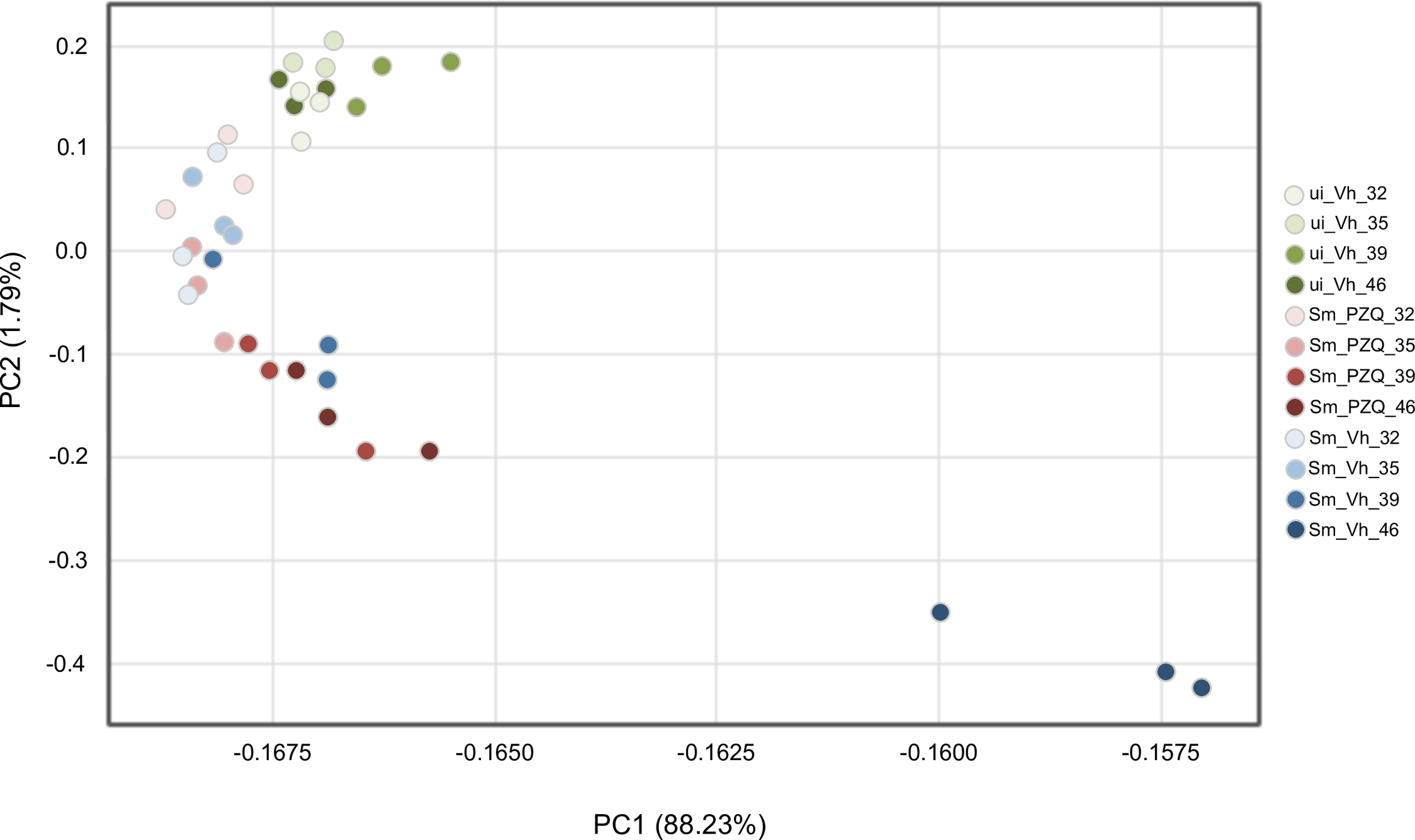

Supplement: S3 Fig — Visualization of the clustering or scattering of hepatic transcriptome replicates. (TIFF) [file pntd.0005691.s003.tiff]

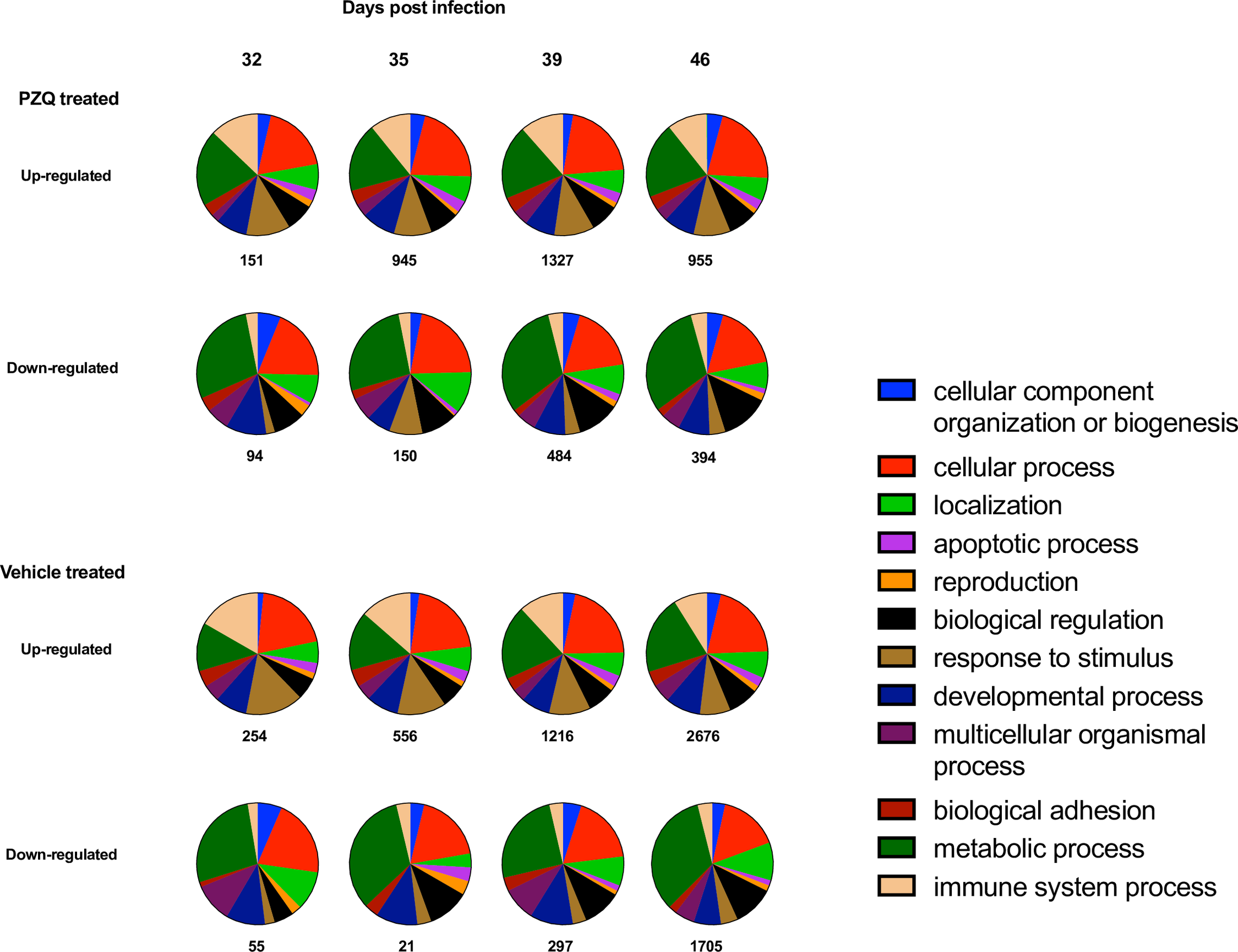

Supplement: S4 Fig — Pie charts of the enriched biological processes for genes significantly up- and down-regulated at days 32, 35, 39 and 46 in infected PZQ and Vh treated mice. Data were generated using gene ontology (GO) analysis with a Bonferroni-adjusted p value <0.05. (TIFF) [file pntd.0005691.s004.tiff]

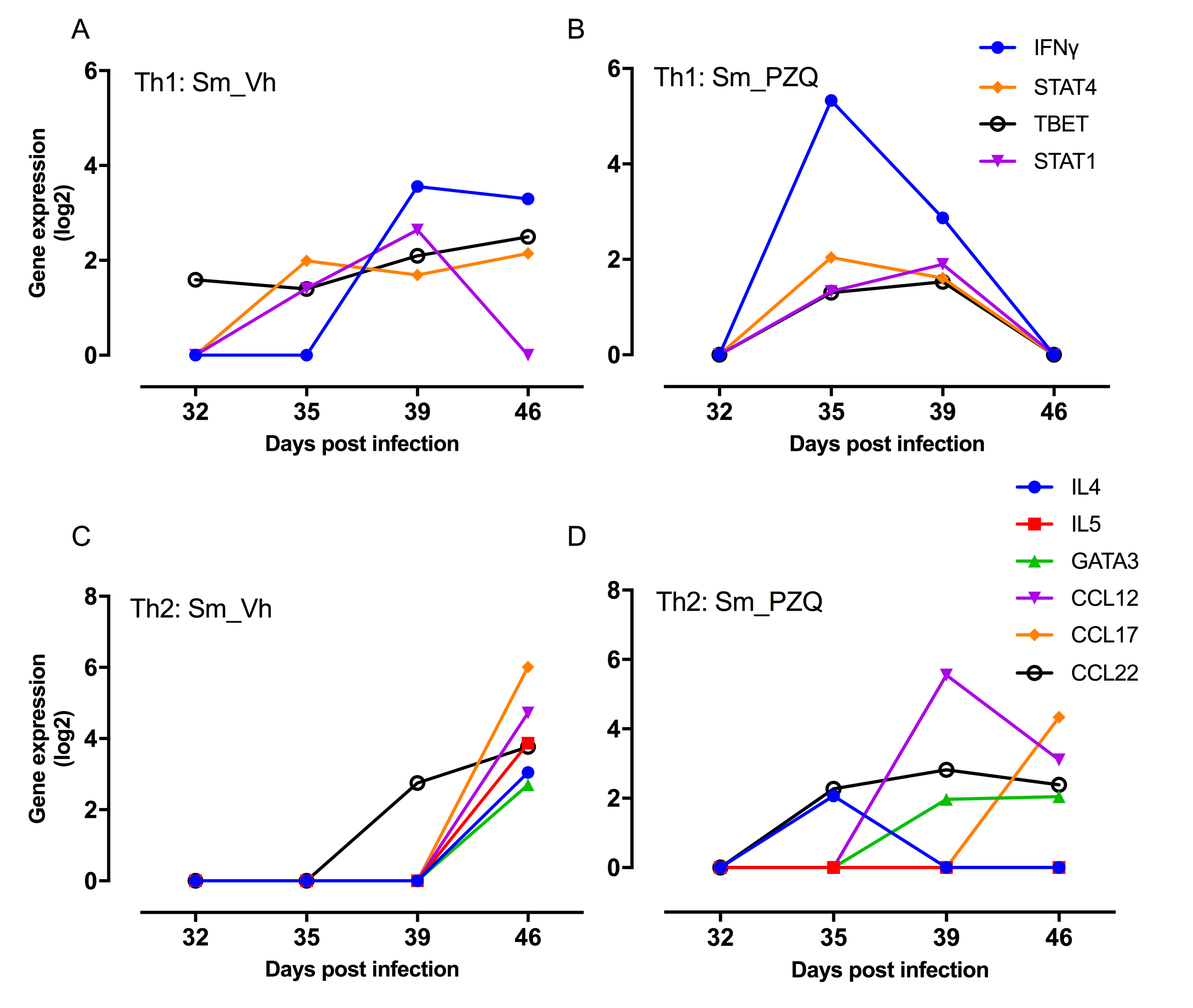

Supplement: S5 Fig — (A) and (B) show the change in IFNγ, STAT1, STAT4 and TBET expression during and after treatment with Vh (Sm_Vh) and PZQ (Sm_PZQ) respectively. (C) and (D) show the change in expression of IL4, IL5, GATA3, CCL12, CCL17 and CCL22 expression during and after treatment with Vh (Sm_Vh) and PZQ (Sm_PZQ) respectively. (TIFF) [file pntd.0005691.s005.tiff]

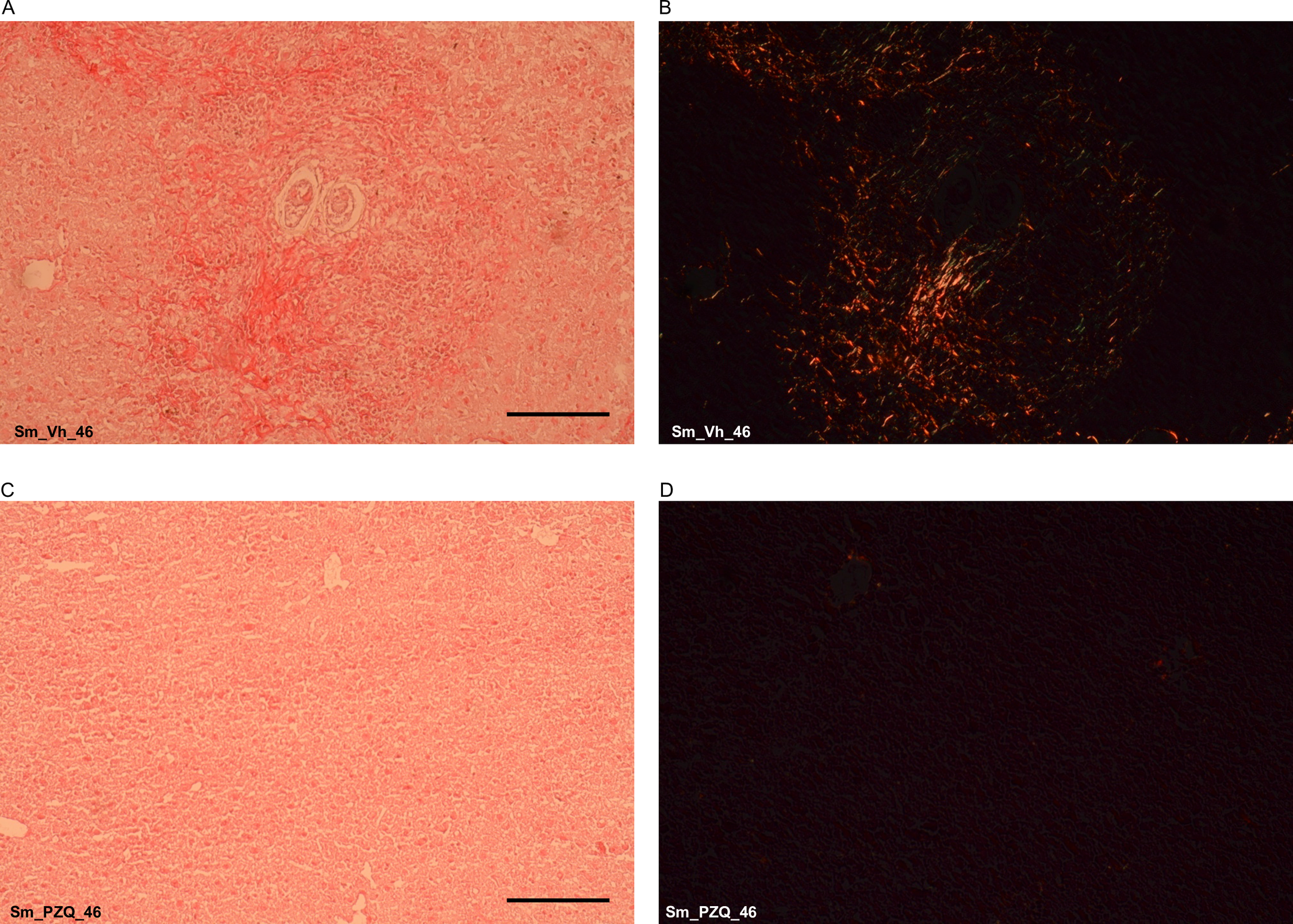

Supplement: S6 Fig — Picrosirius staining (PolySciences Inc., Washington, PA) was performed according to the manufacturer’s protocol to determine hepatic fibrosis progression. (A) Bright red stain around the two schistosome eggs in the center field indicates picrosirius dye binding to collagen fibrils within the granuloma. The section was taken from the liver of an infected mouse treated with Vh 45 days after infection. (B) The same field of view shown in (A) but under polarizing light. Yellow-orange birefringence indicates type I collagen fibers while green birefringence indicates type III. (C) and (D). Section from PZQ treated mouse liver 45 days after S. mansoni infection. No granuloma or collagen fibrils were evident. Slides were visualized on a Zeiss Axio Scope.A1 using a 20x objective and images were acquired with a Nikon D5200 Camera fitted with a MM-SLR Adapter. Scale bar = 130 μm. (TIFF) [file pntd.0005691.s006.tiff]

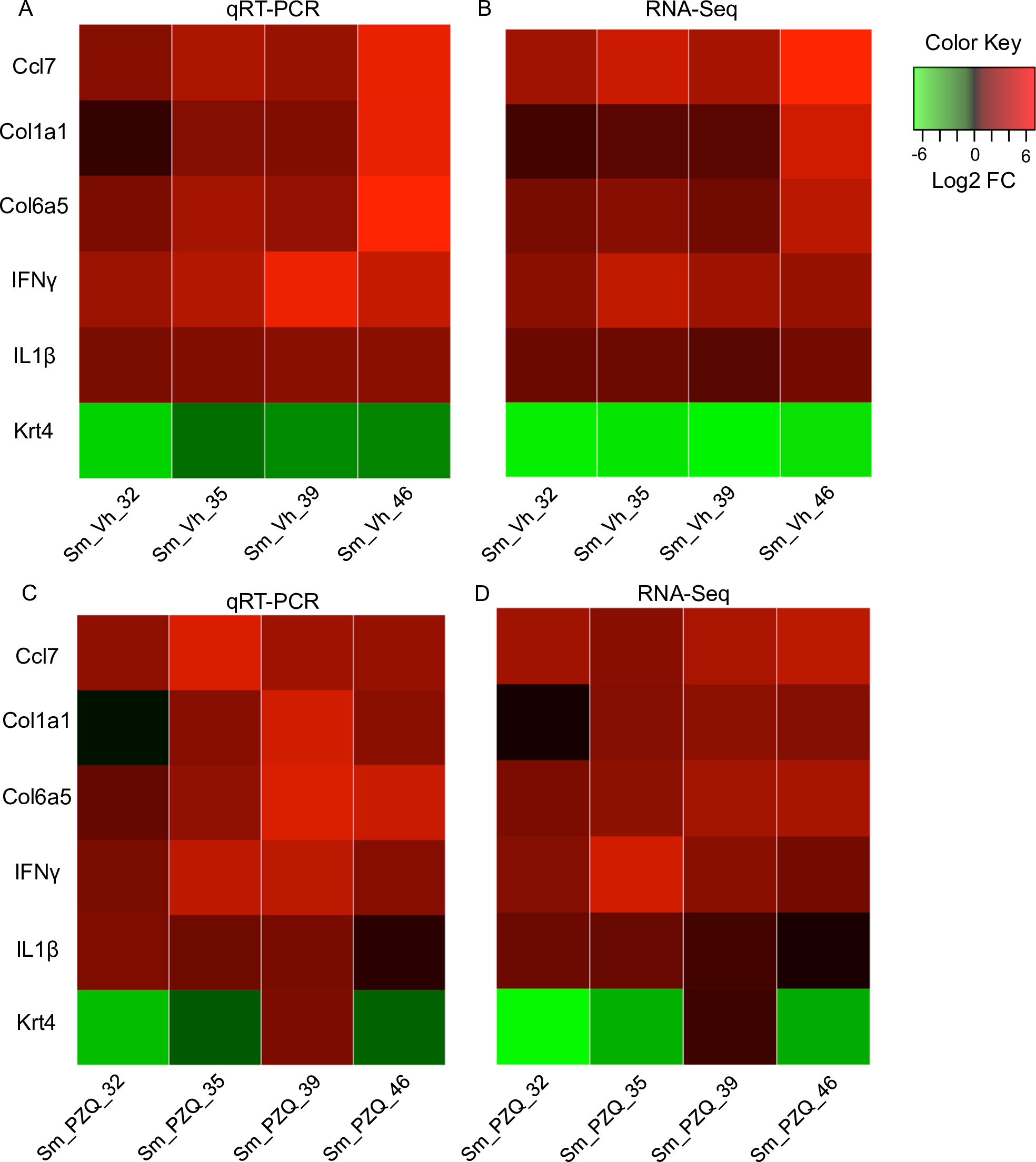

Supplement: S7 Fig — Log2 changes in expression of genes encoding chemokine Ccl7, collagen type I pro-α chain (col1a1), collagen type VI α5 chain (Col6a5), interferon γ (IFNγ) interleukin 1β (IL1β) and keratin 4 (Krt4) in Vh and PZQ treated infected mice at days 32, 35, 39 and 46 post infection. Gene expression was assessed by qRT-PCR (A, C)) and RNA-Seq (B, D) after Vh and PZQ treatment respectively. The gene expression profile at each point is the average of three biological replicates. For both RNA-Seq and qRT-PCR data, regions of red and green indicate gene expression has increased and decreased respectively. (TIFF) [file pntd.0005691.s007.tiff]

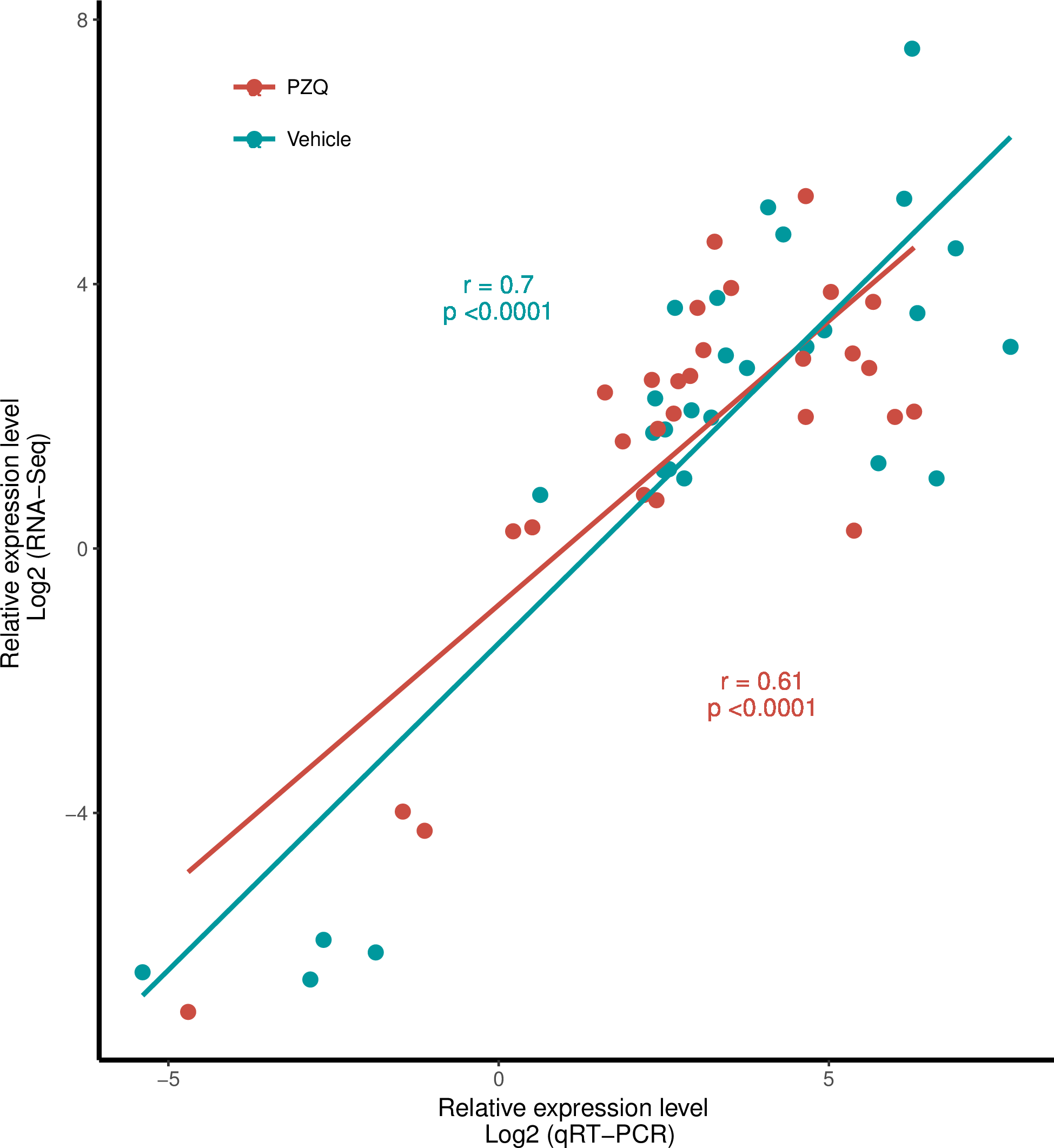

Supplement: S8 Fig — Relationship between RNA-Seq and qRT-PCR data for six genes over each time point and for both treatments was established using Spearman’s Rho correlation. (TIFF) [file pntd.0005691.s008.tiff]
