# Supplementary material for: Effect of praziquantel on the differential expression of mouse hepatic genes and parasite ATP binding cassette transporter gene family members during Schistosoma mansoni infection
Source: PLoS Negl Trop Dis. 2017 Jun 26;11(6):e0005691. doi: 10.1371/journal.pntd.0005691 (PMC5501684; doi:10.1371/journal.pntd.0005691)
Supplement: S1 Table — GAPDH (ENSMUSG00000057666) primer set is part of the PrimePCR Probe Assay (BIORAD qMmuCED0027497). (PDF) [file pntd.0005691.s009.pdf]

S1 Table. Primers used in *Mus musculus* quantitative real-time PCR reactions.

Ensemble Gene ID	Gene Symbol	Forward Primer	Reverse Primer
ENSMUSG00000035373	Ccl7	TTTGTTTCTTGACATAGCAGCAT	TCTCACTCTCTTTCTCCAACCA
ENSMUSG00000001506	Col1α1	GAAACCTCTCTCGCCTCTTG	TGGTGAAGCAGGCAAGC
ENSMUSG00000091345	Col6α5	TCCACGATTTCCACTTGATCC	GCCGTCCTAGCTCCAAAG
ENSMUSG00000055170	IFNγ	CTCTTCTCATGGCTGTTTCT	TTCTTCCACATCTATGCCACTT
ENSMUSG00000027398	IL1β	CCACCTCAATGGACAGAATATCA	CCCAAGGCCACAGGTATTT
ENSMUSG00000059668	Krt4	CTCTGCATCAGCCACAGAT3	GATAGCCTGAAGACCACCAAG
