# Supplementary material for: Effect of praziquantel on the differential expression of mouse hepatic genes and parasite ATP binding cassette transporter gene family members during Schistosoma mansoni infection
Source: PLoS Negl Trop Dis. 2017 Jun 26;11(6):e0005691. doi: 10.1371/journal.pntd.0005691 (PMC5501684; doi:10.1371/journal.pntd.0005691)
Supplement: S2 Table — (PDF) [file pntd.0005691.s010.pdf]

S2 Table. Real-time PCR cycle threshold (Cq) raw data for *S. mansoni* reference gene GAPDH.

Well	Fluor	Target	Content	Sample	Biological Set Name	Cq	Cq Technical Mean	Cq Technical Std. Dev	Cq Biological Mean	Cq Biological Std. Dev
A01	SYBR	GAPDH5	Unkn-01	25V1	Sm_Vh_25	24.45	24.43	0.02	23.95	0.45
A03	SYBR	GAPDH5	Unkn-01	25V1	Sm_Vh_25	24.42	24.43	0.02	23.95	0.45
A05	SYBR	GAPDH5	Unkn-02	25V2	Sm_Vh_25	24.01	24.00	0.01	23.95	0.45
A06	SYBR	GAPDH5	Unkn-02	25V2	Sm_Vh_25	23.99	24.00	0.01	23.95	0.45
A07	SYBR	GAPDH5	Unkn-03	25V3	Sm_Vh_25	23.42	23.42	0.00	23.95	0.45
A08	SYBR	GAPDH5	Unkn-03	25V3	Sm_Vh_25	23.43	23.42	0.00	23.95	0.45
A10	SYBR	GAPDH5	Unkn-04	28V1	Sm_Vh_28	23.53	23.55	0.03	23.36	0.19
A11	SYBR	GAPDH5	Unkn-04	28V1	Sm_Vh_28	23.57	23.55	0.03	23.36	0.19
B02	SYBR	GAPDH5	Unkn-05	28V2	Sm_Vh_28	23.11	23.13	0.02	23.36	0.19
B03	SYBR	GAPDH5	Unkn-05	28V2	Sm_Vh_28	23.14	23.13	0.02	23.36	0.19
B05	SYBR	GAPDH5	Unkn-06	28V3	Sm_Vh_28	23.40	23.40	0.00	23.36	0.19
B06	SYBR	GAPDH5	Unkn-06	28V3	Sm_Vh_28	23.39	23.40	0.00	23.36	0.19
B08	SYBR	GAPDH5	Unkn-07	32V1	Sm_Vh_32	24.86	24.84	0.02	23.70	0.90
B09	SYBR	GAPDH5	Unkn-07	32V1	Sm_Vh_32	24.83	24.84	0.02	23.70	0.90
B11	SYBR	GAPDH5	Unkn-08	32V2	Sm_Vh_32	22.96	22.98	0.04	23.70	0.90
B12	SYBR	GAPDH5	Unkn-08	32V2	Sm_Vh_32	23.01	22.98	0.04	23.70	0.90
C01	SYBR	GAPDH5	Unkn-09	32V3	Sm_Vh_32	23.29	23.26	0.04	23.70	0.90
C03	SYBR	GAPDH5	Unkn-09	32V3	Sm_Vh_32	23.24	23.26	0.04	23.70	0.90
C05	SYBR	GAPDH5	Unkn-10	35V1	Sm_Vh_35	22.50	22.48	0.03	23.36	0.74
C06	SYBR	GAPDH5	Unkn-10	35V1	Sm_Vh_35	22.46	22.48	0.03	23.36	0.74
C07	SYBR	GAPDH5	Unkn-11	35V2	Sm_Vh_35	24.13	24.12	0.01	23.36	0.74
C08	SYBR	GAPDH5	Unkn-11	35V2	Sm_Vh_35	24.11	24.12	0.01	23.36	0.74
C11	SYBR	GAPDH5	Unkn-12	35V3	Sm_Vh_35	23.47	23.47	0.01	23.36	0.74
C12	SYBR	GAPDH5	Unkn-12	35V3	Sm_Vh_35	23.46	23.47	0.01	23.36	0.74
D02	SYBR	GAPDH5	Unkn-13	25P1	Sm_PZQ_25	22.91	22.94	0.04	23.73	0.64
D03	SYBR	GAPDH5	Unkn-13	25P1	Sm_PZQ_25	22.97	22.94	0.04	23.73	0.64
D05	SYBR	GAPDH5	Unkn-14	25P2	Sm_PZQ_25	24.32	24.34	0.03	23.73	0.64
D06	SYBR	GAPDH5	Unkn-14	25P2	Sm_PZQ_25	24.36	24.34	0.03	23.73	0.64
D07	SYBR	GAPDH5	Unkn-15	25P3	Sm_PZQ_25	23.88	23.91	0.05	23.73	0.64
D09	SYBR	GAPDH5	Unkn-15	25P3	Sm_PZQ_25	23.94	23.91	0.05	23.73	0.64
D11	SYBR	GAPDH5	Unkn-16	28P1	Sm_PZQ_28	24.38	24.39	0.01	24.20	0.36
D12	SYBR	GAPDH5	Unkn-16	28P1	Sm_PZQ_28	24.39	24.39	0.01	24.20	0.36
E01	SYBR	GAPDH5	Unkn-17	28P2	Sm_PZQ_28	24.48	24.48	0.00	24.20	0.36
E03	SYBR	GAPDH5	Unkn-17	28P2	Sm_PZQ_28	24.49	24.48	0.00	24.20	0.36
E05	SYBR	GAPDH5	Unkn-18	28P3	Sm_PZQ_28	23.73	23.74	0.02	24.20	0.36
E06	SYBR	GAPDH5	Unkn-18	28P3	Sm_PZQ_28	23.75	23.74	0.02	24.20	0.36
E07	SYBR	GAPDH5	Unkn-19	32P1	Sm_PZQ_32	22.71	22.70	0.01	23.94	1.03
E09	SYBR	GAPDH5	Unkn-19	32P1	Sm_PZQ_32	22.70	22.70	0.01	23.94	1.03
E11	SYBR	GAPDH5	Unkn-20	32P2	Sm_PZQ_32	25.01	24.99	0.03	23.94	1.03
E12	SYBR	GAPDH5	Unkn-20	32P2	Sm_PZQ_32	24.97	24.99	0.03	23.94	1.03
F01	SYBR	GAPDH5	Unkn-21	32P3	Sm_PZQ_32	24.15	24.12	0.03	23.94	1.03
F03	SYBR	GAPDH5	Unkn-21	32P3	Sm_PZQ_32	24.10	24.12	0.03	23.94	1.03
F04	SYBR	GAPDH5	Unkn-22	35P1	Sm_PZQ_35	23.79	23.79	0.01	23.57	0.44
F06	SYBR	GAPDH5	Unkn-22	35P1	Sm_PZQ_35	23.78	23.79	0.01	23.57	0.44
F07	SYBR	GAPDH5	Unkn-23	35P2	Sm_PZQ_35	23.04	23.01	0.03	23.57	0.44
F08	SYBR	GAPDH5	Unkn-23	35P2	Sm_PZQ_35	22.99	23.01	0.03	23.57	0.44
F10	SYBR	GAPDH5	Unkn-24	35P3	Sm_PZQ_35	23.89	23.91	0.04	23.57	0.44
F12	SYBR	GAPDH5	Unkn-24	35P3	Sm_PZQ_35	23.94	23.91	0.04	23.57	0.44
