# Supplementary material for: Effect of praziquantel on the differential expression of mouse hepatic genes and parasite ATP binding cassette transporter gene family members during Schistosoma mansoni infection
Source: PLoS Negl Trop Dis. 2017 Jun 26;11(6):e0005691. doi: 10.1371/journal.pntd.0005691 (PMC5501684; doi:10.1371/journal.pntd.0005691)
Supplement: S3 Table — (PDF) [file pntd.0005691.s011.pdf]

S3 Table. Primers used in *S. mansoni* quantitative real-time PCR reactions.

Gene ID	Gene	Forward Primer	Reverse Primer
Smp_055780	ABCB1-1 (SMDR2)	CGGTGCTACTCAGGGTTATTT	TCACCAAGGAAGCAATGACTAA
Smp_170820	ABCB1-2	TTTGGGAGCTGCAATGTTTATC	CCTCCGAGTGTTGTTCCAATA
Smp_089200.1	ABCB1-3	GGTTCAGCTGCGAAATGTATTC	CACAACCAGAAGGTCCAACTA
Smp_063000	ABCB8 (SMDR1)	CTTATTCGGTGCCATTCTGG	TTCGTGAGTCACAACGCATC
Smp_171740	ABCC1-1 (SmMRP1)	GGTCGTACTGGTTCGGGTAA	TGAACGTAACGTGCCAGAG
Smp_129820.1	ABCC1-2 (SmMRP2)	TTATGGCGTGCAATAGAATCTG	AGATTTGCTCCACCTTCACC
Smp_147250	ABCC10	CCTCCCTGGACCTTATACAAAC	CGAAGTAAGGTGAGAGGAACAG
Smp_181150	ABCG1	GTTTAGTCCCTTACCTCGCATC	ATTCACCACAAGCCACTTCC
Smp_126450	ABCG2 (BCRP)	AGACCATTCCCACAACACTATC	CGTCCAGCCAACACATCTAA
Smp_186020.1	Heat Shock Protein 70	ACTTAATCGGACCATACACGG	GCAAACATTCCACGTCTCAAAC
Smp_047660.1	Ferritin 2 (soma)	TGTCAATGGAGAAGGCAGTG	TGTCAATGGAGAAGGCAGTG
Smp_059480.1	Thioredoxin Peroxidase-1	GCTGACCGTAAACAGGAGATT	TTCACCGGACACACTTCAC
Smp_158110.1	Thioredoxin Peroxidase-2	CTTGACGAACAGGAAGGTCAT	GAGAGAGAAGCAACAGGATCAG
Smp_004470.1	Thioredoxin Peroxidase-3	GGTGGTGGATGGCCAATTTA	GTTTCTGATAGTCCGCCAAGAG
Smp_026560	Calmodulin-1	CAGAATCCTACAGAGGCTGAAC	TCGCCCAAGTTAGTCATTACAT
Smp_141660.1	VGCC β Subunit	GGTTGTTTTCGTCGTGCGAA	TCGCTGGTGACGGAATAAAG
Smp_056970.1	SmGAPDH	GTCATTCCAGCACTAAACGG	CCTTCCCTAACCTACATGTCAG
