# Supplementary material for: Effect of praziquantel on the differential expression of mouse hepatic genes and parasite ATP binding cassette transporter gene family members during Schistosoma mansoni infection
Source: PLoS Negl Trop Dis. 2017 Jun 26;11(6):e0005691. doi: 10.1371/journal.pntd.0005691 (PMC5501684; doi:10.1371/journal.pntd.0005691)
Supplement: S4 Table — (PDF) [file pntd.0005691.s012.pdf]

**S4 Table. Summary of Illumina read counts for each sequenced sample.**

| Sample | Total Reads Quality-trimmed reads (Q > 20) | *Mus muscularis* Total mapped reads (%) | *Schistosoma mansoni* Total mapped reads (%) |
|---|---|---|---|
| Mouse liver Sm_Vh_32 replicate 1 | 25,620,124 | 89.65% | 0.14% |
| Mouse liver Sm_Vh_32 replicate 2 | 22,252,855 | 89.47% | 0.16% |
| Mouse liver Sm_Vh_32 replicate 3 | 25,081,458 | 89.56% | 0.10% |
| Mouse liver Sm_Vh_35 replicate 1 | 24,891,081 | 89.80% | 0.17% |
| Mouse liver Sm_Vh_35 replicate 2 | 24,081,593 | 88.61% | 0.11% |
| Mouse liver Sm_Vh_35 replicate 3 | 23,519,336 | 89.14% | 0.10% |
| Mouse liver Sm_Vh_39 replicate 1 | 25,877,298 | 89.86% | 0.09% |
| Mouse liver Sm_Vh_39 replicate 2 | 24,571,558 | 89.45% | 0.09% |
| Mouse liver Sm_Vh_39 replicate 3 | 25,883,964 | 89.51% | 0.04% |
| Mouse liver Sm_Vh_46 replicate 1 | 24,289,752 | 89.55% | 0.08% |
| Mouse liver Sm_Vh_46 replicate 2 | 22,702,480 | 89.04% | 0.09% |
| Mouse liver Sm_Vh_46 replicate 3 | 23,443,615 | 88.23% | 0.06% |
| Mouse liver Sm_PZQ_32 replicate 1 | 22,604,127 | 89.60% | 0.07% |

| Sample | Total Reads Quality-trimmed reads (Q > 20) | *Mus muscularis* Total mapped reads (%) | *Schistosoma mansoni* Total mapped reads (%) |
|---|---|---|---|
| Mouse liver Sm_PZQ_32 replicate 2 | 24,752,867 | 90.12% | 0.05% |
| Mouse liver Sm_PZQ_32 replicate 3 | 25,486,684 | 89.57% | 0.08% |
| Mouse liver Sm_PZQ_35 replicate 1 | 25,052,120 | 89.91% | 0.03% |
| Mouse liver Sm_PZQ_35 replicate 2 | 25,062,968 | 90.07% | 0.04% |
| Mouse liver Sm_PZQ_35 replicate 3 | 26,780,030 | 90.27% | 0.03% |
| Mouse liver Sm_PZQ_39 replicate 1 | 28,606,471 | 91.03% | 0.02% |
| Mouse liver Sm_PZQ_39 replicate 2 | 27,926,097 | 90.19% | 0.03% |
| Mouse liver Sm_PZQ_39 replicate 3 | 26,796,964 | 89.65% | 0.03% |
| Mouse liver Sm_PZQ_46 replicate 1 | 24,861,788 | 89.77% | 0.01% |
| Mouse liver Sm_PZQ_46 replicate 2 | 27,194,443 | 90.84% | 0.02% |
| Mouse liver Sm_PZQ_46 replicate 3 | 27,273,699 | 90.19% | 0.03% |
| Mouse liver ui_Vh_32 replicate 1 | 25,530,722 | 89.77% | 0.02% |
| Mouse liver ui_Vh_32 replicate 2 | 27,707,093 | 89.01% | 0.02% |

| Sample | Total Reads Quality-trimmed reads (Q > 20) | *Mus muscularis* Total mapped reads (%) | *Schistosoma mansoni* Total mapped reads (%) |
|---|---|---|---|
| Mouse liver ui_Vh_32 replicate 3 | 29,884,662 | 89.31% | 0.02% |
| Mouse liver ui_Vh_35 replicate 1 | 24,735,213 | 89.18% | 0.02% |
| Mouse liver ui_Vh_35 replicate 2 | 28,591,287 | 90.17% | 0.02% |
| Mouse liver ui_Vh_35 replicate 3 | 27,115,159 | 90.06% | 0.01% |
| Mouse liver ui_Vh_39 replicate 1 | 23,688,034 | 88.22% | 0.01% |
| Mouse liver ui_Vh_39 replicate 2 | 26,698,924 | 89.84% | 0.01% |
| Mouse liver ui_Vh_39 replicate 3 | 25,732,157 | 89.41% | 0.01% |
| Mouse liver ui_Vh_46 replicate 1 | 25,245,330 | 89.37% | 0.01% |
| Mouse liver ui_Vh_46 replicate 2 | 24,695,867 | 89.83% | 0.02% |
| Mouse liver ui_Vh_46 replicate 3 | 25,395,334 | 90.05% | 0.01% |
