# Supplementary material for: Effect of praziquantel on the differential expression of mouse hepatic genes and parasite ATP binding cassette transporter gene family members during Schistosoma mansoni infection
Source: PLoS Negl Trop Dis. 2017 Jun 26;11(6):e0005691. doi: 10.1371/journal.pntd.0005691 (PMC5501684; doi:10.1371/journal.pntd.0005691)
Supplement: S5 Table — (PDF) [file pntd.0005691.s013.pdf]

**S5 Table. Number of differentially regulated genes in Vh and PZQ treated samples.**

| Timepoint Treatment | Total genes upregulated | Total genes downregulated | Immune genes upregulated | Immune genes downregulated |
|---|---|---|---|---|
| Sm_Vh_32 | 248 | 47 | 45 | 0 |
| Sm_Vh_35 | 546 | 21 | 91 | 0 |
| Sm_Vh_39 | 1191 | 293 | 150 | 6 |
| Sm_Vh_46 | 2633 | 1687 | 205 | 36 |
| Sm_PZQ_32 | 147 | 80 | 21 | 0 |
| Sm_PZQ_35 | 927 | 149 | 100 | 3 |
| Sm_PZQ_39 | 1304 | 474 | 153 | 10 |
| Sm_PZQ_46 | 937 | 387 | 93 | 9 |
