# Supplementary material for: Effect of praziquantel on the differential expression of mouse hepatic genes and parasite ATP binding cassette transporter gene family members during Schistosoma mansoni infection
Source: PLoS Negl Trop Dis. 2017 Jun 26;11(6):e0005691. doi: 10.1371/journal.pntd.0005691 (PMC5501684; doi:10.1371/journal.pntd.0005691)
Supplement: S6 Table — (PDF) [file pntd.0005691.s014.pdf]

S6 Table. Identities of all differentially expressed hepatic genes at each time point and treatment.

Sm_PZQ_32

Ensembl gene id	log2FC	p-adjusted
ENSMUSG00000063779	8.01	4.1015E-10
ENSMUSG00000026582	5.37	3.01732E-11
ENSMUSG00000009185	3.83	0.000623302
ENSMUSG00000028415	3.73	0.016098003
ENSMUSG00000025002	3.65	0.000539228
ENSMUSG00000035373	3.64	0.003530533
ENSMUSG00000029188	3.45	1.09776E-08
ENSMUSG00000037887	3.40	0.040469546
ENSMUSG00000015134	3.35	0.005141729
ENSMUSG00000020676	3.27	0.000170218
ENSMUSG00000061100	3.17	0.009504309
ENSMUSG00000091971	3.15	0.020518289
ENSMUSG00000015312	3.11	0.009221328
ENSMUSG00000090877	2.99	0.000103962
ENSMUSG00000022651	2.74	0.023477463
ENSMUSG00000030483	2.70	0.003138884
ENSMUSG00000058427	2.69	0.030157928
ENSMUSG00000057465	2.68	0.018388107
ENSMUSG00000037411	2.65	1.58952E-21
ENSMUSG00000018924	2.61	0.023301535
ENSMUSG00000026628	2.61	0.001086847
ENSMUSG00000048191	2.59	7.61412E-05
ENSMUSG00000040809	2.57	0.025422597
ENSMUSG00000020431	2.53	1.76316E-06
ENSMUSG00000040026	2.49	5.17828E-05
ENSMUSG00000009350	2.42	0.005265203
ENSMUSG00000032484	2.41	0.016958879
ENSMUSG00000046006	2.40	0.009662061
ENSMUSG00000091345	2.36	0.000358803
ENSMUSG00000044309	2.36	0.041952439
ENSMUSG00000039013	2.34	0.005229605
ENSMUSG00000022346	2.33	0.02548505
ENSMUSG00000037071	2.32	0.000353528
ENSMUSG00000041653	2.20	4.2674E-16
ENSMUSG00000035385	2.20	0.000216042
ENSMUSG00000049580	2.16	0.003825518
ENSMUSG00000074115	2.15	0.011115807
ENSMUSG00000025804	2.15	0.014984485
ENSMUSG00000062991	2.13	0.01246962
ENSMUSG00000022456	2.12	0.010639796
ENSMUSG00000021453	2.10	0.021359983
ENSMUSG00000095170	2.10	0.013565959
ENSMUSG00000029352	2.06	0.008767956
ENSMUSG00000029417	2.02	4.7073E-05
ENSMUSG00000058672	1.99	1.58952E-21
ENSMUSG00000034855	1.94	6.57962E-06
ENSMUSG00000040152	1.92	0.00736127
ENSMUSG00000097768	1.88	0.000585969
ENSMUSG00000022797	1.84	0.004754394
ENSMUSG00000031289	1.83	0.007918422

Ensembl gene id	log2FC	p-adjusted
ENSMUSG00000027398	1.81	2.84955E-14
ENSMUSG00000026069	1.80	0.029572666
ENSMUSG00000092075	1.80	0.006783202
ENSMUSG00000029657	1.79	9.28727E-11
ENSMUSG00000032578	1.79	0.000656538
ENSMUSG00000023078	1.77	0.015996073
ENSMUSG00000026580	1.77	0.016368022
ENSMUSG00000026348	1.76	2.12563E-07
ENSMUSG00000023132	1.74	1.19046E-05
ENSMUSG00000021322	1.73	0.017675952
ENSMUSG00000055116	1.72	1.12778E-06
ENSMUSG00000030789	1.71	6.48824E-07
ENSMUSG00000026536	1.71	0.045655698
ENSMUSG00000094786	1.68	0.010375584
ENSMUSG00000048782	1.66	0.042793838
ENSMUSG00000061540	1.65	0.021167105
ENSMUSG00000060950	1.59	0.001137614
ENSMUSG00000027230	1.58	0.007269371
ENSMUSG00000026822	1.56	0.017463444
ENSMUSG00000041220	1.55	0.000240494
ENSMUSG00000022057	1.55	3.77414E-12
ENSMUSG00000023067	1.53	0.006724398
ENSMUSG00000004038	1.53	0.018760231
ENSMUSG00000073406	1.51	0.046707128
ENSMUSG00000021208	1.50	0.022905287
ENSMUSG00000032786	1.49	0.005068895
ENSMUSG00000003721	1.49	0.002844524
ENSMUSG00000030428	1.48	0.011030725
ENSMUSG00000026614	1.48	0.026740085
ENSMUSG00000021270	1.48	0.000309062
ENSMUSG00000041481	1.46	0.012619629
ENSMUSG00000081058	1.44	0.006840784
ENSMUSG00000052684	1.44	0.000159831
ENSMUSG00000028633	1.42	0.008360226
ENSMUSG00000049804	1.39	0.034059467
ENSMUSG00000078901	1.39	0.022630432
ENSMUSG00000053113	1.38	0.037258447
ENSMUSG00000022003	1.37	0.007052921
ENSMUSG00000038037	1.35	0.018658265
ENSMUSG00000097815	1.34	0.011337351
ENSMUSG00000052726	1.33	0.001319334
ENSMUSG00000035049	1.33	0.002709315
ENSMUSG00000078650	1.33	3.86427E-13
ENSMUSG00000096606	1.32	1.77144E-05
ENSMUSG00000029135	1.31	0.024126476
ENSMUSG00000001020	1.31	0.050074334
ENSMUSG00000028698	1.31	0.002810705
ENSMUSG00000027750	1.30	0.007523605
ENSMUSG00000018927	1.30	0.030242251
ENSMUSG00000024679	1.30	4.69346E-09
ENSMUSG00000061578	1.28	0.037393655
ENSMUSG00000023905	1.28	0.009334761

Ensembl gene id	log2FC	p-adjusted
ENSMUSG00000022951	1.27	1.88036E-08
ENSMUSG00000026548	1.27	0.007961287
ENSMUSG00000028459	1.27	0.025438471
ENSMUSG00000070419	1.25	0.012509891
ENSMUSG00000056529	1.24	0.039590332
ENSMUSG00000032735	1.23	0.016509362
ENSMUSG00000049723	1.23	0.017754552
ENSMUSG00000020656	1.22	0.013972734
ENSMUSG00000021338	1.22	0.000640425
ENSMUSG00000038233	1.21	0.032816639
ENSMUSG00000026890	1.21	0.001025386
ENSMUSG00000022534	1.21	0.015650802
ENSMUSG00000035686	1.21	0.000467534
ENSMUSG00000026390	1.20	0.008857815
ENSMUSG00000029630	1.18	0.003477393
ENSMUSG00000001774	1.17	1.64338E-13
ENSMUSG00000023988	1.17	2.16156E-07
ENSMUSG00000028967	1.14	0.000303386
ENSMUSG00000022584	1.13	0.008060654
ENSMUSG00000078866	1.13	0.006710572
ENSMUSG00000021365	1.12	0.044005102
ENSMUSG00000026020	1.12	0.00016661
ENSMUSG00000089726	1.11	0.016084986
ENSMUSG00000021958	1.11	0.002094963
ENSMUSG00000074743	1.11	0.007580256
ENSMUSG00000004814	1.09	0.019520467
ENSMUSG00000049037	1.09	0.000300463
ENSMUSG00000052776	1.08	0.024234066
ENSMUSG00000031506	1.08	0.044884425
ENSMUSG00000036752	1.07	2.38721E-10
ENSMUSG00000078899	1.07	0.013972734
ENSMUSG00000030847	1.05	7.20719E-10
ENSMUSG00000085156	1.05	0.05029595
ENSMUSG00000062515	1.04	0.026095259
ENSMUSG00000043091	1.04	0.017881215
ENSMUSG00000055730	1.04	0.000216839
ENSMUSG00000071637	1.03	0.00113042
ENSMUSG00000029817	1.03	6.41634E-09
ENSMUSG00000038508	1.02	0.000252602
ENSMUSG00000025190	1.01	8.96011E-12
ENSMUSG00000024270	1.01	0.002464301
ENSMUSG00000015656	1.01	5.9332E-11
ENSMUSG00000042354	1.01	3.23213E-09
ENSMUSG00000028680	1.00	6.8664E-09
ENSMUSG00000079293	1.00	4.80905E-05
ENSMUSG00000021228	-1.02	0.00061117
ENSMUSG00000024580	-1.04	1.43668E-10
ENSMUSG00000073835	-1.04	0.013524789
ENSMUSG00000031561	-1.04	3.3441E-10
ENSMUSG00000097426	-1.05	0.000915494
ENSMUSG00000044345	-1.07	4.85076E-11
ENSMUSG00000020623	-1.09	0.026292413

Ensembl gene id	log2FC	p-adjusted
ENSMUSG00000027376	-1.09	0.005688723
ENSMUSG00000043760	-1.09	0.042418245
ENSMUSG00000028527	-1.09	0.00578927
ENSMUSG00000037606	-1.09	0.041399874
ENSMUSG00000024665	-1.10	1.02331E-14
ENSMUSG00000026639	-1.10	0.011735855
ENSMUSG00000072949	-1.10	0.011735855
ENSMUSG00000095532	-1.10	0.01141204
ENSMUSG00000026259	-1.13	3.73055E-09
ENSMUSG00000091867	-1.15	0.01632668
ENSMUSG00000028712	-1.16	0.000447874
ENSMUSG00000037348	-1.18	4.31742E-11
ENSMUSG00000052302	-1.19	0.009567202
ENSMUSG00000091387	-1.20	0.000252072
ENSMUSG00000032010	-1.21	1.40774E-08
ENSMUSG00000027296	-1.21	0.00257522
ENSMUSG00000034774	-1.21	0.012387173
ENSMUSG00000085873	-1.21	0.011115807
ENSMUSG00000017688	-1.21	0.035983673
ENSMUSG00000087413	-1.21	0.002304503
ENSMUSG00000029797	-1.21	0.00434009
ENSMUSG00000097762	-1.23	3.3662E-06
ENSMUSG00000005677	-1.23	0.00033977
ENSMUSG00000021226	-1.24	0.028296786
ENSMUSG00000081207	-1.24	0.004897059
ENSMUSG00000017723	-1.24	0.007790339
ENSMUSG00000020131	-1.25	3.91758E-09
ENSMUSG00000053964	-1.26	6.54023E-07
ENSMUSG00000048445	-1.31	0.000149542
ENSMUSG00000026610	-1.32	0.018485227
ENSMUSG00000048489	-1.33	0.005535108
ENSMUSG00000089694	-1.34	0.019354287
ENSMUSG00000048058	-1.35	1.83049E-11
ENSMUSG00000042834	-1.38	1.96796E-09
ENSMUSG00000028789	-1.39	1.76316E-06
ENSMUSG00000078685	-1.42	0.013594934
ENSMUSG00000031618	-1.43	1.63293E-14
ENSMUSG00000008461	-1.44	0.042475403
ENSMUSG00000041078	-1.53	0.019491027
ENSMUSG00000030510	-1.55	0.021019207
ENSMUSG00000026315	-1.55	1.37311E-15
ENSMUSG00000061132	-1.59	0.014947144
ENSMUSG00000036027	-1.64	0.000203438
ENSMUSG00000040093	-1.67	1.55913E-28
ENSMUSG00000022762	-1.68	0.013575473
ENSMUSG00000097286	-1.72	0.044924595
ENSMUSG00000031765	-1.73	0.031879511
ENSMUSG00000074345	-1.73	0.008395386
ENSMUSG00000031762	-1.75	0.045752248
ENSMUSG00000094948	-1.78	0.001384998
ENSMUSG00000024411	-1.81	1.26857E-07
ENSMUSG00000087054	-1.83	0.029572666

Ensembl gene id	log2FC	p-adjusted
ENSMUSG00000066072	-1.86	1.67015E-05
ENSMUSG00000006462	-1.90	0.005037728
ENSMUSG00000020553	-1.91	0.013287427
ENSMUSG00000069441	-1.99	0.044005102
ENSMUSG00000052133	-2.01	0.049994588
ENSMUSG00000020722	-2.53	0.043079926
ENSMUSG00000006777	-2.58	0.003121684
ENSMUSG00000079465	-3.04	0.006539397
ENSMUSG00000016386	-3.31	0.001291842
ENSMUSG00000055775	-3.80	0.005638402
ENSMUSG00000043165	-3.93	0.001173585
ENSMUSG00000068614	-4.10	0.017430224
ENSMUSG00000022491	-4.36	0.005537328
ENSMUSG00000083079	-4.51	0.006631543
ENSMUSG00000096770	-5.36	0.005627376
ENSMUSG00000096493	-5.38	0.006840784
ENSMUSG00000031896	-5.73	0.006146201
ENSMUSG00000093931	-5.74	0.00535717
ENSMUSG00000096569	-5.80	0.005539912
ENSMUSG00000059668	-7.01	0.021843504
ENSMUSG00000042179	-7.57	0.044526508
**Sm_Vh_32**		
ENSMUSG00000044071	7.84	0.00903431
ENSMUSG00000063779	6.43	2.9466E-33
ENSMUSG00000076614	5.92	0.013974681
ENSMUSG00000087642	5.58	0.000653261
ENSMUSG00000094749	5.55	2.06718E-05
ENSMUSG00000076655	5.29	0.001484064
ENSMUSG00000032323	5.29	0.005557959
ENSMUSG00000026582	5.12	6.35795E-09
ENSMUSG00000095771	5.10	0.036425424
ENSMUSG00000009185	5.08	4.97972E-20
ENSMUSG00000095170	4.17	0.011633875
ENSMUSG00000093861	4.12	0.033771091
ENSMUSG00000022157	3.86	0.000692887
ENSMUSG00000072596	3.83	0.009536624
ENSMUSG00000028415	3.72	0.025382556
ENSMUSG00000035373	3.64	0.005253873
ENSMUSG00000079298	3.62	0.000409569
ENSMUSG00000040197	3.59	0.009551451
ENSMUSG00000022651	3.48	0.008517717
ENSMUSG00000067149	3.32	0.03673481
ENSMUSG00000020676	3.29	0.000209332
ENSMUSG00000016498	3.26	0.010288327
ENSMUSG00000094335	3.24	0.020122286
ENSMUSG00000057465	3.23	0.000258642
ENSMUSG00000071552	3.23	0.013893403
ENSMUSG00000035186	3.16	0.041065049
ENSMUSG00000029417	3.12	4.87507E-54
ENSMUSG00000094769	3.12	0.008627013
ENSMUSG00000040026	3.07	0.000209332
ENSMUSG00000015134	3.01	0.000215916

Ensembl gene id	log2FC	p-adjusted
ENSMUSG00000095704	3.00	0.01384189
ENSMUSG00000026011	2.99	0.016294603
ENSMUSG00000044309	2.88	5.24626E-06
ENSMUSG00000074115	2.78	0.000530381
ENSMUSG00000089727	2.74	0.039385176
ENSMUSG00000061100	2.68	0.002347361
ENSMUSG00000068129	2.59	0.042313081
ENSMUSG00000018924	2.57	0.000215916
ENSMUSG00000076609	2.57	0.036488383
ENSMUSG00000025804	2.56	0.000764714
ENSMUSG00000076934	2.53	0.028959045
ENSMUSG00000040809	2.48	0.000756974
ENSMUSG00000023132	2.48	0.011672645
ENSMUSG00000039013	2.46	0.014641403
ENSMUSG00000030789	2.41	0.007111232
ENSMUSG00000044162	2.39	0.029737251
ENSMUSG00000061780	2.38	0.000518503
ENSMUSG00000022456	2.38	0.001265832
ENSMUSG00000046006	2.37	0.008849614
ENSMUSG00000076564	2.34	0.00747182
ENSMUSG00000026822	2.32	2.50711E-08
ENSMUSG00000021322	2.28	0.011545784
ENSMUSG00000091345	2.27	4.97972E-20
ENSMUSG00000016283	2.25	0.007636859
ENSMUSG00000054203	2.24	0.005483014
ENSMUSG00000041481	2.24	0.016264068
ENSMUSG00000034855	2.23	1.04783E-08
ENSMUSG00000035448	2.21	0.002214517
ENSMUSG00000025473	2.20	9.23796E-07
ENSMUSG00000061540	2.17	0.000409569
ENSMUSG00000040152	2.13	0.00084348
ENSMUSG00000031289	2.12	0.000737901
ENSMUSG00000006403	2.07	0.015351536
ENSMUSG00000069763	2.06	0.030975355
ENSMUSG00000071005	2.04	0.005052917
ENSMUSG00000050578	2.04	0.004619323
ENSMUSG00000031880	2.02	0.007158392
ENSMUSG00000022126	2.01	0.027574172
ENSMUSG00000037868	2.00	0.000235875
ENSMUSG00000018930	1.99	0.031591307
ENSMUSG00000030124	1.99	0.015942959
ENSMUSG00000035385	1.98	8.23883E-05
ENSMUSG00000004814	1.98	0.008470307
ENSMUSG00000026069	1.96	0.013327989
ENSMUSG00000024353	1.94	0.010987865
ENSMUSG00000018927	1.94	0.000293949
ENSMUSG00000028341	1.93	0.044772681
ENSMUSG00000053318	1.92	3.65345E-06
ENSMUSG00000036067	1.91	0.00757470
ENSMUSG00000053977	1.90	4.54346E-05
ENSMUSG00000001020	1.90	0.000400439
ENSMUSG00000038037	1.87	0.008830261

Ensembl gene id	log2FC	p-adjusted
ENSMUSG00000064147	1.84	0.013327989
ENSMUSG00000029352	1.83	0.03673481
ENSMUSG00000030165	1.79	0.004929506
ENSMUSG00000040907	1.78	0.028213433
ENSMUSG00000027579	1.78	0.034358216
ENSMUSG00000025163	1.78	0.012009406
ENSMUSG00000026580	1.76	1.5255E-08
ENSMUSG00000062524	1.76	0.013954702
ENSMUSG00000022057	1.75	0.006493028
ENSMUSG00000035042	1.75	0.007149962
ENSMUSG00000027398	1.75	2.58071E-12
ENSMUSG00000003411	1.74	0.00887016
ENSMUSG00000079547	1.73	0.017897454
ENSMUSG00000017002	1.73	0.007443344
ENSMUSG00000073421	1.70	0.035631037
ENSMUSG00000037071	1.70	0.00773643
ENSMUSG00000049723	1.69	0.007408014
ENSMUSG00000087150	1.69	0.000621089
ENSMUSG00000015396	1.68	2.56539E-14
ENSMUSG00000022534	1.66	0.000215916
ENSMUSG00000037411	1.64	1.16921E-09
ENSMUSG00000026875	1.64	0.000692887
ENSMUSG00000049130	1.63	0.018534959
ENSMUSG00000036594	1.61	0.00066938
ENSMUSG00000023078	1.61	0.025382556
ENSMUSG00000049103	1.61	0.012867165
ENSMUSG00000021765	1.61	0.03120301
ENSMUSG00000026348	1.60	0.008646465
ENSMUSG00000015437	1.60	0.000235875
ENSMUSG00000000682	1.60	7.28054E-06
ENSMUSG00000001444	1.59	0.006360012
ENSMUSG00000028874	1.57	0.011633875
ENSMUSG00000026390	1.55	5.00032E-07
ENSMUSG00000085963	1.55	0.023587049
ENSMUSG00000025701	1.55	0.014844587
ENSMUSG00000055116	1.55	0.00887016
ENSMUSG00000032271	1.54	0.01079432
ENSMUSG00000017652	1.52	0.000354703
ENSMUSG00000076617	1.52	0.01295010
ENSMUSG00000024401	1.51	0.014871496
ENSMUSG00000045502	1.50	0.009292133
ENSMUSG00000027368	1.48	0.029616025
ENSMUSG00000024610	1.48	0.033056956
ENSMUSG00000042817	1.47	0.011692696
ENSMUSG00000028270	1.46	0.005563489
ENSMUSG00000079293	1.44	0.003372972
ENSMUSG00000026628	1.41	0.005461532
ENSMUSG00000056529	1.40	0.00027356
ENSMUSG00000027230	1.40	0.00389290
ENSMUSG00000004612	1.40	0.002781256
ENSMUSG00000063193	1.40	0.000512916
ENSMUSG00000062545	1.40	0.012867165

Ensembl gene id	log2FC	p-adjusted
ENSMUSG00000044811	1.39	0.007622556
ENSMUSG00000089672	1.39	0.011706409
ENSMUSG00000044827	1.38	0.000992974
ENSMUSG00000071714	1.36	0.000518503
ENSMUSG00000040751	1.36	0.012700567
ENSMUSG00000021356	1.35	0.040865813
ENSMUSG00000026271	1.35	0.00028370
ENSMUSG00000035929	1.34	0.006522559
ENSMUSG00000024679	1.34	0.000413327
ENSMUSG00000048534	1.34	0.047810269
ENSMUSG00000049588	1.33	0.023709671
ENSMUSG00000027962	1.32	1.3334E-06
ENSMUSG00000021423	1.32	0.00559377
ENSMUSG00000037649	1.32	0.013761498
ENSMUSG00000021728	1.31	0.025591081
ENSMUSG00000096606	1.31	0.008708187
ENSMUSG00000034266	1.31	0.009389701
ENSMUSG00000000318	1.31	0.043547738
ENSMUSG00000031506	1.31	0.014666675
ENSMUSG00000026070	1.30	0.032224569
ENSMUSG00000049037	1.30	0.000178216
ENSMUSG00000063605	1.30	0.024028615
ENSMUSG00000026358	1.29	0.011894815
ENSMUSG00000046031	1.28	0.025382556
ENSMUSG00000061578	1.28	0.046507847
ENSMUSG00000046223	1.28	0.006371934
ENSMUSG00000027611	1.27	0.000178021
ENSMUSG00000022876	1.27	0.007919107
ENSMUSG00000028359	1.26	9.23796E-07
ENSMUSG00000028480	1.26	0.001612644
ENSMUSG00000001119	1.24	7.28054E-06
ENSMUSG00000073409	1.24	0.005236218
ENSMUSG00000029298	1.23	0.001229284
ENSMUSG00000075602	1.23	0.001852507
ENSMUSG00000027408	1.22	0.055634895
ENSMUSG00000022584	1.22	0.005269024
ENSMUSG00000060044	1.22	0.001167745
ENSMUSG00000008845	1.22	0.00122764
ENSMUSG00000097815	1.21	0.006042103
ENSMUSG00000028459	1.20	0.000206968
ENSMUSG00000027750	1.20	1.53261E-08
ENSMUSG00000023274	1.20	0.026367231
ENSMUSG00000068227	1.20	0.011455796
ENSMUSG00000045763	1.19	0.012240463
ENSMUSG00000030742	1.18	0.005646271
ENSMUSG00000027347	1.18	0.009739814
ENSMUSG00000021453	1.17	0.019987362
ENSMUSG00000050335	1.16	0.018245578
ENSMUSG00000009687	1.15	0.007358206
ENSMUSG00000022372	1.15	1.18444E-05
ENSMUSG00000042759	1.15	0.000209332
ENSMUSG00000000409	1.15	0.045309822

Ensembl gene id	log2FC	p-adjusted
ENSMUSG00000032093	1.15	0.028676277
ENSMUSG00000020241	1.14	0.000643123
ENSMUSG00000057058	1.14	0.010082993
ENSMUSG00000030047	1.14	0.013327989
ENSMUSG00000003352	1.14	0.010404942
ENSMUSG00000040264	1.13	0.030351437
ENSMUSG00000026630	1.13	0.01202368
ENSMUSG00000015355	1.13	0.005783645
ENSMUSG00000024300	1.13	0.000259676
ENSMUSG00000058672	1.13	0.000499499
ENSMUSG00000056737	1.12	0.006937924
ENSMUSG00000020926	1.12	4.60852E-07
ENSMUSG00000066363	1.12	0.028105956
ENSMUSG00000002033	1.11	0.026367231
ENSMUSG00000027004	1.11	0.039792036
ENSMUSG00000059089	1.11	0.007588562
ENSMUSG00000021262	1.10	0.001484064
ENSMUSG00000062210	1.10	0.000470556
ENSMUSG00000061232	1.10	0.034265843
ENSMUSG00000021943	1.10	0.000342047
ENSMUSG00000023913	1.10	0.000512916
ENSMUSG00000079507	1.09	0.032139206
ENSMUSG00000026480	1.09	0.036932066
ENSMUSG00000060550	1.08	0.028070793
ENSMUSG00000073489	1.08	4.54346E-05
ENSMUSG00000049804	1.08	0.000151378
ENSMUSG00000078771	1.08	0.001924286
ENSMUSG00000041420	1.08	0.03227231
ENSMUSG00000030659	1.07	0.00852365
ENSMUSG00000018920	1.07	0.004230366
ENSMUSG00000001918	1.07	0.027574172
ENSMUSG00000062515	1.07	1.18444E-05
ENSMUSG00000039232	1.07	0.043949799
ENSMUSG00000068220	1.07	0.012072188
ENSMUSG00000036944	1.07	0.018245578
ENSMUSG00000022439	1.06	1.71703E-05
ENSMUSG00000026728	1.05	0.000582549
ENSMUSG00000020057	1.05	0.03626947
ENSMUSG00000079227	1.04	0.009368988
ENSMUSG00000037337	1.04	0.028018425
ENSMUSG00000038642	1.03	0.005962743
ENSMUSG00000024521	1.03	0.007454473
ENSMUSG00000020395	1.03	0.032429921
ENSMUSG00000042485	1.03	0.023587049
ENSMUSG00000051457	1.03	0.002350914
ENSMUSG00000022952	1.03	0.017719374
ENSMUSG00000039981	1.03	0.026367231
ENSMUSG00000072620	1.02	0.012868158
ENSMUSG00000020120	1.02	0.00014638
ENSMUSG00000021886	1.02	0.012444768
ENSMUSG00000029322	1.01	0.001484064
ENSMUSG00000026126	1.01	0.013953969

Ensembl gene id	log2FC	p-adjusted
ENSMUSG00000005087	1.01	0.003122023
ENSMUSG00000027995	1.01	0.010923566
ENSMUSG00000023349	1.01	0.000408481
ENSMUSG00000051439	1.01	0.003604618
ENSMUSG00000028698	1.01	0.037810055
ENSMUSG00000062593	1.01	0.022360543
ENSMUSG00000020407	0.99	0.016252074
ENSMUSG00000030148	0.97	0.032659871
ENSMUSG00000024397	0.96	0.012653138
ENSMUSG00000029413	0.95	0.000400439
ENSMUSG00000010025	-1.01	2.81641E-10
ENSMUSG00000034528	-1.01	0.001930714
ENSMUSG00000031016	-1.02	4.25946E-05
ENSMUSG00000017723	-1.02	0.038574416
ENSMUSG00000042834	-1.08	0.021454187
ENSMUSG00000028527	-1.10	0.000672537
ENSMUSG00000034785	-1.10	8.94044E-12
ENSMUSG00000095532	-1.11	0.016051469
ENSMUSG00000090622	-1.12	0.000154751
ENSMUSG00000031765	-1.13	0.026765139
ENSMUSG00000040584	-1.14	3.33914E-07
ENSMUSG00000027820	-1.15	0.006365481
ENSMUSG00000056666	-1.16	0.010551546
ENSMUSG00000072949	-1.22	0.011457887
ENSMUSG00000021226	-1.24	0.033056956
ENSMUSG00000056035	-1.28	0.011012253
ENSMUSG00000091867	-1.28	0.037792864
ENSMUSG00000081207	-1.31	0.005578949
ENSMUSG00000073835	-1.31	0.000352676
ENSMUSG00000097762	-1.33	6.56497E-07
ENSMUSG00000044566	-1.36	0.004792361
ENSMUSG00000028712	-1.42	0.003320649
ENSMUSG00000083478	-1.43	0.023723117
ENSMUSG00000029309	-1.45	0.021072427
ENSMUSG00000071551	-1.47	0.023319684
ENSMUSG00000026639	-1.51	0.032429921
ENSMUSG00000069170	-1.51	0.044310627
ENSMUSG00000022041	-1.72	3.82164E-06
ENSMUSG00000059741	-1.77	0.002767204
ENSMUSG00000024411	-1.96	4.97527E-08
ENSMUSG00000069441	-2.29	0.025921902
ENSMUSG00000094948	-2.46	1.18444E-05
ENSMUSG00000006777	-2.50	0.040559354
ENSMUSG00000052133	-2.62	0.004405658
ENSMUSG00000055775	-3.69	0.008830261
ENSMUSG00000096493	-3.95	0.020828869
ENSMUSG00000093931	-4.06	0.008365809
ENSMUSG00000074268	-4.17	0.008276518
ENSMUSG00000096569	-4.19	0.008418987
ENSMUSG00000096770	-4.52	0.007793056
ENSMUSG00000070332	-4.75	0.040621907
ENSMUSG00000058126	-4.78	0.007802449

Ensembl gene id	log2FC	p-adjusted
ENSMUSG00000041984	-5.47	0.036876929
ENSMUSG00000042179	-5.57	0.007475707
ENSMUSG00000031896	-5.58	0.007616277
ENSMUSG00000059668	-6.41	0.043516825
ENSMUSG00000044041	-7.74	0.003484547
**Sm_PZQ_35**		
ENSMUSG00000063779	10.19	0.000350582
ENSMUSG00000030111	8.41	0.04443039
ENSMUSG00000030162	7.07	1.35491E-08
ENSMUSG00000040809	6.77	0.046890337
ENSMUSG00000062345	6.51	0.003860742
ENSMUSG00000044162	5.93	6.26521E-07
ENSMUSG00000021697	5.84	5.4027E-05
ENSMUSG00000050578	5.82	9.2785E-31
ENSMUSG00000076655	5.65	0.027250059
ENSMUSG00000020676	5.48	0.000313714
ENSMUSG00000055170	5.33	0.000201461
ENSMUSG00000031933	5.33	0.000201461
ENSMUSG00000069733	5.32	0.044875756
ENSMUSG00000035373	5.16	0.000333343
ENSMUSG00000046006	5.12	2.30029E-11
ENSMUSG00000028175	5.11	0.001499256
ENSMUSG00000050370	5.10	1.09189E-08
ENSMUSG00000066364	5.09	3.65385E-17
ENSMUSG00000022651	5.03	1.2897E-09
ENSMUSG00000020826	4.99	6.46347E-11
ENSMUSG00000027379	4.94	1.6084E-06
ENSMUSG00000016498	4.92	2.91824E-11
ENSMUSG00000064147	4.92	3.70219E-08
ENSMUSG00000009185	4.89	3.29301E-05
ENSMUSG00000044201	4.78	6.71694E-06
ENSMUSG00000031162	4.75	0.010676767
ENSMUSG00000001131	4.74	0.021027219
ENSMUSG00000050359	4.60	0.014001855
ENSMUSG00000073529	4.54	0.017963476
ENSMUSG00000026822	4.52	0.018096837
ENSMUSG00000051378	4.45	3.15028E-10
ENSMUSG00000061100	4.43	0.029963095
ENSMUSG00000042265	4.41	0.003769276
ENSMUSG00000057465	4.40	0.049414386
ENSMUSG00000074183	4.26	5.27336E-41
ENSMUSG00000026077	4.23	8.46003E-07
ENSMUSG00000025473	4.22	6.62282E-11
ENSMUSG00000041431	4.18	7.89102E-05
ENSMUSG00000076614	4.17	0.001533862
ENSMUSG00000021758	4.16	0.045575386
ENSMUSG00000025804	4.12	9.80192E-05
ENSMUSG00000074179	4.08	2.87073E-28
ENSMUSG00000035186	4.02	3.70291E-06
ENSMUSG00000026582	4.01	1.72414E-11
ENSMUSG00000021953	3.97	0.009380964
ENSMUSG00000024029	3.96	0.005524896

Ensembl gene id	log2FC	p-adjusted
ENSMUSG00000000805	3.95	0.002280509
ENSMUSG00000090802	3.94	0.003261407
ENSMUSG00000094749	3.83	0.008524453
ENSMUSG00000006398	3.80	1.46824E-05
ENSMUSG00000074115	3.78	0.00744903
ENSMUSG00000022126	3.78	8.39485E-10
ENSMUSG00000032783	3.76	0.010799723
ENSMUSG00000057729	3.75	0.03974486
ENSMUSG00000051314	3.75	0.004335508
ENSMUSG00000024529	3.70	4.00495E-11
ENSMUSG00000000562	3.70	0.026960475
ENSMUSG00000072599	3.68	0.021311131
ENSMUSG00000026011	3.66	2.85523E-05
ENSMUSG00000022033	3.66	0.002754016
ENSMUSG00000041481	3.66	1.93374E-07
ENSMUSG00000022157	3.65	0.033179225
ENSMUSG00000037725	3.64	3.7801E-12
ENSMUSG00000004814	3.61	1.51228E-05
ENSMUSG00000027469	3.61	7.54053E-05
ENSMUSG00000026955	3.59	0.043602391
ENSMUSG00000082976	3.58	0.000583868
ENSMUSG00000072596	3.56	0.000110227
ENSMUSG00000000182	3.55	0.000335887
ENSMUSG00000029816	3.54	0.000549649
ENSMUSG00000040152	3.52	0.00023278
ENSMUSG00000044309	3.51	0.010575678
ENSMUSG00000027073	3.50	0.002627066
ENSMUSG00000032487	3.49	0.010192664
ENSMUSG00000027654	3.46	4.71629E-07
ENSMUSG00000032218	3.43	1.33645E-06
ENSMUSG00000022021	3.40	6.08525E-05
ENSMUSG00000030268	3.39	0.005692926
ENSMUSG00000030867	3.39	0.000395325
ENSMUSG00000049723	3.37	1.2897E-09
ENSMUSG00000068606	3.37	0.001186086
ENSMUSG00000006403	3.36	6.30984E-08
ENSMUSG00000024791	3.33	5.59448E-06
ENSMUSG00000019942	3.33	0.000129303
ENSMUSG00000035385	3.32	7.12282E-08
ENSMUSG00000023940	3.29	3.28164E-08
ENSMUSG00000048327	3.28	0.001121311
ENSMUSG00000051839	3.26	0.003562589
ENSMUSG00000028068	3.25	2.96911E-07
ENSMUSG00000020897	3.23	0.001055789
ENSMUSG00000058755	3.23	0.004203968
ENSMUSG00000095704	3.23	0.006135022
ENSMUSG00000062148	3.22	0.002177727
ENSMUSG00000026683	3.21	3.08896E-05
ENSMUSG00000028965	3.19	0.000156672
ENSMUSG00000044313	3.17	0.01001139
ENSMUSG00000050014	3.16	0.011645193
ENSMUSG00000037411	3.15	2.27757E-49

Ensembl gene id	log2FC	p-adjusted
ENSMUSG00000022034	3.14	1.01998E-05
ENSMUSG00000068744	3.14	0.007959647
ENSMUSG00000034883	3.13	0.013899981
ENSMUSG00000048922	3.13	0.022291558
ENSMUSG00000023908	3.13	3.07713E-07
ENSMUSG00000017499	3.13	0.003428832
ENSMUSG00000040197	3.12	0.003500449
ENSMUSG00000047222	3.12	0.003825999
ENSMUSG00000064246	3.12	7.40123E-07
ENSMUSG00000020279	3.11	0.000903088
ENSMUSG00000015134	3.11	0.000135427
ENSMUSG00000019992	3.08	0.047039315
ENSMUSG00000023067	3.07	2.54013E-08
ENSMUSG00000045328	3.07	0.000384562
ENSMUSG00000027715	3.05	4.56137E-08
ENSMUSG00000053101	3.05	0.000514754
ENSMUSG00000026605	3.04	7.1139E-08
ENSMUSG00000061540	3.04	0.012827031
ENSMUSG00000028587	3.03	0.00455296
ENSMUSG00000037868	3.03	1.24468E-08
ENSMUSG00000091345	3.00	2.63332E-15
ENSMUSG00000015880	2.97	8.8449E-06
ENSMUSG00000037628	2.96	0.000231755
ENSMUSG00000038379	2.95	7.02438E-08
ENSMUSG00000020383	2.94	0.011748349
ENSMUSG00000038943	2.94	0.012056247
ENSMUSG00000046341	2.92	0.011361348
ENSMUSG00000020914	2.92	9.8857E-06
ENSMUSG00000079553	2.92	0.027583317
ENSMUSG00000003484	2.91	5.00992E-07
ENSMUSG00000063388	2.90	0.002455253
ENSMUSG00000028678	2.90	5.0655E-08
ENSMUSG00000091694	2.88	1.31737E-06
ENSMUSG00000062380	2.87	0.01095519
ENSMUSG00000087642	2.87	0.048386806
ENSMUSG00000098318	2.85	0.026785585
ENSMUSG00000033952	2.83	3.55559E-15
ENSMUSG00000046295	2.83	6.80165E-05
ENSMUSG00000026039	2.82	0.001702637
ENSMUSG00000040084	2.82	0.001210991
ENSMUSG00000029352	2.82	0.027811475
ENSMUSG00000024056	2.81	0.002433204
ENSMUSG00000003779	2.81	7.07425E-07
ENSMUSG00000030677	2.80	9.67652E-05
ENSMUSG00000024989	2.80	0.000681521
ENSMUSG00000057933	2.79	1.593E-27
ENSMUSG00000026622	2.78	4.98238E-09
ENSMUSG00000020808	2.77	0.000168967
ENSMUSG00000025161	2.77	1.70442E-09
ENSMUSG00000018924	2.76	9.4592E-06
ENSMUSG00000035683	2.76	0.000494353
ENSMUSG00000029414	2.73	0.00078801

Ensembl gene id	log2FC	p-adjusted
ENSMUSG00000023903	2.73	0.009408151
ENSMUSG00000034634	2.72	1.79542E-09
ENSMUSG00000039013	2.71	2.79595E-05
ENSMUSG00000055116	2.71	2.69129E-07
ENSMUSG00000030789	2.71	2.19286E-22
ENSMUSG00000020330	2.71	4.86657E-05
ENSMUSG00000021403	2.71	0.039936263
ENSMUSG00000028718	2.68	0.012582938
ENSMUSG00000017716	2.67	0.007619178
ENSMUSG00000042489	2.67	0.007025866
ENSMUSG00000089929	2.67	0.000612674
ENSMUSG00000002055	2.67	0.001903893
ENSMUSG00000025701	2.66	0.000608408
ENSMUSG00000026628	2.65	2.52817E-12
ENSMUSG00000037887	2.65	7.17614E-05
ENSMUSG00000031289	2.64	0.000371894
ENSMUSG00000028715	2.63	0.007174508
ENSMUSG00000035165	2.61	0.001070475
ENSMUSG00000034855	2.60	2.71749E-12
ENSMUSG00000021485	2.59	0.00011139
ENSMUSG00000051517	2.58	0.010278461
ENSMUSG00000022322	2.58	0.017406466
ENSMUSG00000027326	2.58	0.00014049
ENSMUSG00000040950	2.56	0.007998448
ENSMUSG00000023992	2.56	8.55866E-09
ENSMUSG00000041498	2.55	3.14441E-05
ENSMUSG00000039109	2.54	0.031483162
ENSMUSG00000030142	2.54	0.006770157
ENSMUSG00000001506	2.53	2.28471E-08
ENSMUSG00000058290	2.53	0.012900103
ENSMUSG00000001228	2.52	0.001322041
ENSMUSG00000053318	2.52	0.001055789
ENSMUSG00000036768	2.52	0.007321001
ENSMUSG00000022534	2.51	2.78408E-07
ENSMUSG00000073386	2.50	0.043033584
ENSMUSG00000026069	2.50	8.30443E-06
ENSMUSG00000001517	2.49	0.000175064
ENSMUSG00000030144	2.49	0.043025808
ENSMUSG00000005470	2.48	0.012592489
ENSMUSG00000027331	2.48	3.50714E-07
ENSMUSG00000036777	2.47	0.000224836
ENSMUSG00000045502	2.47	0.000773477
ENSMUSG00000028480	2.47	1.19839E-12
ENSMUSG00000023015	2.46	1.87805E-06
ENSMUSG00000037466	2.46	0.001532858
ENSMUSG00000038037	2.44	5.50095E-06
ENSMUSG00000028832	2.43	3.53934E-07
ENSMUSG00000018930	2.43	0.000722882
ENSMUSG00000046591	2.43	0.000904033
ENSMUSG00000034570	2.41	0.034058716
ENSMUSG00000060044	2.40	1.24645E-09
ENSMUSG00000086564	2.38	0.025585276

Ensembl gene id	log2FC	p-adjusted
ENSMUSG00000039774	2.37	0.016310086
ENSMUSG00000001403	2.37	0.00218961
ENSMUSG00000041064	2.36	0.002113089
ENSMUSG00000034023	2.36	0.000683413
ENSMUSG00000019214	2.36	0.000337874
ENSMUSG00000031004	2.35	7.17538E-06
ENSMUSG00000006574	2.35	3.28164E-08
ENSMUSG00000035448	2.34	0.000121493
ENSMUSG00000052485	2.34	0.007156853
ENSMUSG00000071714	2.32	0.000813287
ENSMUSG00000026764	2.32	0.039964676
ENSMUSG00000040907	2.32	0.004878685
ENSMUSG00000023505	2.32	4.80307E-06
ENSMUSG00000021614	2.32	0.001379056
ENSMUSG00000040473	2.31	0.005434261
ENSMUSG00000032098	2.30	0.009408151
ENSMUSG00000038264	2.28	0.000123675
ENSMUSG00000028364	2.28	0.002046542
ENSMUSG00000020493	2.28	1.67804E-07
ENSMUSG00000031779	2.27	0.000341508
ENSMUSG00000034413	2.27	4.40835E-10
ENSMUSG00000005800	2.27	0.010174932
ENSMUSG00000035365	2.26	0.00123441
ENSMUSG00000034206	2.26	0.048292959
ENSMUSG00000037544	2.26	2.06382E-07
ENSMUSG00000050335	2.25	6.87558E-10
ENSMUSG00000034394	2.24	0.049664081
ENSMUSG00000021596	2.24	0.00202559
ENSMUSG00000026390	2.22	1.03842E-16
ENSMUSG00000027656	2.22	0.040447138
ENSMUSG00000056054	2.21	0.019808474
ENSMUSG00000032254	2.21	0.021496085
ENSMUSG00000028270	2.21	3.18786E-05
ENSMUSG00000004709	2.21	0.007180247
ENSMUSG00000036223	2.19	0.026660675
ENSMUSG00000029417	2.18	2.79486E-38
ENSMUSG00000047497	2.18	6.06685E-05
ENSMUSG00000029811	2.16	0.005857134
ENSMUSG00000028873	2.15	2.18056E-06
ENSMUSG00000034612	2.15	1.3784E-05
ENSMUSG00000021728	2.14	0.003531078
ENSMUSG00000012443	2.14	0.00057629
ENSMUSG00000005667	2.14	0.004056422
ENSMUSG00000040026	2.13	0.004119937
ENSMUSG00000039187	2.12	0.00853606
ENSMUSG00000049130	2.12	0.014276265
ENSMUSG00000022422	2.12	0.037153477
ENSMUSG00000052726	2.12	0.005101455
ENSMUSG00000028702	2.12	0.037856712
ENSMUSG00000047878	2.12	0.005559839
ENSMUSG00000018927	2.10	1.50776E-05
ENSMUSG00000034311	2.09	0.00045518

Ensembl gene id	log2FC	p-adjusted
ENSMUSG00000029075	2.08	0.006152627
ENSMUSG00000042817	2.08	0.00045518
ENSMUSG00000031662	2.08	3.87439E-06
ENSMUSG00000026123	2.07	1.14121E-06
ENSMUSG00000041202	2.07	0.001992885
ENSMUSG00000000869	2.07	0.013914892
ENSMUSG00000024670	2.05	5.30187E-05
ENSMUSG00000027750	2.05	1.73119E-08
ENSMUSG00000026630	2.05	0.002350284
ENSMUSG00000001020	2.04	0.000198684
ENSMUSG00000020431	2.04	0.002786171
ENSMUSG00000062939	2.04	0.000494353
ENSMUSG00000032496	2.04	0.015831221
ENSMUSG00000030413	2.03	0.007269922
ENSMUSG00000034591	2.03	0.008724266
ENSMUSG00000046223	2.02	0.000420133
ENSMUSG00000022504	2.02	0.000816418
ENSMUSG00000025912	2.02	0.000644711
ENSMUSG00000031494	2.02	0.006711671
ENSMUSG00000026196	2.01	0.002595401
ENSMUSG00000049103	2.01	2.07979E-05
ENSMUSG00000027230	2.01	0.008904983
ENSMUSG00000000318	2.01	1.42167E-06
ENSMUSG00000034906	2.01	0.000341876
ENSMUSG00000056071	2.01	0.016298786
ENSMUSG00000027306	2.00	6.59157E-05
ENSMUSG00000013766	2.00	0.035091106
ENSMUSG00000079293	2.00	6.95722E-08
ENSMUSG00000030641	1.99	0.000660432
ENSMUSG00000027323	1.99	2.79174E-05
ENSMUSG00000031262	1.99	0.01459005
ENSMUSG00000036067	1.99	0.000928326
ENSMUSG00000003051	1.98	0.00018603
ENSMUSG00000027797	1.98	0.001046772
ENSMUSG00000022440	1.97	5.65468E-11
ENSMUSG00000040412	1.96	4.42718E-09
ENSMUSG00000037313	1.95	0.004175645
ENSMUSG00000005410	1.95	0.002144371
ENSMUSG00000024660	1.95	3.19342E-05
ENSMUSG00000061577	1.94	0.002042299
ENSMUSG00000069910	1.94	0.016594823
ENSMUSG00000079547	1.94	0.006719985
ENSMUSG00000032815	1.94	0.011682052
ENSMUSG00000046058	1.94	0.036455152
ENSMUSG00000058794	1.94	0.021942984
ENSMUSG00000026271	1.93	1.24645E-09
ENSMUSG00000038508	1.93	0.001177535
ENSMUSG00000005824	1.92	0.003174258
ENSMUSG00000026580	1.90	0.001286728
ENSMUSG00000020649	1.89	0.007327726
ENSMUSG00000037649	1.89	0.000249021
ENSMUSG00000029752	1.87	0.019051515

Ensembl gene id	log2FC	p-adjusted
ENSMUSG00000030786	1.87	0.016395341
ENSMUSG00000073705	1.87	0.007321001
ENSMUSG00000023078	1.85	0.000860556
ENSMUSG00000037820	1.85	3.53934E-07
ENSMUSG00000036594	1.85	0.012426046
ENSMUSG00000069324	1.85	0.008982568
ENSMUSG00000022057	1.85	1.55308E-06
ENSMUSG00000056737	1.84	1.12659E-11
ENSMUSG00000024672	1.84	0.001245987
ENSMUSG00000041449	1.84	1.07369E-15
ENSMUSG00000092517	1.83	0.005733975
ENSMUSG00000078606	1.83	0.012586515
ENSMUSG00000066363	1.83	0.000117113
ENSMUSG00000030346	1.82	0.011003239
ENSMUSG00000003541	1.82	8.07945E-05
ENSMUSG00000074676	1.82	0.008240154
ENSMUSG00000000682	1.82	3.65385E-17
ENSMUSG00000030589	1.81	0.000521374
ENSMUSG00000023132	1.81	0.020148315
ENSMUSG00000047534	1.81	0.047239852
ENSMUSG00000084796	1.81	0.005584898
ENSMUSG00000004098	1.81	1.29936E-08
ENSMUSG00000029283	1.81	0.018992005
ENSMUSG00000023349	1.81	7.8297E-13
ENSMUSG00000001119	1.81	1.49945E-11
ENSMUSG00000020241	1.81	3.98775E-15
ENSMUSG00000025002	1.80	0.041020308
ENSMUSG00000095788	1.80	0.007257784
ENSMUSG00000027347	1.79	3.67516E-05
ENSMUSG00000031506	1.79	0.001284713
ENSMUSG00000042254	1.79	3.55741E-05
ENSMUSG00000068129	1.79	0.011973501
ENSMUSG00000070000	1.79	0.001577834
ENSMUSG00000073421	1.78	0.014396713
ENSMUSG00000047139	1.78	3.23149E-05
ENSMUSG00000021298	1.78	0.002125332
ENSMUSG00000029372	1.78	0.040129303
ENSMUSG00000040264	1.78	1.94339E-05
ENSMUSG00000035232	1.77	0.000116443
ENSMUSG00000000204	1.76	0.009953627
ENSMUSG00000079363	1.76	0.000587713
ENSMUSG00000056498	1.76	0.000202477
ENSMUSG00000040441	1.76	0.000392777
ENSMUSG00000039153	1.76	0.004013883
ENSMUSG00000026581	1.76	1.20745E-10
ENSMUSG00000021765	1.75	0.001924962
ENSMUSG00000052087	1.74	0.001284846
ENSMUSG00000031821	1.74	0.003561562
ENSMUSG00000063193	1.74	3.6504E-13
ENSMUSG00000021822	1.74	0.000106364
ENSMUSG00000053113	1.74	1.25561E-05
ENSMUSG00000002204	1.73	1.48095E-05

Ensembl gene id	log2FC	p-adjusted
ENSMUSG00000056529	1.73	7.71354E-05
ENSMUSG00000028931	1.73	6.30984E-08
ENSMUSG00000037474	1.73	0.004933044
ENSMUSG00000026875	1.73	0.000185501
ENSMUSG00000030124	1.73	0.000685472
ENSMUSG00000017737	1.73	0.000517526
ENSMUSG00000021280	1.73	0.002123992
ENSMUSG00000032484	1.72	0.018177175
ENSMUSG00000024679	1.72	0.000192192
ENSMUSG00000031880	1.72	0.0080852
ENSMUSG00000022456	1.72	0.024849072
ENSMUSG00000040528	1.72	0.000149622
ENSMUSG00000032322	1.71	0.000150226
ENSMUSG00000002257	1.71	0.001032095
ENSMUSG00000020057	1.71	4.53715E-05
ENSMUSG00000029661	1.71	2.11836E-10
ENSMUSG00000024610	1.70	0.006052765
ENSMUSG00000024675	1.70	0.003924608
ENSMUSG00000034317	1.70	2.94902E-05
ENSMUSG00000040751	1.70	0.00037528
ENSMUSG00000026043	1.70	9.91161E-13
ENSMUSG00000049307	1.70	0.012946446
ENSMUSG00000032053	1.70	1.6084E-06
ENSMUSG00000021965	1.69	0.004629276
ENSMUSG00000059089	1.69	0.003880852
ENSMUSG00000089672	1.69	5.01109E-05
ENSMUSG00000000028	1.69	6.27831E-05
ENSMUSG00000006517	1.69	0.007655257
ENSMUSG00000026532	1.68	0.00580453
ENSMUSG00000078853	1.68	0.007025866
ENSMUSG00000078763	1.68	5.3009E-05
ENSMUSG00000022876	1.68	2.56679E-06
ENSMUSG00000024401	1.68	0.048653529
ENSMUSG00000016496	1.68	9.51045E-05
ENSMUSG00000071713	1.67	0.000196044
ENSMUSG00000022385	1.67	0.000115576
ENSMUSG00000029373	1.67	0.035654163
ENSMUSG00000075611	1.67	0.042232757
ENSMUSG00000021196	1.67	3.42224E-05
ENSMUSG00000037548	1.67	0.000609645
ENSMUSG00000040663	1.67	0.016191839
ENSMUSG00000033826	1.67	0.001581551
ENSMUSG00000033031	1.66	2.74022E-07
ENSMUSG00000068220	1.66	4.07888E-05
ENSMUSG00000034792	1.66	0.005807492
ENSMUSG00000058672	1.66	1.17981E-05
ENSMUSG00000024669	1.65	0.044686571
ENSMUSG00000027408	1.65	8.14455E-11
ENSMUSG00000051225	1.64	0.002245091
ENSMUSG00000034266	1.64	0.007969317
ENSMUSG00000029552	1.64	1.17487E-05
ENSMUSG00000031934	1.63	0.001389047

Ensembl gene id	log2FC	p-adjusted
ENSMUSG00000079014	1.63	5.10414E-06
ENSMUSG00000050357	1.63	0.008669937
ENSMUSG00000027398	1.62	5.81502E-08
ENSMUSG00000026429	1.62	0.022287165
ENSMUSG00000030468	1.61	0.001379056
ENSMUSG00000035455	1.61	0.00145996
ENSMUSG00000098090	1.61	0.048219839
ENSMUSG00000026832	1.61	1.76826E-08
ENSMUSG00000034438	1.61	0.002870488
ENSMUSG00000054342	1.61	0.007532751
ENSMUSG00000032400	1.61	0.007032005
ENSMUSG00000002835	1.61	0.00021659
ENSMUSG00000050107	1.61	0.010773536
ENSMUSG00000062545	1.60	9.77298E-09
ENSMUSG00000045763	1.60	9.10282E-06
ENSMUSG00000004933	1.60	0.000757829
ENSMUSG00000037337	1.60	5.49763E-05
ENSMUSG00000087107	1.60	2.91323E-09
ENSMUSG00000004038	1.59	0.00416743
ENSMUSG00000079442	1.59	8.5452E-09
ENSMUSG00000041219	1.59	8.55866E-09
ENSMUSG00000050921	1.59	0.001991812
ENSMUSG00000000486	1.59	3.10786E-07
ENSMUSG00000017652	1.59	0.000198684
ENSMUSG00000024349	1.59	1.53329E-09
ENSMUSG00000029380	1.59	0.020951617
ENSMUSG00000085963	1.59	0.04737498
ENSMUSG00000061578	1.58	0.02117915
ENSMUSG00000024795	1.58	0.006296586
ENSMUSG00000000791	1.58	0.035728898
ENSMUSG00000022221	1.58	0.000341508
ENSMUSG00000022372	1.58	6.05515E-14
ENSMUSG00000082292	1.58	0.011212128
ENSMUSG00000020120	1.58	1.09571E-05
ENSMUSG00000000409	1.58	1.28227E-07
ENSMUSG00000005087	1.58	0.001530511
ENSMUSG00000062510	1.57	0.000278816
ENSMUSG00000029910	1.57	0.000509714
ENSMUSG00000027635	1.57	0.004386456
ENSMUSG00000043740	1.57	0.000422404
ENSMUSG00000044827	1.56	2.03352E-06
ENSMUSG00000022439	1.56	1.69542E-10
ENSMUSG00000042638	1.55	0.010631403
ENSMUSG00000032294	1.55	8.22015E-07
ENSMUSG00000024301	1.55	0.041734288
ENSMUSG00000021795	1.55	0.009072471
ENSMUSG00000043832	1.55	2.79155E-07
ENSMUSG00000054555	1.55	0.000172674
ENSMUSG00000000861	1.55	0.01417007
ENSMUSG00000039899	1.55	0.000158316
ENSMUSG00000025351	1.55	3.86093E-08
ENSMUSG00000006219	1.55	3.28164E-08

Ensembl gene id	log2FC	p-adjusted
ENSMUSG00000043157	1.55	0.007289781
ENSMUSG00000071068	1.54	0.006587325
ENSMUSG00000021423	1.54	1.12659E-11
ENSMUSG00000024737	1.54	4.06259E-07
ENSMUSG00000039699	1.54	3.71672E-05
ENSMUSG00000030707	1.53	2.84278E-16
ENSMUSG00000026728	1.53	8.55866E-09
ENSMUSG00000072620	1.53	6.40196E-06
ENSMUSG00000052684	1.53	3.87047E-05
ENSMUSG00000045826	1.53	9.21141E-05
ENSMUSG00000070691	1.53	0.007289781
ENSMUSG00000022584	1.53	0.029119079
ENSMUSG00000062524	1.53	0.005819679
ENSMUSG00000047330	1.53	0.045367811
ENSMUSG00000015314	1.53	0.000554907
ENSMUSG00000029204	1.53	0.000350359
ENSMUSG00000024640	1.53	0.029669634
ENSMUSG00000011008	1.53	0.007233487
ENSMUSG00000046031	1.52	0.00060612
ENSMUSG00000024334	1.52	0.03990065
ENSMUSG00000027962	1.52	5.13364E-05
ENSMUSG00000047517	1.52	0.045800324
ENSMUSG00000038156	1.52	6.86316E-05
ENSMUSG00000051212	1.51	0.005789553
ENSMUSG00000030793	1.51	2.36159E-05
ENSMUSG00000015437	1.51	0.025889669
ENSMUSG00000021262	1.51	3.2854E-07
ENSMUSG00000024673	1.51	2.25765E-06
ENSMUSG00000025877	1.50	5.11463E-06
ENSMUSG00000023274	1.50	0.011314639
ENSMUSG00000021613	1.50	0.043001679
ENSMUSG00000025648	1.50	0.000113149
ENSMUSG00000090164	1.49	0.000350582
ENSMUSG00000051439	1.49	0.001574916
ENSMUSG00000008318	1.49	0.001689687
ENSMUSG00000048779	1.49	0.000243019
ENSMUSG00000062593	1.49	0.002177727
ENSMUSG00000051220	1.49	0.000554907
ENSMUSG00000018983	1.48	0.009999627
ENSMUSG00000024677	1.48	2.99746E-06
ENSMUSG00000079419	1.48	0.001010319
ENSMUSG00000038059	1.48	0.015081594
ENSMUSG00000039942	1.48	0.000608408
ENSMUSG00000009687	1.47	5.64259E-08
ENSMUSG00000029675	1.47	7.46039E-06
ENSMUSG00000026042	1.47	0.002571048
ENSMUSG00000060586	1.47	0.034500514
ENSMUSG00000020399	1.46	0.004751966
ENSMUSG00000030651	1.46	0.007293486
ENSMUSG00000024910	1.45	0.00722095
ENSMUSG00000002870	1.45	0.016786613
ENSMUSG00000020077	1.45	0.000797109

Ensembl gene id	log2FC	p-adjusted
ENSMUSG00000038151	1.45	0.0001635
ENSMUSG00000052142	1.44	0.000117722
ENSMUSG00000040711	1.44	1.86968E-09
ENSMUSG00000024965	1.44	1.50344E-09
ENSMUSG00000024397	1.44	6.99353E-07
ENSMUSG00000030208	1.44	0.000358026
ENSMUSG00000004267	1.44	0.049664081
ENSMUSG00000060509	1.44	0.018910762
ENSMUSG00000056413	1.44	1.0551E-05
ENSMUSG00000024885	1.43	4.93358E-05
ENSMUSG00000024742	1.43	0.028265738
ENSMUSG00000078780	1.43	0.010198577
ENSMUSG00000038642	1.43	7.75264E-05
ENSMUSG00000049804	1.43	0.000395325
ENSMUSG00000076498	1.43	0.01096134
ENSMUSG00000026480	1.43	0.002453187
ENSMUSG00000045322	1.42	0.03165635
ENSMUSG00000048163	1.42	0.001218734
ENSMUSG00000021451	1.42	5.81502E-08
ENSMUSG00000055541	1.42	1.67804E-07
ENSMUSG00000010830	1.42	0.025568355
ENSMUSG00000027496	1.42	0.019643616
ENSMUSG00000032094	1.42	0.00529869
ENSMUSG00000021356	1.42	0.000648563
ENSMUSG00000052270	1.42	0.000329243
ENSMUSG00000031304	1.42	6.55071E-07
ENSMUSG00000027907	1.42	4.60574E-07
ENSMUSG00000059498	1.41	8.75648E-06
ENSMUSG00000049866	1.41	1.18307E-09
ENSMUSG00000022952	1.41	0.000351164
ENSMUSG00000024501	1.41	0.001188389
ENSMUSG00000026012	1.41	0.006373941
ENSMUSG00000017861	1.40	0.008153116
ENSMUSG00000032011	1.40	1.34601E-05
ENSMUSG00000048251	1.40	0.018983668
ENSMUSG00000026355	1.40	0.005543742
ENSMUSG00000025007	1.40	0.015092797
ENSMUSG00000033220	1.39	7.89645E-09
ENSMUSG00000039232	1.39	0.011086809
ENSMUSG00000047798	1.39	0.001499256
ENSMUSG00000018774	1.39	7.53696E-09
ENSMUSG00000031756	1.39	0.008299292
ENSMUSG00000051506	1.38	8.52544E-12
ENSMUSG00000029553	1.38	2.47792E-08
ENSMUSG00000037035	1.38	0.016443354
ENSMUSG00000030220	1.38	2.40994E-13
ENSMUSG00000030742	1.38	0.004797409
ENSMUSG00000027514	1.38	0.000676389
ENSMUSG00000079523	1.38	1.7404E-07
ENSMUSG00000042684	1.37	0.01313051
ENSMUSG00000024353	1.37	0.008678919
ENSMUSG00000028859	1.37	0.003385134

Ensembl gene id	log2FC	p-adjusted
ENSMUSG00000034593	1.37	1.94253E-05
ENSMUSG00000021322	1.37	0.024307027
ENSMUSG00000029188	1.37	0.018435318
ENSMUSG00000015355	1.37	2.25011E-05
ENSMUSG00000057135	1.37	3.569E-05
ENSMUSG00000021250	1.37	0.019013265
ENSMUSG00000048612	1.37	8.01133E-12
ENSMUSG00000032344	1.37	0.009817489
ENSMUSG00000001025	1.36	0.012582938
ENSMUSG00000032374	1.36	0.002556581
ENSMUSG00000036931	1.36	0.048989303
ENSMUSG00000027555	1.36	0.003264176
ENSMUSG00000020182	1.36	6.69161E-07
ENSMUSG00000013707	1.36	7.54436E-05
ENSMUSG00000024300	1.36	6.30984E-08
ENSMUSG00000003882	1.36	0.005117832
ENSMUSG00000000244	1.36	0.011682052
ENSMUSG00000015947	1.35	0.00400396
ENSMUSG00000074141	1.35	3.78931E-05
ENSMUSG00000076928	1.35	0.027375619
ENSMUSG00000055805	1.35	1.56957E-08
ENSMUSG00000020901	1.35	0.000313228
ENSMUSG00000030745	1.35	0.015092797
ENSMUSG00000031838	1.35	0.000399985
ENSMUSG00000098112	1.34	3.96516E-06
ENSMUSG00000006519	1.34	5.98774E-06
ENSMUSG00000032091	1.34	0.004530184
ENSMUSG00000028268	1.34	0.001245987
ENSMUSG00000071715	1.34	0.010465393
ENSMUSG00000015950	1.34	4.40835E-10
ENSMUSG00000030137	1.34	0.012275519
ENSMUSG00000076609	1.34	0.001686861
ENSMUSG00000043091	1.34	0.033022513
ENSMUSG00000030148	1.34	0.004096144
ENSMUSG00000031165	1.34	6.72356E-05
ENSMUSG00000022102	1.34	2.52635E-06
ENSMUSG00000076617	1.34	0.001713687
ENSMUSG00000091649	1.34	0.002723175
ENSMUSG00000072235	1.34	0.001029955
ENSMUSG00000030577	1.33	0.000121493
ENSMUSG00000040616	1.33	1.29906E-06
ENSMUSG00000031101	1.33	0.004046862
ENSMUSG00000005540	1.33	0.00045166
ENSMUSG00000056145	1.33	0.012389244
ENSMUSG00000041313	1.33	0.001511586
ENSMUSG00000072214	1.33	0.045558276
ENSMUSG00000002699	1.33	2.70133E-07
ENSMUSG00000048782	1.33	5.20251E-05
ENSMUSG00000026104	1.33	0.000501171
ENSMUSG00000024696	1.32	0.002223614
ENSMUSG00000027339	1.32	6.45805E-07
ENSMUSG00000002297	1.32	0.011338861

Ensembl gene id	log2FC	p-adjusted
ENSMUSG00000059326	1.32	1.09571E-05
ENSMUSG00000011256	1.32	2.25765E-06
ENSMUSG00000018008	1.32	3.66745E-05
ENSMUSG00000039936	1.32	2.40312E-05
ENSMUSG00000027330	1.32	0.002144371
ENSMUSG00000028581	1.32	5.00992E-07
ENSMUSG00000005360	1.32	0.011474902
ENSMUSG00000018507	1.31	7.46039E-06
ENSMUSG00000020785	1.31	0.035502463
ENSMUSG00000052336	1.31	0.000949409
ENSMUSG00000049625	1.31	3.33126E-07
ENSMUSG00000053977	1.30	0.025007067
ENSMUSG00000041112	1.30	4.63706E-07
ENSMUSG00000041859	1.30	0.000338135
ENSMUSG00000056220	1.30	0.000448119
ENSMUSG00000001444	1.30	0.047539939
ENSMUSG00000031906	1.30	8.27442E-07
ENSMUSG00000048534	1.29	0.041678928
ENSMUSG00000041515	1.29	6.28994E-06
ENSMUSG00000014158	1.29	0.010544124
ENSMUSG00000026288	1.29	9.60797E-09
ENSMUSG00000029298	1.29	0.005658686
ENSMUSG00000048376	1.29	2.6849E-13
ENSMUSG00000037020	1.29	1.95186E-05
ENSMUSG00000044811	1.29	0.000218266
ENSMUSG00000032691	1.29	0.001239803
ENSMUSG00000028671	1.29	3.90995E-09
ENSMUSG00000054720	1.28	4.8965E-09
ENSMUSG00000021457	1.28	5.31427E-05
ENSMUSG00000026395	1.28	4.12783E-12
ENSMUSG00000055994	1.28	0.024032773
ENSMUSG00000032231	1.28	5.98038E-05
ENSMUSG00000026110	1.28	0.000115576
ENSMUSG00000034595	1.27	3.57934E-10
ENSMUSG00000022488	1.27	4.97814E-07
ENSMUSG00000029923	1.27	0.007573667
ENSMUSG00000030830	1.27	2.12832E-05
ENSMUSG00000039396	1.27	0.018727172
ENSMUSG00000030156	1.27	0.014082501
ENSMUSG00000036944	1.27	0.000274466
ENSMUSG00000022831	1.27	3.15028E-10
ENSMUSG00000026547	1.27	0.00018603
ENSMUSG00000031264	1.27	2.86001E-06
ENSMUSG00000024691	1.27	0.00060612
ENSMUSG00000042759	1.26	0.000645536
ENSMUSG00000024521	1.26	0.000479156
ENSMUSG00000036752	1.26	5.26688E-07
ENSMUSG00000026068	1.26	0.01459005
ENSMUSG00000040990	1.26	3.50979E-07
ENSMUSG00000074802	1.26	3.2634E-06
ENSMUSG00000029925	1.26	1.317E-05
ENSMUSG00000051457	1.25	0.000168967

Ensembl gene id	log2FC	p-adjusted
ENSMUSG00000029304	1.25	0.04321826
ENSMUSG00000018168	1.25	0.00242042
ENSMUSG00000020788	1.25	6.23977E-06
ENSMUSG00000000628	1.25	4.56404E-06
ENSMUSG00000079227	1.25	0.003323927
ENSMUSG00000008193	1.25	0.000590575
ENSMUSG00000034330	1.25	0.00089422
ENSMUSG00000070436	1.25	0.00040759
ENSMUSG00000029009	1.24	1.57891E-06
ENSMUSG00000002111	1.24	5.3009E-05
ENSMUSG00000038252	1.24	1.12657E-06
ENSMUSG00000001918	1.24	0.000644443
ENSMUSG00000029272	1.24	0.007156853
ENSMUSG00000056394	1.24	0.017963476
ENSMUSG00000022489	1.24	0.004705159
ENSMUSG00000003153	1.24	0.002445134
ENSMUSG00000032093	1.24	0.005265266
ENSMUSG00000037613	1.24	0.005576762
ENSMUSG00000024013	1.23	8.18693E-05
ENSMUSG00000031904	1.23	0.023751353
ENSMUSG00000032436	1.23	0.001815925
ENSMUSG00000022180	1.23	4.25881E-05
ENSMUSG00000024044	1.23	0.00209996
ENSMUSG00000029322	1.23	0.020404563
ENSMUSG00000037217	1.23	0.034414128
ENSMUSG00000073599	1.23	1.93213E-05
ENSMUSG00000037379	1.23	0.037036064
ENSMUSG00000068101	1.23	0.000386293
ENSMUSG00000072082	1.22	0.012245051
ENSMUSG00000049775	1.22	3.64712E-07
ENSMUSG00000076937	1.22	0.021351277
ENSMUSG00000054892	1.22	0.009142522
ENSMUSG00000028019	1.22	3.11409E-06
ENSMUSG00000062210	1.22	0.006338264
ENSMUSG00000079362	1.22	0.010421672
ENSMUSG00000039697	1.22	5.10671E-05
ENSMUSG00000097768	1.22	0.045522793
ENSMUSG00000034255	1.22	0.000330352
ENSMUSG00000039264	1.22	0.012647081
ENSMUSG00000028312	1.22	2.19841E-05
ENSMUSG00000034116	1.22	6.7232E-09
ENSMUSG00000046841	1.22	0.000394054
ENSMUSG00000020388	1.22	0.001158802
ENSMUSG00000042379	1.21	0.007850761
ENSMUSG00000026180	1.21	0.028242625
ENSMUSG00000033453	1.21	0.005433301
ENSMUSG00000027293	1.21	0.000536885
ENSMUSG00000034786	1.21	0.000111733
ENSMUSG00000027843	1.21	0.00168086
ENSMUSG00000020437	1.20	9.48758E-05
ENSMUSG00000004665	1.20	3.51041E-05
ENSMUSG00000052160	1.20	6.95722E-08

Ensembl gene id	log2FC	p-adjusted
ENSMUSG00000037280	1.20	0.000511205
ENSMUSG00000035283	1.20	0.049280937
ENSMUSG00000063286	1.20	4.70987E-08
ENSMUSG00000023169	1.20	0.001027083
ENSMUSG00000055675	1.20	0.001757159
ENSMUSG00000021624	1.20	0.000296637
ENSMUSG00000043263	1.19	0.014385993
ENSMUSG00000003283	1.19	1.40486E-05
ENSMUSG00000087150	1.19	0.017401817
ENSMUSG00000030427	1.19	0.001156647
ENSMUSG00000036526	1.19	0.005998164
ENSMUSG00000032113	1.19	0.035936325
ENSMUSG00000054640	1.19	0.018135176
ENSMUSG00000029413	1.19	1.12478E-06
ENSMUSG00000020407	1.19	0.003822145
ENSMUSG00000058715	1.19	0.001860802
ENSMUSG00000057058	1.19	0.039638665
ENSMUSG00000041798	1.19	0.000263382
ENSMUSG00000019850	1.18	4.44548E-05
ENSMUSG00000070427	1.18	1.83981E-05
ENSMUSG00000090877	1.18	0.039978316
ENSMUSG00000031827	1.18	8.10825E-10
ENSMUSG00000002944	1.18	2.43967E-13
ENSMUSG00000049744	1.18	0.00580453
ENSMUSG00000027955	1.18	0.001858259
ENSMUSG00000039193	1.18	0.042672258
ENSMUSG00000040522	1.17	6.90172E-06
ENSMUSG00000031538	1.17	0.025753759
ENSMUSG00000096727	1.17	0.000269812
ENSMUSG00000049988	1.17	0.004674122
ENSMUSG00000045165	1.17	0.00580453
ENSMUSG00000051735	1.17	5.61388E-05
ENSMUSG00000030724	1.17	0.000248638
ENSMUSG00000027699	1.17	9.48349E-06
ENSMUSG00000074874	1.17	0.000449845
ENSMUSG00000021175	1.17	0.003323927
ENSMUSG00000024556	1.17	0.001126711
ENSMUSG00000020363	1.16	0.027207815
ENSMUSG00000002983	1.16	5.30741E-05
ENSMUSG00000008845	1.16	0.000644711
ENSMUSG00000073412	1.16	0.006053371
ENSMUSG00000052013	1.16	0.001621389
ENSMUSG00000037321	1.16	0.003642256
ENSMUSG00000003379	1.16	0.002071998
ENSMUSG00000037661	1.15	0.034788494
ENSMUSG00000020806	1.15	1.31663E-07
ENSMUSG00000028159	1.15	0.000640868
ENSMUSG00000027204	1.15	6.95722E-08
ENSMUSG00000049517	1.15	0.010956778
ENSMUSG00000030022	1.15	1.83221E-05
ENSMUSG00000027611	1.15	0.009379279
ENSMUSG00000021886	1.15	0.000115548

Ensembl gene id	log2FC	p-adjusted
ENSMUSG00000018819	1.15	2.60113E-07
ENSMUSG00000027459	1.15	0.001434448
ENSMUSG00000018486	1.15	0.001877279
ENSMUSG00000037731	1.15	0.002966003
ENSMUSG00000075602	1.15	0.012413841
ENSMUSG00000039126	1.14	0.000722882
ENSMUSG00000032271	1.14	0.007070367
ENSMUSG00000031709	1.14	0.00011909
ENSMUSG00000001281	1.14	3.47222E-06
ENSMUSG00000051998	1.14	0.010584236
ENSMUSG00000025758	1.14	0.0080852
ENSMUSG00000089942	1.14	0.033179225
ENSMUSG00000057191	1.13	0.005922717
ENSMUSG00000022103	1.13	0.010164417
ENSMUSG00000049313	1.13	4.53715E-05
ENSMUSG00000062515	1.13	0.00034587
ENSMUSG00000005672	1.13	2.14381E-05
ENSMUSG00000064254	1.13	3.78286E-11
ENSMUSG00000031502	1.12	2.52817E-12
ENSMUSG00000074743	1.12	3.08896E-05
ENSMUSG00000059970	1.12	6.71694E-06
ENSMUSG00000075304	1.12	0.021068256
ENSMUSG00000029581	1.12	3.89386E-06
ENSMUSG00000048865	1.12	0.000144271
ENSMUSG00000026009	1.12	0.003500449
ENSMUSG00000030774	1.12	0.000109013
ENSMUSG00000046805	1.12	0.000138611
ENSMUSG00000030245	1.11	6.45882E-06
ENSMUSG00000049690	1.11	0.003055921
ENSMUSG00000025888	1.11	6.53259E-06
ENSMUSG00000027452	1.11	0.007233487
ENSMUSG00000059791	1.11	0.011507524
ENSMUSG00000026548	1.11	0.014396713
ENSMUSG00000026177	1.11	3.23264E-05
ENSMUSG00000040747	1.11	0.000125266
ENSMUSG00000024621	1.11	0.000322816
ENSMUSG00000030536	1.11	2.91824E-11
ENSMUSG00000022673	1.10	0.005039601
ENSMUSG00000030878	1.10	0.023471259
ENSMUSG00000022667	1.10	0.004530184
ENSMUSG00000015340	1.10	4.99933E-11
ENSMUSG00000037972	1.10	5.02682E-05
ENSMUSG00000028059	1.10	5.39145E-06
ENSMUSG00000078920	1.10	0.000520466
ENSMUSG00000025185	1.10	0.035268183
ENSMUSG00000036896	1.10	0.003977274
ENSMUSG00000052760	1.10	0.018705429
ENSMUSG00000033685	1.10	4.50922E-05
ENSMUSG00000049037	1.09	5.1732E-05
ENSMUSG00000069792	1.09	0.025691292
ENSMUSG00000023805	1.09	4.72644E-07
ENSMUSG00000018654	1.09	1.67144E-06

Ensembl gene id	log2FC	p-adjusted
ENSMUSG00000004707	1.09	0.014046215
ENSMUSG00000023004	1.09	5.00193E-05
ENSMUSG00000036905	1.09	0.003021835
ENSMUSG00000003352	1.09	0.027510639
ENSMUSG00000015396	1.09	0.004124635
ENSMUSG00000059430	1.09	0.006384115
ENSMUSG00000050234	1.09	0.00251618
ENSMUSG00000019139	1.08	0.009630016
ENSMUSG00000028369	1.08	0.029598097
ENSMUSG00000020101	1.08	2.94031E-08
ENSMUSG00000040247	1.08	0.008230019
ENSMUSG00000027490	1.08	0.001840901
ENSMUSG00000056708	1.08	2.11825E-06
ENSMUSG00000029484	1.08	1.60278E-05
ENSMUSG00000031207	1.08	1.34688E-05
ENSMUSG00000034271	1.08	0.01774302
ENSMUSG00000034205	1.08	0.006221447
ENSMUSG00000030659	1.08	0.000589015
ENSMUSG00000034675	1.08	0.021241086
ENSMUSG00000047250	1.08	3.24794E-05
ENSMUSG00000028028	1.08	0.000111008
ENSMUSG00000031103	1.08	4.47626E-05
ENSMUSG00000073490	1.08	0.000245287
ENSMUSG00000020143	1.07	2.40581E-05
ENSMUSG00000043004	1.07	3.23149E-05
ENSMUSG00000019564	1.07	0.006533894
ENSMUSG00000002458	1.07	0.004078402
ENSMUSG00000069874	1.07	0.023557783
ENSMUSG00000006360	1.07	0.00018603
ENSMUSG00000024014	1.07	0.006152627
ENSMUSG00000093930	1.07	5.49315E-09
ENSMUSG00000035031	1.06	0.013305063
ENSMUSG00000020423	1.06	0.000407227
ENSMUSG00000021365	1.06	3.10786E-07
ENSMUSG00000078771	1.06	0.000670415
ENSMUSG00000040552	1.05	0.001029955
ENSMUSG00000026786	1.05	3.70385E-06
ENSMUSG00000047230	1.05	3.10786E-07
ENSMUSG00000048126	1.05	4.73857E-08
ENSMUSG00000027995	1.05	1.4585E-05
ENSMUSG00000026837	1.05	8.80387E-06
ENSMUSG00000057948	1.05	0.003014112
ENSMUSG00000019823	1.05	0.004107549
ENSMUSG00000022436	1.05	0.000118181
ENSMUSG00000051504	1.05	0.015364452
ENSMUSG00000041538	1.04	0.029669634
ENSMUSG00000044092	1.04	0.038292573
ENSMUSG00000040212	1.04	0.002232623
ENSMUSG00000026360	1.04	1.17446E-05
ENSMUSG00000043421	1.04	0.001986122
ENSMUSG00000039959	1.04	2.29878E-08
ENSMUSG00000036469	1.04	0.00016885

Ensembl gene id	log2FC	p-adjusted
ENSMUSG00000026821	1.04	5.45049E-05
ENSMUSG00000035095	1.04	0.000308084
ENSMUSG00000024338	1.04	4.9892E-05
ENSMUSG00000027199	1.04	0.000195759
ENSMUSG00000037405	1.04	8.25861E-07
ENSMUSG00000030844	1.03	0.001702637
ENSMUSG00000073902	1.03	3.04382E-06
ENSMUSG00000037946	1.03	0.001125188
ENSMUSG00000001473	1.03	0.000818836
ENSMUSG00000040345	1.03	0.000156664
ENSMUSG00000040061	1.03	0.000175064
ENSMUSG00000027322	1.02	0.00982841
ENSMUSG00000047810	1.02	0.001750732
ENSMUSG00000031659	1.02	1.14121E-06
ENSMUSG00000029059	1.02	0.032977344
ENSMUSG00000035439	1.02	0.000685472
ENSMUSG00000036943	1.02	1.87299E-07
ENSMUSG00000032089	1.02	4.32781E-05
ENSMUSG00000070034	1.02	0.000771929
ENSMUSG00000026773	1.02	1.57891E-06
ENSMUSG00000022148	1.02	4.50922E-05
ENSMUSG00000024843	1.02	0.002547136
ENSMUSG00000026566	1.02	0.00254299
ENSMUSG00000026421	1.02	0.006338264
ENSMUSG00000050989	1.02	0.000677078
ENSMUSG00000052727	1.02	0.038722865
ENSMUSG00000052889	1.01	0.000115137
ENSMUSG00000024399	1.01	0.002198438
ENSMUSG00000036006	1.01	0.001065171
ENSMUSG00000006585	1.01	0.036793536
ENSMUSG00000025044	1.01	2.36159E-05
ENSMUSG00000040592	1.01	0.000959215
ENSMUSG00000097415	1.01	0.008062991
ENSMUSG00000044534	1.01	0.001032504
ENSMUSG00000012519	1.01	2.8523E-05
ENSMUSG00000068227	1.01	0.005589296
ENSMUSG00000026070	1.01	0.006409415
ENSMUSG00000030759	1.00	7.17538E-06
ENSMUSG00000033287	1.00	0.001259769
ENSMUSG00000022360	1.00	0.025695704
ENSMUSG00000033788	1.00	2.8523E-05
ENSMUSG00000001555	1.00	0.022821577
ENSMUSG00000002033	0.98	0.013148295
ENSMUSG00000021263	0.91	0.018129205
ENSMUSG00000097993	0.90	0.031384399
ENSMUSG00000033538	0.86	0.006960001
ENSMUSG00000085156	0.84	0.012568464
ENSMUSG00000020695	0.83	0.03081836
ENSMUSG00000037447	0.82	0.00580453
ENSMUSG00000042579	-1.00	0.003500449
ENSMUSG00000019726	-1.00	7.25114E-07
ENSMUSG00000027016	-1.00	5.20251E-05

Ensembl gene id	log2FC	p-adjusted
ENSMUSG00000018727	-1.02	0.007128072
ENSMUSG00000099146	-1.02	3.5678E-09
ENSMUSG00000036492	-1.03	4.29422E-05
ENSMUSG00000022025	-1.03	2.0204E-05
ENSMUSG00000027253	-1.04	1.33496E-07
ENSMUSG00000026691	-1.04	0.002327236
ENSMUSG00000022389	-1.05	7.7938E-12
ENSMUSG00000029167	-1.05	0.002063159
ENSMUSG00000053964	-1.06	5.10671E-05
ENSMUSG00000041044	-1.07	0.000467124
ENSMUSG00000037348	-1.07	7.95213E-05
ENSMUSG00000021097	-1.08	2.04651E-08
ENSMUSG00000046532	-1.09	0.009063266
ENSMUSG00000022347	-1.10	0.026854468
ENSMUSG00000030244	-1.11	1.31867E-05
ENSMUSG00000001334	-1.12	6.86316E-05
ENSMUSG00000027762	-1.12	0.001879051
ENSMUSG00000001995	-1.13	0.000593638
ENSMUSG00000062181	-1.13	0.000407227
ENSMUSG00000028527	-1.13	3.50714E-07
ENSMUSG00000020893	-1.15	0.027345216
ENSMUSG00000020122	-1.16	0.003079135
ENSMUSG00000031016	-1.16	3.92799E-08
ENSMUSG00000021259	-1.16	4.17437E-05
ENSMUSG00000059824	-1.16	1.37717E-05
ENSMUSG00000036885	-1.16	0.000158316
ENSMUSG00000097762	-1.17	0.000613903
ENSMUSG00000095385	-1.18	0.000413193
ENSMUSG00000006777	-1.18	0.048219839
ENSMUSG00000069170	-1.18	0.035535412
ENSMUSG00000027296	-1.18	0.001216981
ENSMUSG00000029563	-1.19	7.46039E-06
ENSMUSG00000087478	-1.19	0.020757439
ENSMUSG00000028150	-1.21	1.63669E-05
ENSMUSG00000036027	-1.21	0.031289924
ENSMUSG00000044505	-1.21	0.040539396
ENSMUSG00000044139	-1.22	0.007619178
ENSMUSG00000052392	-1.23	3.66134E-06
ENSMUSG00000041237	-1.23	0.002280509
ENSMUSG00000029151	-1.23	0.021968212
ENSMUSG00000086670	-1.24	0.040190806
ENSMUSG00000052981	-1.24	0.021682776
ENSMUSG00000097730	-1.25	2.6751E-05
ENSMUSG00000075511	-1.25	4.62801E-07
ENSMUSG00000050069	-1.26	4.73145E-06
ENSMUSG00000048087	-1.26	0.00097438
ENSMUSG00000085834	-1.26	0.028656817
ENSMUSG00000034780	-1.26	0.001193525
ENSMUSG00000028976	-1.28	0.014061623
ENSMUSG00000028957	-1.29	2.59086E-06
ENSMUSG00000074254	-1.29	1.08728E-09
ENSMUSG00000063455	-1.29	0.009453407

Ensembl gene id	log2FC	p-adjusted
ENSMUSG00000032773	-1.30	0.002628389
ENSMUSG00000030510	-1.30	0.010405757
ENSMUSG00000084132	-1.30	0.017222966
ENSMUSG00000091405	-1.31	0.013006756
ENSMUSG00000034645	-1.31	8.88748E-06
ENSMUSG00000022041	-1.32	0.001029955
ENSMUSG00000028240	-1.33	0.002144371
ENSMUSG00000021573	-1.33	8.94195E-13
ENSMUSG00000001095	-1.35	0.005200184
ENSMUSG00000089694	-1.36	3.30765E-05
ENSMUSG00000024992	-1.37	0.016464019
ENSMUSG00000092075	-1.39	0.043152859
ENSMUSG00000079662	-1.39	0.001216981
ENSMUSG00000005677	-1.39	7.86536E-07
ENSMUSG00000026475	-1.40	0.037958226
ENSMUSG00000023087	-1.40	0.008817903
ENSMUSG00000037847	-1.41	5.26688E-07
ENSMUSG00000003555	-1.42	1.50344E-09
ENSMUSG00000031618	-1.44	1.34398E-14
ENSMUSG00000032010	-1.46	4.07183E-05
ENSMUSG00000027796	-1.49	0.027676203
ENSMUSG00000021228	-1.49	0.010709309
ENSMUSG00000042834	-1.50	0.035436871
ENSMUSG00000021260	-1.51	0.009742941
ENSMUSG00000052407	-1.51	0.000321149
ENSMUSG00000023800	-1.58	4.00255E-07
ENSMUSG00000000903	-1.58	0.011587019
ENSMUSG00000055561	-1.60	0.021037532
ENSMUSG00000040828	-1.60	0.012017045
ENSMUSG00000028051	-1.61	0.00122671
ENSMUSG00000041567	-1.61	4.16917E-10
ENSMUSG00000017688	-1.61	0.009199671
ENSMUSG00000071691	-1.62	0.046395257
ENSMUSG00000072944	-1.64	0.049653675
ENSMUSG00000000739	-1.64	0.000324685
ENSMUSG00000040093	-1.65	0.004282691
ENSMUSG00000082065	-1.69	0.006858715
ENSMUSG00000024558	-1.69	0.041133937
ENSMUSG00000041333	-1.71	0.005692926
ENSMUSG00000049985	-1.72	0.032468834
ENSMUSG00000078680	-1.72	0.000198684
ENSMUSG00000042671	-1.73	0.029669634
ENSMUSG00000089873	-1.75	0.000424768
ENSMUSG00000078689	-1.77	0.000233232
ENSMUSG00000058523	-1.78	6.36355E-05
ENSMUSG00000078688	-1.79	0.000129516
ENSMUSG00000094793	-1.79	8.64937E-05
ENSMUSG00000086253	-1.80	0.005270879
ENSMUSG00000096674	-1.80	0.000121908
ENSMUSG00000027376	-1.81	0.010216471
ENSMUSG00000078674	-1.83	6.81405E-05
ENSMUSG00000078673	-1.83	6.96146E-05

Ensembl gene id	log2FC	p-adjusted
ENSMUSG00000078683	-1.83	0.000117113
ENSMUSG00000096688	-1.83	0.000153041
ENSMUSG00000020623	-1.84	2.25555E-18
ENSMUSG00000078687	-1.84	0.000116994
ENSMUSG00000079495	-1.85	0.005215197
ENSMUSG00000057425	-1.85	0.003971897
ENSMUSG00000095532	-1.85	2.6751E-05
ENSMUSG00000078675	-1.86	7.46471E-05
ENSMUSG00000073842	-1.87	1.56318E-05
ENSMUSG00000082363	-1.87	0.000339895
ENSMUSG00000073830	-1.87	6.80165E-05
ENSMUSG00000078686	-1.87	0.000430398
ENSMUSG00000073834	-1.88	4.77279E-05
ENSMUSG00000088022	-1.88	0.006931909
ENSMUSG00000081053	-1.94	5.80839E-05
ENSMUSG00000027871	-1.96	1.12477E-05
ENSMUSG00000048424	-2.07	1.69204E-06
ENSMUSG00000090622	-2.09	3.39338E-08
ENSMUSG00000055775	-2.09	0.005639861
ENSMUSG00000080700	-2.11	0.004784123
ENSMUSG00000082173	-2.16	0.016740149
ENSMUSG00000027014	-2.19	1.13348E-05
ENSMUSG00000083478	-2.24	4.23316E-06
ENSMUSG00000091648	-2.30	0.009611432
ENSMUSG00000051497	-2.38	0.019543638
ENSMUSG00000073835	-2.44	1.12659E-11
ENSMUSG00000021198	-2.52	0.002327236
ENSMUSG00000081207	-2.67	0.000117113
ENSMUSG00000094222	-2.69	0.005802032
ENSMUSG00000078685	-2.73	0.000216676
ENSMUSG00000050974	-2.82	0.020136366
ENSMUSG00000062647	-2.89	7.18377E-08
ENSMUSG00000079465	-2.94	0.031351778
ENSMUSG00000038092	-3.05	3.11373E-29
ENSMUSG00000096652	-3.40	0.002241795
ENSMUSG00000005220	-3.55	0.006006581
ENSMUSG00000094948	-3.64	0.005389282
ENSMUSG00000031736	-3.70	0.003433741
ENSMUSG00000050663	-3.70	4.86031E-05
ENSMUSG00000081049	-4.18	0.006311791
ENSMUSG00000059668	-4.27	0.006181234
ENSMUSG00000038541	-6.70	0.029670779
**Sm_Vh_35**		
ENSMUSG00000000290	9.20	0.000346052
ENSMUSG00000063779	8.52	4.39878E-16
ENSMUSG00000024211	7.68	0.030518233
ENSMUSG00000076655	6.16	2.22147E-07
ENSMUSG00000044162	5.53	0.000163608
ENSMUSG00000095120	5.32	0.000279845
ENSMUSG00000094749	5.27	9.27491E-24
ENSMUSG00000076614	5.21	5.65557E-85
ENSMUSG00000031933	5.11	0.00343567

Ensembl gene id	log2FC	p-adjusted
ENSMUSG00000022157	5.03	0.002130125
ENSMUSG00000066364	4.92	0.018394062
ENSMUSG00000021697	4.85	0.0230903
ENSMUSG00000037990	4.84	4.18313E-05
ENSMUSG00000044071	4.58	0.008577192
ENSMUSG00000064147	4.52	4.43882E-10
ENSMUSG00000046006	4.35	2.42507E-05
ENSMUSG00000009185	4.17	1.14808E-13
ENSMUSG00000013974	3.89	0.00343567
ENSMUSG00000036322	3.86	2.66659E-18
ENSMUSG00000093861	3.83	0.041257307
ENSMUSG00000022651	3.78	2.8577E-07
ENSMUSG00000068606	3.75	0.020400324
ENSMUSG00000041468	3.73	0.038401339
ENSMUSG00000016498	3.72	6.08522E-05
ENSMUSG00000050578	3.69	0.007210931
ENSMUSG00000020676	3.51	2.07019E-05
ENSMUSG00000063388	3.48	0.033439108
ENSMUSG00000026011	3.38	0.001556062
ENSMUSG00000035186	3.34	0.043239762
ENSMUSG00000041481	3.34	9.89707E-08
ENSMUSG00000044309	3.31	6.25382E-05
ENSMUSG00000095170	3.30	0.034198265
ENSMUSG00000087642	3.17	0.004312586
ENSMUSG00000040152	3.03	2.73388E-12
ENSMUSG00000050014	3.03	0.035345767
ENSMUSG00000057465	3.01	7.71279E-12
ENSMUSG00000022126	2.93	0.017148207
ENSMUSG00000025473	2.81	0.001776088
ENSMUSG00000026582	2.81	0.00340881
ENSMUSG00000094335	2.77	0.031226099
ENSMUSG00000091345	2.73	3.10026E-06
ENSMUSG00000025804	2.69	0.001287664
ENSMUSG00000078922	2.65	0.038304072
ENSMUSG00000026822	2.62	1.23282E-05
ENSMUSG00000037868	2.61	0.00197012
ENSMUSG00000031289	2.56	0.000110789
ENSMUSG00000074115	2.55	3.70344E-07
ENSMUSG00000078921	2.54	0.004698763
ENSMUSG00000089929	2.52	0.009883942
ENSMUSG00000004814	2.48	1.97175E-07
ENSMUSG00000024791	2.48	0.004774618
ENSMUSG00000029417	2.45	0.028028426
ENSMUSG00000030789	2.44	0.000434319
ENSMUSG00000024401	2.43	0.021265001
ENSMUSG00000022534	2.43	1.75426E-08
ENSMUSG00000056054	2.42	0.007659767
ENSMUSG00000061100	2.40	0.007278712
ENSMUSG00000079547	2.40	8.72704E-08
ENSMUSG00000024353	2.38	0.005167472
ENSMUSG00000034855	2.38	0.02670593
ENSMUSG00000029352	2.37	0.009803407

Ensembl gene id	log2FC	p-adjusted
ENSMUSG00000028270	2.33	0.013612389
ENSMUSG00000039013	2.33	0.00687128
ENSMUSG00000023908	2.32	0.001771893
ENSMUSG00000041202	2.30	0.000155111
ENSMUSG00000061578	2.27	0.007011317
ENSMUSG00000076609	2.26	2.62316E-23
ENSMUSG00000096422	2.26	0.005520102
ENSMUSG00000085963	2.24	0.007955938
ENSMUSG00000076617	2.24	1.96525E-27
ENSMUSG00000004709	2.23	0.000500367
ENSMUSG00000053318	2.22	0.039217186
ENSMUSG00000018924	2.18	9.01058E-05
ENSMUSG00000073421	2.17	2.48348E-17
ENSMUSG00000056071	2.15	0.008556205
ENSMUSG00000029272	2.14	0.009219441
ENSMUSG00000091694	2.14	0.007165228
ENSMUSG00000082292	2.14	0.035478545
ENSMUSG00000003484	2.12	0.00879198
ENSMUSG00000017499	2.11	0.006401415
ENSMUSG00000062545	2.11	0.000106174
ENSMUSG00000022057	2.11	2.01032E-07
ENSMUSG00000026630	2.10	3.79561E-06
ENSMUSG00000035683	2.09	0.00567823
ENSMUSG00000027656	2.08	0.026850775
ENSMUSG00000026875	2.07	1.09189E-05
ENSMUSG00000036594	2.07	0.000272351
ENSMUSG00000067149	2.06	1.53466E-12
ENSMUSG00000037649	2.06	1.64487E-05
ENSMUSG00000022504	2.05	0.013557142
ENSMUSG00000020897	2.04	0.00450317
ENSMUSG00000060044	2.03	1.71071E-05
ENSMUSG00000079293	2.03	3.40045E-05
ENSMUSG00000018930	2.03	0.005849142
ENSMUSG00000015437	2.03	0.005055143
ENSMUSG00000023132	2.02	0.00120529
ENSMUSG00000044827	2.02	2.69654E-05
ENSMUSG00000052726	2.02	0.007571088
ENSMUSG00000024334	2.02	0.00343567
ENSMUSG00000000682	2.02	4.39878E-16
ENSMUSG00000034634	2.00	4.86774E-06
ENSMUSG00000026573	2.00	0.017324232
ENSMUSG00000042817	2.00	0.005310834
ENSMUSG00000062939	1.99	0.000445661
ENSMUSG00000061577	1.98	0.007929419
ENSMUSG00000019942	1.98	0.020903447
ENSMUSG00000031662	1.97	0.000166898
ENSMUSG00000024989	1.95	0.005543591
ENSMUSG00000085887	1.95	0.023863642
ENSMUSG00000087450	1.95	0.007669854
ENSMUSG00000035385	1.94	0.012148616
ENSMUSG00000056737	1.92	2.05054E-12
ENSMUSG00000048922	1.92	0.026841368

Ensembl gene id	log2FC	p-adjusted
ENSMUSG00000014030	1.92	0.027015941
ENSMUSG00000030468	1.91	0.001776088
ENSMUSG00000062524	1.90	0.000418485
ENSMUSG00000038037	1.90	0.033349077
ENSMUSG00000036067	1.89	0.009510258
ENSMUSG00000018927	1.89	0.008865331
ENSMUSG00000090298	1.87	0.024152513
ENSMUSG00000026390	1.87	0.000114706
ENSMUSG00000024910	1.86	0.03206432
ENSMUSG00000024610	1.85	5.78611E-14
ENSMUSG00000024670	1.84	0.007318071
ENSMUSG00000032053	1.83	2.98492E-06
ENSMUSG00000015134	1.82	0.004687898
ENSMUSG00000028480	1.81	6.14348E-05
ENSMUSG00000020914	1.81	0.005662262
ENSMUSG00000034438	1.80	0.02203487
ENSMUSG00000027398	1.80	1.64276E-10
ENSMUSG00000070000	1.79	0.00701362
ENSMUSG00000027347	1.79	0.013708001
ENSMUSG00000021423	1.79	2.69654E-05
ENSMUSG00000028459	1.78	0.011948518
ENSMUSG00000078853	1.78	0.019641732
ENSMUSG00000020057	1.78	0.004896144
ENSMUSG00000083798	1.77	0.006539487
ENSMUSG00000056498	1.77	0.001271284
ENSMUSG00000048852	1.77	0.036197417
ENSMUSG00000078763	1.76	0.005599431
ENSMUSG00000076937	1.76	0.000815503
ENSMUSG00000055809	1.76	0.034599764
ENSMUSG00000051998	1.75	0.004111167
ENSMUSG00000026683	1.73	0.005637087
ENSMUSG00000024669	1.73	0.043579392
ENSMUSG00000005667	1.73	0.003062423
ENSMUSG00000024397	1.72	1.27628E-05
ENSMUSG00000017652	1.72	0.002313678
ENSMUSG00000016496	1.72	0.030295387
ENSMUSG00000026628	1.72	0.033453312
ENSMUSG00000037725	1.71	0.02762058
ENSMUSG00000030124	1.71	0.004470332
ENSMUSG00000022439	1.71	0.000393938
ENSMUSG00000040528	1.71	0.000839202
ENSMUSG00000090164	1.71	0.012953991
ENSMUSG00000063193	1.70	1.52974E-09
ENSMUSG00000059089	1.70	8.36372E-13
ENSMUSG00000027797	1.69	0.043110558
ENSMUSG00000029298	1.69	0.025693628
ENSMUSG00000026069	1.69	0.032141016
ENSMUSG00000034266	1.69	0.011596964
ENSMUSG00000043931	1.68	0.004590476
ENSMUSG00000005540	1.68	0.000136771
ENSMUSG00000024679	1.67	5.10128E-11
ENSMUSG00000079363	1.67	0.040718495

Ensembl gene id	log2FC	p-adjusted
ENSMUSG00000042254	1.67	0.014759093
ENSMUSG00000026123	1.67	0.043470961
ENSMUSG00000026271	1.67	0.001830603
ENSMUSG00000022686	1.67	0.021136546
ENSMUSG00000060586	1.66	0.02605193
ENSMUSG00000028268	1.66	0.021949373
ENSMUSG00000050335	1.66	3.07168E-12
ENSMUSG00000085913	1.66	0.048813296
ENSMUSG00000075602	1.66	0.011653364
ENSMUSG00000071714	1.65	3.23229E-05
ENSMUSG00000024737	1.65	1.2099E-09
ENSMUSG00000031506	1.64	0.010551195
ENSMUSG00000001020	1.64	0.008675652
ENSMUSG00000023078	1.64	0.007319169
ENSMUSG00000046031	1.64	0.01880997
ENSMUSG00000040751	1.64	0.00479308
ENSMUSG00000030165	1.64	0.005421006
ENSMUSG00000039264	1.63	0.00197012
ENSMUSG00000066363	1.63	0.004449224
ENSMUSG00000091649	1.62	0.005270781
ENSMUSG00000029372	1.62	0.026869764
ENSMUSG00000002204	1.62	0.004433441
ENSMUSG00000027514	1.62	0.021893465
ENSMUSG00000026458	1.61	0.004660114
ENSMUSG00000040950	1.61	0.013703829
ENSMUSG00000031934	1.61	0.021005492
ENSMUSG00000024521	1.60	2.69259E-05
ENSMUSG00000035448	1.59	0.000122253
ENSMUSG00000022102	1.59	7.62987E-08
ENSMUSG00000045502	1.59	0.012143743
ENSMUSG00000073412	1.59	0.003593318
ENSMUSG00000049103	1.59	3.94805E-06
ENSMUSG00000060509	1.57	0.026963332
ENSMUSG00000005087	1.57	0.000612627
ENSMUSG00000049723	1.56	0.020122621
ENSMUSG00000037337	1.56	0.000839202
ENSMUSG00000079362	1.56	0.006491938
ENSMUSG00000048327	1.56	0.007163238
ENSMUSG00000025161	1.56	0.034599764
ENSMUSG00000040026	1.55	0.004624799
ENSMUSG00000040264	1.55	0.004771966
ENSMUSG00000049130	1.55	0.005085433
ENSMUSG00000021262	1.55	1.12611E-08
ENSMUSG00000057135	1.54	7.74384E-06
ENSMUSG00000030867	1.54	0.010112468
ENSMUSG00000002257	1.54	0.026243357
ENSMUSG00000000409	1.54	0.023232376
ENSMUSG00000031304	1.54	0.002796204
ENSMUSG00000054342	1.53	0.005410393
ENSMUSG00000026580	1.53	7.4424E-07
ENSMUSG00000021880	1.52	0.033300658
ENSMUSG00000052142	1.52	1.78311E-05

Ensembl gene id	log2FC	p-adjusted
ENSMUSG00000000486	1.52	3.2035E-05
ENSMUSG00000037217	1.52	0.009942913
ENSMUSG00000076615	1.52	0.034081209
ENSMUSG00000011008	1.51	0.033619557
ENSMUSG00000028931	1.51	0.001327339
ENSMUSG00000039232	1.51	0.002338277
ENSMUSG00000030707	1.51	6.46981E-07
ENSMUSG00000070691	1.51	0.013130497
ENSMUSG00000022372	1.50	0.005270781
ENSMUSG00000004933	1.50	0.00825766
ENSMUSG00000076490	1.49	0.014097912
ENSMUSG00000050921	1.48	0.028028615
ENSMUSG00000037548	1.48	0.000473595
ENSMUSG00000001119	1.48	1.64276E-10
ENSMUSG00000079018	1.48	0.004544466
ENSMUSG00000038642	1.47	1.94713E-13
ENSMUSG00000024677	1.47	0.024461802
ENSMUSG00000020241	1.47	8.18099E-07
ENSMUSG00000027750	1.46	0.013042554
ENSMUSG00000035232	1.46	0.023618105
ENSMUSG00000004952	1.46	3.82967E-07
ENSMUSG00000049625	1.45	0.000567832
ENSMUSG00000008318	1.45	0.013994085
ENSMUSG00000061540	1.45	1.12611E-08
ENSMUSG00000021728	1.45	0.023527727
ENSMUSG00000063286	1.44	0.00694727
ENSMUSG00000022103	1.44	0.019108504
ENSMUSG00000039699	1.44	0.000829759
ENSMUSG00000045826	1.44	0.002523611
ENSMUSG00000045322	1.44	0.006242467
ENSMUSG00000021322	1.44	0.00703391
ENSMUSG00000072620	1.44	0.00322229
ENSMUSG00000021196	1.44	2.73422E-06
ENSMUSG00000043740	1.43	1.09189E-05
ENSMUSG00000026928	1.43	0.035839337
ENSMUSG00000079419	1.43	0.006242467
ENSMUSG00000030724	1.43	4.2647E-05
ENSMUSG00000030677	1.42	0.032281099
ENSMUSG00000057058	1.42	0.025763998
ENSMUSG00000025163	1.42	0.010077579
ENSMUSG00000026360	1.42	7.44674E-09
ENSMUSG00000039942	1.42	0.007571088
ENSMUSG00000039899	1.42	0.036561983
ENSMUSG00000016283	1.42	0.011206417
ENSMUSG00000043832	1.42	2.93058E-07
ENSMUSG00000033220	1.42	1.59135E-07
ENSMUSG00000026104	1.41	0.035629799
ENSMUSG00000015396	1.41	2.8577E-07
ENSMUSG00000076613	1.41	0.035246601
ENSMUSG00000025877	1.40	0.012985813
ENSMUSG00000001444	1.40	0.008409718
ENSMUSG00000021356	1.40	0.006043159

Ensembl gene id	log2FC	p-adjusted
ENSMUSG00000032691	1.40	0.033453312
ENSMUSG00000022221	1.40	4.93195E-05
ENSMUSG00000071715	1.40	5.24562E-07
ENSMUSG00000041538	1.39	0.000441369
ENSMUSG00000032011	1.39	0.001332989
ENSMUSG00000038943	1.39	0.005593421
ENSMUSG00000059326	1.39	4.93195E-05
ENSMUSG00000034786	1.39	7.318E-05
ENSMUSG00000044811	1.39	0.000434319
ENSMUSG00000051506	1.38	0.010384117
ENSMUSG00000037166	1.38	0.011798175
ENSMUSG00000024673	1.38	0.000414692
ENSMUSG00000023274	1.37	0.035224698
ENSMUSG00000079227	1.37	0.039552762
ENSMUSG00000026480	1.37	4.9122E-07
ENSMUSG00000021298	1.37	0.01599935
ENSMUSG00000070323	1.37	0.012903801
ENSMUSG00000026009	1.37	0.001698622
ENSMUSG00000024300	1.36	1.45264E-06
ENSMUSG00000056413	1.36	0.000515788
ENSMUSG00000055116	1.36	0.03524238
ENSMUSG00000003379	1.36	0.001099883
ENSMUSG00000045763	1.35	0.001728652
ENSMUSG00000024965	1.35	7.74304E-06
ENSMUSG00000032322	1.35	0.040785734
ENSMUSG00000078920	1.35	0.015264137
ENSMUSG00000040247	1.35	0.002105726
ENSMUSG00000071713	1.35	0.004700492
ENSMUSG00000027611	1.34	0.003062423
ENSMUSG00000048612	1.34	0.011945226
ENSMUSG00000048163	1.34	6.93424E-07
ENSMUSG00000021250	1.34	0.000848146
ENSMUSG00000039936	1.34	0.004618731
ENSMUSG00000032093	1.34	0.009538385
ENSMUSG00000031838	1.34	2.15079E-10
ENSMUSG00000032094	1.33	0.013824902
ENSMUSG00000020120	1.33	0.026980991
ENSMUSG00000030220	1.33	4.33459E-05
ENSMUSG00000040592	1.33	5.55526E-05
ENSMUSG00000087107	1.33	0.013674576
ENSMUSG00000032218	1.33	0.007539165
ENSMUSG00000027408	1.33	1.27286E-05
ENSMUSG00000076928	1.33	0.01662198
ENSMUSG00000037731	1.33	0.02246185
ENSMUSG00000024349	1.33	0.000821987
ENSMUSG00000089672	1.33	0.000119751
ENSMUSG00000013707	1.32	0.000368604
ENSMUSG00000034116	1.32	7.09138E-07
ENSMUSG00000074874	1.32	3.53256E-06
ENSMUSG00000004707	1.32	0.002263824
ENSMUSG00000036905	1.32	8.15983E-11
ENSMUSG00000018507	1.32	0.000135913

Ensembl gene id	log2FC	p-adjusted
ENSMUSG00000035929	1.32	0.000187924
ENSMUSG00000009687	1.32	6.26655E-07
ENSMUSG00000040345	1.32	5.82965E-05
ENSMUSG00000022831	1.32	2.90606E-08
ENSMUSG00000052760	1.32	0.007899953
ENSMUSG00000049988	1.31	2.94955E-05
ENSMUSG00000004612	1.31	0.005598034
ENSMUSG00000024885	1.31	0.001053835
ENSMUSG00000006219	1.31	0.000209647
ENSMUSG00000030830	1.31	0.006635419
ENSMUSG00000048779	1.31	6.14348E-05
ENSMUSG00000028581	1.31	4.15815E-10
ENSMUSG00000018008	1.31	6.74184E-05
ENSMUSG00000098112	1.31	0.001921326
ENSMUSG00000015947	1.31	0.001771893
ENSMUSG00000047798	1.31	3.23229E-05
ENSMUSG00000041449	1.31	4.44738E-07
ENSMUSG00000031101	1.30	0.000839202
ENSMUSG00000096727	1.30	0.001039874
ENSMUSG00000028874	1.30	0.001243125
ENSMUSG00000002111	1.30	1.48298E-07
ENSMUSG00000026832	1.30	0.000453733
ENSMUSG00000092021	1.29	0.02298869
ENSMUSG00000047880	1.29	0.005131422
ENSMUSG00000036896	1.29	3.80783E-10
ENSMUSG00000029204	1.29	0.001776088
ENSMUSG00000041112	1.29	0.001465474
ENSMUSG00000020788	1.29	1.21225E-05
ENSMUSG00000030793	1.29	0.004791623
ENSMUSG00000052013	1.29	0.002497607
ENSMUSG00000055541	1.29	0.004470332
ENSMUSG00000051457	1.28	0.038719349
ENSMUSG00000031659	1.28	0.009855867
ENSMUSG00000022440	1.28	0.003916331
ENSMUSG00000021886	1.28	0.000184125
ENSMUSG00000038608	1.28	0.011275799
ENSMUSG00000030742	1.28	0.005447418
ENSMUSG00000022322	1.28	0.014481898
ENSMUSG00000026548	1.28	0.013631253
ENSMUSG00000035283	1.28	0.010097465
ENSMUSG00000073489	1.28	7.76957E-05
ENSMUSG00000029925	1.28	2.30538E-06
ENSMUSG00000030577	1.27	0.00010843
ENSMUSG00000023067	1.27	0.048502209
ENSMUSG00000046223	1.27	0.011289194
ENSMUSG00000036526	1.27	0.018097582
ENSMUSG00000026043	1.27	0.04761658
ENSMUSG00000058715	1.27	1.49139E-07
ENSMUSG00000024013	1.27	0.000203168
ENSMUSG00000017716	1.27	0.013268803
ENSMUSG00000059994	1.27	0.011653364
ENSMUSG00000068227	1.27	0.021560127

Ensembl gene id	log2FC	p-adjusted
ENSMUSG00000030774	1.27	0.003521611
ENSMUSG00000040663	1.26	0.034726408
ENSMUSG00000002033	1.26	0.00284072
ENSMUSG00000026288	1.26	6.74184E-05
ENSMUSG00000015950	1.26	0.000175048
ENSMUSG00000027322	1.26	0.015095697
ENSMUSG00000018774	1.25	9.80341E-08
ENSMUSG00000029553	1.25	4.75163E-05
ENSMUSG00000049775	1.25	4.43183E-10
ENSMUSG00000032436	1.25	0.000187924
ENSMUSG00000028859	1.25	0.014244545
ENSMUSG00000052160	1.25	1.27156E-06
ENSMUSG00000032089	1.25	0.000359366
ENSMUSG00000015568	1.25	7.81099E-06
ENSMUSG00000000318	1.25	0.021364055
ENSMUSG00000043263	1.25	0.036715424
ENSMUSG00000026110	1.24	0.012401686
ENSMUSG00000040990	1.24	3.73628E-05
ENSMUSG00000006519	1.24	2.9191E-07
ENSMUSG00000038151	1.24	0.015208327
ENSMUSG00000047250	1.24	1.09189E-05
ENSMUSG00000058163	1.24	0.038049972
ENSMUSG00000036887	1.24	1.90413E-09
ENSMUSG00000021457	1.23	0.000293305
ENSMUSG00000026581	1.23	0.040112905
ENSMUSG00000019874	1.23	3.34711E-06
ENSMUSG00000020901	1.23	0.001845817
ENSMUSG00000020388	1.23	0.00127951
ENSMUSG00000037860	1.23	0.000809184
ENSMUSG00000022876	1.22	0.018466536
ENSMUSG00000031165	1.22	0.033739532
ENSMUSG00000022488	1.22	0.003496811
ENSMUSG00000034591	1.22	0.00128509
ENSMUSG00000034255	1.22	0.002270907
ENSMUSG00000022901	1.22	2.32173E-05
ENSMUSG00000045165	1.22	0.021032993
ENSMUSG00000042759	1.22	0.000382045
ENSMUSG00000030798	1.22	2.45068E-05
ENSMUSG00000054640	1.22	0.004886906
ENSMUSG00000012889	1.22	0.016091298
ENSMUSG00000038508	1.22	0.005310834
ENSMUSG00000024696	1.21	0.004159167
ENSMUSG00000070427	1.21	0.001424958
ENSMUSG00000047139	1.21	0.000607044
ENSMUSG00000041515	1.21	0.006775861
ENSMUSG00000048865	1.21	0.018210884
ENSMUSG00000032491	1.21	0.013322300
ENSMUSG00000024621	1.20	1.69222E-06
ENSMUSG00000062515	1.20	3.11748E-06
ENSMUSG00000062593	1.20	4.18313E-05
ENSMUSG00000026395	1.20	0.012830117
ENSMUSG00000001506	1.20	0.026659868

Ensembl gene id	log2FC	p-adjusted
ENSMUSG00000073409	1.19	0.01585865
ENSMUSG00000034330	1.19	0.005044429
ENSMUSG00000032294	1.18	5.12824E-05
ENSMUSG00000015854	1.18	3.23229E-05
ENSMUSG00000018819	1.18	1.47471E-05
ENSMUSG00000057191	1.18	6.2895E-06
ENSMUSG00000046157	1.18	0.000252258
ENSMUSG00000069874	1.18	0.004773127
ENSMUSG00000029484	1.18	7.53022E-05
ENSMUSG00000034317	1.18	0.044056943
ENSMUSG00000023169	1.17	0.035973286
ENSMUSG00000022180	1.17	0.000839202
ENSMUSG00000070034	1.17	0.000815503
ENSMUSG00000038754	1.17	0.013505652
ENSMUSG00000015355	1.17	0.000635146
ENSMUSG00000031004	1.17	0.006957371
ENSMUSG00000040711	1.17	0.021753158
ENSMUSG00000038156	1.17	0.033797404
ENSMUSG00000018654	1.17	0.009509625
ENSMUSG00000053063	1.16	0.007468266
ENSMUSG00000042684	1.16	0.000260138
ENSMUSG00000034595	1.16	8.59145E-06
ENSMUSG00000037318	1.16	0.007044058
ENSMUSG00000031264	1.16	0.006711496
ENSMUSG00000021263	1.16	0.008594505
ENSMUSG00000027339	1.16	0.011948518
ENSMUSG00000020437	1.15	0.000129052
ENSMUSG00000054072	1.15	0.007513624
ENSMUSG00000050022	1.15	0.002263824
ENSMUSG00000003283	1.15	0.000184125
ENSMUSG00000059498	1.15	7.25037E-06
ENSMUSG00000030745	1.15	0.031090562
ENSMUSG00000026728	1.14	0.000174895
ENSMUSG00000021822	1.14	0.005394401
ENSMUSG00000024338	1.14	0.002428217
ENSMUSG00000056069	1.14	0.000504434
ENSMUSG00000028159	1.14	0.00673827
ENSMUSG00000025498	1.14	0.033333798
ENSMUSG00000018920	1.14	3.22451E-06
ENSMUSG00000029675	1.14	0.015875133
ENSMUSG00000027230	1.14	0.02920865
ENSMUSG00000037944	1.13	0.013683654
ENSMUSG00000037820	1.13	4.32892E-08
ENSMUSG00000058672	1.13	0.034855338
ENSMUSG00000023349	1.13	0.011196884
ENSMUSG00000046805	1.13	0.008711401
ENSMUSG00000031253	1.13	0.031324931
ENSMUSG00000055546	1.12	0.007468266
ENSMUSG00000015340	1.12	0.012524456
ENSMUSG00000049866	1.12	0.000166567
ENSMUSG00000028214	1.12	0.022131043
ENSMUSG00000046245	1.12	7.76957E-05

Ensembl gene id	log2FC	p-adjusted
ENSMUSG00000058099	1.12	0.001611188
ENSMUSG00000011256	1.12	0.002236218
ENSMUSG00000021725	1.12	0.022819676
ENSMUSG00000029413	1.12	0.034599764
ENSMUSG00000022148	1.11	0.006932045
ENSMUSG00000008193	1.11	0.023133398
ENSMUSG00000022952	1.10	0.024632281
ENSMUSG00000073490	1.09	0.031874595
ENSMUSG00000031389	1.09	7.648E-05
ENSMUSG00000038811	1.09	5.55526E-05
ENSMUSG00000052270	1.09	0.043007522
ENSMUSG00000026126	1.09	0.009723887
ENSMUSG00000040747	1.09	0.000101889
ENSMUSG00000052776	1.08	0.015476952
ENSMUSG00000047810	1.08	0.000277843
ENSMUSG00000024501	1.08	0.000105025
ENSMUSG00000027962	1.08	0.010853046
ENSMUSG00000033685	1.08	5.05841E-07
ENSMUSG00000020695	1.07	0.006040039
ENSMUSG00000034593	1.07	0.021364055
ENSMUSG00000060550	1.07	0.008866597
ENSMUSG00000056529	1.07	0.018954387
ENSMUSG00000029581	1.07	0.000472888
ENSMUSG00000079014	1.07	0.005044429
ENSMUSG00000030148	1.07	0.011197141
ENSMUSG00000026177	1.07	0.004725769
ENSMUSG00000049037	1.07	0.000834856
ENSMUSG00000061232	1.06	7.76957E-05
ENSMUSG00000030047	1.06	0.000538284
ENSMUSG00000022489	1.06	0.032163086
ENSMUSG00000035042	1.06	0.010124952
ENSMUSG00000033192	1.05	0.031364488
ENSMUSG00000004730	1.05	0.047598464
ENSMUSG00000036353	1.05	0.02260064
ENSMUSG00000032690	1.05	0.002967755
ENSMUSG00000055805	1.05	0.000829758
ENSMUSG00000049804	1.05	0.004425547
ENSMUSG00000026600	1.05	0.031207433
ENSMUSG00000068220	1.05	0.000245072
ENSMUSG00000050721	1.05	0.018954387
ENSMUSG00000040522	1.04	0.029097903
ENSMUSG00000068245	1.04	0.015877005
ENSMUSG00000051439	1.04	0.000473595
ENSMUSG00000022014	1.04	0.025577623
ENSMUSG00000051735	1.04	0.006155802
ENSMUSG00000023913	1.04	6.2895E-06
ENSMUSG00000037972	1.04	0.002669383
ENSMUSG00000062210	1.04	0.001776088
ENSMUSG00000037031	1.04	7.8047E-05
ENSMUSG00000052384	1.03	3.37875E-05
ENSMUSG00000005465	1.03	0.005599431
ENSMUSG00000098557	1.03	3.40045E-05

Ensembl gene id	log2FC	p-adjusted
ENSMUSG00000030579	1.03	1.27286E-05
ENSMUSG00000002458	1.03	0.006149817
ENSMUSG00000020077	1.02	4.59392E-05
ENSMUSG00000001128	1.02	3.34711E-06
ENSMUSG00000028059	1.01	0.010711296
ENSMUSG00000015745	1.01	0.000136771
ENSMUSG00000041642	1.01	0.005167472
ENSMUSG00000073411	1.01	1.32393E-06
ENSMUSG00000023277	1.01	0.002931437
ENSMUSG00000037946	1.00	0.028867926
ENSMUSG00000032246	1.00	0.045863512
ENSMUSG00000001281	1.00	0.001830603
ENSMUSG00000030263	1.00	0.000417182
ENSMUSG00000057948	1.00	0.033619557
ENSMUSG00000078771	0.98	0.0450764
ENSMUSG00000026821	0.98	0.008337091
ENSMUSG00000022186	0.96	0.005270781
ENSMUSG00000021747	-1.12	0.011780737
ENSMUSG00000053846	-1.14	1.54219E-05
ENSMUSG00000055254	-1.16	0.017479241
ENSMUSG00000041220	-1.17	1.1914E-08
ENSMUSG00000083478	-1.19	0.014616401
ENSMUSG00000018868	-1.21	0.007804947
ENSMUSG00000078452	-1.21	0.019681344
ENSMUSG00000001334	-1.21	8.45302E-05
ENSMUSG00000050195	-1.22	0.004548077
ENSMUSG00000037071	-1.23	0.018388893
ENSMUSG00000028051	-1.24	0.017033533
ENSMUSG00000090622	-1.37	2.93257E-05
ENSMUSG00000020122	-1.37	6.23873E-12
ENSMUSG00000034645	-1.48	2.45068E-05
ENSMUSG00000025202	-1.55	0.010477896
ENSMUSG00000006777	-1.55	0.001913366
ENSMUSG00000026475	-1.58	0.005734863
ENSMUSG00000066687	-1.69	0.006975227
ENSMUSG00000085834	-1.72	0.014134594
ENSMUSG00000062647	-2.85	2.72172E-07
ENSMUSG00000005220	-3.69	0.009010925
**Sm_PZQ_39**		
ENSMUSG00000063779	9.30	4.67804E-18
ENSMUSG00000040809	7.94	3.90337E-34
ENSMUSG00000009185	7.10	7.47756E-36
ENSMUSG00000001131	6.33	7.3531E-22
ENSMUSG00000058126	6.02	0.034973303
ENSMUSG00000061100	5.82	1.88211E-51
ENSMUSG00000040498	5.82	1.62894E-24
ENSMUSG00000016498	5.81	4.04585E-19
ENSMUSG00000028072	5.58	3.14076E-06
ENSMUSG00000035352	5.55	2.81118E-05
ENSMUSG00000032487	5.53	1.68607E-05
ENSMUSG00000076614	5.47	0.009425457
ENSMUSG00000020676	5.42	3.70157E-27

Ensembl gene id	log2FC	p-adjusted
ENSMUSG00000050370	5.36	8.30189E-09
ENSMUSG00000047222	5.31	5.77314E-08
ENSMUSG00000018623	5.22	0.000222989
ENSMUSG00000054196	5.20	0.001680756
ENSMUSG00000028415	5.19	3.89979E-05
ENSMUSG00000092116	5.18	0.041219218
ENSMUSG00000022651	4.93	5.10204E-16
ENSMUSG00000068452	4.90	0.001838439
ENSMUSG00000015134	4.87	2.75132E-23
ENSMUSG00000030111	4.84	0.027004289
ENSMUSG00000078521	4.64	0.048561018
ENSMUSG00000027994	4.60	0.008815373
ENSMUSG00000064272	4.54	0.006701911
ENSMUSG00000068606	4.43	9.81517E-72
ENSMUSG00000026822	4.39	1.84915E-12
ENSMUSG00000092243	4.35	0.031154007
ENSMUSG00000057729	4.33	3.88934E-07
ENSMUSG00000047992	4.32	0.019035169
ENSMUSG00000082976	4.32	1.1135E-06
ENSMUSG00000030162	4.25	2.78354E-06
ENSMUSG00000052435	4.23	0.000473212
ENSMUSG00000040197	4.22	6.19947E-06
ENSMUSG00000063388	4.19	1.65773E-14
ENSMUSG00000051314	4.17	0.000727421
ENSMUSG00000035186	4.16	5.28833E-14
ENSMUSG00000084956	4.15	0.047533669
ENSMUSG00000044309	4.14	0.022359921
ENSMUSG00000018924	4.14	9.73112E-14
ENSMUSG00000020826	4.10	1.21932E-09
ENSMUSG00000052212	4.06	0.00179636
ENSMUSG00000050578	4.04	2.76167E-34
ENSMUSG00000028965	3.97	4.20596E-06
ENSMUSG00000022126	3.96	0.00049859
ENSMUSG00000035373	3.94	1.08335E-11
ENSMUSG00000032010	3.89	9.56936E-60
ENSMUSG00000000805	3.87	1.05136E-12
ENSMUSG00000058755	3.87	6.73915E-05
ENSMUSG00000049723	3.87	5.16145E-12
ENSMUSG00000057465	3.85	7.07885E-15
ENSMUSG00000059824	3.84	3.01619E-26
ENSMUSG00000021953	3.77	0.000613287
ENSMUSG00000038550	3.73	0.010268598
ENSMUSG00000091345	3.73	1.07794E-14
ENSMUSG00000070645	3.72	0.001786481
ENSMUSG00000074115	3.71	2.81965E-21
ENSMUSG00000021758	3.70	0.000237344
ENSMUSG00000072599	3.70	0.000230049
ENSMUSG00000062380	3.70	0.000215944
ENSMUSG00000026011	3.69	0.000431207
ENSMUSG00000031162	3.66	0.000508306
ENSMUSG00000037474	3.65	0.021175643
ENSMUSG00000032271	3.64	7.00939E-14

Ensembl gene id	log2FC	p-adjusted
ENSMUSG00000072601	3.62	0.025376704
ENSMUSG00000087213	3.62	0.011117334
ENSMUSG00000033213	3.61	0.020592811
ENSMUSG00000030268	3.61	0.00025569
ENSMUSG00000064147	3.59	3.78618E-12
ENSMUSG00000082292	3.54	1.36891E-22
ENSMUSG00000046591	3.53	0.018521597
ENSMUSG00000062345	3.51	1.03872E-06
ENSMUSG00000063903	3.50	0.045334116
ENSMUSG00000053318	3.50	7.9279E-16
ENSMUSG00000072596	3.48	1.79337E-05
ENSMUSG00000042345	3.47	9.97423E-05
ENSMUSG00000098318	3.46	0.007838405
ENSMUSG00000042385	3.44	0.005288393
ENSMUSG00000025804	3.42	5.44127E-16
ENSMUSG00000007888	3.39	0.045570674
ENSMUSG00000019890	3.36	0.024173322
ENSMUSG00000031016	3.36	8.81634E-34
ENSMUSG00000035455	3.35	0.014552947
ENSMUSG00000052133	3.34	4.94329E-05
ENSMUSG00000024401	3.34	0.000780283
ENSMUSG00000031762	3.34	3.41864E-13
ENSMUSG00000018930	3.33	5.62465E-08
ENSMUSG00000092920	3.32	0.002227107
ENSMUSG00000044162	3.31	0.000145992
ENSMUSG00000044827	3.30	1.24162E-23
ENSMUSG00000027656	3.30	4.38185E-10
ENSMUSG00000025473	3.29	1.04648E-13
ENSMUSG00000030413	3.28	6.33264E-08
ENSMUSG00000022479	3.28	0.000172945
ENSMUSG00000025701	3.27	0.000151202
ENSMUSG00000031495	3.27	0.004128053
ENSMUSG00000030283	3.24	0.009119014
ENSMUSG00000001228	3.23	0.009575619
ENSMUSG00000040950	3.23	4.00274E-14
ENSMUSG00000091954	3.23	0.004798798
ENSMUSG00000078922	3.22	2.70745E-17
ENSMUSG00000037868	3.22	1.3421E-09
ENSMUSG00000090622	3.18	5.20976E-19
ENSMUSG00000046031	3.18	1.61164E-24
ENSMUSG00000026285	3.17	0.01981635
ENSMUSG00000028587	3.16	9.98193E-05
ENSMUSG00000040026	3.16	1.78308E-10
ENSMUSG00000017499	3.15	0.005026374
ENSMUSG00000029811	3.15	0.000468872
ENSMUSG00000045502	3.15	0.000299765
ENSMUSG00000073386	3.14	0.032684464
ENSMUSG00000039013	3.13	5.71488E-07
ENSMUSG00000026390	3.13	1.08251E-47
ENSMUSG00000020279	3.13	0.000452445
ENSMUSG00000026429	3.13	0.01250198
ENSMUSG00000091573	3.12	0.008956784

Ensembl gene id	log2FC	p-adjusted
ENSMUSG00000021464	3.12	0.006030723
ENSMUSG00000053338	3.12	0.006022078
ENSMUSG00000002835	3.12	0.031216615
ENSMUSG00000084839	3.10	0.001911449
ENSMUSG00000061540	3.08	4.46268E-08
ENSMUSG00000005410	3.07	0.006010929
ENSMUSG00000003484	3.07	1.30122E-05
ENSMUSG00000004791	3.06	0.023657572
ENSMUSG00000054203	3.06	0.029785283
ENSMUSG00000078921	3.05	3.79723E-17
ENSMUSG00000060586	3.04	3.16803E-47
ENSMUSG00000004814	3.04	4.81405E-36
ENSMUSG00000024529	3.04	1.41073E-13
ENSMUSG00000031562	3.04	0.012140907
ENSMUSG00000083679	3.03	0.004468455
ENSMUSG00000032586	3.03	0.013727117
ENSMUSG00000038037	3.01	2.24099E-09
ENSMUSG00000035165	3.01	0.000762542
ENSMUSG00000015314	3.00	1.80877E-10
ENSMUSG00000089929	2.99	7.19076E-07
ENSMUSG00000022422	2.98	0.013416293
ENSMUSG00000026981	2.98	5.30854E-15
ENSMUSG00000029380	2.96	0.01583277
ENSMUSG00000001506	2.95	3.38476E-16
ENSMUSG00000028957	2.95	7.88469E-45
ENSMUSG00000028362	2.94	0.002408091
ENSMUSG00000064246	2.92	1.36328E-11
ENSMUSG00000026546	2.90	0.006758096
ENSMUSG00000033847	2.90	0.005288393
ENSMUSG00000000682	2.89	2.39395E-49
ENSMUSG00000037466	2.88	0.006481973
ENSMUSG00000055170	2.87	0.003423883
ENSMUSG00000006585	2.86	0.044435202
ENSMUSG00000028270	2.85	2.9883E-28
ENSMUSG00000068744	2.85	0.025043058
ENSMUSG00000034855	2.85	3.88592E-16
ENSMUSG00000034773	2.85	0.015770293
ENSMUSG00000042489	2.84	0.036232901
ENSMUSG00000087611	2.83	0.016811769
ENSMUSG00000069910	2.82	0.026729072
ENSMUSG00000031896	2.82	0.008041272
ENSMUSG00000041754	2.82	0.030145691
ENSMUSG00000031779	2.82	1.85708E-07
ENSMUSG00000022033	2.82	0.0171503
ENSMUSG00000022226	2.81	0.041779997
ENSMUSG00000030200	2.81	0.019661675
ENSMUSG00000020702	2.81	0.019374149
ENSMUSG00000082902	2.81	0.000643718
ENSMUSG00000013766	2.79	0.007003201
ENSMUSG00000033762	2.79	6.16206E-09
ENSMUSG00000030789	2.76	3.02629E-26
ENSMUSG00000016283	2.76	8.77201E-07

Ensembl gene id	log2FC	p-adjusted
ENSMUSG00000024675	2.76	5.31012E-13
ENSMUSG00000024989	2.76	0.014432055
ENSMUSG00000074034	2.74	0.009456464
ENSMUSG00000056737	2.74	2.15751E-32
ENSMUSG00000046006	2.73	1.4418E-05
ENSMUSG00000031933	2.73	0.005455378
ENSMUSG00000001865	2.71	0.022115918
ENSMUSG00000040675	2.70	1.19811E-19
ENSMUSG00000022584	2.68	1.66215E-05
ENSMUSG00000067341	2.68	0.001705172
ENSMUSG00000078853	2.67	2.4838E-32
ENSMUSG00000022015	2.66	0.031586712
ENSMUSG00000030402	2.66	0.031319249
ENSMUSG00000096965	2.66	0.031034473
ENSMUSG00000030077	2.66	0.004768372
ENSMUSG00000041202	2.66	0.000132333
ENSMUSG00000021062	2.65	0.009198354
ENSMUSG00000005696	2.65	0.006975246
ENSMUSG00000018927	2.65	7.81021E-17
ENSMUSG00000027073	2.64	0.011133327
ENSMUSG00000029417	2.64	7.53755E-14
ENSMUSG00000045273	2.64	0.012341413
ENSMUSG00000043931	2.64	1.87938E-12
ENSMUSG00000038379	2.63	0.012012876
ENSMUSG00000019214	2.63	0.015443073
ENSMUSG00000030346	2.63	0.014079830
ENSMUSG00000071552	2.63	0.029169653
ENSMUSG00000058427	2.62	0.017207571
ENSMUSG00000098021	2.62	0.00348934
ENSMUSG00000020581	2.61	0.024462847
ENSMUSG00000000982	2.61	0.003585468
ENSMUSG00000027323	2.60	0.009818823
ENSMUSG00000030144	2.60	0.017763135
ENSMUSG00000026630	2.60	4.56347E-14
ENSMUSG00000034634	2.60	3.58978E-08
ENSMUSG00000064109	2.60	0.000702781
ENSMUSG00000079445	2.56	0.035679836
ENSMUSG00000092021	2.56	1.71447E-28
ENSMUSG00000005540	2.56	8.59934E-14
ENSMUSG00000002870	2.56	0.002228282
ENSMUSG00000036587	2.56	0.000196611
ENSMUSG00000037411	2.55	7.63034E-05
ENSMUSG00000085708	2.53	0.034900168
ENSMUSG00000028268	2.53	3.39432E-12
ENSMUSG00000079293	2.53	1.48615E-29
ENSMUSG00000037548	2.53	1.37849E-24
ENSMUSG00000087273	2.53	0.000718319
ENSMUSG00000041774	2.52	0.004817993
ENSMUSG00000037991	2.52	0.014262742
ENSMUSG00000076490	2.52	0.000102215
ENSMUSG00000053977	2.52	8.65482E-07
ENSMUSG00000017737	2.51	2.95446E-06

Ensembl gene id	log2FC	p-adjusted
ENSMUSG00000027379	2.51	0.000323262
ENSMUSG00000034266	2.50	1.54854E-07
ENSMUSG00000024028	2.50	0.012396892
ENSMUSG00000020057	2.50	1.13418E-17
ENSMUSG00000023992	2.50	5.35057E-09
ENSMUSG00000020383	2.50	0.049318963
ENSMUSG00000058252	2.50	0.048706561
ENSMUSG00000004267	2.50	0.000142613
ENSMUSG00000051225	2.50	4.77115E-07
ENSMUSG00000039055	2.49	0.032434497
ENSMUSG00000030156	2.49	0.001835076
ENSMUSG00000035042	2.49	2.02528E-13
ENSMUSG00000046341	2.48	0.005027846
ENSMUSG00000050335	2.48	2.86915E-48
ENSMUSG00000037649	2.46	2.20498E-23
ENSMUSG00000049103	2.46	3.9483E-30
ENSMUSG00000019590	2.46	0.011881728
ENSMUSG00000029414	2.46	0.020976424
ENSMUSG00000022322	2.46	0.024852715
ENSMUSG00000015316	2.45	0.000708218
ENSMUSG00000027368	2.43	4.93441E-05
ENSMUSG00000037628	2.42	0.034691247
ENSMUSG00000046295	2.42	0.012770564
ENSMUSG00000041859	2.42	0.019823289
ENSMUSG00000025582	2.42	0.000898546
ENSMUSG00000039187	2.42	0.02191036
ENSMUSG00000041481	2.41	1.84481E-31
ENSMUSG00000073421	2.41	1.61428E-17
ENSMUSG00000047880	2.41	9.37213E-06
ENSMUSG00000021384	2.41	0.014983568
ENSMUSG00000042265	2.40	0.010422992
ENSMUSG00000025912	2.40	0.018700676
ENSMUSG00000030867	2.40	0.005608604
ENSMUSG00000095649	2.40	0.005491634
ENSMUSG00000076937	2.40	2.56866E-07
ENSMUSG00000024334	2.40	8.99899E-05
ENSMUSG00000040899	2.39	0.014656495
ENSMUSG00000022673	2.39	0.00745121
ENSMUSG00000079455	2.38	0.038699861
ENSMUSG00000027514	2.38	6.38708E-25
ENSMUSG00000079547	2.38	2.73079E-07
ENSMUSG00000035683	2.38	2.04562E-06
ENSMUSG00000002068	2.38	0.015893504
ENSMUSG00000024672	2.37	9.71048E-09
ENSMUSG00000045826	2.37	6.76437E-06
ENSMUSG00000025161	2.37	2.40884E-12
ENSMUSG00000002228	2.36	0.000226565
ENSMUSG00000031142	2.36	0.017437234
ENSMUSG00000020808	2.36	0.00591155
ENSMUSG00000022945	2.36	0.02063852
ENSMUSG00000050410	2.36	0.006848843
ENSMUSG00000079363	2.36	8.14099E-13

Ensembl gene id	log2FC	p-adjusted
ENSMUSG00000047592	2.35	0.00902523
ENSMUSG00000024791	2.35	0.01232203
ENSMUSG00000030004	2.35	0.014790989
ENSMUSG00000004612	2.35	6.18781E-12
ENSMUSG00000021880	2.35	4.72867E-07
ENSMUSG00000001020	2.34	1.13636E-12
ENSMUSG00000037544	2.33	0.015054032
ENSMUSG00000059108	2.33	0.002150806
ENSMUSG00000036067	2.33	2.03838E-06
ENSMUSG00000020897	2.33	0.02567757
ENSMUSG00000040907	2.33	1.31079E-06
ENSMUSG00000004933	2.33	5.01575E-08
ENSMUSG00000023132	2.32	2.31946E-09
ENSMUSG00000005470	2.32	0.007538667
ENSMUSG00000059994	2.31	3.13342E-05
ENSMUSG00000024056	2.31	0.039743398
ENSMUSG00000026355	2.31	0.009872473
ENSMUSG00000051998	2.31	3.73136E-06
ENSMUSG00000002033	2.30	5.93248E-14
ENSMUSG00000051444	2.30	6.04209E-07
ENSMUSG00000028364	2.30	8.31265E-18
ENSMUSG00000040592	2.29	5.12351E-09
ENSMUSG00000026683	2.29	0.040775954
ENSMUSG00000031289	2.28	1.89897E-13
ENSMUSG00000032322	2.28	1.90504E-08
ENSMUSG00000096727	2.27	1.69736E-25
ENSMUSG00000051969	2.27	0.004443953
ENSMUSG00000029272	2.27	0.03276568
ENSMUSG00000020377	2.27	0.044789702
ENSMUSG00000059089	2.26	6.43815E-15
ENSMUSG00000028460	2.26	0.019481054
ENSMUSG00000053398	2.26	0.020148523
ENSMUSG00000040152	2.26	6.39769E-29
ENSMUSG00000037321	2.26	1.18046E-53
ENSMUSG00000026582	2.25	0.048931151
ENSMUSG00000084796	2.25	0.001750583
ENSMUSG00000017652	2.25	2.32308E-10
ENSMUSG00000005800	2.25	0.030862946
ENSMUSG00000048852	2.24	5.54884E-06
ENSMUSG00000020798	2.24	0.044213471
ENSMUSG00000029915	2.23	0.0060624
ENSMUSG00000030742	2.23	0.003023474
ENSMUSG00000024670	2.23	4.05813E-07
ENSMUSG00000085887	2.23	0.000412624
ENSMUSG00000024610	2.22	9.73621E-30
ENSMUSG00000013974	2.22	0.011316256
ENSMUSG00000002204	2.21	2.14271E-08
ENSMUSG00000074345	2.21	0.000202829
ENSMUSG00000027408	2.20	6.92628E-24
ENSMUSG00000021262	2.20	4.31056E-25
ENSMUSG00000030468	2.20	3.9264E-06
ENSMUSG00000048191	2.20	4.76219E-06

Ensembl gene id	log2FC	p-adjusted
ENSMUSG00000078963	2.20	0.023966253
ENSMUSG00000075602	2.19	6.72792E-22
ENSMUSG00000016496	2.19	9.55344E-09
ENSMUSG00000031906	2.19	6.16163E-14
ENSMUSG00000034438	2.19	0.001162262
ENSMUSG00000020241	2.19	2.57569E-14
ENSMUSG00000055809	2.19	0.000731898
ENSMUSG00000027863	2.19	9.91559E-08
ENSMUSG00000030793	2.18	1.12799E-11
ENSMUSG00000030474	2.18	3.6057E-21
ENSMUSG00000053044	2.18	0.011812871
ENSMUSG00000019942	2.18	0.008195326
ENSMUSG00000020914	2.17	0.008634708
ENSMUSG00000039774	2.17	0.015255739
ENSMUSG00000024669	2.17	0.000656421
ENSMUSG00000026875	2.16	3.84152E-09
ENSMUSG00000095788	2.16	0.025545438
ENSMUSG00000070000	2.16	1.72793E-05
ENSMUSG00000023908	2.15	0.011753143
ENSMUSG00000061132	2.15	0.000960196
ENSMUSG00000079018	2.15	1.09138E-15
ENSMUSG00000073412	2.15	5.20746E-12
ENSMUSG00000049580	2.15	1.67759E-15
ENSMUSG00000036594	2.14	1.66471E-18
ENSMUSG00000021175	2.14	1.99325E-06
ENSMUSG00000050994	2.14	0.041416894
ENSMUSG00000029923	2.13	0.001800171
ENSMUSG00000076609	2.13	0.007291724
ENSMUSG00000021624	2.13	5.34524E-16
ENSMUSG00000045087	2.12	0.040695183
ENSMUSG00000039981	2.12	8.18947E-07
ENSMUSG00000022440	2.11	8.85588E-16
ENSMUSG00000035385	2.11	1.31605E-06
ENSMUSG00000053063	2.10	5.93106E-11
ENSMUSG00000068129	2.10	0.027071313
ENSMUSG00000087060	2.09	0.015058667
ENSMUSG00000076617	2.09	0.017437234
ENSMUSG00000017716	2.09	0.01074863
ENSMUSG00000024301	2.09	0.021753618
ENSMUSG00000037348	2.09	1.68702E-06
ENSMUSG00000027490	2.08	0.010826224
ENSMUSG00000003379	2.07	3.98862E-07
ENSMUSG00000021423	2.07	2.40884E-12
ENSMUSG00000030577	2.07	1.12135E-17
ENSMUSG00000022389	2.07	4.92837E-51
ENSMUSG00000024397	2.07	2.08354E-27
ENSMUSG00000073705	2.06	0.006276955
ENSMUSG00000078920	2.06	1.87947E-12
ENSMUSG00000040264	2.06	0.002046035
ENSMUSG00000014030	2.06	0.003715946
ENSMUSG00000045763	2.06	1.36761E-11
ENSMUSG00000045679	2.05	3.69008E-07

Ensembl gene id	log2FC	p-adjusted
ENSMUSG00000093861	2.05	0.018764031
ENSMUSG00000051220	2.05	0.009248745
ENSMUSG00000030724	2.04	4.87536E-07
ENSMUSG00000040751	2.03	4.68883E-07
ENSMUSG00000000791	2.03	0.001564956
ENSMUSG00000030170	2.02	8.637E-16
ENSMUSG00000024679	2.01	8.02248E-23
ENSMUSG00000021614	2.01	1.95713E-05
ENSMUSG00000041538	2.01	1.35933E-09
ENSMUSG00000076498	2.01	7.20891E-05
ENSMUSG00000003541	2.00	1.67253E-07
ENSMUSG00000048534	2.00	2.76372E-05
ENSMUSG00000021298	2.00	7.5889E-05
ENSMUSG00000056498	1.99	1.06434E-05
ENSMUSG00000078763	1.99	1.74814E-08
ENSMUSG00000029661	1.99	1.59286E-15
ENSMUSG00000034059	1.99	0.014578424
ENSMUSG00000029161	1.99	0.028060089
ENSMUSG00000044811	1.98	1.69019E-11
ENSMUSG00000044505	1.98	1.61168E-07
ENSMUSG00000010142	1.97	1.16012E-05
ENSMUSG00000015619	1.97	0.012675569
ENSMUSG00000081067	1.97	0.041906305
ENSMUSG00000001119	1.97	1.32541E-24
ENSMUSG00000055541	1.97	1.7947E-10
ENSMUSG00000026649	1.96	0.015731253
ENSMUSG00000022586	1.96	0.001605299
ENSMUSG00000031304	1.96	3.56811E-16
ENSMUSG00000038264	1.96	0.002815532
ENSMUSG00000023033	1.96	0.01389043
ENSMUSG00000061577	1.96	5.88803E-05
ENSMUSG00000031385	1.96	0.040695183
ENSMUSG00000050921	1.96	2.51594E-05
ENSMUSG00000030137	1.95	2.57825E-06
ENSMUSG00000029075	1.95	0.004409381
ENSMUSG00000037337	1.95	4.76511E-08
ENSMUSG00000042474	1.95	1.14212E-05
ENSMUSG00000030707	1.95	3.85395E-09
ENSMUSG00000022504	1.95	1.12124E-09
ENSMUSG00000032400	1.95	0.015245336
ENSMUSG00000034987	1.94	0.03311111
ENSMUSG00000094335	1.94	0.021448975
ENSMUSG00000032011	1.94	1.52975E-13
ENSMUSG00000025351	1.94	1.01815E-15
ENSMUSG00000062510	1.93	0.005943733
ENSMUSG00000026271	1.93	2.27891E-08
ENSMUSG00000083890	1.93	0.006722038
ENSMUSG00000020974	1.93	5.80755E-06
ENSMUSG00000045868	1.93	0.007633057
ENSMUSG00000071714	1.93	3.68085E-21
ENSMUSG00000029204	1.93	2.95565E-10
ENSMUSG00000029322	1.93	1.64285E-29

Ensembl gene id	log2FC	p-adjusted
ENSMUSG00000067591	1.93	0.007467232
ENSMUSG00000032113	1.93	0.010583300
ENSMUSG00000027331	1.93	0.013851448
ENSMUSG00000022034	1.92	0.029935946
ENSMUSG00000000817	1.92	0.020747014
ENSMUSG00000026581	1.92	5.96837E-12
ENSMUSG00000026080	1.92	0.010509885
ENSMUSG00000057058	1.92	0.000694464
ENSMUSG00000025586	1.92	5.04843E-09
ENSMUSG00000051811	1.92	0.010743116
ENSMUSG00000053101	1.91	0.018212574
ENSMUSG00000024300	1.91	2.87331E-27
ENSMUSG00000071005	1.91	0.001911954
ENSMUSG00000001025	1.91	6.05972E-06
ENSMUSG00000000486	1.90	9.28037E-11
ENSMUSG00000026104	1.90	1.5833E-44
ENSMUSG00000028718	1.90	0.011688799
ENSMUSG00000039942	1.89	1.89453E-06
ENSMUSG00000091694	1.89	0.049318963
ENSMUSG00000037725	1.89	0.027680123
ENSMUSG00000070691	1.89	0.000236801
ENSMUSG00000027962	1.89	8.90023E-34
ENSMUSG00000022102	1.89	4.60347E-18
ENSMUSG00000013155	1.89	0.042041303
ENSMUSG00000031838	1.89	1.14762E-37
ENSMUSG00000087187	1.89	0.041135357
ENSMUSG00000022385	1.89	0.008431861
ENSMUSG00000085786	1.88	0.02506179
ENSMUSG00000020077	1.88	1.74375E-25
ENSMUSG00000015437	1.88	0.000336793
ENSMUSG00000021822	1.88	4.97786E-07
ENSMUSG00000017861	1.88	0.00725291
ENSMUSG00000026069	1.88	0.00012598
ENSMUSG00000042041	1.88	2.40548E-07
ENSMUSG00000046971	1.88	0.035657531
ENSMUSG00000018819	1.87	5.82828E-22
ENSMUSG00000020732	1.86	0.001747988
ENSMUSG00000024353	1.86	0.023657572
ENSMUSG00000051212	1.86	0.000992104
ENSMUSG00000068105	1.86	0.001226387
ENSMUSG00000041707	1.86	2.89296E-06
ENSMUSG00000002257	1.86	2.12875E-05
ENSMUSG00000032053	1.86	2.32641E-07
ENSMUSG00000041498	1.85	0.025250717
ENSMUSG00000025058	1.85	0.000387902
ENSMUSG00000025877	1.85	5.75661E-11
ENSMUSG00000052760	1.85	1.98916E-06
ENSMUSG00000070034	1.85	1.29027E-11
ENSMUSG00000034612	1.85	0.000340809
ENSMUSG00000022021	1.85	0.048020898
ENSMUSG00000030641	1.84	0.019487745
ENSMUSG00000040247	1.84	4.99554E-05

Ensembl gene id	log2FC	p-adjusted
ENSMUSG00000045751	1.84	0.006007568
ENSMUSG00000028459	1.84	3.46696E-11
ENSMUSG00000028976	1.84	0.000422564
ENSMUSG00000072620	1.83	3.5087E-24
ENSMUSG00000028359	1.83	0.039743398
ENSMUSG00000024399	1.83	4.87169E-11
ENSMUSG00000069874	1.83	1.10017E-08
ENSMUSG00000002111	1.83	1.36891E-22
ENSMUSG00000054717	1.83	0.01050058
ENSMUSG00000074570	1.83	0.026086204
ENSMUSG00000035448	1.82	0.017119469
ENSMUSG00000037731	1.82	8.03632E-09
ENSMUSG00000021263	1.82	0.000247363
ENSMUSG00000038179	1.82	0.045144577
ENSMUSG00000026042	1.82	4.03956E-22
ENSMUSG00000015355	1.82	3.12444E-16
ENSMUSG00000000903	1.82	0.006623137
ENSMUSG00000033220	1.82	1.86335E-29
ENSMUSG00000015880	1.82	0.009562089
ENSMUSG00000050107	1.82	0.013891697
ENSMUSG00000026580	1.81	1.04213E-08
ENSMUSG00000002055	1.81	0.018796674
ENSMUSG00000026928	1.81	2.26991E-05
ENSMUSG00000000204	1.80	0.015516018
ENSMUSG00000074896	1.80	2.5756E-20
ENSMUSG00000056413	1.80	2.09652E-10
ENSMUSG00000097415	1.80	8.81459E-08
ENSMUSG00000091649	1.80	0.001286334
ENSMUSG00000030798	1.79	3.91779E-08
ENSMUSG00000036634	1.79	0.011234761
ENSMUSG00000000409	1.79	6.52043E-12
ENSMUSG00000045775	1.79	0.002446666
ENSMUSG00000039264	1.79	3.45089E-07
ENSMUSG00000028212	1.79	0.017472412
ENSMUSG00000024338	1.79	3.38534E-36
ENSMUSG00000062753	1.79	5.42176E-07
ENSMUSG00000049775	1.79	9.71051E-41
ENSMUSG00000032093	1.78	0.000690549
ENSMUSG00000085761	1.78	0.005175549
ENSMUSG00000093677	1.78	0.009143762
ENSMUSG00000043832	1.78	5.09139E-17
ENSMUSG00000047798	1.78	1.16749E-05
ENSMUSG00000045328	1.78	0.016409078
ENSMUSG00000030149	1.78	1.64223E-10
ENSMUSG00000006519	1.77	2.1936E-23
ENSMUSG00000078798	1.77	3.19322E-05
ENSMUSG00000097993	1.77	0.01498332
ENSMUSG00000097317	1.77	0.037942549
ENSMUSG00000052013	1.77	9.33928E-08
ENSMUSG00000078780	1.77	2.6729E-06
ENSMUSG00000030677	1.77	0.00889568
ENSMUSG00000073489	1.76	2.06881E-05

Ensembl gene id	log2FC	p-adjusted
ENSMUSG00000043740	1.76	2.51559E-05
ENSMUSG00000062545	1.76	3.41864E-13
ENSMUSG00000038642	1.76	1.3414E-17
ENSMUSG00000025163	1.76	2.51592E-07
ENSMUSG00000026573	1.76	0.017095874
ENSMUSG00000027797	1.75	0.002076033
ENSMUSG00000000318	1.75	1.56221E-07
ENSMUSG00000021596	1.75	0.023048428
ENSMUSG00000068220	1.75	1.0746E-18
ENSMUSG00000026043	1.74	5.65592E-30
ENSMUSG00000078122	1.74	9.30344E-06
ENSMUSG00000030365	1.74	0.010204992
ENSMUSG00000047139	1.74	6.37837E-14
ENSMUSG00000098090	1.74	0.032705977
ENSMUSG00000002633	1.73	0.029925035
ENSMUSG00000049037	1.73	1.14541E-13
ENSMUSG00000048327	1.73	0.016421943
ENSMUSG00000012889	1.73	4.75369E-05
ENSMUSG00000043015	1.73	3.38303E-05
ENSMUSG00000015981	1.73	0.032813182
ENSMUSG00000009687	1.73	1.42943E-20
ENSMUSG00000038768	1.72	0.01023858
ENSMUSG00000050350	1.72	0.034129798
ENSMUSG00000051517	1.72	0.013999625
ENSMUSG00000032218	1.72	0.033771958
ENSMUSG00000021714	1.72	0.017119586
ENSMUSG00000003283	1.72	4.33983E-15
ENSMUSG00000079523	1.72	9.19232E-15
ENSMUSG00000057135	1.72	5.52981E-10
ENSMUSG00000024910	1.72	1.84588E-05
ENSMUSG00000062593	1.71	1.7854E-15
ENSMUSG00000029309	1.71	0.011084292
ENSMUSG00000030148	1.71	2.36099E-07
ENSMUSG00000004707	1.70	8.52238E-16
ENSMUSG00000020787	1.70	0.049600896
ENSMUSG00000085873	1.70	0.000326216
ENSMUSG00000028873	1.70	0.014748169
ENSMUSG00000071715	1.70	1.32455E-16
ENSMUSG00000030220	1.70	1.77627E-23
ENSMUSG00000051457	1.70	3.94976E-10
ENSMUSG00000028885	1.70	0.021666179
ENSMUSG00000097039	1.70	6.78221E-05
ENSMUSG00000028555	1.69	4.2022E-09
ENSMUSG00000055235	1.69	0.000948495
ENSMUSG00000019845	1.69	0.007187649
ENSMUSG00000005547	1.69	6.94285E-15
ENSMUSG00000026126	1.69	6.7148E-08
ENSMUSG00000047757	1.68	0.000914966
ENSMUSG00000034906	1.68	0.009451324
ENSMUSG00000026068	1.68	8.72312E-05
ENSMUSG00000018774	1.67	2.47885E-24
ENSMUSG00000078249	1.67	0.000174431

Ensembl gene id	log2FC	p-adjusted
ENSMUSG00000028111	1.67	0.001949172
ENSMUSG00000029910	1.67	0.005931234
ENSMUSG00000089672	1.67	0.000269149
ENSMUSG00000079553	1.67	0.035195594
ENSMUSG00000031101	1.67	3.89228E-14
ENSMUSG00000030142	1.67	0.011342957
ENSMUSG00000052270	1.67	4.05813E-07
ENSMUSG00000027995	1.67	3.30873E-12
ENSMUSG00000032094	1.67	1.81672E-05
ENSMUSG00000039899	1.67	1.72698E-05
ENSMUSG00000031662	1.66	2.14413E-05
ENSMUSG00000069792	1.66	2.44711E-22
ENSMUSG00000015950	1.66	3.13619E-12
ENSMUSG00000074623	1.66	5.49276E-05
ENSMUSG00000040329	1.66	1.61227E-05
ENSMUSG00000046223	1.66	1.68607E-05
ENSMUSG00000060509	1.66	0.000672984
ENSMUSG00000037095	1.66	0.006052252
ENSMUSG00000067149	1.66	0.031290365
ENSMUSG00000030165	1.66	0.010704589
ENSMUSG00000031740	1.65	1.30937E-06
ENSMUSG00000022057	1.65	4.86877E-09
ENSMUSG00000053862	1.65	0.019508887
ENSMUSG00000027907	1.65	5.98157E-10
ENSMUSG00000013584	1.65	0.024006444
ENSMUSG00000026009	1.65	1.19131E-05
ENSMUSG00000028463	1.65	0.025597112
ENSMUSG00000021356	1.65	0.000513637
ENSMUSG00000022686	1.65	0.005642415
ENSMUSG00000005233	1.65	0.013266151
ENSMUSG00000029359	1.65	0.005120698
ENSMUSG00000056394	1.64	0.047282233
ENSMUSG00000030579	1.64	2.93128E-26
ENSMUSG00000024885	1.64	9.68246E-09
ENSMUSG00000024673	1.64	8.69685E-08
ENSMUSG00000034853	1.64	1.35423E-15
ENSMUSG00000094786	1.64	0.02293228
ENSMUSG00000074419	1.64	0.021125743
ENSMUSG00000039699	1.64	6.25006E-05
ENSMUSG00000027715	1.64	0.012599178
ENSMUSG00000030786	1.64	3.16933E-12
ENSMUSG00000015947	1.64	4.03329E-12
ENSMUSG00000005087	1.64	4.44228E-10
ENSMUSG00000032311	1.63	1.18227E-12
ENSMUSG00000022103	1.63	8.60412E-08
ENSMUSG00000071068	1.63	0.001662581
ENSMUSG00000030745	1.63	1.66812E-06
ENSMUSG00000020437	1.63	2.15E-13
ENSMUSG00000036768	1.63	0.006596436
ENSMUSG00000030589	1.63	0.001231862
ENSMUSG00000024036	1.63	5.27703E-13
ENSMUSG00000063193	1.62	1.79739E-10

Ensembl gene id	log2FC	p-adjusted
ENSMUSG00000056069	1.62	1.08973E-11
ENSMUSG00000031264	1.62	3.43934E-05
ENSMUSG00000022657	1.62	0.02501166
ENSMUSG00000078771	1.62	4.85917E-06
ENSMUSG00000052142	1.62	1.85662E-09
ENSMUSG00000070323	1.61	0.003277639
ENSMUSG00000034116	1.61	5.25882E-17
ENSMUSG00000038421	1.61	0.002232995
ENSMUSG00000062939	1.61	0.000190317
ENSMUSG00000034786	1.61	1.09474E-09
ENSMUSG00000062488	1.60	2.93925E-10
ENSMUSG00000032773	1.60	0.025686603
ENSMUSG00000035692	1.60	0.000736068
ENSMUSG00000079071	1.60	0.009404505
ENSMUSG00000050022	1.60	2.48974E-09
ENSMUSG00000018008	1.60	3.03163E-21
ENSMUSG00000029061	1.60	0.002274757
ENSMUSG00000097113	1.60	0.034696068
ENSMUSG00000028874	1.60	1.35692E-14
ENSMUSG00000054200	1.60	0.008336332
ENSMUSG00000043263	1.59	1.59468E-06
ENSMUSG00000079362	1.59	9.73347E-22
ENSMUSG00000024987	1.59	7.10355E-05
ENSMUSG00000060183	1.59	4.59274E-10
ENSMUSG00000071713	1.59	9.94959E-08
ENSMUSG00000037679	1.59	1.32567E-25
ENSMUSG00000028179	1.59	0.012127585
ENSMUSG00000026480	1.59	2.69446E-16
ENSMUSG00000031712	1.59	3.38466E-06
ENSMUSG00000030107	1.59	1.73387E-11
ENSMUSG00000086583	1.59	0.016137337
ENSMUSG00000036905	1.59	2.01125E-27
ENSMUSG00000054342	1.58	0.001584792
ENSMUSG00000040212	1.58	2.07352E-11
ENSMUSG00000042759	1.58	1.42689E-09
ENSMUSG00000059791	1.58	0.006527203
ENSMUSG00000025511	1.58	8.27595E-29
ENSMUSG00000020778	1.58	0.00026039
ENSMUSG00000044345	1.58	2.42925E-25
ENSMUSG00000031506	1.58	2.37658E-05
ENSMUSG00000020838	1.57	0.043547891
ENSMUSG00000022372	1.57	4.74131E-15
ENSMUSG00000038644	1.57	0.038816236
ENSMUSG00000031903	1.57	2.069E-22
ENSMUSG00000044092	1.57	0.000261151
ENSMUSG00000028597	1.57	0.000316888
ENSMUSG00000064023	1.57	0.044976523
ENSMUSG00000078503	1.56	0.046802022
ENSMUSG00000046080	1.56	0.000403998
ENSMUSG00000004446	1.56	0.00246087
ENSMUSG00000050357	1.56	0.003833341
ENSMUSG00000049625	1.56	2.58067E-11

Ensembl gene id	log2FC	p-adjusted
ENSMUSG00000022489	1.56	0.00083104
ENSMUSG00000044328	1.56	3.06985E-05
ENSMUSG00000034783	1.55	0.017822762
ENSMUSG00000026786	1.55	4.221E-13
ENSMUSG00000041954	1.55	0.024314254
ENSMUSG00000051735	1.55	1.46969E-09
ENSMUSG00000075010	1.55	5.97375E-30
ENSMUSG00000041515	1.55	2.31797E-20
ENSMUSG00000006360	1.55	4.75783E-17
ENSMUSG00000070427	1.55	2.42925E-25
ENSMUSG00000026728	1.55	3.92438E-27
ENSMUSG00000004952	1.55	2.73723E-10
ENSMUSG00000015396	1.55	8.02815E-11
ENSMUSG00000034459	1.55	1.63539E-19
ENSMUSG00000001270	1.54	3.81789E-10
ENSMUSG00000057191	1.54	2.45647E-07
ENSMUSG00000029413	1.54	2.6052E-12
ENSMUSG00000059336	1.54	0.014884698
ENSMUSG00000049988	1.54	1.4117E-10
ENSMUSG00000001444	1.53	0.022018234
ENSMUSG00000023349	1.53	5.4162E-10
ENSMUSG00000022474	1.53	2.01227E-05
ENSMUSG00000086763	1.53	0.048007759
ENSMUSG00000033453	1.53	0.000132956
ENSMUSG00000043939	1.53	0.010063809
ENSMUSG00000048779	1.53	5.75607E-11
ENSMUSG00000021728	1.53	1.65726E-05
ENSMUSG00000025165	1.53	0.000419507
ENSMUSG00000006715	1.53	0.003136926
ENSMUSG00000013707	1.52	4.76511E-08
ENSMUSG00000059326	1.52	4.9202E-06
ENSMUSG00000001555	1.52	7.40345E-05
ENSMUSG00000030223	1.52	4.63785E-05
ENSMUSG00000048521	1.52	7.39571E-08
ENSMUSG00000038811	1.52	1.657E-09
ENSMUSG00000000386	1.52	2.15259E-06
ENSMUSG00000036887	1.52	1.18227E-12
ENSMUSG00000008318	1.52	0.000797227
ENSMUSG00000026360	1.51	1.00225E-14
ENSMUSG00000024696	1.51	2.65041E-07
ENSMUSG00000083863	1.51	9.13057E-05
ENSMUSG00000022876	1.51	1.02952E-05
ENSMUSG00000092517	1.51	0.006105182
ENSMUSG00000037972	1.51	1.35933E-09
ENSMUSG00000006310	1.51	0.044182462
ENSMUSG00000027454	1.51	0.010427279
ENSMUSG00000073409	1.51	4.16467E-07
ENSMUSG00000045625	1.51	0.014465533
ENSMUSG00000027347	1.50	7.48992E-10
ENSMUSG00000031756	1.50	0.014967039
ENSMUSG00000033355	1.50	3.03163E-21
ENSMUSG00000068335	1.50	9.50318E-08

Ensembl gene id	log2FC	p-adjusted
ENSMUSG00000024677	1.50	1.15384E-11
ENSMUSG00000031722	1.50	8.40565E-09
ENSMUSG00000001281	1.50	4.96149E-13
ENSMUSG00000029254	1.50	4.65844E-06
ENSMUSG00000069793	1.50	0.000176321
ENSMUSG00000004698	1.50	0.008277596
ENSMUSG00000036006	1.50	7.33567E-07
ENSMUSG00000079429	1.50	0.018713338
ENSMUSG00000024013	1.50	7.68799E-14
ENSMUSG00000063458	1.49	0.022754478
ENSMUSG00000030124	1.49	0.034696068
ENSMUSG00000006219	1.49	7.7979E-09
ENSMUSG00000026972	1.49	1.01935E-08
ENSMUSG00000074151	1.49	8.3272E-09
ENSMUSG00000056145	1.49	0.002872883
ENSMUSG00000000244	1.49	0.001016777
ENSMUSG00000036896	1.49	7.21828E-13
ENSMUSG00000028843	1.49	7.46127E-14
ENSMUSG00000046688	1.49	2.53423E-13
ENSMUSG00000021280	1.49	0.002570472
ENSMUSG00000039109	1.49	0.001939046
ENSMUSG00000020388	1.49	5.08938E-08
ENSMUSG00000020623	1.49	0.002926277
ENSMUSG00000044703	1.49	0.016627272
ENSMUSG00000083817	1.49	0.025642389
ENSMUSG00000026832	1.49	7.59462E-08
ENSMUSG00000074183	1.48	0.016736397
ENSMUSG00000040345	1.48	1.78805E-10
ENSMUSG00000028931	1.48	1.29918E-06
ENSMUSG00000031778	1.48	0.001207414
ENSMUSG00000024011	1.48	0.002270106
ENSMUSG00000052087	1.48	0.001554561
ENSMUSG00000079491	1.48	0.012402025
ENSMUSG00000058715	1.48	1.23615E-18
ENSMUSG00000079227	1.48	2.64795E-08
ENSMUSG00000066363	1.47	1.07997E-23
ENSMUSG00000018648	1.47	0.012362255
ENSMUSG00000031880	1.47	0.00893201
ENSMUSG00000030187	1.47	6.01422E-05
ENSMUSG00000008193	1.47	5.72267E-06
ENSMUSG00000037572	1.47	0.002094212
ENSMUSG00000037860	1.47	1.74573E-06
ENSMUSG00000066456	1.47	0.010081454
ENSMUSG00000028645	1.47	5.4723E-14
ENSMUSG00000055866	1.47	6.5934E-08
ENSMUSG00000002699	1.47	1.19943E-10
ENSMUSG00000010830	1.47	0.001867451
ENSMUSG00000017969	1.47	0.002228282
ENSMUSG00000036931	1.46	0.006192936
ENSMUSG00000036390	1.46	0.000171984
ENSMUSG00000030528	1.46	0.015542966
ENSMUSG00000020120	1.46	7.52539E-16

Ensembl gene id	log2FC	p-adjusted
ENSMUSG00000028637	1.46	0.023997769
ENSMUSG00000038352	1.46	0.001097447
ENSMUSG00000020395	1.46	1.5911E-05
ENSMUSG00000078612	1.46	6.73744E-07
ENSMUSG00000034023	1.46	0.017266792
ENSMUSG00000034959	1.45	0.034060928
ENSMUSG00000047810	1.45	4.22448E-11
ENSMUSG00000003153	1.45	0.000120245
ENSMUSG00000034317	1.45	0.008819562
ENSMUSG00000057346	1.45	0.004856725
ENSMUSG00000040747	1.45	1.46199E-13
ENSMUSG00000028480	1.45	8.9586E-09
ENSMUSG00000028832	1.45	0.019261903
ENSMUSG00000021965	1.45	0.018566776
ENSMUSG00000060989	1.44	0.022838918
ENSMUSG00000022439	1.44	1.86703E-11
ENSMUSG00000096472	1.44	1.96827E-05
ENSMUSG00000078502	1.44	0.037167019
ENSMUSG00000058818	1.43	4.52393E-14
ENSMUSG00000030089	1.43	7.31859E-05
ENSMUSG00000039601	1.43	0.015527007
ENSMUSG00000048445	1.43	7.36684E-06
ENSMUSG00000046805	1.43	2.94514E-23
ENSMUSG00000036894	1.43	4.95082E-08
ENSMUSG00000050014	1.43	0.004910144
ENSMUSG00000028581	1.43	2.85313E-22
ENSMUSG00000044783	1.43	0.003337846
ENSMUSG00000050232	1.42	0.018230054
ENSMUSG00000022346	1.42	5.25821E-09
ENSMUSG00000034792	1.42	0.003390219
ENSMUSG00000031765	1.42	1.06616E-05
ENSMUSG00000023755	1.42	0.009100823
ENSMUSG00000068758	1.42	8.61697E-06
ENSMUSG00000027639	1.42	2.75132E-23
ENSMUSG00000042379	1.42	0.008685971
ENSMUSG00000029484	1.41	2.38877E-11
ENSMUSG00000020695	1.41	2.84515E-05
ENSMUSG00000021886	1.41	0.000189425
ENSMUSG00000079419	1.41	1.11423E-14
ENSMUSG00000018507	1.41	4.3206E-08
ENSMUSG00000076928	1.41	0.001566028
ENSMUSG00000024965	1.41	2.2771E-15
ENSMUSG00000022947	1.41	0.01003497
ENSMUSG00000062524	1.41	0.008638756
ENSMUSG00000056054	1.41	0.000187779
ENSMUSG00000005360	1.41	0.005712593
ENSMUSG00000078897	1.41	0.025667253
ENSMUSG00000052160	1.40	4.11067E-19
ENSMUSG00000035232	1.40	0.002755201
ENSMUSG00000032436	1.40	1.06005E-09
ENSMUSG00000066861	1.40	3.09201E-05
ENSMUSG00000043017	1.40	0.030982011

Ensembl gene id	log2FC	p-adjusted
ENSMUSG00000017167	1.40	7.42732E-05
ENSMUSG00000022221	1.40	1.05931E-07
ENSMUSG00000024737	1.40	3.04155E-08
ENSMUSG00000017002	1.39	0.011384046
ENSMUSG00000022432	1.39	0.02063852
ENSMUSG00000087107	1.39	1.05394E-09
ENSMUSG00000074874	1.39	9.50553E-09
ENSMUSG00000044250	1.39	1.11961E-05
ENSMUSG00000003051	1.39	5.33655E-05
ENSMUSG00000038034	1.39	2.45126E-13
ENSMUSG00000025888	1.39	5.72542E-10
ENSMUSG00000041420	1.38	0.000162248
ENSMUSG00000031897	1.38	1.24136E-09
ENSMUSG00000082433	1.38	0.007520848
ENSMUSG00000039936	1.38	1.38131E-14
ENSMUSG00000055546	1.38	6.45383E-19
ENSMUSG00000034614	1.38	0.015052862
ENSMUSG00000030188	1.38	0.000145464
ENSMUSG00000068246	1.38	0.009863558
ENSMUSG00000040483	1.38	3.77963E-13
ENSMUSG00000021569	1.38	0.030655017
ENSMUSG00000056481	1.38	8.00139E-05
ENSMUSG00000033685	1.38	3.53938E-22
ENSMUSG00000041827	1.38	3.51256E-07
ENSMUSG00000028071	1.38	0.005002794
ENSMUSG00000018920	1.37	7.8171E-16
ENSMUSG00000028150	1.37	7.69589E-06
ENSMUSG00000045322	1.37	0.003776437
ENSMUSG00000010307	1.37	2.18901E-15
ENSMUSG00000031494	1.37	0.017865176
ENSMUSG00000030844	1.37	6.52555E-07
ENSMUSG00000046879	1.37	6.86463E-24
ENSMUSG00000037902	1.37	7.3531E-22
ENSMUSG00000027750	1.37	7.07885E-15
ENSMUSG00000030587	1.36	0.001528978
ENSMUSG00000025498	1.36	2.91829E-13
ENSMUSG00000060044	1.36	2.48431E-09
ENSMUSG00000087413	1.36	0.014582362
ENSMUSG00000026358	1.36	1.69241E-05
ENSMUSG00000024349	1.36	3.17075E-10
ENSMUSG00000034593	1.36	4.82028E-05
ENSMUSG00000030830	1.36	1.60396E-12
ENSMUSG00000034255	1.36	1.34954E-07
ENSMUSG00000041219	1.35	0.010894586
ENSMUSG00000048163	1.35	3.43829E-12
ENSMUSG00000032577	1.35	3.19322E-05
ENSMUSG00000040253	1.35	9.65177E-06
ENSMUSG00000033192	1.35	1.16691E-06
ENSMUSG00000069516	1.35	8.80414E-23
ENSMUSG00000085455	1.35	7.92417E-06
ENSMUSG00000029304	1.35	0.004000618
ENSMUSG00000032661	1.35	4.35852E-05

Ensembl gene id	log2FC	p-adjusted
ENSMUSG00000057068	1.35	9.10329E-16
ENSMUSG00000007080	1.35	0.00503848
ENSMUSG00000051439	1.35	1.27182E-05
ENSMUSG00000008153	1.34	0.002234723
ENSMUSG00000056071	1.34	2.67763E-06
ENSMUSG00000034413	1.34	0.027232691
ENSMUSG00000052248	1.34	0.006685237
ENSMUSG00000029570	1.34	3.6548E-10
ENSMUSG00000018899	1.34	1.52447E-12
ENSMUSG00000038370	1.34	0.036258538
ENSMUSG00000018654	1.34	1.62286E-11
ENSMUSG00000012519	1.34	4.00739E-12
ENSMUSG00000047878	1.33	0.034198607
ENSMUSG00000090213	1.33	1.42034E-09
ENSMUSG00000074476	1.33	0.03076753
ENSMUSG00000015854	1.32	4.11119E-22
ENSMUSG00000070803	1.32	0.020958702
ENSMUSG00000023828	1.32	1.86337E-07
ENSMUSG00000085175	1.32	0.013933578
ENSMUSG00000038943	1.32	0.023151393
ENSMUSG00000019876	1.32	9.38451E-06
ENSMUSG00000016206	1.32	8.22872E-09
ENSMUSG00000029561	1.32	1.79739E-10
ENSMUSG00000030047	1.32	3.59088E-09
ENSMUSG00000021640	1.32	0.028982257
ENSMUSG00000039304	1.32	7.90296E-07
ENSMUSG00000034652	1.32	4.3474E-09
ENSMUSG00000053113	1.32	0.007966969
ENSMUSG00000030218	1.31	1.22096E-09
ENSMUSG00000045165	1.31	0.00068407
ENSMUSG00000000489	1.31	8.09985E-05
ENSMUSG00000027611	1.31	9.56247E-06
ENSMUSG00000062210	1.31	2.1214E-08
ENSMUSG00000038807	1.31	0.000141296
ENSMUSG00000022488	1.31	1.26699E-05
ENSMUSG00000030774	1.31	7.04008E-05
ENSMUSG00000029581	1.31	3.43687E-10
ENSMUSG00000002458	1.31	5.87872E-08
ENSMUSG00000022831	1.31	5.12579E-14
ENSMUSG00000033538	1.31	1.03807E-05
ENSMUSG00000072844	1.31	0.042694917
ENSMUSG00000027848	1.31	8.87983E-08
ENSMUSG00000020788	1.30	8.95042E-09
ENSMUSG00000032294	1.30	3.14499E-17
ENSMUSG00000001417	1.30	6.47563E-09
ENSMUSG00000022534	1.30	0.000725908
ENSMUSG00000091898	1.30	0.006925436
ENSMUSG00000037997	1.30	6.71217E-12
ENSMUSG00000090164	1.30	0.00077967
ENSMUSG00000028789	1.30	0.004656189
ENSMUSG00000056220	1.30	8.33669E-08
ENSMUSG00000041801	1.30	0.001992311

Ensembl gene id	log2FC	p-adjusted
ENSMUSG00000060063	1.29	0.000152405
ENSMUSG00000038060	1.29	0.031138995
ENSMUSG00000047497	1.29	0.034584127
ENSMUSG00000042029	1.29	0.006686008
ENSMUSG00000031103	1.29	3.53619E-07
ENSMUSG00000084274	1.29	0.016275948
ENSMUSG00000096768	1.29	0.024883690
ENSMUSG00000005947	1.29	0.025878469
ENSMUSG00000029591	1.29	6.47425E-08
ENSMUSG00000052776	1.29	6.29951E-09
ENSMUSG00000052384	1.28	2.73472E-13
ENSMUSG00000037594	1.28	0.009385129
ENSMUSG00000025232	1.28	1.71888E-18
ENSMUSG00000039886	1.28	3.05011E-18
ENSMUSG00000039457	1.28	0.000249207
ENSMUSG00000020774	1.28	8.09167E-13
ENSMUSG00000049744	1.28	0.001747785
ENSMUSG00000051146	1.28	0.016624962
ENSMUSG00000037280	1.28	4.42734E-05
ENSMUSG00000042817	1.28	0.019540017
ENSMUSG00000011008	1.28	0.025394438
ENSMUSG00000027843	1.28	4.01461E-05
ENSMUSG00000019874	1.28	1.31078E-11
ENSMUSG00000031904	1.27	0.025662922
ENSMUSG00000040552	1.27	1.42081E-08
ENSMUSG00000036526	1.27	0.001307761
ENSMUSG00000040990	1.27	4.69978E-08
ENSMUSG00000074269	1.27	0.007209432
ENSMUSG00000006411	1.27	0.045433907
ENSMUSG00000037847	1.27	0.000202829
ENSMUSG00000033970	1.27	5.10112E-05
ENSMUSG00000018459	1.27	1.78997E-18
ENSMUSG00000011884	1.27	2.88162E-11
ENSMUSG00000032374	1.27	0.001418026
ENSMUSG00000097194	1.27	5.62125E-05
ENSMUSG00000055612	1.26	0.019298656
ENSMUSG00000049307	1.26	0.041623667
ENSMUSG00000060791	1.26	3.89502E-07
ENSMUSG00000053310	1.26	0.025967606
ENSMUSG00000095098	1.26	2.79789E-07
ENSMUSG00000040061	1.26	7.87211E-07
ENSMUSG00000035208	1.26	1.10665E-06
ENSMUSG00000029521	1.26	0.002437497
ENSMUSG00000047180	1.26	0.00012172
ENSMUSG00000062012	1.26	0.001869913
ENSMUSG00000030263	1.25	0.004297717
ENSMUSG00000031004	1.25	0.006235946
ENSMUSG00000046417	1.25	4.00128E-05
ENSMUSG00000043439	1.25	0.038832677
ENSMUSG00000030208	1.25	2.93735E-07
ENSMUSG00000048612	1.25	1.34984E-07
ENSMUSG00000075611	1.24	0.065163323

Ensembl gene id	log2FC	p-adjusted
ENSMUSG00000047517	1.24	0.000423936
ENSMUSG00000021451	1.24	5.7474E-06
ENSMUSG00000030244	1.24	0.004081573
ENSMUSG00000010021	1.24	0.005385965
ENSMUSG00000026979	1.24	1.73575E-06
ENSMUSG00000040528	1.24	0.00656803
ENSMUSG00000037138	1.24	0.040270735
ENSMUSG00000029298	1.24	0.017597971
ENSMUSG00000019737	1.24	0.028099243
ENSMUSG00000026547	1.24	1.14786E-15
ENSMUSG00000025602	1.24	0.004283126
ENSMUSG00000097296	1.24	0.00553891
ENSMUSG00000000628	1.24	0.000222718
ENSMUSG00000067608	1.24	0.006901824
ENSMUSG00000009585	1.24	8.15851E-08
ENSMUSG00000031165	1.24	8.56127E-07
ENSMUSG00000054072	1.24	9.74639E-11
ENSMUSG00000027955	1.23	0.001226972
ENSMUSG00000002983	1.23	6.25376E-06
ENSMUSG00000056888	1.23	0.006137206
ENSMUSG00000021932	1.23	8.05201E-08
ENSMUSG00000000732	1.23	0.00033883
ENSMUSG00000010660	1.23	0.041106794
ENSMUSG00000023274	1.23	5.49217E-06
ENSMUSG00000007041	1.23	6.50916E-16
ENSMUSG00000029553	1.23	9.725E-08
ENSMUSG00000024810	1.23	1.30329E-06
ENSMUSG00000001750	1.22	5.25192E-17
ENSMUSG00000086432	1.22	2.4904E-05
ENSMUSG00000020649	1.22	0.035286715
ENSMUSG00000025395	1.22	0.003669413
ENSMUSG00000039994	1.22	0.004276931
ENSMUSG00000026395	1.22	7.90213E-16
ENSMUSG00000039632	1.22	0.049600896
ENSMUSG00000049685	1.22	0.002613565
ENSMUSG00000059498	1.22	2.17853E-12
ENSMUSG00000026548	1.22	0.003634736
ENSMUSG00000078899	1.22	0.009305286
ENSMUSG00000026858	1.21	4.89651E-05
ENSMUSG00000020901	1.21	2.9986E-06
ENSMUSG00000033467	1.21	2.66798E-05
ENSMUSG00000042622	1.21	0.000221458
ENSMUSG00000099065	1.21	0.010312951
ENSMUSG00000037419	1.21	2.01311E-08
ENSMUSG00000090523	1.21	3.83831E-06
ENSMUSG00000026177	1.21	1.01294E-06
ENSMUSG00000037820	1.21	6.2224E-06
ENSMUSG00000042485	1.21	0.000135195
ENSMUSG00000034837	1.21	3.32934E-07
ENSMUSG00000037816	1.21	0.000468136
ENSMUSG00000073902	1.21	0.006747685
ENSMUSG00000033777	1.21	3.15483E-09

Ensembl gene id	log2FC	p-adjusted
ENSMUSG00000011256	1.20	2.8788E-06
ENSMUSG00000079442	1.20	0.018304946
ENSMUSG00000069515	1.20	0.004514003
ENSMUSG00000046245	1.20	2.66863E-05
ENSMUSG00000074141	1.20	0.000246387
ENSMUSG00000044199	1.20	0.01649083
ENSMUSG00000015843	1.20	3.99509E-08
ENSMUSG00000024795	1.20	0.013542506
ENSMUSG00000000440	1.20	0.000242789
ENSMUSG00000014453	1.19	0.037123494
ENSMUSG00000087150	1.19	0.003943222
ENSMUSG00000038540	1.19	0.03303066
ENSMUSG00000036469	1.19	7.13295E-08
ENSMUSG00000015340	1.19	2.50044E-09
ENSMUSG00000038151	1.19	0.00638163
ENSMUSG00000037217	1.19	0.00704293
ENSMUSG00000047246	1.19	0.01706639
ENSMUSG00000032231	1.19	1.5432E-14
ENSMUSG00000042834	1.19	5.19345E-05
ENSMUSG00000044708	1.19	7.09578E-06
ENSMUSG00000049866	1.19	1.32851E-08
ENSMUSG00000031934	1.19	0.00204855
ENSMUSG00000036181	1.19	0.028023291
ENSMUSG00000036381	1.18	1.56896E-05
ENSMUSG00000023505	1.18	0.031247084
ENSMUSG00000060675	1.18	5.64849E-13
ENSMUSG00000029552	1.18	0.002399553
ENSMUSG00000019866	1.18	1.19709E-05
ENSMUSG00000001918	1.18	5.33655E-05
ENSMUSG00000030651	1.18	0.007820403
ENSMUSG00000001473	1.18	0.000407124
ENSMUSG00000028933	1.17	0.012141559
ENSMUSG00000024590	1.17	0.000998854
ENSMUSG00000029101	1.17	1.18845E-07
ENSMUSG00000034330	1.17	8.5544E-08
ENSMUSG00000026288	1.17	8.21941E-11
ENSMUSG00000023078	1.17	0.010677846
ENSMUSG00000034591	1.17	6.02064E-14
ENSMUSG00000037318	1.17	0.000520226
ENSMUSG00000019139	1.17	6.60783E-08
ENSMUSG00000021508	1.17	0.004181269
ENSMUSG00000071379	1.17	3.10591E-07
ENSMUSG00000045659	1.17	1.36225E-06
ENSMUSG00000043004	1.17	4.51877E-07
ENSMUSG00000061578	1.17	0.04213995
ENSMUSG00000043157	1.17	0.005715309
ENSMUSG00000024339	1.17	1.00016E-14
ENSMUSG00000030159	1.16	1.97895E-10
ENSMUSG00000020407	1.16	0.00123335
ENSMUSG00000061825	1.16	0.017235251
ENSMUSG00000056529	1.16	0.000886555
ENSMUSG00000032334	1.16	0.000132333

Ensembl gene id	log2FC	p-adjusted
ENSMUSG00000024538	1.16	7.25273E-07
ENSMUSG00000002602	1.16	3.75621E-08
ENSMUSG00000021457	1.16	4.98498E-12
ENSMUSG00000027219	1.16	0.034959281
ENSMUSG00000068227	1.16	2.91294E-06
ENSMUSG00000027134	1.16	0.014344576
ENSMUSG00000021250	1.16	0.017469976
ENSMUSG00000091705	1.15	0.014179002
ENSMUSG00000045404	1.15	0.005265809
ENSMUSG00000048865	1.15	6.21061E-07
ENSMUSG00000029925	1.15	0.001896116
ENSMUSG00000055675	1.15	0.000548277
ENSMUSG00000015745	1.15	7.73187E-10
ENSMUSG00000025758	1.15	0.018845441
ENSMUSG00000021773	1.15	0.038940869
ENSMUSG00000064326	1.15	0.000487406
ENSMUSG00000024621	1.14	9.10514E-17
ENSMUSG00000031389	1.14	6.67441E-09
ENSMUSG00000018339	1.14	0.018764431
ENSMUSG00000080845	1.14	0.016465086
ENSMUSG00000001128	1.14	5.90372E-15
ENSMUSG00000022180	1.14	2.3173E-10
ENSMUSG00000031618	1.14	1.05931E-07
ENSMUSG00000016495	1.14	1.26969E-08
ENSMUSG00000050721	1.14	6.85702E-12
ENSMUSG00000034595	1.14	4.43564E-10
ENSMUSG00000039208	1.14	0.000204566
ENSMUSG00000074661	1.13	0.002685246
ENSMUSG00000020143	1.13	1.2543E-07
ENSMUSG00000022978	1.13	0.000167564
ENSMUSG00000081769	1.13	0.04132996
ENSMUSG00000057789	1.13	9.89024E-11
ENSMUSG00000023032	1.13	0.03492954
ENSMUSG00000038252	1.12	0.005894223
ENSMUSG00000040711	1.12	1.18731E-06
ENSMUSG00000040710	1.12	0.001378521
ENSMUSG00000021811	1.12	0.003910436
ENSMUSG00000022090	1.12	0.004128206
ENSMUSG00000025007	1.12	0.000202847
ENSMUSG00000032254	1.12	0.013430135
ENSMUSG00000050069	1.12	0.016029266
ENSMUSG00000027078	1.12	0.02072086
ENSMUSG00000022378	1.12	3.77692E-11
ENSMUSG00000045382	1.12	0.001644994
ENSMUSG00000026616	1.12	0.023351545
ENSMUSG00000024030	1.12	1.31228E-06
ENSMUSG00000032089	1.12	1.93468E-09
ENSMUSG00000035356	1.12	0.032286022
ENSMUSG00000042436	1.11	2.94461E-05
ENSMUSG00000028860	1.11	0.026495188
ENSMUSG00000026259	1.11	4.12453E-13
ENSMUSG00000031697	1.11	0.023413933

Ensembl gene id	log2FC	p-adjusted
ENSMUSG00000047712	1.11	0.005543036
ENSMUSG00000045551	1.11	7.57952E-05
ENSMUSG00000024660	1.11	0.01072622
ENSMUSG00000029326	1.11	1.28752E-05
ENSMUSG00000042684	1.11	9.65635E-07
ENSMUSG00000047250	1.11	5.42194E-12
ENSMUSG00000020330	1.11	0.03328507
ENSMUSG00000048251	1.11	0.020831095
ENSMUSG00000032085	1.10	7.56545E-10
ENSMUSG00000056665	1.10	0.006263071
ENSMUSG00000040471	1.10	0.007117995
ENSMUSG00000024521	1.10	0.001348564
ENSMUSG00000041488	1.10	9.11176E-06
ENSMUSG00000038843	1.10	0.002198929
ENSMUSG00000019850	1.10	9.54721E-07
ENSMUSG00000025077	1.10	1.21467E-09
ENSMUSG00000070501	1.10	0.015643009
ENSMUSG00000078867	1.10	0.013851862
ENSMUSG00000028551	1.10	0.010894586
ENSMUSG00000039633	1.10	4.90908E-07
ENSMUSG00000025875	1.10	0.00920437
ENSMUSG00000040447	1.09	2.31946E-09
ENSMUSG00000031827	1.09	3.63797E-13
ENSMUSG00000001995	1.09	5.04156E-05
ENSMUSG00000052889	1.09	1.47673E-05
ENSMUSG00000020895	1.09	0.015719325
ENSMUSG00000030895	1.09	2.72866E-15
ENSMUSG00000019823	1.09	0.0004967
ENSMUSG00000022014	1.09	1.22101E-05
ENSMUSG00000034785	1.09	0.002263539
ENSMUSG00000098112	1.09	1.05454E-07
ENSMUSG00000022436	1.08	1.38106E-05
ENSMUSG00000001588	1.08	6.2965E-06
ENSMUSG00000060550	1.08	0.002273762
ENSMUSG00000053158	1.08	4.26308E-08
ENSMUSG00000040204	1.08	0.01803221
ENSMUSG00000054626	1.08	0.016902819
ENSMUSG00000031504	1.08	1.19131E-05
ENSMUSG00000021998	1.08	3.49749E-12
ENSMUSG00000025154	1.08	8.27834E-08
ENSMUSG00000081058	1.08	0.003156466
ENSMUSG00000064065	1.07	0.030342237
ENSMUSG00000029366	1.07	0.000365267
ENSMUSG00000032184	1.07	0.006755528
ENSMUSG00000031805	1.07	1.91696E-09
ENSMUSG00000032470	1.07	0.000138557
ENSMUSG00000034765	1.07	0.003973796
ENSMUSG00000036908	1.07	4.06116E-13
ENSMUSG00000037035	1.07	0.000189014
ENSMUSG00000023886	1.07	0.003676616
ENSMUSG00000036362	1.07	2.57478E-07
ENSMUSG00000041147	1.07	0.002668782

Ensembl gene id	log2FC	p-adjusted
ENSMUSG00000039157	1.07	7.09892E-13
ENSMUSG00000035914	1.07	0.005091367
ENSMUSG00000023947	1.07	1.65747E-05
ENSMUSG00000022181	1.06	1.12124E-09
ENSMUSG00000040663	1.06	0.011225509
ENSMUSG00000055805	1.06	1.28182E-06
ENSMUSG00000026628	1.06	4.43548E-08
ENSMUSG00000020886	1.06	0.005358009
ENSMUSG00000025856	1.06	0.000403998
ENSMUSG00000040829	1.06	8.00222E-06
ENSMUSG00000028059	1.06	3.92587E-05
ENSMUSG00000063268	1.06	1.36328E-11
ENSMUSG00000027318	1.06	0.012202189
ENSMUSG00000036672	1.06	0.00319783
ENSMUSG00000074165	1.06	0.000117024
ENSMUSG00000048218	1.05	0.03533059
ENSMUSG00000070003	1.05	1.6841E-05
ENSMUSG00000025422	1.05	0.00061881
ENSMUSG00000024909	1.05	3.63456E-06
ENSMUSG00000026821	1.05	2.58022E-05
ENSMUSG00000026110	1.05	0.000170264
ENSMUSG00000028859	1.05	6.36524E-05
ENSMUSG00000025402	1.05	4.75369E-05
ENSMUSG00000097187	1.05	0.036090095
ENSMUSG00000039232	1.05	0.01690829
ENSMUSG00000045917	1.05	5.72542E-10
ENSMUSG00000022148	1.05	4.09654E-05
ENSMUSG00000005514	1.05	0.001443351
ENSMUSG00000021196	1.05	2.27069E-06
ENSMUSG00000023913	1.05	9.89244E-05
ENSMUSG00000027293	1.04	4.55966E-08
ENSMUSG00000031253	1.04	0.009516337
ENSMUSG00000037946	1.04	0.000199439
ENSMUSG00000057836	1.04	0.018030806
ENSMUSG00000004730	1.04	4.87429E-06
ENSMUSG00000022856	1.04	1.98951E-06
ENSMUSG00000071203	1.04	0.00018443
ENSMUSG00000022965	1.04	2.57834E-10
ENSMUSG00000027353	1.04	0.00294836
ENSMUSG00000018381	1.04	0.001612167
ENSMUSG00000023827	1.04	0.029628977
ENSMUSG00000006589	1.04	2.20622E-05
ENSMUSG00000061322	1.04	6.38073E-06
ENSMUSG00000073411	1.04	0.008564379
ENSMUSG00000026956	1.03	7.81264E-06
ENSMUSG00000025732	1.03	3.82157E-06
ENSMUSG00000057948	1.03	0.000686286
ENSMUSG00000032643	1.03	0.043637512
ENSMUSG00000098557	1.03	3.01927E-05
ENSMUSG00000050592	1.03	0.000161091
ENSMUSG00000093661	1.03	6.796E-08
ENSMUSG00000064267	1.03	6.77905E-05

Ensembl gene id	log2FC	p-adjusted
ENSMUSG00000040312	1.03	0.000743922
ENSMUSG00000032725	1.02	5.29579E-10
ENSMUSG00000018168	1.02	0.007866361
ENSMUSG00000031659	1.02	1.54539E-07
ENSMUSG00000024486	1.02	0.008222533
ENSMUSG00000014599	1.02	6.63868E-11
ENSMUSG00000024824	1.02	0.000309734
ENSMUSG00000026456	1.02	1.1459E-05
ENSMUSG00000009292	1.02	7.49555E-05
ENSMUSG00000047945	1.02	1.09828E-08
ENSMUSG00000005958	1.02	0.018936817
ENSMUSG00000048498	1.02	0.001840421
ENSMUSG00000023277	1.02	1.72095E-05
ENSMUSG00000024524	1.02	6.32017E-05
ENSMUSG00000042638	1.02	0.000971249
ENSMUSG00000042842	1.02	0.00288636
ENSMUSG00000044206	1.01	1.53565E-12
ENSMUSG00000034192	1.01	9.33378E-05
ENSMUSG00000030282	1.01	8.83532E-12
ENSMUSG00000044702	1.01	0.027818978
ENSMUSG00000004266	1.01	1.08363E-10
ENSMUSG00000037405	1.01	1.18703E-09
ENSMUSG00000020810	1.01	0.001822351
ENSMUSG00000020424	1.01	0.040685371
ENSMUSG00000040616	1.01	9.2689E-06
ENSMUSG00000029449	1.00	0.038533159
ENSMUSG00000022369	1.00	0.005306771
ENSMUSG00000002233	1.00	4.05819E-05
ENSMUSG00000037613	1.00	0.040138808
ENSMUSG00000022952	1.00	0.004798798
ENSMUSG00000048497	1.00	0.001122192
ENSMUSG00000037020	1.00	0.011133327
ENSMUSG00000073982	1.00	4.64479E-09
ENSMUSG00000015568	1.00	1.86703E-11
ENSMUSG00000029007	1.00	4.13853E-06
ENSMUSG00000038543	0.99	0.005152936
ENSMUSG00000031478	0.93	0.028363045
ENSMUSG00000020131	0.92	0.032182271
ENSMUSG00000032782	0.92	4.88058E-05
ENSMUSG00000090019	0.91	0.005142832
ENSMUSG00000031093	0.90	0.000314771
ENSMUSG00000036040	0.89	0.029269277
ENSMUSG00000042784	0.85	0.008966972
ENSMUSG00000053334	-1.00	1.85708E-07
ENSMUSG00000033295	-1.00	1.03771E-13
ENSMUSG00000068959	-1.00	0.002757727
ENSMUSG00000002028	-1.00	1.85315E-07
ENSMUSG00000011831	-1.00	3.51331E-13
ENSMUSG00000041471	-1.00	7.63424E-12
ENSMUSG00000043418	-1.00	0.002800477
ENSMUSG00000024241	-1.00	8.49866E-12
ENSMUSG00000070690	-1.01	2.81458E-06

Ensembl gene id	log2FC	p-adjusted
ENSMUSG00000032902	-1.01	0.003304734
ENSMUSG00000029016	-1.01	4.10503E-05
ENSMUSG00000031889	-1.01	7.25638E-11
ENSMUSG00000055320	-1.01	2.21872E-08
ENSMUSG00000020634	-1.01	3.53999E-07
ENSMUSG00000015305	-1.02	2.70749E-12
ENSMUSG00000045095	-1.02	9.76483E-11
ENSMUSG00000059495	-1.02	7.68799E-14
ENSMUSG00000034297	-1.02	3.09485E-12
ENSMUSG00000024039	-1.02	2.36393E-13
ENSMUSG00000029571	-1.02	4.89009E-14
ENSMUSG00000023019	-1.02	8.71906E-09
ENSMUSG00000040128	-1.03	4.18587E-13
ENSMUSG00000046138	-1.03	1.4541E-10
ENSMUSG00000041293	-1.03	0.032363731
ENSMUSG00000096463	-1.03	0.03137952
ENSMUSG00000059149	-1.03	2.69E-09
ENSMUSG00000032599	-1.03	5.15534E-09
ENSMUSG00000039004	-1.03	0.003676616
ENSMUSG00000032119	-1.03	1.50857E-05
ENSMUSG00000039831	-1.03	1.37094E-09
ENSMUSG00000021638	-1.03	4.44228E-10
ENSMUSG00000022141	-1.03	1.07276E-12
ENSMUSG00000025504	-1.03	3.19158E-07
ENSMUSG00000020038	-1.04	1.4117E-10
ENSMUSG00000066441	-1.04	0.034995512
ENSMUSG00000028864	-1.04	3.64686E-08
ENSMUSG00000066154	-1.04	0.000108873
ENSMUSG00000063663	-1.04	5.12351E-09
ENSMUSG00000085328	-1.04	1.48676E-05
ENSMUSG00000073664	-1.04	1.79774E-12
ENSMUSG00000033943	-1.04	1.83488E-10
ENSMUSG00000032525	-1.05	8.3272E-09
ENSMUSG00000005373	-1.05	5.2427E-05
ENSMUSG00000051910	-1.05	7.98485E-08
ENSMUSG00000030102	-1.05	2.31016E-08
ENSMUSG00000037416	-1.05	8.26991E-11
ENSMUSG00000042659	-1.05	4.33683E-12
ENSMUSG00000055485	-1.05	4.10503E-05
ENSMUSG00000022241	-1.05	1.68527E-14
ENSMUSG00000040536	-1.06	2.84164E-06
ENSMUSG00000048154	-1.06	3.78586E-12
ENSMUSG00000016756	-1.06	1.5919E-14
ENSMUSG00000050730	-1.06	1.55697E-07
ENSMUSG00000044749	-1.06	4.95266E-13
ENSMUSG00000040524	-1.06	6.32153E-12
ENSMUSG00000018501	-1.06	7.57676E-15
ENSMUSG00000026502	-1.06	5.04878E-13
ENSMUSG00000050310	-1.06	2.50683E-11
ENSMUSG00000060012	-1.06	1.32953E-11
ENSMUSG00000035948	-1.06	3.26115E-09
ENSMUSG00000033022	-1.06	7.3082E-16

Ensembl gene id	log2FC	p-adjusted
ENSMUSG00000025937	-1.06	5.27181E-15
ENSMUSG00000033382	-1.06	8.18873E-14
ENSMUSG00000019889	-1.06	8.25053E-14
ENSMUSG00000071633	-1.06	0.024977262
ENSMUSG00000024807	-1.07	0.002869073
ENSMUSG00000018166	-1.07	6.00144E-13
ENSMUSG00000028327	-1.07	2.26469E-15
ENSMUSG00000053617	-1.07	0.00725948
ENSMUSG00000040225	-1.07	6.39073E-13
ENSMUSG00000026610	-1.07	0.005017313
ENSMUSG00000022772	-1.07	9.05407E-10
ENSMUSG00000022347	-1.08	0.000326094
ENSMUSG00000035877	-1.08	1.5919E-14
ENSMUSG00000047036	-1.08	0.000622673
ENSMUSG00000035349	-1.08	2.36737E-10
ENSMUSG00000002222	-1.08	8.01727E-16
ENSMUSG00000063550	-1.08	3.15497E-09
ENSMUSG00000015755	-1.08	1.08716E-14
ENSMUSG00000028842	-1.08	4.24575E-09
ENSMUSG00000039809	-1.09	0.002793041
ENSMUSG00000032349	-1.09	1.55441E-16
ENSMUSG00000097434	-1.09	0.043810452
ENSMUSG00000015890	-1.09	7.15711E-16
ENSMUSG00000064254	-1.09	5.73527E-05
ENSMUSG00000042496	-1.09	6.14169E-05
ENSMUSG00000032607	-1.09	1.55845E-12
ENSMUSG00000056749	-1.09	2.25661E-11
ENSMUSG00000037234	-1.09	3.49325E-15
ENSMUSG00000026692	-1.09	2.87662E-05
ENSMUSG00000096979	-1.10	0.03638324
ENSMUSG00000033306	-1.10	2.13847E-15
ENSMUSG00000033416	-1.10	6.03598E-08
ENSMUSG00000020021	-1.10	2.18901E-15
ENSMUSG00000030655	-1.10	7.81778E-15
ENSMUSG00000022383	-1.10	6.25672E-10
ENSMUSG00000043019	-1.11	8.93584E-15
ENSMUSG00000026638	-1.11	6.75108E-15
ENSMUSG00000024130	-1.11	8.19565E-17
ENSMUSG00000021514	-1.11	1.9337E-08
ENSMUSG00000055480	-1.11	0.01389043
ENSMUSG00000097657	-1.11	0.024520798
ENSMUSG00000028630	-1.12	0.001288424
ENSMUSG00000003178	-1.12	1.70675E-07
ENSMUSG00000030315	-1.12	8.32457E-14
ENSMUSG00000063077	-1.12	9.10514E-17
ENSMUSG00000028053	-1.12	5.30854E-15
ENSMUSG00000079550	-1.13	0.028070965
ENSMUSG00000028476	-1.13	9.54812E-06
ENSMUSG00000021990	-1.13	1.51994E-14
ENSMUSG00000069456	-1.13	8.57835E-15
ENSMUSG00000063889	-1.13	9.01788E-15
ENSMUSG00000037887	-1.13	0.000139292

Ensembl gene id	log2FC	p-adjusted
ENSMUSG00000031523	-1.13	1.5173E-15
ENSMUSG00000040943	-1.13	4.39616E-08
ENSMUSG00000032018	-1.14	4.99311E-05
ENSMUSG00000016520	-1.14	5.37477E-12
ENSMUSG00000047446	-1.14	2.6141E-06
ENSMUSG00000060317	-1.14	5.9517E-17
ENSMUSG00000070368	-1.14	0.00140001
ENSMUSG00000048240	-1.15	0.033212758
ENSMUSG00000020275	-1.15	0.000152235
ENSMUSG00000020154	-1.15	1.80947E-15
ENSMUSG00000040620	-1.15	1.4505E-14
ENSMUSG00000072623	-1.15	1.93586E-07
ENSMUSG00000029467	-1.16	4.5986E-06
ENSMUSG00000041741	-1.16	0.022772921
ENSMUSG00000027478	-1.16	1.0837E-07
ENSMUSG00000062624	-1.16	3.71924E-07
ENSMUSG00000020300	-1.16	2.51259E-16
ENSMUSG00000098678	-1.16	0.000648442
ENSMUSG00000038591	-1.16	2.26576E-16
ENSMUSG00000058056	-1.16	5.87849E-14
ENSMUSG00000028030	-1.17	8.88765E-08
ENSMUSG00000020123	-1.17	2.93535E-14
ENSMUSG00000026017	-1.17	0.000927504
ENSMUSG00000033487	-1.17	2.55926E-13
ENSMUSG00000044359	-1.17	1.85237E-09
ENSMUSG00000028519	-1.17	0.002769584
ENSMUSG00000021559	-1.17	3.92576E-18
ENSMUSG00000090942	-1.17	8.04832E-06
ENSMUSG00000037379	-1.18	0.020617062
ENSMUSG00000090290	-1.18	8.07801E-08
ENSMUSG00000025326	-1.18	2.94089E-10
ENSMUSG00000030424	-1.18	0.036546244
ENSMUSG00000024978	-1.18	1.85767E-10
ENSMUSG00000045776	-1.18	3.48098E-11
ENSMUSG00000019564	-1.18	0.008816411
ENSMUSG00000070576	-1.18	1.83934E-05
ENSMUSG00000018566	-1.19	0.00611071
ENSMUSG00000034342	-1.19	3.61559E-06
ENSMUSG00000038244	-1.19	4.6468E-15
ENSMUSG00000089948	-1.19	0.000940088
ENSMUSG00000020716	-1.19	5.33534E-15
ENSMUSG00000001229	-1.19	8.31265E-18
ENSMUSG00000072889	-1.19	1.06005E-09
ENSMUSG00000073016	-1.19	0.000819027
ENSMUSG00000045294	-1.20	0.009135039
ENSMUSG00000069825	-1.20	1.14556E-05
ENSMUSG00000098014	-1.20	0.000757283
ENSMUSG00000003847	-1.20	1.1734E-08
ENSMUSG00000028525	-1.20	0.012785903
ENSMUSG00000035840	-1.20	5.75221E-15
ENSMUSG00000075595	-1.20	3.76527E-17
ENSMUSG00000081788	-1.21	0.025398562

Ensembl gene id	log2FC	p-adjusted
ENSMUSG00000075470	-1.21	4.84922E-15
ENSMUSG00000078650	-1.22	2.84901E-13
ENSMUSG00000028715	-1.22	0.019544344
ENSMUSG00000097221	-1.22	0.024004465
ENSMUSG00000090641	-1.22	0.037231414
ENSMUSG00000025880	-1.23	5.36718E-08
ENSMUSG00000038538	-1.23	3.65241E-14
ENSMUSG00000032410	-1.23	2.53965E-07
ENSMUSG00000014547	-1.23	5.05174E-05
ENSMUSG00000042942	-1.23	0.000820417
ENSMUSG00000020429	-1.23	0.005921054
ENSMUSG00000031668	-1.24	1.50857E-05
ENSMUSG00000005580	-1.24	5.33427E-12
ENSMUSG00000047888	-1.24	2.54909E-17
ENSMUSG00000041180	-1.24	0.001093569
ENSMUSG00000097554	-1.24	6.84678E-15
ENSMUSG00000021690	-1.24	5.12579E-14
ENSMUSG00000033792	-1.24	4.49309E-08
ENSMUSG00000048271	-1.24	5.03337E-07
ENSMUSG00000035284	-1.24	3.11222E-17
ENSMUSG00000026384	-1.24	2.80236E-07
ENSMUSG00000027022	-1.24	0.002889405
ENSMUSG00000020883	-1.24	6.51892E-16
ENSMUSG00000063730	-1.24	0.00843191
ENSMUSG00000067219	-1.25	2.96755E-17
ENSMUSG00000038024	-1.25	1.08049E-14
ENSMUSG00000031665	-1.25	1.31345E-19
ENSMUSG00000032724	-1.25	1.49132E-14
ENSMUSG00000042010	-1.25	4.77317E-06
ENSMUSG00000044646	-1.25	0.00304643
ENSMUSG00000045180	-1.25	2.74211E-15
ENSMUSG00000027048	-1.25	1.60771E-19
ENSMUSG00000027077	-1.25	0.033827841
ENSMUSG00000018417	-1.25	1.99573E-20
ENSMUSG00000058006	-1.25	1.65477E-05
ENSMUSG00000068284	-1.26	2.82605E-19
ENSMUSG00000050445	-1.26	0.000821538
ENSMUSG00000035164	-1.26	6.69855E-10
ENSMUSG00000034936	-1.27	9.77939E-11
ENSMUSG00000039701	-1.27	0.000101301
ENSMUSG00000032238	-1.27	1.64898E-11
ENSMUSG00000002265	-1.27	0.010685727
ENSMUSG00000039879	-1.27	9.85361E-14
ENSMUSG00000044452	-1.27	2.16827E-12
ENSMUSG00000038056	-1.28	2.52997E-18
ENSMUSG00000020715	-1.28	7.26258E-19
ENSMUSG00000025194	-1.28	5.05248E-22
ENSMUSG00000060429	-1.28	1.57891E-15
ENSMUSG00000047153	-1.28	5.44174E-17
ENSMUSG00000054823	-1.28	5.37971E-05
ENSMUSG00000000567	-1.28	3.29799E-06
ENSMUSG00000032009	-1.28	3.93674E-10

Ensembl gene id	log2FC	p-adjusted
ENSMUSG00000020917	-1.29	0.011225509
ENSMUSG00000047787	-1.29	0.030195321
ENSMUSG00000042460	-1.29	6.59161E-15
ENSMUSG00000023067	-1.29	9.2394E-10
ENSMUSG00000029004	-1.30	1.70181E-13
ENSMUSG00000041482	-1.30	0.000198475
ENSMUSG00000027871	-1.30	0.037432473
ENSMUSG00000089774	-1.30	0.000131476
ENSMUSG00000026950	-1.30	6.81301E-07
ENSMUSG00000074272	-1.31	2.81965E-21
ENSMUSG00000048280	-1.31	7.72935E-05
ENSMUSG00000030771	-1.31	1.11223E-05
ENSMUSG00000047793	-1.31	2.09436E-13
ENSMUSG00000063446	-1.31	0.006703903
ENSMUSG00000046404	-1.31	7.69589E-06
ENSMUSG00000015090	-1.32	0.024743754
ENSMUSG00000027068	-1.32	0.018335546
ENSMUSG00000039835	-1.32	2.93551E-11
ENSMUSG00000052920	-1.32	0.010794302
ENSMUSG00000046962	-1.33	4.2782E-08
ENSMUSG00000027684	-1.34	6.01422E-05
ENSMUSG00000066364	-1.34	0.008124822
ENSMUSG00000072720	-1.34	0.000969968
ENSMUSG00000044938	-1.34	0.048810805
ENSMUSG00000039046	-1.34	1.70623E-20
ENSMUSG00000020532	-1.34	0.001181382
ENSMUSG00000041237	-1.34	1.54854E-07
ENSMUSG00000027313	-1.34	0.005757421
ENSMUSG00000038725	-1.34	0.018335546
ENSMUSG00000041702	-1.35	8.32457E-14
ENSMUSG00000029188	-1.35	0.00342786
ENSMUSG00000086819	-1.35	0.009096899
ENSMUSG00000064325	-1.35	0.00067534
ENSMUSG00000028354	-1.35	0.046501361
ENSMUSG00000029869	-1.35	0.044405403
ENSMUSG00000000305	-1.35	0.002261995
ENSMUSG00000014905	-1.35	1.3421E-07
ENSMUSG00000018362	-1.36	2.51594E-05
ENSMUSG00000015405	-1.36	0.008447903
ENSMUSG00000055980	-1.36	7.74654E-17
ENSMUSG00000031024	-1.36	0.004231317
ENSMUSG00000097615	-1.37	0.00010103
ENSMUSG00000025262	-1.37	9.51373E-09
ENSMUSG00000050919	-1.37	0.004768116
ENSMUSG00000028051	-1.37	0.001476593
ENSMUSG00000037621	-1.37	1.14673E-16
ENSMUSG00000027870	-1.37	1.81553E-13
ENSMUSG00000028681	-1.37	0.001127153
ENSMUSG00000045094	-1.38	3.34213E-11
ENSMUSG00000035133	-1.38	8.47488E-16
ENSMUSG00000091562	-1.38	0.006278512
ENSMUSG00000056342	-1.38	2.39037E-22

Ensembl gene id	log2FC	p-adjusted
ENSMUSG00000052812	-1.39	1.05433E-13
ENSMUSG00000003233	-1.39	5.28149E-15
ENSMUSG00000092008	-1.39	2.99657E-13
ENSMUSG00000024827	-1.39	4.11105E-25
ENSMUSG00000040957	-1.39	8.19835E-12
ENSMUSG00000029195	-1.39	3.03163E-21
ENSMUSG00000039967	-1.39	2.81965E-21
ENSMUSG00000031010	-1.40	1.15802E-24
ENSMUSG00000057637	-1.40	1.81575E-15
ENSMUSG00000053219	-1.40	0.000190417
ENSMUSG00000033855	-1.41	7.07289E-12
ENSMUSG00000038384	-1.41	8.77251E-20
ENSMUSG00000051747	-1.41	0.000862212
ENSMUSG00000025004	-1.41	6.8533E-13
ENSMUSG00000052632	-1.41	3.61686E-13
ENSMUSG00000022708	-1.42	2.32917E-06
ENSMUSG00000054178	-1.42	4.42593E-06
ENSMUSG00000034687	-1.42	1.01061E-12
ENSMUSG00000070985	-1.42	6.72792E-22
ENSMUSG00000016239	-1.42	4.58187E-06
ENSMUSG00000031529	-1.42	1.61818E-15
ENSMUSG00000046101	-1.42	0.005629123
ENSMUSG00000087574	-1.42	0.002989433
ENSMUSG00000025949	-1.43	1.67323E-17
ENSMUSG00000086389	-1.43	0.000120804
ENSMUSG00000025153	-1.43	0.005919403
ENSMUSG00000031770	-1.43	8.43828E-07
ENSMUSG00000001095	-1.43	0.009072869
ENSMUSG00000046876	-1.43	1.44295E-05
ENSMUSG00000027660	-1.44	5.58368E-22
ENSMUSG00000022521	-1.44	1.48301E-19
ENSMUSG00000067199	-1.45	0.030524196
ENSMUSG00000023044	-1.45	3.78618E-12
ENSMUSG00000023845	-1.45	9.11225E-17
ENSMUSG00000071083	-1.45	0.023351545
ENSMUSG00000040761	-1.45	1.77671E-22
ENSMUSG00000029512	-1.46	9.77462E-26
ENSMUSG00000030257	-1.46	4.89397E-05
ENSMUSG00000062901	-1.47	4.7054E-23
ENSMUSG00000033713	-1.47	1.35301E-13
ENSMUSG00000048732	-1.47	1.83629E-05
ENSMUSG00000020037	-1.48	0.033708821
ENSMUSG00000087691	-1.48	0.005962276
ENSMUSG00000033624	-1.48	1.04622E-19
ENSMUSG00000098290	-1.48	0.014025295
ENSMUSG00000024589	-1.48	4.76182E-16
ENSMUSG00000079065	-1.49	9.25532E-13
ENSMUSG00000020034	-1.49	4.68768E-19
ENSMUSG00000090369	-1.49	3.97058E-06
ENSMUSG00000097536	-1.50	0.003574756
ENSMUSG00000063975	-1.50	0.03877004
ENSMUSG00000084981	-1.50	0.00094158

Ensembl gene id	log2FC	p-adjusted
ENSMUSG00000030827	-1.51	0.010060269
ENSMUSG00000029385	-1.51	2.76679E-17
ENSMUSG00000038984	-1.51	0.039359607
ENSMUSG00000021767	-1.52	1.16251E-17
ENSMUSG00000084132	-1.52	0.045739832
ENSMUSG00000025272	-1.53	0.006539202
ENSMUSG00000043013	-1.53	0.002542667
ENSMUSG00000041930	-1.53	3.85811E-06
ENSMUSG00000091277	-1.54	0.026589516
ENSMUSG00000027605	-1.54	0.002944867
ENSMUSG00000025255	-1.55	3.00042E-22
ENSMUSG00000041078	-1.55	0.000149485
ENSMUSG00000040380	-1.55	0.007472188
ENSMUSG00000019935	-1.56	0.025438346
ENSMUSG00000063047	-1.56	8.22131E-06
ENSMUSG00000051335	-1.56	2.03558E-06
ENSMUSG00000085834	-1.56	9.29715E-07
ENSMUSG00000097715	-1.57	0.031637792
ENSMUSG00000001985	-1.57	0.012707707
ENSMUSG00000052595	-1.58	4.12805E-20
ENSMUSG00000021466	-1.58	1.89118E-13
ENSMUSG00000005893	-1.58	2.64334E-20
ENSMUSG00000083777	-1.58	0.006004494
ENSMUSG00000002250	-1.59	5.67504E-05
ENSMUSG00000054385	-1.59	8.6033E-05
ENSMUSG00000032735	-1.59	3.32251E-14
ENSMUSG00000020620	-1.59	3.22692E-30
ENSMUSG00000052229	-1.60	0.000121693
ENSMUSG00000042390	-1.60	3.17874E-13
ENSMUSG00000089943	-1.60	2.06E-25
ENSMUSG00000033863	-1.60	4.00469E-26
ENSMUSG00000038648	-1.60	1.36328E-11
ENSMUSG00000032417	-1.61	0.048587279
ENSMUSG00000039145	-1.61	2.55664E-32
ENSMUSG00000020027	-1.62	4.24381E-10
ENSMUSG00000048489	-1.63	3.40705E-27
ENSMUSG00000037541	-1.63	3.7497E-05
ENSMUSG00000015357	-1.64	3.41675E-20
ENSMUSG00000071456	-1.64	0.000320225
ENSMUSG00000032418	-1.65	1.52372E-06
ENSMUSG00000082364	-1.66	0.01690829
ENSMUSG00000037270	-1.66	7.40439E-33
ENSMUSG00000058672	-1.66	4.12665E-07
ENSMUSG00000031637	-1.66	5.03462E-08
ENSMUSG00000097090	-1.67	0.014138116
ENSMUSG00000053877	-1.67	1.48619E-20
ENSMUSG00000041536	-1.67	0.010039032
ENSMUSG00000022003	-1.68	2.77002E-06
ENSMUSG00000086245	-1.68	0.000387687
ENSMUSG00000042363	-1.70	2.78674E-29
ENSMUSG00000028039	-1.70	0.004129985
ENSMUSG00000049630	-1.71	0.004798798

Ensembl gene id	log2FC	p-adjusted
ENSMUSG00000072999	-1.72	3.94114E-06
ENSMUSG00000020354	-1.73	0.008859487
ENSMUSG00000019947	-1.73	1.30329E-06
ENSMUSG00000074925	-1.73	5.12351E-09
ENSMUSG00000094410	-1.74	7.42755E-17
ENSMUSG00000057230	-1.74	4.18421E-11
ENSMUSG00000018581	-1.74	8.50939E-05
ENSMUSG00000039704	-1.76	6.32153E-12
ENSMUSG00000026614	-1.78	4.02343E-38
ENSMUSG00000078185	-1.78	0.012026664
ENSMUSG00000051452	-1.79	1.07195E-10
ENSMUSG00000084923	-1.80	0.021172983
ENSMUSG00000061436	-1.80	1.00007E-30
ENSMUSG00000051000	-1.80	5.33641E-08
ENSMUSG00000085963	-1.80	5.4849E-07
ENSMUSG00000024298	-1.81	8.2461E-27
ENSMUSG00000084788	-1.84	0.0069143
ENSMUSG00000029238	-1.86	1.44583E-38
ENSMUSG00000098867	-1.87	0.031309881
ENSMUSG00000086645	-1.88	0.012956133
ENSMUSG00000020122	-1.88	1.61296E-06
ENSMUSG00000018868	-1.89	0.024180254
ENSMUSG00000086682	-1.89	1.79337E-05
ENSMUSG00000030762	-1.91	2.93245E-09
ENSMUSG00000043461	-1.91	0.002959474
ENSMUSG00000067049	-1.91	0.009228855
ENSMUSG00000026489	-1.92	1.29269E-46
ENSMUSG00000020889	-1.92	9.40973E-38
ENSMUSG00000072612	-1.92	0.032065423
ENSMUSG00000086231	-1.93	4.32596E-07
ENSMUSG00000052544	-1.93	0.005632125
ENSMUSG00000031286	-1.93	2.66759E-15
ENSMUSG00000090877	-1.94	0.01568655
ENSMUSG00000022615	-1.97	1.95825E-25
ENSMUSG00000074280	-1.98	4.13633E-16
ENSMUSG00000038594	-1.98	4.91358E-22
ENSMUSG00000099032	-1.98	0.009482149
ENSMUSG00000067336	-1.99	2.91668E-27
ENSMUSG00000041324	-2.02	1.65477E-05
ENSMUSG00000097417	-2.02	0.000248909
ENSMUSG00000031326	-2.02	1.73236E-09
ENSMUSG00000027796	-2.05	5.13885E-07
ENSMUSG00000097766	-2.05	1.33117E-06
ENSMUSG00000037071	-2.05	4.70523E-19
ENSMUSG00000091405	-2.09	0.007509183
ENSMUSG00000095079	-2.09	0.014860721
ENSMUSG00000027577	-2.10	0.037898711
ENSMUSG00000025019	-2.10	1.55792E-26
ENSMUSG00000071204	-2.11	7.3531E-22
ENSMUSG00000030046	-2.11	7.24297E-08
ENSMUSG00000072294	-2.12	1.27763E-26
ENSMUSG00000029491	-2.12	0.04012636

Ensembl gene id	log2FC	p-adjusted
ENSMUSG00000039552	-2.13	0.002677917
ENSMUSG00000056133	-2.17	0.005901202
ENSMUSG00000098741	-2.18	1.53081E-05
ENSMUSG00000038894	-2.18	5.06975E-35
ENSMUSG00000018427	-2.20	1.5629E-10
ENSMUSG00000083409	-2.22	0.005554247
ENSMUSG00000042515	-2.23	0.016406064
ENSMUSG00000067279	-2.24	5.80766E-34
ENSMUSG00000041596	-2.30	5.32518E-09
ENSMUSG00000020182	-2.31	0.009434777
ENSMUSG00000020593	-2.32	0.000582046
ENSMUSG00000092075	-2.33	0.000295996
ENSMUSG00000034584	-2.34	2.99044E-10
ENSMUSG00000091971	-2.39	1.82221E-11
ENSMUSG00000096991	-2.39	0.001348538
ENSMUSG00000024843	-2.41	4.09015E-21
ENSMUSG00000041653	-2.48	0.003119964
ENSMUSG00000025202	-2.52	1.05993E-11
ENSMUSG00000051906	-2.54	0.008727211
ENSMUSG00000089827	-2.59	4.86654E-05
ENSMUSG00000038917	-2.65	0.01443105
ENSMUSG00000038233	-2.77	7.55486E-55
ENSMUSG00000084834	-2.78	0.000187898
ENSMUSG00000097267	-2.78	0.002096094
ENSMUSG00000051497	-2.82	7.63494E-06
ENSMUSG00000072944	-2.88	0.037721373
ENSMUSG00000087579	-2.94	2.5935E-49
ENSMUSG00000066687	-2.94	9.22925E-37
ENSMUSG00000049690	-2.94	5.58368E-22
ENSMUSG00000033107	-3.03	1.24115E-75
ENSMUSG00000050195	-3.08	8.59934E-14
ENSMUSG00000041624	-3.09	2.58826E-10
ENSMUSG00000098022	-3.10	0.000434667
ENSMUSG00000029009	-3.11	1.25014E-24
ENSMUSG00000066407	-3.13	0.014659107
ENSMUSG00000045441	-3.24	1.96968E-56
ENSMUSG00000054453	-3.27	9.96753E-30
ENSMUSG00000086047	-3.31	0.029062614
ENSMUSG00000033717	-3.53	0.028303055
ENSMUSG00000097009	-3.63	0.007209432
ENSMUSG00000081909	-3.67	0.038144675
ENSMUSG00000074569	-3.70	0.000523438
ENSMUSG00000055116	-3.81	1.18046E-53
ENSMUSG00000020431	-3.90	2.02147E-38
ENSMUSG00000098037	-4.10	0.002686244
ENSMUSG00000082633	-4.36	0.033763882
ENSMUSG00000098882	-4.53	0.018565107
ENSMUSG00000085558	-4.83	0.035569973
ENSMUSG00000026077	-5.31	6.94398E-69
**Sm_Vh_39**		
ENSMUSG00000076614	7.94	0.012290075
ENSMUSG00000094749	7.42	6.62302E-38

Ensembl gene id	log2FC	p-adjusted
ENSMUSG00000063779	6.50	3.95363E-08
ENSMUSG00000083019	6.19	0.025887525
ENSMUSG00000058126	6.17	0.040620074
ENSMUSG00000095120	5.94	0.007452333
ENSMUSG00000068606	5.90	3.17141E-75
ENSMUSG00000020870	5.73	0.044513191
ENSMUSG00000016498	5.73	0.000181288
ENSMUSG00000032487	5.59	0.000999368
ENSMUSG00000076655	5.54	6.73748E-15
ENSMUSG00000094335	5.51	0.017671038
ENSMUSG00000095170	5.47	0.013663011
ENSMUSG00000050974	5.46	0.026662519
ENSMUSG00000009185	5.38	6.10405E-23
ENSMUSG00000063388	5.22	1.20209E-31
ENSMUSG00000022126	5.15	0.000107561
ENSMUSG00000093861	4.94	0.011528117
ENSMUSG00000035186	4.74	0.002965947
ENSMUSG00000068452	4.73	0.011150103
ENSMUSG00000095488	4.66	0.008521131
ENSMUSG00000094769	4.64	0.020704793
ENSMUSG00000044309	4.63	1.43299E-05
ENSMUSG00000040498	4.45	0.005475554
ENSMUSG00000005883	4.40	0.004761385
ENSMUSG00000063903	4.40	0.001458222
ENSMUSG00000028602	4.36	0.00462071
ENSMUSG00000053318	4.21	0.001286534
ENSMUSG00000082292	4.20	2.04094E-09
ENSMUSG00000050370	4.20	0.000138588
ENSMUSG00000020826	4.19	0.003674538
ENSMUSG00000096422	4.16	0.022625467
ENSMUSG00000057729	4.13	0.000129793
ENSMUSG00000028175	4.03	0.01778737
ENSMUSG00000095704	4.00	0.007923693
ENSMUSG00000029417	3.96	8.56399E-06
ENSMUSG00000095771	3.94	0.036288371
ENSMUSG00000030162	3.92	0.000156634
ENSMUSG00000034855	3.91	0.000229051
ENSMUSG00000026011	3.88	0.024816077
ENSMUSG00000024401	3.85	1.17353E-05
ENSMUSG00000087642	3.84	0.007355644
ENSMUSG00000028965	3.83	0.000123397
ENSMUSG00000076609	3.80	0.012446141
ENSMUSG00000035373	3.79	1.42647E-06
ENSMUSG00000078922	3.78	3.03253E-09
ENSMUSG00000057465	3.75	4.06821E-19
ENSMUSG00000046031	3.74	2.57353E-06
ENSMUSG00000027656	3.71	1.23057E-07
ENSMUSG00000084072	3.70	0.000383283
ENSMUSG00000067149	3.69	0.017433197
ENSMUSG00000026390	3.68	0.001579602
ENSMUSG00000026822	3.67	9.25973E-10
ENSMUSG00000078921	3.65	1.67333E-08

Ensembl gene id	log2FC	p-adjusted
ENSMUSG00000022651	3.64	9.83949E-05
ENSMUSG00000001131	3.64	0.000105263
ENSMUSG00000028270	3.63	1.48907E-05
ENSMUSG00000058755	3.58	0.000789111
ENSMUSG00000055170	3.56	0.005209281
ENSMUSG00000030283	3.54	0.004537522
ENSMUSG00000042345	3.54	0.00268507
ENSMUSG00000054203	3.54	0.015576332
ENSMUSG00000044827	3.50	8.18902E-07
ENSMUSG00000074115	3.49	1.75311E-14
ENSMUSG00000091694	3.49	0.000102367
ENSMUSG00000046591	3.46	0.007033544
ENSMUSG00000038037	3.45	0.000469884
ENSMUSG00000020676	3.45	0.000124998
ENSMUSG00000076617	3.43	3.76877E-27
ENSMUSG00000062380	3.42	0.002040509
ENSMUSG00000045502	3.40	0.001664278
ENSMUSG00000026285	3.39	0.016314496
ENSMUSG00000028362	3.35	0.000374904
ENSMUSG00000027718	3.32	0.003257311
ENSMUSG00000071552	3.30	0.009380103
ENSMUSG00000015134	3.29	3.30573E-05
ENSMUSG00000028268	3.29	0.000121745
ENSMUSG00000076564	3.29	0.030719593
ENSMUSG00000018930	3.29	0.005673254
ENSMUSG00000082319	3.23	0.004870729
ENSMUSG00000037868	3.22	0.010442506
ENSMUSG00000005696	3.20	0.006225132
ENSMUSG00000076934	3.20	0.005195492
ENSMUSG00000048852	3.19	4.42612E-06
ENSMUSG00000064246	3.19	0.000800665
ENSMUSG00000004814	3.17	0.000124154
ENSMUSG00000044162	3.16	0.004971379
ENSMUSG00000041754	3.15	0.014642548
ENSMUSG00000042489	3.15	0.001440948
ENSMUSG00000024989	3.15	0.005859262
ENSMUSG00000060586	3.15	2.11094E-05
ENSMUSG00000079363	3.14	0.000221214
ENSMUSG00000040264	3.14	0.000179674
ENSMUSG00000047462	3.13	0.002172172
ENSMUSG00000078963	3.12	9.50091E-14
ENSMUSG00000085154	3.12	0.007923693
ENSMUSG00000096336	3.10	0.033177349
ENSMUSG00000024675	3.10	2.08304E-08
ENSMUSG00000041481	3.08	0.003892423
ENSMUSG00000018500	3.08	0.004751933
ENSMUSG00000040026	3.07	1.60445E-05
ENSMUSG00000030200	3.06	0.009848511
ENSMUSG00000078853	3.06	1.75499E-10
ENSMUSG00000059824	3.05	1.40649E-55
ENSMUSG00000016496	3.05	3.47108E-05
ENSMUSG00000031495	3.05	0.024071698

Ensembl gene id	log2FC	p-adjusted
ENSMUSG00000051969	3.05	0.001624164
ENSMUSG00000089929	3.05	0.000123372
ENSMUSG00000074345	3.04	0.002820902
ENSMUSG00000058427	3.04	0.007918117
ENSMUSG00000020057	3.02	0.000259353
ENSMUSG00000044701	3.02	0.001624164
ENSMUSG00000020808	3.02	0.014632366
ENSMUSG00000022479	3.02	0.004206762
ENSMUSG00000087273	3.00	2.44419E-05
ENSMUSG00000000682	3.00	0.000309062
ENSMUSG00000027514	3.00	7.94848E-12
ENSMUSG00000038179	2.99	0.034264618
ENSMUSG00000019027	2.99	0.004924185
ENSMUSG00000040809	2.99	0.002875181
ENSMUSG00000023132	2.98	0.040109993
ENSMUSG00000034987	2.98	0.016130358
ENSMUSG00000076583	2.98	0.045951993
ENSMUSG00000022033	2.98	0.000189481
ENSMUSG00000037474	2.98	0.002875181
ENSMUSG00000053977	2.95	0.002716629
ENSMUSG00000013766	2.95	0.02569825
ENSMUSG00000034438	2.94	0.00050231
ENSMUSG00000020914	2.94	0.00076807
ENSMUSG00000082976	2.94	0.015950034
ENSMUSG00000072599	2.94	0.015413606
ENSMUSG00000015437	2.92	0.002212686
ENSMUSG00000027379	2.92	0.008153872
ENSMUSG00000076586	2.91	0.005035551
ENSMUSG00000079018	2.91	1.36398E-07
ENSMUSG00000022586	2.90	0.000374621
ENSMUSG00000024353	2.89	0.010051935
ENSMUSG00000022584	2.89	3.71665E-06
ENSMUSG00000036067	2.88	0.000717617
ENSMUSG00000053101	2.87	0.012079654
ENSMUSG00000030156	2.87	0.001359761
ENSMUSG00000030867	2.86	0.000307254
ENSMUSG00000002835	2.86	0.00027325
ENSMUSG00000098318	2.85	0.011232492
ENSMUSG00000021624	2.85	0.000420484
ENSMUSG00000076937	2.85	0.018116821
ENSMUSG00000001228	2.84	0.000357871
ENSMUSG00000035683	2.83	0.015721575
ENSMUSG00000079455	2.82	0.009319772
ENSMUSG00000035385	2.81	4.26746E-05
ENSMUSG00000058252	2.81	0.025533058
ENSMUSG00000024056	2.81	0.00671526
ENSMUSG00000026582	2.81	2.56669E-05
ENSMUSG00000072596	2.81	0.012995618
ENSMUSG00000035455	2.79	0.000484988
ENSMUSG00000015314	2.79	0.001142979
ENSMUSG00000067341	2.79	0.018058489
ENSMUSG00000020897	2.78	0.000878529

Ensembl gene id	log2FC	p-adjusted
ENSMUSG00000050994	2.78	0.002692689
ENSMUSG00000022021	2.78	0.000936682
ENSMUSG00000030413	2.78	0.000950131
ENSMUSG00000082902	2.78	0.004908717
ENSMUSG00000079362	2.77	3.63684E-08
ENSMUSG00000075602	2.77	5.60351E-05
ENSMUSG00000029275	2.76	0.015904813
ENSMUSG00000031779	2.75	6.3026E-06
ENSMUSG00000021259	2.74	0.006613881
ENSMUSG00000046341	2.74	6.49172E-05
ENSMUSG00000030154	2.73	0.04326988
ENSMUSG00000094872	2.73	0.019102172
ENSMUSG00000030789	2.73	0.007241188
ENSMUSG00000000982	2.73	0.005113796
ENSMUSG00000078521	2.72	0.034558301
ENSMUSG00000026981	2.72	0.000186751
ENSMUSG00000026630	2.72	1.83357E-06
ENSMUSG00000037548	2.71	0.000452734
ENSMUSG00000045328	2.70	0.000166795
ENSMUSG00000022322	2.70	0.000123397
ENSMUSG00000018924	2.70	5.01237E-05
ENSMUSG00000016283	2.69	0.007923693
ENSMUSG00000022504	2.69	0.000121649
ENSMUSG00000022422	2.69	0.005725636
ENSMUSG00000025804	2.69	0.006340739
ENSMUSG00000041202	2.69	0.004572447
ENSMUSG00000029414	2.69	0.000852961
ENSMUSG00000030144	2.68	0.024414472
ENSMUSG00000068129	2.68	0.001637648
ENSMUSG00000069910	2.68	2.23704E-05
ENSMUSG00000096727	2.66	6.34954E-13
ENSMUSG00000035042	2.66	0.012940909
ENSMUSG00000037411	2.66	6.73273E-12
ENSMUSG00000044313	2.65	0.010581435
ENSMUSG00000090272	2.65	0.005509899
ENSMUSG00000092021	2.65	1.05027E-06
ENSMUSG00000051378	2.65	0.002897169
ENSMUSG00000026104	2.64	6.3026E-06
ENSMUSG00000041498	2.63	0.029687408
ENSMUSG00000000791	2.63	1.19643E-05
ENSMUSG00000021880	2.63	4.76565E-08
ENSMUSG00000017499	2.63	0.005583801
ENSMUSG00000003484	2.62	0.00011956
ENSMUSG00000037649	2.62	2.22133E-05
ENSMUSG00000067768	2.62	0.015861121
ENSMUSG00000025701	2.62	0.033006521
ENSMUSG00000032783	2.61	0.0026091
ENSMUSG00000038379	2.61	0.003546449
ENSMUSG00000024669	2.61	0.017473329
ENSMUSG00000031933	2.61	0.007813096
ENSMUSG00000035165	2.60	0.023693288
ENSMUSG00000024791	2.60	0.001128005

Ensembl gene id	log2FC	p-adjusted
ENSMUSG00000015316	2.60	0.034264618
ENSMUSG00000032586	2.60	0.005892918
ENSMUSG00000004612	2.59	0.003093846
ENSMUSG00000037321	2.59	6.45643E-07
ENSMUSG00000021175	2.59	0.006649005
ENSMUSG00000034266	2.58	4.63181E-07
ENSMUSG00000031896	2.58	0.004541668
ENSMUSG00000029923	2.58	9.09835E-05
ENSMUSG00000079547	2.57	0.000530449
ENSMUSG00000005410	2.57	0.000263277
ENSMUSG00000045868	2.56	0.033702007
ENSMUSG00000002204	2.56	1.08473E-08
ENSMUSG00000017652	2.55	0.0012391
ENSMUSG00000023908	2.55	0.000182495
ENSMUSG00000046971	2.55	0.032644414
ENSMUSG00000049103	2.54	0.001322959
ENSMUSG00000078920	2.53	8.61691E-09
ENSMUSG00000033847	2.53	0.006400947
ENSMUSG00000032446	2.51	0.005436904
ENSMUSG00000013974	2.51	0.004095507
ENSMUSG00000024670	2.51	0.002560511
ENSMUSG00000005360	2.50	4.70894E-06
ENSMUSG00000036768	2.49	0.020704793
ENSMUSG00000037628	2.49	0.008711776
ENSMUSG00000056737	2.48	5.96155E-07
ENSMUSG00000097993	2.48	0.013982965
ENSMUSG00000031304	2.47	1.15336E-05
ENSMUSG00000073421	2.47	0.00011685
ENSMUSG00000095649	2.46	0.042313788
ENSMUSG00000025877	2.45	2.07159E-05
ENSMUSG00000055809	2.45	0.000364523
ENSMUSG00000039187	2.45	0.001321666
ENSMUSG00000034773	2.45	0.011195161
ENSMUSG00000064147	2.45	0.010255418
ENSMUSG00000070000	2.45	0.010428113
ENSMUSG00000076613	2.44	0.005294852
ENSMUSG00000059089	2.44	3.50263E-08
ENSMUSG00000028068	2.44	0.01778737
ENSMUSG00000095788	2.44	0.007169504
ENSMUSG00000025163	2.43	0.001158377
ENSMUSG00000026683	2.43	7.5909E-05
ENSMUSG00000041831	2.43	0.031664559
ENSMUSG00000039699	2.43	3.51512E-05
ENSMUSG00000037991	2.43	0.013883837
ENSMUSG00000076490	2.43	0.001206074
ENSMUSG00000006398	2.43	4.68591E-07
ENSMUSG00000029272	2.42	0.012916889
ENSMUSG00000043931	2.42	0.011616882
ENSMUSG00000030793	2.41	3.10369E-05
ENSMUSG00000024397	2.39	0.0007518
ENSMUSG00000030346	2.39	0.010161334
ENSMUSG00000026573	2.39	0.000778885

Ensembl gene id	log2FC	p-adjusted
ENSMUSG00000051998	2.39	0.005995206
ENSMUSG00000064109	2.38	0.003046406
ENSMUSG00000031004	2.37	3.17453E-07
ENSMUSG00000031762	2.37	2.77004E-31
ENSMUSG00000087060	2.36	0.011433553
ENSMUSG00000037544	2.35	0.008712286
ENSMUSG00000029298	2.35	0.00433524
ENSMUSG00000036587	2.35	0.021351571
ENSMUSG00000028678	2.34	1.9535E-05
ENSMUSG00000054072	2.34	5.92477E-05
ENSMUSG00000026271	2.33	0.000241862
ENSMUSG00000040899	2.33	0.0397742
ENSMUSG00000005470	2.33	0.003932735
ENSMUSG00000040152	2.33	6.14958E-05
ENSMUSG00000062545	2.33	0.000327327
ENSMUSG00000043932	2.33	0.023392249
ENSMUSG00000029915	2.33	0.011789373
ENSMUSG00000046295	2.33	0.039951272
ENSMUSG00000050335	2.32	0.000158856
ENSMUSG00000031884	2.31	0.001878618
ENSMUSG00000037337	2.31	0.000210483
ENSMUSG00000025473	2.31	0.001624164
ENSMUSG00000069874	2.31	9.55421E-15
ENSMUSG00000006585	2.30	0.000521122
ENSMUSG00000067714	2.30	0.006473271
ENSMUSG00000030142	2.30	0.036725087
ENSMUSG00000015880	2.30	0.020026261
ENSMUSG00000045826	2.29	9.90116E-05
ENSMUSG00000062524	2.29	0.006519728
ENSMUSG00000048215	2.29	0.022196597
ENSMUSG00000021262	2.29	3.74409E-05
ENSMUSG00000039013	2.28	0.0120376
ENSMUSG00000060183	2.28	2.44634E-21
ENSMUSG00000027368	2.28	0.01064642
ENSMUSG00000051220	2.28	0.005046565
ENSMUSG00000027469	2.28	0.00099502
ENSMUSG00000061540	2.27	6.10405E-23
ENSMUSG00000024610	2.27	0.000138479
ENSMUSG00000019942	2.27	0.002456075
ENSMUSG00000052131	2.27	4.75041E-07
ENSMUSG00000026581	2.27	0.000125375
ENSMUSG00000048327	2.26	0.00910478
ENSMUSG00000039055	2.25	0.032337404
ENSMUSG00000028718	2.24	0.005337936
ENSMUSG00000023033	2.24	0.005599983
ENSMUSG00000039981	2.24	0.000342021
ENSMUSG00000050014	2.24	0.007235659
ENSMUSG00000070691	2.23	0.006347156
ENSMUSG00000024300	2.23	0.000536862
ENSMUSG00000002033	2.23	1.39877E-07
ENSMUSG00000026875	2.23	5.12526E-05
ENSMUSG00000039899	2.23	0.001712157

Ensembl gene id	log2FC	p-adjusted
ENSMUSG00000024334	2.23	0.001995154
ENSMUSG00000046080	2.22	0.039165208
ENSMUSG00000079293	2.22	2.44588E-07
ENSMUSG00000052270	2.21	0.01281305
ENSMUSG00000027715	2.21	6.06936E-05
ENSMUSG00000002870	2.20	0.000373502
ENSMUSG00000026009	2.20	9.73635E-10
ENSMUSG00000045763	2.20	0.002447198
ENSMUSG00000048534	2.20	0.00664331
ENSMUSG00000015981	2.20	0.003905933
ENSMUSG00000035692	2.19	3.75503E-07
ENSMUSG00000037731	2.19	7.84316E-06
ENSMUSG00000021822	2.19	0.001536416
ENSMUSG00000040084	2.18	0.010456411
ENSMUSG00000023015	2.18	0.001344686
ENSMUSG00000024301	2.17	0.014843628
ENSMUSG00000029380	2.17	7.03438E-08
ENSMUSG00000051457	2.17	0.000224259
ENSMUSG00000031821	2.17	3.8839E-05
ENSMUSG00000022034	2.17	0.009360686
ENSMUSG00000021322	2.17	0.033221414
ENSMUSG00000053044	2.17	0.031078651
ENSMUSG00000001020	2.17	0.000357871
ENSMUSG00000040253	2.17	0.001846359
ENSMUSG00000036594	2.17	0.000271312
ENSMUSG00000040950	2.16	0.000658936
ENSMUSG00000021356	2.16	0.005730097
ENSMUSG00000030677	2.16	0.005761548
ENSMUSG00000053063	2.16	0.009990688
ENSMUSG00000000204	2.16	3.74284E-05
ENSMUSG00000037466	2.15	0.008603983
ENSMUSG00000055235	2.15	0.004978993
ENSMUSG00000047880	2.15	0.003884494
ENSMUSG00000021263	2.15	0.001857912
ENSMUSG00000022673	2.14	9.45264E-05
ENSMUSG00000021423	2.14	0.000214188
ENSMUSG00000041859	2.14	3.71895E-05
ENSMUSG00000030149	2.13	0.002641045
ENSMUSG00000027863	2.13	0.005166935
ENSMUSG00000030577	2.13	0.000546235
ENSMUSG00000056162	2.13	0.03231002
ENSMUSG00000061100	2.13	0.001770597
ENSMUSG00000076498	2.12	0.003799128
ENSMUSG00000032254	2.12	0.009538362
ENSMUSG00000005540	2.12	3.2497E-07
ENSMUSG00000039942	2.12	0.001261539
ENSMUSG00000030742	2.12	0.000753408
ENSMUSG00000026429	2.12	0.016076685
ENSMUSG00000073902	2.11	4.6462E-05
ENSMUSG00000024679	2.11	2.94686E-05
ENSMUSG00000029322	2.11	4.54034E-07
ENSMUSG00000073489	2.11	0.005301338

Ensembl gene id	log2FC	p-adjusted
ENSMUSG00000027347	2.11	0.000465412
ENSMUSG00000044703	2.11	0.001378859
ENSMUSG00000066363	2.11	0.001117118
ENSMUSG00000061825	2.11	7.51467E-07
ENSMUSG00000001444	2.10	0.001094695
ENSMUSG00000051212	2.10	0.000327327
ENSMUSG00000027331	2.10	0.000281658
ENSMUSG00000050578	2.09	0.013697783
ENSMUSG00000017716	2.09	0.012150351
ENSMUSG00000000817	2.08	0.016584555
ENSMUSG00000039774	2.08	0.00470023
ENSMUSG00000060509	2.08	2.52762E-05
ENSMUSG00000041538	2.08	0.000125481
ENSMUSG00000026196	2.08	0.005583801
ENSMUSG00000012519	2.07	4.63507E-09
ENSMUSG00000027995	2.07	0.002082935
ENSMUSG00000028459	2.07	1.28959E-09
ENSMUSG00000004698	2.07	2.05702E-06
ENSMUSG00000050107	2.07	0.000947702
ENSMUSG00000076469	2.07	0.008350535
ENSMUSG00000072980	2.07	0.043934338
ENSMUSG00000049723	2.07	0.020567711
ENSMUSG00000084796	2.06	0.033845041
ENSMUSG00000061577	2.06	0.000182842
ENSMUSG00000070034	2.05	1.89412E-06
ENSMUSG00000012889	2.05	0.024746702
ENSMUSG00000071714	2.05	2.61206E-05
ENSMUSG00000092517	2.05	0.01343727
ENSMUSG00000031289	2.05	0.000747545
ENSMUSG00000040329	2.04	8.12002E-08
ENSMUSG00000022657	2.04	0.002954565
ENSMUSG00000056413	2.04	0.000155466
ENSMUSG00000032271	2.03	0.024338674
ENSMUSG00000032053	2.03	0.00332519
ENSMUSG00000004267	2.03	0.018610825
ENSMUSG00000010142	2.03	0.005883757
ENSMUSG00000031016	2.03	4.68922E-08
ENSMUSG00000004707	2.03	0.000247746
ENSMUSG00000000486	2.02	3.15282E-05
ENSMUSG00000026039	2.02	0.040849622
ENSMUSG00000031506	2.02	0.009049246
ENSMUSG00000045322	2.02	0.002998292
ENSMUSG00000024338	2.02	3.75503E-07
ENSMUSG00000023940	2.01	0.044414086
ENSMUSG00000038943	2.01	6.26206E-06
ENSMUSG00000022102	2.01	3.15202E-05
ENSMUSG00000057058	2.01	0.019198588
ENSMUSG00000022057	2.01	0.000212844
ENSMUSG00000039304	2.00	0.004270862
ENSMUSG00000055541	2.00	0.001472646
ENSMUSG00000029075	2.00	0.016753882
ENSMUSG00000063646	2.00	0.014053206

Ensembl gene id	log2FC	p-adjusted
ENSMUSG00000031838	2.00	7.68012E-06
ENSMUSG00000034459	2.00	6.56179E-06
ENSMUSG00000062593	1.99	0.001178579
ENSMUSG00000091649	1.99	0.001718283
ENSMUSG00000046879	1.99	7.57046E-07
ENSMUSG00000049625	1.99	0.000586339
ENSMUSG00000026605	1.99	0.000211388
ENSMUSG00000025161	1.99	6.11358E-05
ENSMUSG00000018168	1.98	0.003932417
ENSMUSG00000091345	1.98	4.6117E-08
ENSMUSG00000074375	1.98	0.020239449
ENSMUSG00000002055	1.97	0.005397676
ENSMUSG00000033762	1.97	0.003572869
ENSMUSG00000087213	1.97	0.002172172
ENSMUSG00000033220	1.97	3.71895E-05
ENSMUSG00000040592	1.97	2.53421E-06
ENSMUSG00000074896	1.96	3.44891E-05
ENSMUSG00000098090	1.96	0.015761041
ENSMUSG00000034853	1.96	0.002919014
ENSMUSG00000027962	1.96	0.00023894
ENSMUSG00000018927	1.95	4.84581E-06
ENSMUSG00000000318	1.95	0.035465343
ENSMUSG00000090164	1.95	0.004504825
ENSMUSG00000071068	1.94	0.007523815
ENSMUSG00000020120	1.94	0.002981934
ENSMUSG00000085501	1.94	0.004079018
ENSMUSG00000073412	1.94	0.000865161
ENSMUSG00000022534	1.94	9.85701E-05
ENSMUSG00000031662	1.94	0.006474796
ENSMUSG00000029204	1.93	4.73056E-05
ENSMUSG00000020732	1.93	0.005413918
ENSMUSG00000030707	1.93	0.001344686
ENSMUSG00000070427	1.93	0.000126602
ENSMUSG00000046805	1.93	9.60981E-07
ENSMUSG00000041515	1.93	2.96124E-05
ENSMUSG00000019214	1.93	0.008352566
ENSMUSG00000022103	1.93	0.00028814
ENSMUSG00000079071	1.93	0.001634767
ENSMUSG00000023349	1.92	6.34463E-05
ENSMUSG00000032322	1.92	0.000104181
ENSMUSG00000034317	1.92	0.002360432
ENSMUSG00000052013	1.92	7.84316E-06
ENSMUSG00000027306	1.91	0.007922222
ENSMUSG00000034634	1.91	0.015521705
ENSMUSG00000039126	1.91	0.001123305
ENSMUSG00000051444	1.91	0.000841083
ENSMUSG00000081512	1.91	0.007148423
ENSMUSG00000072620	1.91	0.001668458
ENSMUSG00000041431	1.91	0.010102096
ENSMUSG00000076752	1.91	0.018698953
ENSMUSG00000038768	1.91	0.011222242
ENSMUSG00000073008	1.90	0.009709784

Ensembl gene id	log2FC	p-adjusted
ENSMUSG00000004933	1.90	0.000357564
ENSMUSG00000017861	1.90	0.020023566
ENSMUSG00000074151	1.90	7.51814E-10
ENSMUSG00000030165	1.90	0.016653193
ENSMUSG00000030830	1.90	0.00013209
ENSMUSG00000000386	1.89	0.001000896
ENSMUSG00000026649	1.89	0.044697695
ENSMUSG00000025912	1.89	0.010170921
ENSMUSG00000057135	1.89	7.87536E-07
ENSMUSG00000003283	1.89	4.93159E-05
ENSMUSG00000015355	1.89	0.002747995
ENSMUSG00000040751	1.89	0.012445288
ENSMUSG00000085887	1.89	0.018130262
ENSMUSG00000030468	1.88	0.000182842
ENSMUSG00000028702	1.88	0.006458252
ENSMUSG00000071005	1.88	0.006525025
ENSMUSG00000028931	1.88	0.008108385
ENSMUSG00000000861	1.88	0.000712241
ENSMUSG00000030724	1.88	4.25315E-08
ENSMUSG00000000409	1.88	0.00254579
ENSMUSG00000021451	1.88	0.001076093
ENSMUSG00000032218	1.88	4.82167E-06
ENSMUSG00000027639	1.87	4.37406E-06
ENSMUSG00000026068	1.87	0.000735683
ENSMUSG00000025058	1.87	0.001160231
ENSMUSG00000015947	1.87	6.1408E-11
ENSMUSG00000036634	1.87	0.014688915
ENSMUSG00000041707	1.87	7.78751E-06
ENSMUSG00000026535	1.87	0.020947223
ENSMUSG00000052087	1.87	9.02501E-05
ENSMUSG00000027323	1.86	0.010836905
ENSMUSG00000050921	1.86	0.00312237
ENSMUSG00000032091	1.86	0.021093032
ENSMUSG00000030148	1.86	0.001423476
ENSMUSG00000079553	1.86	0.002426769
ENSMUSG00000076612	1.85	0.009109149
ENSMUSG00000027635	1.85	0.000281639
ENSMUSG00000086763	1.85	0.026480267
ENSMUSG00000021298	1.84	0.001940709
ENSMUSG00000054717	1.84	0.000883884
ENSMUSG00000032400	1.84	0.01415394
ENSMUSG00000027219	1.83	0.00019295
ENSMUSG00000019590	1.83	0.012373449
ENSMUSG00000035232	1.83	4.97139E-05
ENSMUSG00000027611	1.83	0.002182209
ENSMUSG00000040247	1.83	0.00273455
ENSMUSG00000058290	1.83	0.001858882
ENSMUSG00000022439	1.83	0.003974674
ENSMUSG00000083847	1.83	0.017443769
ENSMUSG00000033355	1.83	5.43266E-07
ENSMUSG00000052760	1.83	0.001114709
ENSMUSG00000050350	1.82	0.017102761

Ensembl gene id	log2FC	p-adjusted
ENSMUSG00000078763	1.82	0.005332553
ENSMUSG00000002068	1.82	0.036742635
ENSMUSG00000047810	1.82	0.001675816
ENSMUSG00000025888	1.82	0.000123269
ENSMUSG00000036526	1.82	0.005069957
ENSMUSG00000040204	1.82	0.015761041
ENSMUSG00000005087	1.82	0.000311139
ENSMUSG00000031264	1.82	4.38214E-08
ENSMUSG00000024910	1.82	0.00273455
ENSMUSG00000034792	1.81	0.000123269
ENSMUSG00000089672	1.81	0.006347156
ENSMUSG00000022945	1.81	0.000217875
ENSMUSG00000001517	1.81	0.032288457
ENSMUSG00000025001	1.80	0.003879083
ENSMUSG00000031101	1.80	1.01496E-07
ENSMUSG00000079419	1.80	0.000634592
ENSMUSG00000023274	1.80	0.001854038
ENSMUSG00000028873	1.80	0.006934866
ENSMUSG00000078965	1.80	0.010170077
ENSMUSG00000051735	1.80	0.000546235
ENSMUSG00000068105	1.80	0.006282369
ENSMUSG00000095298	1.80	0.019635723
ENSMUSG00000002111	1.80	1.85394E-07
ENSMUSG00000064023	1.80	0.030810546
ENSMUSG00000030107	1.79	1.52827E-12
ENSMUSG00000038642	1.79	5.12526E-05
ENSMUSG00000073705	1.79	0.033636498
ENSMUSG00000009687	1.79	0.000453969
ENSMUSG00000002699	1.79	8.38062E-05
ENSMUSG00000026126	1.78	0.000890107
ENSMUSG00000018899	1.78	8.72151E-10
ENSMUSG00000063193	1.78	1.41801E-10
ENSMUSG00000022221	1.78	2.09901E-05
ENSMUSG00000022385	1.78	0.04180054
ENSMUSG00000069793	1.78	0.006293192
ENSMUSG00000097194	1.78	0.000765392
ENSMUSG00000045087	1.77	0.008038434
ENSMUSG00000051076	1.77	0.009839427
ENSMUSG00000004709	1.77	0.000121758
ENSMUSG00000004952	1.77	0.000492832
ENSMUSG00000030220	1.77	3.43391E-06
ENSMUSG00000034116	1.77	2.60435E-06
ENSMUSG00000071713	1.76	4.43214E-06
ENSMUSG00000051517	1.76	0.011706379
ENSMUSG00000068227	1.76	0.015665805
ENSMUSG00000022372	1.76	0.000139705
ENSMUSG00000049775	1.76	0.000122899
ENSMUSG00000079227	1.76	0.000329759
ENSMUSG00000018008	1.75	0.000121758
ENSMUSG00000026786	1.75	0.000729513
ENSMUSG00000032661	1.74	2.33581E-07
ENSMUSG00000024673	1.74	1.25197E-07

Ensembl gene id	log2FC	p-adjusted
ENSMUSG00000037997	1.74	3.66895E-08
ENSMUSG00000013155	1.74	0.00641707
ENSMUSG00000029254	1.74	0.001185162
ENSMUSG00000029561	1.74	4.97463E-07
ENSMUSG00000045679	1.74	0.007238322
ENSMUSG00000024036	1.74	3.04543E-13
ENSMUSG00000026832	1.74	0.000342363
ENSMUSG00000034786	1.73	8.23404E-07
ENSMUSG00000003379	1.73	1.29649E-05
ENSMUSG00000092550	1.73	0.027975009
ENSMUSG00000039109	1.73	0.00048738
ENSMUSG00000024885	1.73	0.00010954
ENSMUSG00000024013	1.73	0.000405016
ENSMUSG00000043740	1.73	2.00428E-06
ENSMUSG00000018819	1.73	0.002543098
ENSMUSG00000028874	1.72	0.003906939
ENSMUSG00000024677	1.72	0.000155737
ENSMUSG00000001403	1.72	0.001914939
ENSMUSG00000056069	1.72	0.002413235
ENSMUSG00000059994	1.71	0.024736578
ENSMUSG00000025007	1.71	7.74907E-06
ENSMUSG00000029413	1.71	1.26829E-05
ENSMUSG00000012443	1.71	0.018528324
ENSMUSG00000021614	1.71	0.003142598
ENSMUSG00000037725	1.71	0.000286497
ENSMUSG00000050357	1.70	0.004810502
ENSMUSG00000038421	1.70	0.002834127
ENSMUSG00000020395	1.70	0.005212796
ENSMUSG00000030124	1.70	0.012587708
ENSMUSG00000062510	1.70	0.004927516
ENSMUSG00000056145	1.70	0.016527582
ENSMUSG00000000805	1.70	0.019364255
ENSMUSG00000047798	1.70	0.008454666
ENSMUSG00000019845	1.69	0.013126922
ENSMUSG00000040907	1.69	0.034372165
ENSMUSG00000062939	1.69	0.014028595
ENSMUSG00000032011	1.69	4.20674E-05
ENSMUSG00000027797	1.69	0.009286891
ENSMUSG00000018654	1.68	0.001142979
ENSMUSG00000074183	1.68	0.009983214
ENSMUSG00000006519	1.68	0.000215126
ENSMUSG00000003541	1.68	0.000196031
ENSMUSG00000041827	1.68	0.000246523
ENSMUSG00000024987	1.68	0.023834591
ENSMUSG00000060675	1.68	0.001354106
ENSMUSG00000040483	1.68	1.87679E-08
ENSMUSG00000034783	1.67	0.011307867
ENSMUSG00000028957	1.67	6.0009E-19
ENSMUSG00000043832	1.67	0.002116786
ENSMUSG00000028037	1.67	0.000651058
ENSMUSG00000097180	1.67	0.005890102
ENSMUSG00000039936	1.67	0.000974371

Ensembl gene id	log2FC	p-adjusted
ENSMUSG00000044708	1.67	0.035465343
ENSMUSG00000037860	1.67	0.000366633
ENSMUSG00000038352	1.67	0.007315344
ENSMUSG00000048521	1.67	0.002477249
ENSMUSG00000031897	1.67	5.29019E-08
ENSMUSG00000034311	1.66	0.006843612
ENSMUSG00000041219	1.66	0.006257373
ENSMUSG00000003779	1.66	0.001160231
ENSMUSG00000046688	1.66	7.20168E-17
ENSMUSG00000021208	1.66	0.000343859
ENSMUSG00000059326	1.66	1.42544E-06
ENSMUSG00000078798	1.66	0.001186213
ENSMUSG00000028480	1.66	0.003313709
ENSMUSG00000030589	1.66	0.00187125
ENSMUSG00000092277	1.66	0.004744391
ENSMUSG00000045165	1.66	0.000138106
ENSMUSG00000027326	1.66	0.034025356
ENSMUSG00000032093	1.66	0.00309131
ENSMUSG00000006219	1.66	1.41755E-09
ENSMUSG00000049988	1.65	0.000477722
ENSMUSG00000036381	1.65	2.09357E-08
ENSMUSG00000024349	1.65	0.00010341
ENSMUSG00000043263	1.65	0.011634554
ENSMUSG00000074469	1.64	0.007983154
ENSMUSG00000050022	1.64	0.001922915
ENSMUSG00000032344	1.64	0.000263304
ENSMUSG00000048612	1.64	0.003267398
ENSMUSG00000039601	1.64	1.54681E-08
ENSMUSG00000002983	1.64	8.14951E-05
ENSMUSG00000002257	1.64	0.016296442
ENSMUSG00000020437	1.64	0.002137212
ENSMUSG00000026821	1.64	0.011711886
ENSMUSG00000062488	1.63	0.00634998
ENSMUSG00000054342	1.63	0.002954565
ENSMUSG00000042759	1.63	0.004902425
ENSMUSG00000020407	1.63	2.61408E-06
ENSMUSG00000026728	1.63	0.000713629
ENSMUSG00000078771	1.63	0.002910366
ENSMUSG00000030774	1.63	6.13617E-06
ENSMUSG00000058715	1.63	0.000123397
ENSMUSG00000028071	1.62	0.030625218
ENSMUSG00000025165	1.62	2.36378E-07
ENSMUSG00000035208	1.62	0.001013075
ENSMUSG00000087107	1.62	0.00661913
ENSMUSG00000060989	1.62	0.016232519
ENSMUSG00000018774	1.62	1.49161E-05
ENSMUSG00000024399	1.62	0.000852961
ENSMUSG00000019737	1.61	0.003457994
ENSMUSG00000071715	1.61	0.000676606
ENSMUSG00000034028	1.61	0.003095309
ENSMUSG00000097415	1.61	0.006215373
ENSMUSG00000026395	1.61	0.005638414

Ensembl gene id	log2FC	p-adjusted
ENSMUSG00000025586	1.61	4.52779E-05
ENSMUSG00000008318	1.61	0.001562128
ENSMUSG00000087450	1.60	0.04289972
ENSMUSG00000044811	1.60	9.7598E-05
ENSMUSG00000015340	1.60	0.004827133
ENSMUSG00000052142	1.60	3.86672E-05
ENSMUSG00000021886	1.60	0.01778737
ENSMUSG00000033031	1.60	0.008473699
ENSMUSG00000079523	1.60	0.001495502
ENSMUSG00000027843	1.60	0.04339245
ENSMUSG00000030579	1.60	2.09357E-08
ENSMUSG00000034591	1.60	7.02551E-06
ENSMUSG00000043015	1.59	0.026820193
ENSMUSG00000022489	1.59	0.011661454
ENSMUSG00000026355	1.59	0.005187497
ENSMUSG00000055546	1.59	2.17971E-06
ENSMUSG00000024737	1.59	1.44716E-05
ENSMUSG00000056220	1.59	0.012226739
ENSMUSG00000033538	1.59	0.000396971
ENSMUSG00000020974	1.59	0.003267398
ENSMUSG00000030651	1.58	0.010932612
ENSMUSG00000056394	1.58	0.004144198
ENSMUSG00000031712	1.58	3.04064E-05
ENSMUSG00000034906	1.58	0.018332423
ENSMUSG00000062753	1.58	0.000155737
ENSMUSG00000085873	1.58	0.002753538
ENSMUSG00000032691	1.58	0.005231992
ENSMUSG00000049744	1.58	0.006555599
ENSMUSG00000073409	1.58	1.87686E-05
ENSMUSG00000038264	1.58	0.038907702
ENSMUSG00000031103	1.58	2.11275E-05
ENSMUSG00000046223	1.57	0.00033624
ENSMUSG00000026548	1.57	0.000133493
ENSMUSG00000034330	1.57	7.85995E-08
ENSMUSG00000075010	1.57	2.96124E-05
ENSMUSG00000026580	1.57	0.00120902
ENSMUSG00000040061	1.57	0.000137363
ENSMUSG00000042041	1.56	8.27768E-07
ENSMUSG00000078780	1.56	0.010369652
ENSMUSG00000029910	1.56	6.44534E-08
ENSMUSG00000030137	1.56	0.033303243
ENSMUSG00000026480	1.56	0.001139542
ENSMUSG00000034255	1.56	7.27821E-06
ENSMUSG00000079442	1.56	0.017120021
ENSMUSG00000097039	1.56	0.001657991
ENSMUSG00000024696	1.56	0.004030724
ENSMUSG00000052477	1.56	0.01890983
ENSMUSG00000015396	1.56	0.001940709
ENSMUSG00000024660	1.56	0.011913153
ENSMUSG00000028832	1.56	0.005408471
ENSMUSG00000026360	1.55	0.000127002
ENSMUSG00000039232	1.55	0.007007791

Ensembl gene id	log2FC	p-adjusted
ENSMUSG00000076928	1.55	0.01467934
ENSMUSG00000021280	1.55	0.010627012
ENSMUSG00000042842	1.55	0.005692194
ENSMUSG00000026274	1.55	0.002074303
ENSMUSG00000025498	1.55	0.00089301
ENSMUSG00000051225	1.55	0.010191078
ENSMUSG00000038304	1.55	0.017137817
ENSMUSG00000022488	1.54	0.00018323
ENSMUSG00000038151	1.54	0.011134633
ENSMUSG00000042474	1.54	0.006335903
ENSMUSG00000020143	1.54	5.21148E-05
ENSMUSG00000037405	1.54	2.05367E-05
ENSMUSG00000033450	1.54	0.018755802
ENSMUSG00000093661	1.54	0.000991004
ENSMUSG00000023032	1.53	0.002813364
ENSMUSG00000049307	1.53	0.0245136
ENSMUSG00000027544	1.53	0.008451956
ENSMUSG00000024795	1.53	0.003363282
ENSMUSG00000057191	1.52	0.014860676
ENSMUSG00000026829	1.52	0.009334524
ENSMUSG00000036777	1.52	0.001164224
ENSMUSG00000037313	1.52	0.016156369
ENSMUSG00000022831	1.52	0.000312216
ENSMUSG00000031262	1.52	0.027975009
ENSMUSG00000079339	1.52	0.007715622
ENSMUSG00000052160	1.52	0.000361218
ENSMUSG00000015950	1.52	3.64503E-06
ENSMUSG00000048922	1.52	0.012250873
ENSMUSG00000074874	1.51	0.005554087
ENSMUSG00000024965	1.51	0.002704001
ENSMUSG00000037820	1.51	2.94196E-22
ENSMUSG00000032094	1.51	0.002876721
ENSMUSG00000076757	1.51	0.006441393
ENSMUSG00000078606	1.51	0.005742831
ENSMUSG00000033952	1.51	0.000189746
ENSMUSG00000022876	1.51	0.008389609
ENSMUSG00000040747	1.51	0.000880185
ENSMUSG00000082433	1.50	0.007218245
ENSMUSG00000027490	1.50	0.000900967
ENSMUSG00000061758	1.50	6.17293E-05
ENSMUSG00000043017	1.50	0.031458653
ENSMUSG00000036905	1.50	2.42217E-07
ENSMUSG00000030187	1.50	0.00524925
ENSMUSG00000031904	1.50	0.010242407
ENSMUSG00000042351	1.50	0.027379575
ENSMUSG00000020330	1.49	0.011150103
ENSMUSG00000050410	1.49	0.015422663
ENSMUSG00000069515	1.49	0.006347156
ENSMUSG00000031165	1.49	0.002298412
ENSMUSG00000034612	1.49	0.022918044
ENSMUSG00000047534	1.49	0.012288441
ENSMUSG00000040675	1.49	0.000794326

Ensembl gene id	log2FC	p-adjusted
ENSMUSG00000032436	1.49	3.65358E-07
ENSMUSG00000039264	1.48	0.003862947
ENSMUSG00000069516	1.48	0.002902064
ENSMUSG00000049037	1.48	0.01659219
ENSMUSG00000025758	1.48	0.000123397
ENSMUSG00000001025	1.48	4.781E-05
ENSMUSG00000029553	1.48	0.003726665
ENSMUSG00000027907	1.48	0.005929098
ENSMUSG00000043157	1.48	0.013884558
ENSMUSG00000030528	1.47	0.004600678
ENSMUSG00000020077	1.47	8.54128E-05
ENSMUSG00000019850	1.47	0.000999368
ENSMUSG00000030798	1.47	0.001487704
ENSMUSG00000036006	1.47	0.006770339
ENSMUSG00000037572	1.47	0.023923819
ENSMUSG00000040345	1.47	0.000618146
ENSMUSG00000061132	1.47	0.004640399
ENSMUSG00000010021	1.47	0.025155716
ENSMUSG00000025352	1.47	0.004669456
ENSMUSG00000004446	1.47	0.014421053
ENSMUSG00000021640	1.46	0.022641588
ENSMUSG00000031765	1.46	1.38356E-11
ENSMUSG00000075122	1.46	0.007738497
ENSMUSG00000028555	1.46	4.70333E-13
ENSMUSG00000010342	1.46	0.00470023
ENSMUSG00000020638	1.46	0.000421954
ENSMUSG00000032294	1.46	0.004002815
ENSMUSG00000001281	1.46	0.00372001
ENSMUSG00000000028	1.45	0.000539652
ENSMUSG00000022148	1.45	0.002686963
ENSMUSG00000032113	1.45	0.007765357
ENSMUSG00000020388	1.45	1.82603E-05
ENSMUSG00000030745	1.45	0.000258037
ENSMUSG00000036896	1.45	2.01247E-08
ENSMUSG00000013707	1.45	0.014973163
ENSMUSG00000081769	1.45	0.007570855
ENSMUSG00000028581	1.45	0.000123733
ENSMUSG00000034593	1.45	0.002875181
ENSMUSG00000036894	1.45	0.000436192
ENSMUSG00000050232	1.45	0.001506555
ENSMUSG00000075611	1.44	0.037674126
ENSMUSG00000038807	1.44	9.11194E-05
ENSMUSG00000036362	1.44	0.003354263
ENSMUSG00000039193	1.44	0.011911578
ENSMUSG00000040711	1.44	0.001319885
ENSMUSG00000009585	1.44	8.08688E-07
ENSMUSG00000024044	1.44	0.002589976
ENSMUSG00000026177	1.44	0.00066735
ENSMUSG00000040990	1.43	0.006400807
ENSMUSG00000033192	1.43	0.00540926
ENSMUSG00000054293	1.43	0.015730783
ENSMUSG00000020493	1.43	0.008486819

Ensembl gene id	log2FC	p-adjusted
ENSMUSG00000052384	1.43	3.99358E-06
ENSMUSG00000020788	1.43	0.004999085
ENSMUSG00000031934	1.43	0.005455739
ENSMUSG00000047712	1.42	0.000364246
ENSMUSG00000031494	1.42	0.024796053
ENSMUSG00000044092	1.42	0.004771214
ENSMUSG00000098112	1.42	0.005626073
ENSMUSG00000030336	1.42	0.009202716
ENSMUSG00000029352	1.42	0.002666956
ENSMUSG00000057596	1.42	0.007338544
ENSMUSG00000024691	1.42	0.000128574
ENSMUSG00000016206	1.41	1.53347E-08
ENSMUSG00000032596	1.41	3.3229E-09
ENSMUSG00000060044	1.41	1.57378E-06
ENSMUSG00000021965	1.41	0.006762652
ENSMUSG00000070501	1.40	0.006154614
ENSMUSG00000045932	1.40	0.001383551
ENSMUSG00000031756	1.40	0.021854775
ENSMUSG00000068335	1.40	0.007921891
ENSMUSG00000026321	1.40	0.005156178
ENSMUSG00000033777	1.40	0.001654698
ENSMUSG00000029925	1.40	0.000180066
ENSMUSG00000046157	1.40	0.000892616
ENSMUSG00000040528	1.39	0.003886794
ENSMUSG00000015854	1.39	1.37594E-08
ENSMUSG00000034795	1.39	0.029334831
ENSMUSG00000030047	1.39	0.008444996
ENSMUSG00000033685	1.39	0.000196787
ENSMUSG00000028843	1.39	2.79662E-05
ENSMUSG00000020901	1.39	0.013712082
ENSMUSG00000048865	1.39	0.000121123
ENSMUSG00000040212	1.38	3.00176E-07
ENSMUSG00000022180	1.38	8.92588E-06
ENSMUSG00000037318	1.38	0.038198255
ENSMUSG00000036469	1.38	0.002666956
ENSMUSG00000049685	1.38	2.69815E-06
ENSMUSG00000021569	1.38	0.025895066
ENSMUSG00000037816	1.38	0.000145397
ENSMUSG00000052776	1.38	0.001323476
ENSMUSG00000024590	1.38	0.001774689
ENSMUSG00000037202	1.38	0.019088217
ENSMUSG00000024339	1.37	1.08259E-06
ENSMUSG00000035448	1.37	0.003116564
ENSMUSG00000031391	1.37	0.028731574
ENSMUSG00000050721	1.37	0.000327642
ENSMUSG00000038644	1.37	5.19767E-06
ENSMUSG00000036887	1.37	2.28795E-11
ENSMUSG00000011256	1.37	0.002612953
ENSMUSG00000038608	1.37	0.002869141
ENSMUSG00000048163	1.37	0.00669197
ENSMUSG00000026288	1.37	0.000371772
ENSMUSG00000030223	1.37	0.005452817

Ensembl gene id	log2FC	p-adjusted
ENSMUSG00000099065	1.37	0.006094334
ENSMUSG00000022014	1.37	0.026062104
ENSMUSG00000057789	1.36	4.47435E-14
ENSMUSG00000031659	1.36	0.000589728
ENSMUSG00000052688	1.36	0.006347156
ENSMUSG00000037902	1.36	0.007334594
ENSMUSG00000034422	1.35	6.33449E-06
ENSMUSG00000069792	1.35	1.21096E-12
ENSMUSG00000036931	1.35	0.03240165
ENSMUSG00000021196	1.35	1.2446E-06
ENSMUSG00000028364	1.35	0.000216741
ENSMUSG00000056529	1.35	0.009146165
ENSMUSG00000066861	1.35	0.000890107
ENSMUSG00000045551	1.35	0.001008447
ENSMUSG00000020649	1.35	0.008272415
ENSMUSG00000062210	1.35	8.5933E-07
ENSMUSG00000021457	1.35	0.004175568
ENSMUSG00000005763	1.35	0.023392249
ENSMUSG00000008193	1.35	0.002053028
ENSMUSG00000038252	1.34	0.002497296
ENSMUSG00000006310	1.34	0.015980378
ENSMUSG00000005547	1.34	2.36338E-06
ENSMUSG00000058163	1.34	0.008757883
ENSMUSG00000000732	1.34	0.01677341
ENSMUSG00000041420	1.34	0.025740981
ENSMUSG00000030188	1.34	0.000985667
ENSMUSG00000026622	1.34	8.97671E-05
ENSMUSG00000020886	1.34	0.037947644
ENSMUSG00000068220	1.34	0.008213276
ENSMUSG00000051439	1.34	0.000478616
ENSMUSG00000017830	1.34	3.00112E-05
ENSMUSG00000037679	1.33	0.000491988
ENSMUSG00000022440	1.33	0.000415762
ENSMUSG00000027641	1.33	0.011245075
ENSMUSG00000001270	1.33	0.001288258
ENSMUSG00000029570	1.33	0.000419417
ENSMUSG00000024155	1.33	0.013024743
ENSMUSG00000044250	1.33	0.000183909
ENSMUSG00000091705	1.33	0.038994421
ENSMUSG00000035004	1.33	0.012246475
ENSMUSG00000052889	1.32	3.52226E-05
ENSMUSG00000002458	1.32	0.008738272
ENSMUSG00000048498	1.32	1.59743E-06
ENSMUSG00000024521	1.32	0.003319935
ENSMUSG00000018920	1.31	0.000349015
ENSMUSG00000032089	1.31	0.006257373
ENSMUSG00000048779	1.31	1.00421E-06
ENSMUSG00000025602	1.30	0.005599983
ENSMUSG00000023505	1.30	0.004558702
ENSMUSG00000027330	1.30	2.03421E-05
ENSMUSG00000029484	1.30	0.007151825
ENSMUSG00000057346	1.29	7.84316E-06

Ensembl gene id	log2FC	p-adjusted
ENSMUSG00000028059	1.29	0.000147066
ENSMUSG00000047878	1.29	0.007704418
ENSMUSG00000000628	1.29	0.010196423
ENSMUSG00000068245	1.29	0.000650959
ENSMUSG00000040710	1.29	0.0384031
ENSMUSG00000022436	1.29	0.005052765
ENSMUSG00000080917	1.29	0.016833195
ENSMUSG00000058818	1.29	0.000665107
ENSMUSG00000042029	1.28	0.007420006
ENSMUSG00000023947	1.28	0.004212881
ENSMUSG00000043004	1.28	0.00713072
ENSMUSG00000030263	1.28	0.004816751
ENSMUSG00000028028	1.27	6.3026E-06
ENSMUSG00000063458	1.27	0.013226731
ENSMUSG00000031778	1.27	0.022809651
ENSMUSG00000042684	1.27	8.70842E-05
ENSMUSG00000029521	1.27	0.007486744
ENSMUSG00000031903	1.26	1.07078E-08
ENSMUSG00000053113	1.26	7.65511E-11
ENSMUSG00000020641	1.26	1.19004E-05
ENSMUSG00000022346	1.26	1.01496E-07
ENSMUSG00000000244	1.26	0.025593871
ENSMUSG00000035275	1.26	0.034709683
ENSMUSG00000021998	1.26	0.000696693
ENSMUSG00000079017	1.26	0.0179087
ENSMUSG00000042190	1.25	0.001624164
ENSMUSG00000009292	1.25	6.55966E-07
ENSMUSG00000031389	1.25	5.88641E-07
ENSMUSG00000027955	1.25	0.003190483
ENSMUSG00000026979	1.25	9.66549E-06
ENSMUSG00000087150	1.25	0.006155795
ENSMUSG00000020878	1.25	0.006204714
ENSMUSG00000029798	1.25	0.000123397
ENSMUSG00000021728	1.25	0.043424544
ENSMUSG00000024308	1.25	8.63644E-06
ENSMUSG00000037972	1.25	0.000493905
ENSMUSG00000036390	1.25	6.18385E-06
ENSMUSG00000037613	1.24	0.010278271
ENSMUSG00000001128	1.24	0.000197948
ENSMUSG00000051682	1.24	0.008283773
ENSMUSG00000031112	1.24	0.019001732
ENSMUSG00000055994	1.24	0.005395633
ENSMUSG00000027293	1.24	0.000183909
ENSMUSG00000027078	1.24	0.015568393
ENSMUSG00000004730	1.24	8.45277E-06
ENSMUSG00000002602	1.23	0.000226845
ENSMUSG00000024621	1.23	2.73181E-06
ENSMUSG00000055805	1.22	0.004555782
ENSMUSG00000015843	1.22	2.36344E-07
ENSMUSG00000030641	1.22	0.017145337
ENSMUSG00000006403	1.22	0.016578301
ENSMUSG00000046245	1.22	0.024736578

Ensembl gene id	log2FC	p-adjusted
ENSMUSG00000045827	1.22	0.000665107
ENSMUSG00000032020	1.22	0.006823758
ENSMUSG00000006360	1.22	0.000488458
ENSMUSG00000027134	1.21	0.01849416
ENSMUSG00000020658	1.21	0.004451874
ENSMUSG00000038811	1.21	0.005113107
ENSMUSG00000018507	1.21	0.028403873
ENSMUSG00000034837	1.21	6.3465E-09
ENSMUSG00000042333	1.21	5.32625E-06
ENSMUSG00000034595	1.21	0.00010341
ENSMUSG00000052248	1.21	0.019442916
ENSMUSG00000026628	1.20	5.16531E-09
ENSMUSG00000031093	1.20	0.0349063
ENSMUSG00000007041	1.20	7.23415E-06
ENSMUSG00000019823	1.20	0.007336165
ENSMUSG00000011884	1.20	9.83109E-09
ENSMUSG00000024030	1.20	0.000565172
ENSMUSG00000031557	1.20	0.001668458
ENSMUSG00000056116	1.19	7.27821E-06
ENSMUSG00000032577	1.19	0.001712598
ENSMUSG00000000489	1.19	0.044513191
ENSMUSG00000028359	1.19	0.000227442
ENSMUSG00000034652	1.19	0.001195698
ENSMUSG00000037166	1.19	0.005683275
ENSMUSG00000085977	1.19	0.026426363
ENSMUSG00000045404	1.19	0.008283773
ENSMUSG00000029581	1.19	3.33176E-07
ENSMUSG00000047945	1.19	1.02139E-07
ENSMUSG00000022432	1.19	0.006079883
ENSMUSG00000028337	1.19	0.000772491
ENSMUSG00000029330	1.19	0.014317616
ENSMUSG00000063268	1.19	6.38915E-13
ENSMUSG00000022474	1.19	0.008736993
ENSMUSG00000045751	1.19	0.002928376
ENSMUSG00000001473	1.19	2.76246E-09
ENSMUSG00000096472	1.18	0.003856746
ENSMUSG00000024742	1.18	0.01456476
ENSMUSG00000070323	1.18	0.005430877
ENSMUSG00000001506	1.18	1.36997E-09
ENSMUSG00000015568	1.18	0.035465343
ENSMUSG00000044350	1.18	2.83091E-11
ENSMUSG00000079197	1.18	8.8332E-15
ENSMUSG00000059791	1.18	0.012008296
ENSMUSG00000030587	1.18	0.024526032
ENSMUSG00000037031	1.18	0.003439454
ENSMUSG00000028859	1.17	0.012127161
ENSMUSG00000034413	1.17	0.005450193
ENSMUSG00000079465	1.17	0.020450583
ENSMUSG00000002944	1.17	3.90988E-11
ENSMUSG00000051506	1.17	1.20038E-05
ENSMUSG00000026547	1.17	1.94436E-07
ENSMUSG00000029366	1.17	0.007621117

Ensembl gene id	log2FC	p-adjusted
ENSMUSG00000055612	1.17	2.05367E-05
ENSMUSG00000001417	1.17	1.66578E-10
ENSMUSG00000079491	1.17	2.48508E-05
ENSMUSG00000022378	1.17	9.85614E-05
ENSMUSG00000029919	1.17	0.004816751
ENSMUSG00000023277	1.17	0.001372316
ENSMUSG00000028238	1.17	4.29941E-11
ENSMUSG00000019866	1.17	0.000507924
ENSMUSG00000004266	1.17	8.74046E-05
ENSMUSG00000022952	1.17	0.007921891
ENSMUSG00000047250	1.17	1.90453E-05
ENSMUSG00000059498	1.16	4.13912E-05
ENSMUSG00000030536	1.16	0.000908606
ENSMUSG00000027454	1.16	0.00343763
ENSMUSG00000039747	1.16	0.014327505
ENSMUSG00000078685	1.16	0.005440394
ENSMUSG00000024824	1.16	0.000126254
ENSMUSG00000037447	1.16	0.000214188
ENSMUSG00000060550	1.15	0.00360835
ENSMUSG00000031827	1.15	6.62934E-05
ENSMUSG00000030844	1.15	0.014968266
ENSMUSG00000024556	1.15	5.20925E-06
ENSMUSG00000003153	1.15	0.043424544
ENSMUSG00000031906	1.15	0.006889498
ENSMUSG00000019874	1.15	1.51615E-07
ENSMUSG00000070003	1.14	1.61082E-05
ENSMUSG00000025887	1.14	0.000482412
ENSMUSG00000026972	1.14	0.00044499
ENSMUSG00000030921	1.14	0.00086717
ENSMUSG00000026110	1.14	0.011881721
ENSMUSG00000042349	1.14	1.0506E-10
ENSMUSG00000038507	1.14	7.46774E-09
ENSMUSG00000068758	1.14	0.003691031
ENSMUSG00000028671	1.13	3.21877E-12
ENSMUSG00000025743	1.13	2.59594E-06
ENSMUSG00000063286	1.13	0.005297054
ENSMUSG00000020589	1.13	0.007922222
ENSMUSG00000026421	1.12	0.003562852
ENSMUSG00000036181	1.12	0.007031787
ENSMUSG00000023827	1.12	0.031558248
ENSMUSG00000057933	1.12	0.029491998
ENSMUSG00000023043	1.12	1.01424E-14
ENSMUSG00000053158	1.12	1.22904E-07
ENSMUSG00000068246	1.12	2.56279E-06
ENSMUSG00000036908	1.12	1.09483E-11
ENSMUSG00000029007	1.12	9.69292E-05
ENSMUSG00000001750	1.12	1.01553E-12
ENSMUSG00000024066	1.12	4.99077E-14
ENSMUSG00000054720	1.11	0.010119194
ENSMUSG00000034329	1.11	0.0061888
ENSMUSG00000031207	1.11	0.000143764
ENSMUSG00000041488	1.11	0.000204341

Ensembl gene id	log2FC	p-adjusted
ENSMUSG00000025287	1.11	0.003348
ENSMUSG00000073490	1.11	7.69099E-05
ENSMUSG00000025044	1.11	3.72089E-05
ENSMUSG00000049866	1.11	0.001068353
ENSMUSG00000031805	1.11	7.87801E-09
ENSMUSG00000034765	1.11	0.037075747
ENSMUSG00000054640	1.11	0.002823267
ENSMUSG00000016495	1.10	5.40864E-06
ENSMUSG00000050592	1.10	0.000226845
ENSMUSG00000026358	1.10	0.014830572
ENSMUSG00000022901	1.10	0.000194896
ENSMUSG00000037020	1.10	0.005096846
ENSMUSG00000040616	1.10	0.002970806
ENSMUSG00000040522	1.10	0.005966505
ENSMUSG00000010307	1.10	0.000168674
ENSMUSG00000003355	1.10	0.031394717
ENSMUSG00000038034	1.10	7.40749E-06
ENSMUSG00000020415	1.09	0.007475392
ENSMUSG00000056888	1.09	0.043453428
ENSMUSG00000031639	1.09	0.000253555
ENSMUSG00000022369	1.09	0.005110097
ENSMUSG00000035095	1.09	0.00664331
ENSMUSG00000024014	1.09	1.17207E-06
ENSMUSG00000022216	1.09	1.48249E-07
ENSMUSG00000048621	1.09	0.004941774
ENSMUSG00000069662	1.08	0.000883884
ENSMUSG00000037280	1.08	0.003609047
ENSMUSG00000025014	1.08	0.014860676
ENSMUSG00000018381	1.08	0.0245136
ENSMUSG00000006715	1.08	0.003245784
ENSMUSG00000079014	1.08	6.20312E-05
ENSMUSG00000027009	1.08	3.13019E-07
ENSMUSG00000020573	1.08	0.025106605
ENSMUSG00000078942	1.08	0.010278271
ENSMUSG00000037419	1.08	1.01175E-05
ENSMUSG00000019810	1.07	2.42792E-07
ENSMUSG00000005667	1.07	0.016571051
ENSMUSG00000057948	1.07	0.002573714
ENSMUSG00000007080	1.07	0.010102096
ENSMUSG00000027398	1.06	0.010037036
ENSMUSG00000037921	1.06	0.002957427
ENSMUSG00000070327	1.06	0.000246523
ENSMUSG00000038301	1.06	0.000282029
ENSMUSG00000071203	1.06	0.005273431
ENSMUSG00000054435	1.06	0.002910366
ENSMUSG00000020227	1.06	0.024690886
ENSMUSG00000033970	1.06	0.020734842
ENSMUSG00000025232	1.06	3.72177E-08
ENSMUSG00000030254	1.05	0.043945576
ENSMUSG00000027399	1.05	0.000404736
ENSMUSG00000062012	1.05	0.035455018
ENSMUSG00000014599	1.05	4.30218E-10

Ensembl gene id	log2FC	p-adjusted
ENSMUSG00000029326	1.05	0.000252047
ENSMUSG00000027322	1.05	2.03476E-08
ENSMUSG00000078234	1.05	7.01648E-10
ENSMUSG00000001588	1.04	0.001029125
ENSMUSG00000025647	1.04	5.47261E-08
ENSMUSG00000030966	1.04	6.86945E-08
ENSMUSG00000026070	1.04	0.025813897
ENSMUSG00000098557	1.04	4.52779E-05
ENSMUSG00000041642	1.04	0.000532128
ENSMUSG00000026980	1.03	8.68985E-05
ENSMUSG00000041112	1.03	0.009245691
ENSMUSG00000022360	1.03	0.043424544
ENSMUSG00000091867	1.03	0.004165089
ENSMUSG00000040829	1.03	0.005258195
ENSMUSG00000027555	1.03	0.035167829
ENSMUSG00000036986	1.03	0.001150723
ENSMUSG00000037095	1.02	2.21669E-12
ENSMUSG00000029771	1.02	1.42575E-08
ENSMUSG00000032698	1.02	0.015392137
ENSMUSG00000022181	1.02	2.40645E-05
ENSMUSG00000028339	1.02	0.001799229
ENSMUSG00000017631	1.02	0.025878252
ENSMUSG00000030403	1.02	0.00021742
ENSMUSG00000042726	1.02	0.002354058
ENSMUSG00000019876	1.02	0.009419065
ENSMUSG00000026946	1.01	1.47294E-08
ENSMUSG00000036249	1.01	0.000109802
ENSMUSG00000015745	1.01	0.000141192
ENSMUSG00000043008	1.01	0.023180685
ENSMUSG00000039621	1.01	0.002711013
ENSMUSG00000028645	1.01	5.796E-05
ENSMUSG00000027901	1.01	4.91732E-05
ENSMUSG00000021725	1.01	0.013954788
ENSMUSG00000029174	1.00	4.4028E-06
ENSMUSG00000028212	1.00	0.035510949
ENSMUSG00000039158	1.00	0.019323953
ENSMUSG00000027408	1.00	0.001474246
ENSMUSG00000001918	1.00	0.002116786
ENSMUSG00000029101	0.96	0.035197252
ENSMUSG00000030159	0.96	0.003980786
ENSMUSG00000090019	0.95	0.008757883
ENSMUSG00000026222	0.88	0.008491268
ENSMUSG00000041449	0.85	1.44223E-05
ENSMUSG00000039997	0.84	0.02284896
ENSMUSG00000024851	0.84	0.003892423
ENSMUSG00000051495	-1.00	0.00045572
ENSMUSG00000047446	-1.00	4.55261E-06
ENSMUSG00000032238	-1.00	2.23641E-08
ENSMUSG00000039046	-1.01	5.81386E-11
ENSMUSG00000048489	-1.01	0.027202093
ENSMUSG00000059908	-1.01	4.29315E-13
ENSMUSG00000025019	-1.01	1.04699E-07

Ensembl gene id	log2FC	p-adjusted
ENSMUSG00000046794	-1.02	0.005447784
ENSMUSG00000038128	-1.02	0.000181054
ENSMUSG00000028476	-1.03	0.000324522
ENSMUSG00000041324	-1.05	0.019068825
ENSMUSG00000027660	-1.06	4.99788E-11
ENSMUSG00000030257	-1.06	0.014973163
ENSMUSG00000067336	-1.06	3.99229E-08
ENSMUSG00000048271	-1.06	2.40828E-05
ENSMUSG00000025004	-1.06	4.39248E-11
ENSMUSG00000030771	-1.06	0.001419962
ENSMUSG00000067279	-1.07	6.00789E-11
ENSMUSG00000021690	-1.08	1.06555E-10
ENSMUSG00000020883	-1.08	8.81993E-11
ENSMUSG00000067225	-1.08	0.00599777
ENSMUSG00000019947	-1.08	0.000855708
ENSMUSG00000024042	-1.09	3.04225E-07
ENSMUSG00000048856	-1.09	0.003295874
ENSMUSG00000021127	-1.10	1.17615E-06
ENSMUSG00000030787	-1.10	0.004717245
ENSMUSG00000030554	-1.10	0.000307254
ENSMUSG00000031665	-1.10	5.48599E-14
ENSMUSG00000048732	-1.10	0.004524596
ENSMUSG00000027684	-1.11	0.003187101
ENSMUSG00000023963	-1.11	0.028060347
ENSMUSG00000029512	-1.12	4.08218E-14
ENSMUSG00000074925	-1.12	0.000539123
ENSMUSG00000066154	-1.13	1.21828E-13
ENSMUSG00000030731	-1.13	0.004886341
ENSMUSG00000029385	-1.13	5.74319E-06
ENSMUSG00000034584	-1.14	0.000711453
ENSMUSG00000026185	-1.14	3.84598E-08
ENSMUSG00000024298	-1.14	6.29259E-11
ENSMUSG00000087579	-1.14	3.34792E-09
ENSMUSG00000032599	-1.16	2.14402E-06
ENSMUSG00000064347	-1.17	0.002037733
ENSMUSG00000025880	-1.17	3.1444E-07
ENSMUSG00000048087	-1.17	5.2462E-09
ENSMUSG00000001670	-1.17	6.82183E-06
ENSMUSG00000030246	-1.17	6.02865E-05
ENSMUSG00000058207	-1.18	7.05399E-18
ENSMUSG00000052684	-1.18	0.000565172
ENSMUSG00000037617	-1.19	0.015586992
ENSMUSG00000024843	-1.19	3.96362E-08
ENSMUSG00000066361	-1.19	1.04273E-17
ENSMUSG00000034936	-1.19	2.04284E-09
ENSMUSG00000022358	-1.19	0.003245784
ENSMUSG00000071204	-1.19	5.19039E-09
ENSMUSG00000051000	-1.20	0.000962624
ENSMUSG00000024190	-1.20	0.000813735
ENSMUSG00000097766	-1.20	0.009196431
ENSMUSG00000063730	-1.20	0.000196716
ENSMUSG00000086682	-1.21	0.014039155

Ensembl gene id	log2FC	p-adjusted
ENSMUSG00000024544	-1.21	0.000319884
ENSMUSG00000033863	-1.21	3.0383E-14
ENSMUSG00000072999	-1.22	0.003605559
ENSMUSG00000029151	-1.22	0.00646122
ENSMUSG00000056749	-1.22	4.47435E-14
ENSMUSG00000060600	-1.23	0.00098125
ENSMUSG00000020027	-1.23	6.01915E-05
ENSMUSG00000056313	-1.23	2.1915E-10
ENSMUSG00000020034	-1.24	1.19235E-08
ENSMUSG00000055561	-1.24	0.016178204
ENSMUSG00000099032	-1.24	0.01019608
ENSMUSG00000051335	-1.25	0.000122899
ENSMUSG00000026207	-1.26	0.004482433
ENSMUSG00000054178	-1.26	0.000106983
ENSMUSG00000057722	-1.28	0.024660621
ENSMUSG00000092400	-1.29	2.36312E-07
ENSMUSG00000026475	-1.29	6.60768E-08
ENSMUSG00000097691	-1.29	0.001839423
ENSMUSG00000064349	-1.29	0.000101703
ENSMUSG00000017817	-1.30	0.020530545
ENSMUSG00000044646	-1.30	0.000733199
ENSMUSG00000059327	-1.30	0.025083954
ENSMUSG00000041482	-1.30	0.000907007
ENSMUSG00000062901	-1.31	4.36128E-14
ENSMUSG00000044807	-1.33	0.039465618
ENSMUSG00000039004	-1.33	2.26839E-05
ENSMUSG00000064344	-1.33	0.000852961
ENSMUSG00000041624	-1.33	0.001861187
ENSMUSG00000002289	-1.34	0.002102084
ENSMUSG00000026610	-1.34	6.83836E-05
ENSMUSG00000072949	-1.35	0.025970041
ENSMUSG00000097417	-1.36	0.010836905
ENSMUSG00000019232	-1.36	6.72705E-14
ENSMUSG00000015090	-1.36	0.040389088
ENSMUSG00000030945	-1.37	0.004390687
ENSMUSG00000091405	-1.37	0.007175621
ENSMUSG00000041550	-1.37	0.011620606
ENSMUSG00000056666	-1.37	2.60447E-14
ENSMUSG00000027513	-1.37	1.02281E-23
ENSMUSG00000025321	-1.38	0.016171446
ENSMUSG00000063929	-1.39	0.014181631
ENSMUSG00000029009	-1.39	6.91866E-08
ENSMUSG00000043013	-1.39	0.003251368
ENSMUSG00000041044	-1.40	3.82004E-11
ENSMUSG00000043418	-1.41	0.000154422
ENSMUSG00000060560	-1.41	0.036545782
ENSMUSG00000018427	-1.41	0.00040406
ENSMUSG00000020038	-1.42	8.21664E-17
ENSMUSG00000074218	-1.43	0.016571051
ENSMUSG00000034295	-1.43	0.036514191
ENSMUSG00000002265	-1.44	0.010050655
ENSMUSG00000072294	-1.44	3.91015E-12

Ensembl gene id	log2FC	p-adjusted
ENSMUSG00000028967	-1.46	0.000586927
ENSMUSG00000002250	-1.47	0.000229256
ENSMUSG00000031770	-1.49	3.27682E-06
ENSMUSG00000097148	-1.52	0.011696818
ENSMUSG00000064179	-1.53	0.002444885
ENSMUSG00000055116	-1.53	1.08403E-15
ENSMUSG00000074075	-1.54	0.016144062
ENSMUSG00000098867	-1.54	0.012446141
ENSMUSG00000086231	-1.54	2.74961E-07
ENSMUSG00000024600	-1.55	0.047901758
ENSMUSG00000019935	-1.55	0.033055447
ENSMUSG00000098741	-1.55	0.015178442
ENSMUSG00000022519	-1.56	2.45405E-07
ENSMUSG00000001095	-1.56	0.011710905
ENSMUSG00000001027	-1.56	0.016251737
ENSMUSG00000031286	-1.58	1.81869E-09
ENSMUSG00000045441	-1.58	5.22402E-16
ENSMUSG00000091971	-1.58	2.11782E-05
ENSMUSG00000018566	-1.61	0.000123623
ENSMUSG00000027313	-1.61	0.002955836
ENSMUSG00000030401	-1.62	0.013624616
ENSMUSG00000038894	-1.64	3.09676E-20
ENSMUSG00000028519	-1.67	0.000182082
ENSMUSG00000022003	-1.68	8.26979E-15
ENSMUSG00000033107	-1.69	1.57422E-25
ENSMUSG00000035112	-1.72	0.000386183
ENSMUSG00000020431	-1.72	1.15829E-07
ENSMUSG00000019577	-1.72	0.015737251
ENSMUSG00000068699	-1.74	6.85888E-06
ENSMUSG00000018581	-1.74	0.000407543
ENSMUSG00000048583	-1.74	0.006441393
ENSMUSG00000031791	-1.75	1.50532E-07
ENSMUSG00000026950	-1.76	4.92771E-24
ENSMUSG00000043461	-1.76	0.017227073
ENSMUSG00000086165	-1.78	0.000516205
ENSMUSG00000037406	-1.79	0.036976108
ENSMUSG00000049985	-1.80	7.33958E-26
ENSMUSG00000085963	-1.81	1.24192E-06
ENSMUSG00000016239	-1.85	1.68588E-07
ENSMUSG00000095079	-1.86	0.023058023
ENSMUSG00000028655	-1.87	8.93635E-08
ENSMUSG00000027861	-1.87	0.002154177
ENSMUSG00000020593	-1.87	0.004155249
ENSMUSG00000041536	-1.87	0.00775926
ENSMUSG00000000031	-1.88	2.38267E-06
ENSMUSG00000041930	-1.89	8.46166E-10
ENSMUSG00000027796	-1.90	4.81944E-06
ENSMUSG00000037139	-1.90	0.000913547
ENSMUSG00000007097	-1.91	5.91635E-11
ENSMUSG00000025272	-1.92	0.046014045
ENSMUSG00000078650	-1.92	3.46388E-26
ENSMUSG00000032845	-1.93	0.037998875

Ensembl gene id	log2FC	p-adjusted
ENSMUSG00000042686	-1.94	0.012289897
ENSMUSG00000041596	-1.95	6.52283E-06
ENSMUSG00000041559	-1.96	0.007717812
ENSMUSG00000085811	-1.96	0.039783825
ENSMUSG00000024049	-1.97	2.05995E-08
ENSMUSG00000089827	-1.97	0.0045976
ENSMUSG00000046480	-1.97	0.001094695
ENSMUSG00000020893	-1.98	7.85463E-16
ENSMUSG00000066687	-2.00	4.46203E-20
ENSMUSG00000040666	-2.01	0.0245136
ENSMUSG00000066705	-2.02	0.000532447
ENSMUSG00000027871	-2.03	0.000114707
ENSMUSG00000026407	-2.03	8.20119E-07
ENSMUSG00000082364	-2.04	0.011307003
ENSMUSG00000090264	-2.07	2.63856E-11
ENSMUSG00000020429	-2.07	3.61609E-15
ENSMUSG00000020475	-2.07	1.00102E-05
ENSMUSG00000028464	-2.09	2.89569E-15
ENSMUSG00000021373	-2.11	0.007060641
ENSMUSG00000040612	-2.12	3.43106E-05
ENSMUSG00000023044	-2.13	9.19438E-26
ENSMUSG00000030827	-2.14	0.017169431
ENSMUSG00000085834	-2.15	4.92233E-09
ENSMUSG00000038917	-2.17	0.045867873
ENSMUSG00000047746	-2.25	0.000746628
ENSMUSG00000032648	-2.25	8.5428E-08
ENSMUSG00000026077	-2.29	1.72003E-21
ENSMUSG00000074569	-2.34	0.028036749
ENSMUSG00000021798	-2.36	2.15309E-09
ENSMUSG00000049641	-2.42	0.037563994
ENSMUSG00000026817	-2.43	2.48971E-07
ENSMUSG00000097810	-2.43	0.03224904
ENSMUSG00000035923	-2.50	0.028582768
ENSMUSG00000020122	-2.51	6.1262E-09
ENSMUSG00000030785	-2.52	2.21061E-08
ENSMUSG00000028773	-2.54	1.29649E-05
ENSMUSG00000067653	-2.56	7.45898E-31
ENSMUSG00000042895	-2.57	0.044702711
ENSMUSG00000029607	-2.58	0.030791815
ENSMUSG00000025488	-2.59	0.002506635
ENSMUSG00000078492	-2.63	0.044324692
ENSMUSG00000039376	-2.64	0.003457994
ENSMUSG00000038763	-2.64	2.96124E-05
ENSMUSG00000083777	-2.66	0.016681026
ENSMUSG00000006457	-2.70	8.74046E-05
ENSMUSG00000028328	-2.72	0.026100515
ENSMUSG00000006221	-2.73	1.37905E-11
ENSMUSG00000039891	-2.75	8.80681E-09
ENSMUSG00000002500	-2.76	1.59518E-05
ENSMUSG00000072720	-2.81	2.98858E-08
ENSMUSG00000038201	-2.83	0.029469566
ENSMUSG00000026418	-2.83	0.000778885

Ensembl gene id	log2FC	p-adjusted
ENSMUSG00000040752	-2.84	0.00298814
ENSMUSG00000001508	-2.87	0.005985348
ENSMUSG00000030592	-2.90	3.91333E-13
ENSMUSG00000028715	-2.90	1.35402E-20
ENSMUSG00000020836	-2.91	9.45264E-05
ENSMUSG00000008658	-2.98	0.039951272
ENSMUSG00000078486	-2.99	3.5523E-09
ENSMUSG00000040694	-3.03	0.044436316
ENSMUSG00000079588	-3.05	0.004377679
ENSMUSG00000049134	-3.05	8.8332E-15
ENSMUSG00000027868	-3.05	0.00433524
ENSMUSG00000081603	-3.13	0.012858704
ENSMUSG00000007877	-3.14	1.7537E-15
ENSMUSG00000007122	-3.16	2.31796E-09
ENSMUSG00000030996	-3.16	0.000669968
ENSMUSG00000042828	-3.17	0.000670709
ENSMUSG00000030672	-3.17	9.4935E-18
ENSMUSG00000038670	-3.18	0.000766293
ENSMUSG00000042717	-3.22	0.000453305
ENSMUSG00000031461	-3.25	0.003324883
ENSMUSG00000020067	-3.25	0.000312216
ENSMUSG00000029869	-3.26	8.81912E-07
ENSMUSG00000053093	-3.27	3.113E-16
ENSMUSG00000090066	-3.31	4.19832E-06
ENSMUSG00000041476	-3.39	0.000101306
ENSMUSG00000019787	-3.41	4.81137E-09
ENSMUSG00000075307	-3.47	1.36023E-09
ENSMUSG00000090105	-3.49	0.018478306
ENSMUSG00000027470	-3.53	0.000586927
ENSMUSG00000030399	-3.53	4.09562E-12
ENSMUSG00000047419	-3.54	8.40294E-34
ENSMUSG00000031097	-3.56	3.62406E-17
ENSMUSG00000021622	-3.58	1.56073E-17
ENSMUSG00000031972	-3.58	1.87496E-20
ENSMUSG00000033717	-3.58	0.035625247
ENSMUSG00000030730	-3.60	7.79863E-39
ENSMUSG00000061723	-3.60	5.9695E-36
ENSMUSG00000024471	-3.60	7.38004E-18
ENSMUSG00000016349	-3.64	4.13646E-19
ENSMUSG00000061816	-3.64	2.1685E-24
ENSMUSG00000061462	-3.65	4.29017E-43
ENSMUSG00000051906	-3.66	0.001104872
ENSMUSG00000028834	-3.66	0.007721522
ENSMUSG00000071540	-3.66	1.10678E-07
ENSMUSG00000021768	-3.67	0.03870672
ENSMUSG00000020216	-3.68	0.000882996
ENSMUSG00000056328	-3.70	5.61681E-16
ENSMUSG00000029158	-3.70	0.03770578
ENSMUSG00000044951	-3.70	0.000123397
ENSMUSG00000013936	-3.71	6.02873E-06
ENSMUSG00000027022	-3.71	2.8383E-31
ENSMUSG00000005716	-3.73	3.53062E-12

Ensembl gene id	log2FC	p-adjusted
ENSMUSG00000038239	-3.74	4.36219E-09
ENSMUSG00000055775	-3.76	0.041580236
ENSMUSG00000051497	-3.79	1.04521E-06
ENSMUSG00000028116	-3.83	2.21478E-05
ENSMUSG00000033196	-3.83	1.45825E-10
ENSMUSG00000051747	-3.87	4.56439E-45
ENSMUSG00000018893	-3.90	3.20243E-53
ENSMUSG00000052374	-3.91	2.80946E-25
ENSMUSG00000027887	-3.93	1.18281E-05
ENSMUSG00000062077	-4.03	0.009402935
ENSMUSG00000092075	-4.05	8.71314E-08
ENSMUSG00000020061	-4.05	5.46019E-44
ENSMUSG00000092240	-4.09	0.007991508
ENSMUSG00000017300	-4.12	3.49511E-09
ENSMUSG00000044938	-4.20	1.28511E-05
ENSMUSG00000042045	-4.26	0.00011956
ENSMUSG00000020354	-4.27	0.002412286
ENSMUSG00000035296	-4.67	0.017887966
ENSMUSG00000029683	-4.68	0.000153415
ENSMUSG00000033044	-4.81	0.010432308
ENSMUSG00000073375	-4.83	0.010150942
ENSMUSG00000027077	-4.90	7.1477E-08
ENSMUSG00000068697	-4.92	7.11107E-08
ENSMUSG00000070385	-5.08	3.32346E-06
ENSMUSG00000074001	-5.19	0.00172312
ENSMUSG00000059668	-6.52	0.038318064
**Sm_PZQ_46**		
ENSMUSG00000063779	8.97	3.94579E-88
ENSMUSG00000009185	7.20	5.66173E-07
ENSMUSG00000050370	6.87	2.02713E-10
ENSMUSG00000040809	6.83	1.69946E-05
ENSMUSG00000061100	6.61	4.32314E-08
ENSMUSG00000047222	6.59	0.000267514
ENSMUSG00000022157	6.43	0.047745949
ENSMUSG00000087642	6.43	0.021274678
ENSMUSG00000022651	5.70	1.02499E-07
ENSMUSG00000027073	5.51	0.015861033
ENSMUSG00000052435	5.40	0.002852846
ENSMUSG00000001131	5.29	5.93523E-18
ENSMUSG00000028415	5.16	1.70239E-06
ENSMUSG00000040197	5.04	3.65539E-05
ENSMUSG00000095170	4.90	0.005134722
ENSMUSG00000078521	4.89	0.001165217
ENSMUSG00000091478	4.78	0.044823743
ENSMUSG00000035186	4.72	3.06325E-18
ENSMUSG00000035373	4.64	3.02466E-07
ENSMUSG00000041831	4.50	0.021966189
ENSMUSG00000022422	4.41	4.12584E-06
ENSMUSG00000016498	4.40	2.97503E-09
ENSMUSG00000031780	4.34	0.032765387
ENSMUSG00000001228	4.29	9.33989E-05
ENSMUSG00000018500	4.26	0.001136095

Ensembl gene id	log2FC	p-adjusted
ENSMUSG00000053338	4.24	0.004973579
ENSMUSG00000082976	4.24	0.048678835
ENSMUSG00000028072	4.24	0.001383491
ENSMUSG00000058755	4.24	0.001449913
ENSMUSG00000018924	4.22	1.54295E-11
ENSMUSG00000068606	4.21	5.48528E-06
ENSMUSG00000020581	4.14	0.026691123
ENSMUSG00000024028	4.13	0.03778126
ENSMUSG00000034883	4.04	0.002709656
ENSMUSG00000057465	4.02	6.53957E-05
ENSMUSG00000022479	4.01	0.000319568
ENSMUSG00000046006	3.98	1.01835E-06
ENSMUSG00000091345	3.88	0.000737592
ENSMUSG00000020676	3.87	2.39899E-11
ENSMUSG00000028587	3.85	0.002545517
ENSMUSG00000024791	3.84	4.66948E-07
ENSMUSG00000038379	3.80	0.000884997
ENSMUSG00000094335	3.77	0.042700862
ENSMUSG00000042489	3.76	4.51189E-05
ENSMUSG00000003484	3.75	1.52583E-06
ENSMUSG00000076614	3.75	0.019230386
ENSMUSG00000004791	3.75	0.00285194
ENSMUSG00000031162	3.74	0.018666678
ENSMUSG00000017499	3.74	0.001038401
ENSMUSG00000068452	3.72	0.018023149
ENSMUSG00000051314	3.68	0.022089934
ENSMUSG00000072596	3.67	0.029540372
ENSMUSG00000062345	3.66	6.34998E-05
ENSMUSG00000031933	3.63	0.000498808
ENSMUSG00000030346	3.59	0.004125138
ENSMUSG00000083019	3.58	0.045337478
ENSMUSG00000019890	3.57	0.048845059
ENSMUSG00000021758	3.56	0.005754676
ENSMUSG00000027323	3.52	4.70721E-05
ENSMUSG00000037466	3.49	8.54071E-06
ENSMUSG00000032484	3.41	0.037051271
ENSMUSG00000027335	3.39	0.012227255
ENSMUSG00000039748	3.37	2.97931E-05
ENSMUSG00000038357	3.37	0.034334929
ENSMUSG00000023908	3.37	4.47855E-16
ENSMUSG00000076596	3.36	0.030842381
ENSMUSG00000036223	3.36	0.000165286
ENSMUSG00000030111	3.35	0.039202898
ENSMUSG00000024989	3.35	6.37749E-10
ENSMUSG00000046341	3.33	4.58069E-09
ENSMUSG00000074115	3.32	3.99644E-05
ENSMUSG00000046591	3.32	0.001637801
ENSMUSG00000000805	3.31	0.000665178
ENSMUSG00000032586	3.31	1.38987E-05
ENSMUSG00000005410	3.30	0.000424629
ENSMUSG00000022033	3.29	0.000151306
ENSMUSG00000050578	3.29	1.38996E-09

Ensembl gene id	log2FC	p-adjusted
ENSMUSG00000027656	3.27	4.08398E-05
ENSMUSG00000040950	3.26	1.37756E-12
ENSMUSG00000000903	3.24	3.08595E-05
ENSMUSG00000015134	3.22	3.78084E-06
ENSMUSG00000024529	3.21	0.000527542
ENSMUSG00000015880	3.21	0.000412542
ENSMUSG00000063903	3.21	0.029472487
ENSMUSG00000030413	3.20	0.000671293
ENSMUSG00000098318	3.19	0.029805496
ENSMUSG00000033213	3.17	0.005376229
ENSMUSG00000022322	3.17	3.09331E-05
ENSMUSG00000035455	3.16	0.001126491
ENSMUSG00000029414	3.15	0.033524999
ENSMUSG00000028965	3.14	0.011111374
ENSMUSG00000005470	3.13	2.71959E-05
ENSMUSG00000021953	3.12	0.006927617
ENSMUSG00000064147	3.12	0.002780903
ENSMUSG00000004814	3.12	0.000628104
ENSMUSG00000022034	3.11	0.008115793
ENSMUSG00000035352	3.11	0.004074317
ENSMUSG00000026011	3.10	0.004158733
ENSMUSG00000002835	3.10	0.002545517
ENSMUSG00000050410	3.09	1.24955E-05
ENSMUSG00000063388	3.08	3.37788E-33
ENSMUSG00000084796	3.07	2.52841E-05
ENSMUSG00000044303	3.07	0.01539292
ENSMUSG00000037991	3.06	0.001534465
ENSMUSG00000087213	3.05	0.006373888
ENSMUSG00000040231	3.04	0.004902597
ENSMUSG00000020826	3.02	0.002218396
ENSMUSG00000023992	3.02	8.1121E-05
ENSMUSG00000019942	3.01	3.85843E-06
ENSMUSG00000026039	3.01	4.5E-05
ENSMUSG00000045273	3.01	0.000174535
ENSMUSG00000020787	3.01	0.010123089
ENSMUSG00000020383	3.00	0.010828116
ENSMUSG00000025473	2.99	1.03207E-06
ENSMUSG00000036322	2.99	6.2266E-16
ENSMUSG00000039013	2.96	1.18215E-05
ENSMUSG00000026355	2.96	0.002629457
ENSMUSG00000020914	2.96	8.89994E-07
ENSMUSG00000031262	2.96	3.21863E-05
ENSMUSG00000072980	2.95	0.010672432
ENSMUSG00000032496	2.95	0.048473360
ENSMUSG00000040152	2.95	4.80628E-08
ENSMUSG00000026196	2.94	3.6305E-06
ENSMUSG00000025001	2.92	0.014192783
ENSMUSG00000040026	2.91	7.3507E-13
ENSMUSG00000021298	2.90	0.004009798
ENSMUSG00000069910	2.90	0.002391333
ENSMUSG00000000028	2.89	4.5E-05
ENSMUSG00000054999	2.87	0.016824178

Ensembl gene id	log2FC	p-adjusted
ENSMUSG00000043903	2.87	0.016853506
ENSMUSG00000024402	2.87	0.017347309
ENSMUSG00000027490	2.87	0.000209956
ENSMUSG00000095488	2.86	0.040857682
ENSMUSG00000019214	2.86	7.78691E-06
ENSMUSG00000032783	2.86	0.00109215
ENSMUSG00000021697	2.85	0.002784036
ENSMUSG00000021062	2.85	0.012701616
ENSMUSG00000030543	2.85	0.003740111
ENSMUSG00000034773	2.84	1.45551E-05
ENSMUSG00000023940	2.84	0.000127993
ENSMUSG00000037725	2.83	0.000900792
ENSMUSG00000076652	2.83	0.041087895
ENSMUSG00000026683	2.82	0.000107765
ENSMUSG00000038037	2.81	1.51374E-09
ENSMUSG00000094749	2.79	0.040340148
ENSMUSG00000068696	2.79	0.004920552
ENSMUSG00000026582	2.79	0.000449817
ENSMUSG00000000983	2.78	0.010242869
ENSMUSG00000030641	2.77	0.015706793
ENSMUSG00000020897	2.77	8.32451E-10
ENSMUSG00000068129	2.76	0.001117012
ENSMUSG00000006585	2.76	0.00144125
ENSMUSG00000073705	2.76	1.61733E-06
ENSMUSG00000037474	2.76	0.003260605
ENSMUSG00000028832	2.74	7.28148E-13
ENSMUSG00000000982	2.74	0.005018872
ENSMUSG00000027408	2.73	6.32771E-05
ENSMUSG00000048191	2.72	0.035395777
ENSMUSG00000056071	2.72	0.028918358
ENSMUSG00000044309	2.71	2.28818E-05
ENSMUSG00000091938	2.70	0.03058403
ENSMUSG00000030789	2.70	1.15022E-05
ENSMUSG00000041859	2.69	0.0001517
ENSMUSG00000035165	2.67	0.008619853
ENSMUSG00000024670	2.67	2.72302E-06
ENSMUSG00000023067	2.67	0.028118716
ENSMUSG00000030589	2.67	2.29676E-05
ENSMUSG00000031495	2.65	0.01007763
ENSMUSG00000041202	2.64	2.70353E-08
ENSMUSG00000032815	2.64	0.015242757
ENSMUSG00000032113	2.63	0.037972495
ENSMUSG00000034906	2.62	0.000247976
ENSMUSG00000001506	2.61	2.30311E-06
ENSMUSG00000056394	2.59	6.58827E-06
ENSMUSG00000039187	2.59	0.000628104
ENSMUSG00000076586	2.59	0.006470495
ENSMUSG00000002055	2.59	7.75785E-07
ENSMUSG00000018930	2.57	6.74621E-05
ENSMUSG00000030162	2.57	0.048447749
ENSMUSG00000050107	2.56	6.00005E-05
ENSMUSG00000022456	2.55	0.002412534

Ensembl gene id	log2FC	p-adjusted
ENSMUSG00000034634	2.55	5.72151E-06
ENSMUSG00000004267	2.54	0.001515588
ENSMUSG00000041481	2.54	0.011909643
ENSMUSG00000026630	2.54	0.00034606
ENSMUSG00000049723	2.54	3.52389E-07
ENSMUSG00000061540	2.53	6.2101E-06
ENSMUSG00000085708	2.52	0.034874596
ENSMUSG00000034023	2.52	0.037454155
ENSMUSG00000051727	2.51	0.034910393
ENSMUSG00000031821	2.51	0.009358027
ENSMUSG00000019992	2.50	0.002856884
ENSMUSG00000031629	2.50	0.018163294
ENSMUSG00000034394	2.50	0.040016018
ENSMUSG00000000562	2.49	0.021359611
ENSMUSG00000020649	2.48	0.013880034
ENSMUSG00000026622	2.48	5.85555E-07
ENSMUSG00000028702	2.47	5.85555E-07
ENSMUSG00000031494	2.47	4.99408E-05
ENSMUSG00000026822	2.46	3.22071E-06
ENSMUSG00000037868	2.45	0.000247304
ENSMUSG00000032400	2.44	0.000781886
ENSMUSG00000030677	2.44	8.2874E-10
ENSMUSG00000006398	2.43	2.36126E-11
ENSMUSG00000060586	2.42	3.79797E-16
ENSMUSG00000022385	2.41	0.013307634
ENSMUSG00000021485	2.39	0.002716929
ENSMUSG00000031779	2.39	0.000553093
ENSMUSG00000079553	2.39	5.32008E-06
ENSMUSG00000027797	2.39	4.89071E-05
ENSMUSG00000021714	2.39	0.003648421
ENSMUSG00000098090	2.39	0.000443562
ENSMUSG00000029417	2.38	3.72575E-10
ENSMUSG00000025804	2.38	0.004612457
ENSMUSG00000002870	2.37	0.00412691
ENSMUSG00000021384	2.36	0.038472779
ENSMUSG00000026429	2.35	0.000433151
ENSMUSG00000022945	2.34	0.000343749
ENSMUSG00000037544	2.34	0.000208973
ENSMUSG00000039055	2.33	0.003550494
ENSMUSG00000034570	2.33	0.016807054
ENSMUSG00000009350	2.33	0.047795666
ENSMUSG00000036587	2.31	0.007697252
ENSMUSG00000027715	2.31	1.62513E-13
ENSMUSG00000024056	2.31	2.29459E-05
ENSMUSG00000064246	2.30	0.000143668
ENSMUSG00000026069	2.30	0.001997933
ENSMUSG00000027379	2.29	0.000146761
ENSMUSG00000033036	2.29	0.01946555
ENSMUSG00000031289	2.29	0.00731428
ENSMUSG00000034413	2.29	7.03878E-05
ENSMUSG00000095771	2.28	0.043486442
ENSMUSG00000025701	2.28	0.001297213

Ensembl gene id	log2FC	p-adjusted
ENSMUSG00000035683	2.27	2.73007E-07
ENSMUSG00000036768	2.27	4.45607E-06
ENSMUSG00000063646	2.27	0.01028774
ENSMUSG00000079523	2.26	0.000800825
ENSMUSG00000037913	2.26	0.025543325
ENSMUSG00000044505	2.25	1.18834E-06
ENSMUSG00000033847	2.24	0.011771631
ENSMUSG00000020241	2.24	5.26378E-07
ENSMUSG00000021208	2.24	0.013677213
ENSMUSG00000031762	2.24	0.015969737
ENSMUSG00000026274	2.24	0.001094378
ENSMUSG00000030742	2.24	4.69806E-07
ENSMUSG00000051378	2.23	0.00372317
ENSMUSG00000029816	2.23	3.32324E-07
ENSMUSG00000053318	2.23	2.38479E-05
ENSMUSG00000025912	2.22	0.002984323
ENSMUSG00000066170	2.22	0.013131097
ENSMUSG00000032322	2.22	5.42772E-06
ENSMUSG00000089929	2.21	0.000768071
ENSMUSG00000024353	2.20	0.041653032
ENSMUSG00000045751	2.19	0.004570528
ENSMUSG00000079547	2.19	5.94777E-22
ENSMUSG00000093861	2.19	0.023932597
ENSMUSG00000029283	2.18	1.69946E-05
ENSMUSG00000038295	2.18	0.024235262
ENSMUSG00000028718	2.17	0.007928759
ENSMUSG00000027811	2.16	0.002984323
ENSMUSG00000021175	2.16	0.020679831
ENSMUSG00000015316	2.16	0.038900793
ENSMUSG00000021822	2.15	4.58069E-09
ENSMUSG00000028068	2.15	6.90101E-05
ENSMUSG00000024742	2.15	0.00323613
ENSMUSG00000035385	2.15	6.62627E-06
ENSMUSG00000024669	2.15	0.000136933
ENSMUSG00000068105	2.14	0.000628104
ENSMUSG00000001119	2.14	9.766E-10
ENSMUSG00000056054	2.13	0.038059849
ENSMUSG00000035365	2.13	0.007386442
ENSMUSG00000049103	2.13	0.000247976
ENSMUSG00000031506	2.12	1.18345E-06
ENSMUSG00000048922	2.12	1.18215E-05
ENSMUSG00000021569	2.12	0.003707092
ENSMUSG00000076937	2.12	8.3575E-08
ENSMUSG00000046295	2.11	0.006779351
ENSMUSG00000073421	2.11	1.01587E-16
ENSMUSG00000031756	2.10	0.007573979
ENSMUSG00000025351	2.08	0.000110635
ENSMUSG00000015312	2.07	0.003037385
ENSMUSG00000034855	2.07	1.64429E-20
ENSMUSG00000021453	2.07	6.43855E-06
ENSMUSG00000062510	2.06	0.002458065
ENSMUSG00000015619	2.05	0.000695776

Ensembl gene id	log2FC	p-adjusted
ENSMUSG00000000682	2.05	3.47738E-13
ENSMUSG00000040592	2.05	1.15645E-07
ENSMUSG00000037628	2.04	0.038787748
ENSMUSG00000067608	2.03	3.21044E-09
ENSMUSG00000021263	2.03	0.000124679
ENSMUSG00000038943	2.02	1.8906E-08
ENSMUSG00000076617	2.02	0.026913336
ENSMUSG00000037887	2.01	4.72283E-06
ENSMUSG00000015314	2.01	0.000236197
ENSMUSG00000050335	2.01	5.78917E-15
ENSMUSG00000028873	2.00	1.46134E-08
ENSMUSG00000022673	2.00	0.013085244
ENSMUSG00000040751	1.99	2.03725E-06
ENSMUSG00000092200	1.99	0.005592943
ENSMUSG00000021965	1.99	0.002275072
ENSMUSG00000027635	1.99	5.65461E-08
ENSMUSG00000000318	1.98	0.021057629
ENSMUSG00000010142	1.98	6.58827E-06
ENSMUSG00000053862	1.98	0.005227096
ENSMUSG00000033707	1.98	0.001040627
ENSMUSG00000001025	1.98	0.0010273
ENSMUSG00000071005	1.96	0.004149179
ENSMUSG00000051220	1.96	0.045119569
ENSMUSG00000029061	1.96	0.001079039
ENSMUSG00000014030	1.96	0.000870636
ENSMUSG00000048327	1.96	4.90076E-06
ENSMUSG00000030004	1.96	0.026839135
ENSMUSG00000018927	1.96	0.000221389
ENSMUSG00000029730	1.96	0.001845293
ENSMUSG00000078773	1.96	0.000488413
ENSMUSG00000028884	1.96	0.002583498
ENSMUSG00000027469	1.95	2.41355E-08
ENSMUSG00000021986	1.95	0.001476228
ENSMUSG00000058290	1.95	0.000185027
ENSMUSG00000073409	1.94	4.35415E-27
ENSMUSG00000097364	1.94	0.039363
ENSMUSG00000027454	1.94	1.70497E-05
ENSMUSG00000051517	1.94	0.00148505
ENSMUSG00000020798	1.94	0.018917956
ENSMUSG00000039774	1.93	0.032017153
ENSMUSG00000026043	1.93	8.93101E-05
ENSMUSG00000001403	1.93	1.27242E-07
ENSMUSG00000038264	1.93	0.004533268
ENSMUSG00000001020	1.93	3.15105E-07
ENSMUSG00000041431	1.92	0.026053821
ENSMUSG00000030510	1.91	0.044538561
ENSMUSG00000056737	1.91	5.08197E-09
ENSMUSG00000017716	1.91	4.80985E-10
ENSMUSG00000023015	1.90	1.0985E-08
ENSMUSG00000037313	1.90	2.71475E-11
ENSMUSG00000030867	1.89	0.001069762
ENSMUSG00000025161	1.89	1.4315E-05

Ensembl gene id	log2FC	p-adjusted
ENSMUSG00000091898	1.89	5.91324E-05
ENSMUSG00000034311	1.88	1.38759E-05
ENSMUSG00000034206	1.88	0.016108420
ENSMUSG00000024334	1.87	0.000695231
ENSMUSG00000042029	1.87	0.014670678
ENSMUSG00000091694	1.87	0.033605514
ENSMUSG00000020974	1.86	0.001789473
ENSMUSG00000022584	1.86	0.025644406
ENSMUSG00000034591	1.86	4.95179E-22
ENSMUSG00000004709	1.86	0.003401897
ENSMUSG00000046223	1.86	0.000172737
ENSMUSG00000022881	1.86	0.010050684
ENSMUSG00000044702	1.86	0.000611932
ENSMUSG00000027368	1.86	0.003597433
ENSMUSG00000030745	1.85	0.000126137
ENSMUSG00000027654	1.85	0.030629094
ENSMUSG00000026875	1.84	0.000443562
ENSMUSG00000075602	1.83	1.76322E-16
ENSMUSG00000045826	1.83	1.75309E-07
ENSMUSG00000037337	1.83	1.06737E-05
ENSMUSG00000040907	1.83	0.004097039
ENSMUSG00000027331	1.83	1.06189E-07
ENSMUSG00000076609	1.83	0.018744613
ENSMUSG00000024610	1.83	8.88232E-19
ENSMUSG00000040441	1.82	1.44479E-07
ENSMUSG00000032011	1.82	1.15917E-05
ENSMUSG00000059791	1.82	1.92415E-06
ENSMUSG00000059323	1.81	0.004840778
ENSMUSG00000037649	1.81	8.44168E-13
ENSMUSG00000038508	1.80	0.000209956
ENSMUSG00000078922	1.80	1.18432E-15
ENSMUSG00000021268	1.80	0.005670526
ENSMUSG00000001517	1.79	7.43426E-05
ENSMUSG00000029581	1.79	1.60451E-14
ENSMUSG00000082902	1.79	0.016324339
ENSMUSG00000017652	1.79	1.4846E-05
ENSMUSG00000022876	1.78	9.52039E-06
ENSMUSG00000036594	1.78	1.96982E-17
ENSMUSG00000029661	1.77	7.74127E-05
ENSMUSG00000002068	1.77	0.021619464
ENSMUSG00000041801	1.77	0.004189902
ENSMUSG00000023505	1.77	1.15645E-07
ENSMUSG00000024672	1.76	0.001299309
ENSMUSG00000020279	1.76	0.014282464
ENSMUSG00000030254	1.76	0.008439768
ENSMUSG00000039109	1.76	0.004911583
ENSMUSG00000070645	1.76	0.031432232
ENSMUSG00000028111	1.76	0.046902754
ENSMUSG00000030707	1.76	9.37497E-11
ENSMUSG00000068220	1.75	3.66791E-05
ENSMUSG00000076498	1.75	0.00160904
ENSMUSG00000025395	1.75	0.00363638

Ensembl gene id	log2FC	p-adjusted
ENSMUSG00000073412	1.74	3.45874E-07
ENSMUSG00000016283	1.74	0.000536326
ENSMUSG00000005540	1.73	1.88023E-08
ENSMUSG00000005233	1.73	0.000414978
ENSMUSG00000031740	1.73	0.011120008
ENSMUSG00000018819	1.73	1.80555E-14
ENSMUSG00000001555	1.72	0.000489274
ENSMUSG00000004612	1.72	1.4407E-05
ENSMUSG00000069793	1.72	0.014475836
ENSMUSG00000034317	1.72	2.00113E-05
ENSMUSG00000028270	1.71	3.27655E-20
ENSMUSG00000082292	1.71	4.56238E-13
ENSMUSG00000032271	1.71	0.020930839
ENSMUSG00000035448	1.71	1.01137E-08
ENSMUSG00000074476	1.71	4.35099E-05
ENSMUSG00000057058	1.70	0.001606663
ENSMUSG00000047139	1.70	9.04358E-10
ENSMUSG00000019845	1.70	0.000929614
ENSMUSG00000013974	1.70	0.018061402
ENSMUSG00000037548	1.70	1.92592E-09
ENSMUSG00000028678	1.70	0.003956078
ENSMUSG00000022021	1.70	0.006320772
ENSMUSG00000078921	1.70	1.5266E-07
ENSMUSG00000027750	1.69	1.34411E-05
ENSMUSG00000031497	1.69	0.009112151
ENSMUSG00000008193	1.69	8.05556E-07
ENSMUSG00000033453	1.69	0.000605437
ENSMUSG00000048534	1.69	0.002136583
ENSMUSG00000052477	1.69	0.036482825
ENSMUSG00000074676	1.69	0.018073397
ENSMUSG00000001281	1.69	2.09983E-11
ENSMUSG00000096768	1.69	0.004598033
ENSMUSG00000003051	1.68	7.73086E-05
ENSMUSG00000085156	1.68	1.46286E-05
ENSMUSG00000021880	1.68	0.001277229
ENSMUSG00000096422	1.68	0.021903655
ENSMUSG00000052087	1.67	0.002161557
ENSMUSG00000092021	1.67	6.5962E-07
ENSMUSG00000042834	1.67	0.000261234
ENSMUSG00000006360	1.66	8.76414E-06
ENSMUSG00000046031	1.66	9.8176E-07
ENSMUSG00000024338	1.66	5.19663E-21
ENSMUSG00000049580	1.66	0.00180213
ENSMUSG00000024245	1.66	0.013885767
ENSMUSG00000029204	1.65	1.97061E-06
ENSMUSG00000026628	1.65	4.92704E-10
ENSMUSG00000000486	1.64	5.22718E-07
ENSMUSG00000024301	1.64	0.004203255
ENSMUSG00000047757	1.64	0.031070468
ENSMUSG00000041406	1.64	0.006036716
ENSMUSG00000037572	1.64	0.046172027
ENSMUSG00000024399	1.64	3.58788E-08

Ensembl gene id	log2FC	p-adjusted
ENSMUSG00000022440	1.64	1.72077E-06
ENSMUSG00000035042	1.63	2.41355E-08
ENSMUSG00000028364	1.63	0.000799421
ENSMUSG00000021728	1.62	0.000119879
ENSMUSG00000003379	1.62	2.94429E-05
ENSMUSG00000024397	1.62	1.38654E-05
ENSMUSG00000040247	1.62	4.46044E-06
ENSMUSG00000022439	1.61	1.30121E-10
ENSMUSG00000013155	1.61	0.023213507
ENSMUSG00000060550	1.61	4.17902E-19
ENSMUSG00000070000	1.61	0.002524873
ENSMUSG00000041219	1.60	5.25083E-08
ENSMUSG00000096727	1.60	4.17902E-19
ENSMUSG00000032091	1.60	0.016080646
ENSMUSG00000017969	1.59	4.90391E-08
ENSMUSG00000038644	1.59	0.003824293
ENSMUSG00000032218	1.59	0.023654957
ENSMUSG00000038059	1.59	0.020948413
ENSMUSG00000003352	1.59	0.002850711
ENSMUSG00000024660	1.59	9.2278E-08
ENSMUSG00000027514	1.58	3.85867E-18
ENSMUSG00000032094	1.58	0.026232758
ENSMUSG00000002257	1.58	0.008228448
ENSMUSG00000017146	1.58	0.039294931
ENSMUSG00000025758	1.57	0.001094378
ENSMUSG00000093565	1.57	0.016192817
ENSMUSG00000061577	1.57	0.010302136
ENSMUSG00000026580	1.57	0.000997676
ENSMUSG00000000861	1.57	0.008506438
ENSMUSG00000028044	1.57	6.03883E-08
ENSMUSG00000030724	1.57	0.003905229
ENSMUSG00000030587	1.56	0.008639984
ENSMUSG00000030978	1.56	0.014750838
ENSMUSG00000095562	1.55	0.006390576
ENSMUSG00000020846	1.55	5.96343E-06
ENSMUSG00000067149	1.55	0.043283562
ENSMUSG00000006715	1.55	6.08142E-09
ENSMUSG00000068101	1.55	0.004651205
ENSMUSG00000059089	1.55	3.71945E-12
ENSMUSG00000003153	1.54	0.000677564
ENSMUSG00000051504	1.54	0.026185031
ENSMUSG00000075266	1.54	0.00038948
ENSMUSG00000024675	1.54	0.001514246
ENSMUSG00000008136	1.54	0.026740481
ENSMUSG00000033031	1.54	0.001042325
ENSMUSG00000056413	1.54	6.22436E-06
ENSMUSG00000007080	1.54	0.017391953
ENSMUSG00000038252	1.54	4.11685E-05
ENSMUSG00000005824	1.54	0.034790987
ENSMUSG00000020415	1.53	2.1352E-05
ENSMUSG00000026605	1.53	9.0399E-05
ENSMUSG00000094686	1.53	0.001788002

Ensembl gene id	log2FC	p-adjusted
ENSMUSG00000042254	1.53	0.001788002
ENSMUSG00000028268	1.53	2.98826E-16
ENSMUSG00000033220	1.53	5.16868E-14
ENSMUSG00000053977	1.52	0.0004815
ENSMUSG00000023132	1.52	0.000495141
ENSMUSG00000050022	1.52	3.32968E-06
ENSMUSG00000020437	1.52	1.29105E-08
ENSMUSG00000052142	1.52	4.91761E-06
ENSMUSG00000089672	1.52	8.2859E-07
ENSMUSG00000004933	1.52	0.003573156
ENSMUSG00000035929	1.51	9.57012E-07
ENSMUSG00000064109	1.51	0.005659495
ENSMUSG00000057092	1.51	0.018943643
ENSMUSG00000034786	1.51	4.50865E-07
ENSMUSG00000044827	1.51	0.002374624
ENSMUSG00000030468	1.51	0.000303405
ENSMUSG00000021811	1.51	0.002557194
ENSMUSG00000086859	1.51	1.11925E-07
ENSMUSG00000030798	1.51	3.63257E-10
ENSMUSG00000027239	1.51	0.011480719
ENSMUSG00000032053	1.50	2.11083E-05
ENSMUSG00000050014	1.50	0.023888461
ENSMUSG00000049775	1.49	1.77382E-08
ENSMUSG00000029373	1.49	0.008444305
ENSMUSG00000097445	1.49	0.015024245
ENSMUSG00000097102	1.49	0.042436719
ENSMUSG00000025058	1.48	0.023823235
ENSMUSG00000020695	1.48	0.001740852
ENSMUSG00000006519	1.48	1.11652E-13
ENSMUSG00000045763	1.47	0.000155644
ENSMUSG00000031765	1.47	0.018689002
ENSMUSG00000036672	1.47	1.71657E-05
ENSMUSG00000042474	1.46	0.000385955
ENSMUSG00000030208	1.46	0.000510895
ENSMUSG00000097296	1.46	0.013222826
ENSMUSG00000051998	1.45	0.004058303
ENSMUSG00000002204	1.45	0.002192097
ENSMUSG00000021508	1.44	0.016430566
ENSMUSG00000040204	1.44	0.004486028
ENSMUSG00000043439	1.44	9.8176E-07
ENSMUSG00000026271	1.44	4.64232E-05
ENSMUSG00000046908	1.44	0.016534358
ENSMUSG00000030220	1.44	2.39899E-11
ENSMUSG00000062591	1.44	0.016946860
ENSMUSG00000054342	1.44	0.008980961
ENSMUSG00000034266	1.44	0.004084733
ENSMUSG00000030793	1.43	0.001571105
ENSMUSG00000030124	1.43	0.003466543
ENSMUSG00000026134	1.43	0.002545517
ENSMUSG00000033952	1.43	0.002126787
ENSMUSG00000022489	1.43	0.000439097
ENSMUSG00000026728	1.42	1.50278E-15

Ensembl gene id	log2FC	p-adjusted
ENSMUSG00000052013	1.42	2.14781E-05
ENSMUSG00000034164	1.42	0.015721902
ENSMUSG00000044199	1.42	0.010806345
ENSMUSG00000098014	1.42	0.003480197
ENSMUSG00000026042	1.41	4.06529E-06
ENSMUSG00000003541	1.41	0.007140659
ENSMUSG00000031101	1.41	1.24493E-07
ENSMUSG00000053656	1.41	0.000520976
ENSMUSG00000068758	1.41	0.000102862
ENSMUSG00000045328	1.41	0.002160902
ENSMUSG00000023345	1.41	5.96471E-05
ENSMUSG00000027018	1.41	0.000282588
ENSMUSG00000078853	1.41	1.37743E-11
ENSMUSG00000054000	1.41	0.000851387
ENSMUSG00000031838	1.40	1.89236E-09
ENSMUSG00000079017	1.40	0.020862369
ENSMUSG00000040084	1.40	0.000552703
ENSMUSG00000070691	1.40	0.007540784
ENSMUSG00000020077	1.40	1.87384E-10
ENSMUSG00000029436	1.40	0.016647416
ENSMUSG00000027342	1.40	0.00081675
ENSMUSG00000027134	1.40	0.006339224
ENSMUSG00000040212	1.40	1.18345E-06
ENSMUSG00000035279	1.39	0.016006295
ENSMUSG00000057123	1.39	0.015953666
ENSMUSG00000052133	1.39	0.026461585
ENSMUSG00000027203	1.39	6.6257E-06
ENSMUSG00000008318	1.39	0.00684584
ENSMUSG00000011256	1.38	6.19875E-06
ENSMUSG00000030218	1.38	0.002399101
ENSMUSG00000022360	1.38	0.018018571
ENSMUSG00000019577	1.38	0.013802391
ENSMUSG00000021226	1.38	0.014488908
ENSMUSG00000047675	1.37	0.011461139
ENSMUSG00000032334	1.37	0.021966189
ENSMUSG00000044934	1.37	0.030247305
ENSMUSG00000026956	1.37	1.18411E-07
ENSMUSG00000002297	1.37	1.81887E-05
ENSMUSG00000028687	1.37	0.014465000
ENSMUSG00000039942	1.37	0.002578739
ENSMUSG00000005667	1.37	0.000577921
ENSMUSG00000034116	1.37	3.00068E-09
ENSMUSG00000022102	1.36	5.85158E-08
ENSMUSG00000028312	1.36	0.006058676
ENSMUSG00000051212	1.36	0.028659439
ENSMUSG00000047798	1.36	1.12112E-05
ENSMUSG00000031304	1.36	4.488E-07
ENSMUSG00000026126	1.36	5.36446E-05
ENSMUSG00000033970	1.36	4.81556E-06
ENSMUSG00000023905	1.36	9.78028E-07
ENSMUSG00000072949	1.35	0.021046343
ENSMUSG00000009687	1.35	1.30688E-09

Ensembl gene id	log2FC	p-adjusted
ENSMUSG00000024795	1.35	0.023021663
ENSMUSG00000018868	1.35	0.026449427
ENSMUSG00000054400	1.35	0.037983801
ENSMUSG00000042817	1.34	0.031210471
ENSMUSG00000055612	1.34	0.009542856
ENSMUSG00000035439	1.34	2.61806E-05
ENSMUSG00000015053	1.34	0.009725124
ENSMUSG00000041538	1.34	0.000172737
ENSMUSG00000000244	1.33	0.006284075
ENSMUSG00000022103	1.33	9.98165E-05
ENSMUSG00000032093	1.33	0.003313289
ENSMUSG00000011008	1.33	0.041161176
ENSMUSG00000056888	1.33	0.014845399
ENSMUSG00000051278	1.33	0.007297028
ENSMUSG00000037020	1.33	0.01093434
ENSMUSG00000028633	1.32	0.025851289
ENSMUSG00000028551	1.32	2.70684E-07
ENSMUSG00000027326	1.32	0.015102452
ENSMUSG00000030717	1.32	0.016223164
ENSMUSG00000042436	1.32	0.001179506
ENSMUSG00000028041	1.32	2.34611E-07
ENSMUSG00000042759	1.32	5.80796E-06
ENSMUSG00000052131	1.32	0.006558528
ENSMUSG00000062753	1.32	0.000910743
ENSMUSG00000027907	1.32	0.000103171
ENSMUSG00000036067	1.31	0.023461232
ENSMUSG00000032436	1.31	1.70239E-06
ENSMUSG00000023886	1.31	2.65634E-06
ENSMUSG00000021262	1.31	2.50773E-07
ENSMUSG00000012443	1.31	0.004614805
ENSMUSG00000079505	1.31	0.040610399
ENSMUSG00000031264	1.30	1.28445E-05
ENSMUSG00000022978	1.30	1.82828E-05
ENSMUSG00000027863	1.30	0.00297238
ENSMUSG00000099065	1.30	0.015561545
ENSMUSG00000027699	1.30	0.001838929
ENSMUSG00000028480	1.30	9.93251E-05
ENSMUSG00000031897	1.30	8.2874E-10
ENSMUSG00000087273	1.29	0.011883845
ENSMUSG00000027962	1.29	4.2529E-10
ENSMUSG00000020388	1.29	0.000129442
ENSMUSG00000074141	1.29	0.000146761
ENSMUSG00000046841	1.29	1.77287E-07
ENSMUSG00000028931	1.29	0.000527793
ENSMUSG00000038811	1.28	9.9487E-09
ENSMUSG00000010830	1.28	0.023054782
ENSMUSG00000061132	1.28	0.002911696
ENSMUSG00000048521	1.28	0.000333914
ENSMUSG00000039601	1.28	0.007259583
ENSMUSG00000026928	1.28	0.027104797
ENSMUSG00000046491	1.28	0.013365802
ENSMUSG00000025877	1.27	4.94221E-09

Ensembl gene id	log2FC	p-adjusted
ENSMUSG00000079018	1.27	0.000435664
ENSMUSG00000023827	1.27	0.014670678
ENSMUSG00000037820	1.27	7.71193E-12
ENSMUSG00000051735	1.26	2.52841E-05
ENSMUSG00000087107	1.26	5.40414E-06
ENSMUSG00000039994	1.26	0.019619557
ENSMUSG00000022436	1.26	1.12112E-05
ENSMUSG00000040675	1.26	0.000475167
ENSMUSG00000000409	1.26	6.32771E-05
ENSMUSG00000050212	1.26	0.000192806
ENSMUSG00000039396	1.26	0.008492244
ENSMUSG00000039264	1.26	0.001625649
ENSMUSG00000073411	1.26	6.67996E-11
ENSMUSG00000015396	1.26	0.000900068
ENSMUSG00000034192	1.25	7.56932E-06
ENSMUSG00000084274	1.25	0.03190278
ENSMUSG00000097415	1.25	0.001494797
ENSMUSG00000002033	1.25	0.000543004
ENSMUSG00000004952	1.25	1.12112E-05
ENSMUSG00000054717	1.25	0.002359259
ENSMUSG00000089726	1.24	0.000913524
ENSMUSG00000060063	1.24	2.61806E-05
ENSMUSG00000070803	1.24	0.007566214
ENSMUSG00000032786	1.24	0.043883514
ENSMUSG00000056498	1.23	0.031276234
ENSMUSG00000044533	1.23	0.000237787
ENSMUSG00000045382	1.23	0.002139538
ENSMUSG00000020878	1.23	0.008265556
ENSMUSG00000020788	1.23	1.60299E-06
ENSMUSG00000037348	1.22	3.91533E-12
ENSMUSG00000044811	1.22	0.001041341
ENSMUSG00000001270	1.22	1.22343E-05
ENSMUSG00000024965	1.22	1.95083E-08
ENSMUSG00000025002	1.22	0.029329998
ENSMUSG00000024349	1.22	7.17224E-06
ENSMUSG00000056612	1.22	1.22861E-06
ENSMUSG00000071068	1.22	0.01509434
ENSMUSG00000013707	1.22	0.000209266
ENSMUSG00000071714	1.22	1.12112E-05
ENSMUSG00000048779	1.22	1.41822E-05
ENSMUSG00000078439	1.22	0.045279858
ENSMUSG00000030148	1.22	0.002850711
ENSMUSG00000047878	1.22	0.006727993
ENSMUSG00000028010	1.21	1.86562E-05
ENSMUSG00000020057	1.21	0.000324617
ENSMUSG00000076928	1.21	0.048154493
ENSMUSG00000002233	1.21	9.17139E-08
ENSMUSG00000023004	1.21	0.000345073
ENSMUSG00000037166	1.20	0.013783552
ENSMUSG00000072082	1.20	3.85811E-05
ENSMUSG00000022048	1.20	3.55391E-05
ENSMUSG00000024910	1.20	0.012485215

Ensembl gene id	log2FC	p-adjusted
ENSMUSG00000004665	1.20	3.62584E-08
ENSMUSG00000026785	1.20	0.004533268
ENSMUSG00000028927	1.20	0.02742074
ENSMUSG00000022831	1.20	9.65016E-09
ENSMUSG00000076613	1.20	0.039619081
ENSMUSG00000056481	1.19	0.011873141
ENSMUSG00000026480	1.19	3.60445E-07
ENSMUSG00000048163	1.19	7.97809E-07
ENSMUSG00000031669	1.19	0.001656373
ENSMUSG00000032374	1.19	0.011097133
ENSMUSG00000058715	1.19	6.17501E-09
ENSMUSG00000031662	1.19	0.012229203
ENSMUSG00000032231	1.19	0.000540133
ENSMUSG00000078762	1.19	0.029588349
ENSMUSG00000056708	1.19	2.13639E-06
ENSMUSG00000066072	1.19	0.024063176
ENSMUSG00000020120	1.19	4.80628E-08
ENSMUSG00000021932	1.19	2.06033E-06
ENSMUSG00000060791	1.19	2.17538E-05
ENSMUSG00000056529	1.18	0.000693818
ENSMUSG00000020407	1.18	0.00952551
ENSMUSG00000029322	1.18	2.10162E-08
ENSMUSG00000020330	1.18	0.006289484
ENSMUSG00000030528	1.18	0.017806323
ENSMUSG00000038400	1.18	0.001395507
ENSMUSG00000034892	1.17	0.007065512
ENSMUSG00000014453	1.17	0.015901913
ENSMUSG00000052760	1.17	0.008445067
ENSMUSG00000036896	1.17	1.01343E-07
ENSMUSG00000006589	1.17	1.30688E-09
ENSMUSG00000062515	1.17	0.012099156
ENSMUSG00000027843	1.17	0.001850751
ENSMUSG00000032344	1.17	0.046341998
ENSMUSG00000024011	1.17	0.016097052
ENSMUSG00000036887	1.17	0.000280358
ENSMUSG00000061232	1.17	3.91533E-12
ENSMUSG00000004707	1.16	6.31645E-06
ENSMUSG00000046718	1.16	9.01282E-12
ENSMUSG00000029591	1.16	7.66446E-08
ENSMUSG00000027848	1.16	0.000269247
ENSMUSG00000049804	1.16	3.32983E-05
ENSMUSG00000069792	1.16	0.000523543
ENSMUSG00000037060	1.16	0.044651575
ENSMUSG00000030839	1.16	0.000324581
ENSMUSG00000047945	1.16	4.04225E-08
ENSMUSG00000042684	1.15	2.55804E-05
ENSMUSG00000022372	1.15	9.68544E-07
ENSMUSG00000028150	1.15	0.000124302
ENSMUSG00000043157	1.15	0.007350287
ENSMUSG00000029366	1.15	0.011724867
ENSMUSG00000023919	1.15	4.56687E-05
ENSMUSG00000039231	1.15	7.571E-07

Ensembl gene id	log2FC	p-adjusted
ENSMUSG00000028459	1.15	0.00943448
ENSMUSG00000028843	1.15	1.58546E-05
ENSMUSG00000034041	1.15	0.010478829
ENSMUSG00000036526	1.15	0.012185909
ENSMUSG00000038421	1.14	0.022910173
ENSMUSG00000021280	1.14	0.005420919
ENSMUSG00000073489	1.14	0.01179245
ENSMUSG00000073599	1.14	0.000188298
ENSMUSG00000027306	1.14	0.00442287
ENSMUSG00000049625	1.14	1.8153E-06
ENSMUSG00000042804	1.14	0.015264364
ENSMUSG00000092517	1.14	0.026791295
ENSMUSG00000022346	1.14	0.00025374
ENSMUSG00000055805	1.13	1.47542E-05
ENSMUSG00000038642	1.13	8.56113E-11
ENSMUSG00000026548	1.13	0.007839302
ENSMUSG00000031776	1.13	2.65513E-05
ENSMUSG00000035711	1.13	0.001148337
ENSMUSG00000026574	1.13	0.016343923
ENSMUSG00000046818	1.13	0.023906934
ENSMUSG00000057948	1.13	0.001481816
ENSMUSG00000034792	1.13	0.04707053
ENSMUSG00000031827	1.12	1.51374E-09
ENSMUSG00000019823	1.12	0.002629378
ENSMUSG00000043832	1.12	0.000190198
ENSMUSG00000061062	1.12	0.031095126
ENSMUSG00000049744	1.12	0.021285306
ENSMUSG00000026832	1.12	0.000528208
ENSMUSG00000024300	1.12	4.63986E-07
ENSMUSG00000029484	1.12	3.02555E-05
ENSMUSG00000066861	1.12	0.04776008
ENSMUSG00000029925	1.12	4.82455E-06
ENSMUSG00000024737	1.12	3.91808E-07
ENSMUSG00000057346	1.12	0.00320385
ENSMUSG00000032294	1.12	7.61636E-09
ENSMUSG00000036678	1.12	0.038194187
ENSMUSG00000031934	1.12	0.018965439
ENSMUSG00000021760	1.11	0.014750838
ENSMUSG00000051457	1.11	0.003260605
ENSMUSG00000058729	1.11	0.00533615
ENSMUSG00000019773	1.11	0.017318427
ENSMUSG00000026547	1.11	5.42349E-09
ENSMUSG00000060044	1.11	9.3378E-05
ENSMUSG00000037103	1.11	5.76204E-06
ENSMUSG00000074873	1.11	0.00137933
ENSMUSG00000060371	1.11	0.000840167
ENSMUSG00000056116	1.11	6.13648E-06
ENSMUSG00000036905	1.11	6.47643E-09
ENSMUSG00000027330	1.11	0.000791072
ENSMUSG00000022221	1.11	0.000678309
ENSMUSG00000037972	1.11	0.000107765
ENSMUSG00000020178	1.11	0.001968427

Ensembl gene id	log2FC	p-adjusted
ENSMUSG00000041515	1.11	1.5266E-07
ENSMUSG00000032643	1.11	0.041016552
ENSMUSG00000079293	1.11	0.000395394
ENSMUSG00000032555	1.10	0.041884387
ENSMUSG00000035692	1.10	6.95021E-08
ENSMUSG00000043421	1.10	0.013783552
ENSMUSG00000026009	1.10	0.018477855
ENSMUSG00000002983	1.10	0.000459202
ENSMUSG00000028581	1.10	1.46697E-09
ENSMUSG00000021115	1.10	4.24261E-05
ENSMUSG00000025511	1.10	9.14299E-10
ENSMUSG00000030579	1.10	1.90751E-08
ENSMUSG00000043079	1.10	1.04051E-06
ENSMUSG00000024590	1.09	1.83723E-06
ENSMUSG00000028874	1.09	4.75834E-05
ENSMUSG00000024013	1.09	1.89541E-05
ENSMUSG00000032085	1.09	6.33825E-07
ENSMUSG00000026358	1.09	0.022060061
ENSMUSG00000031697	1.09	0.004435009
ENSMUSG00000021451	1.09	0.000628104
ENSMUSG00000078771	1.09	0.000680077
ENSMUSG00000032010	1.09	9.91239E-08
ENSMUSG00000046157	1.09	0.000296504
ENSMUSG00000071715	1.08	1.71851E-05
ENSMUSG00000000740	1.08	7.82325E-05
ENSMUSG00000020038	1.08	0.014902944
ENSMUSG00000032135	1.08	2.96719E-05
ENSMUSG00000003283	1.08	1.10755E-06
ENSMUSG00000021624	1.08	0.000701029
ENSMUSG00000024909	1.08	0.000791761
ENSMUSG00000037601	1.08	6.36692E-06
ENSMUSG00000063838	1.08	0.020855866
ENSMUSG00000040345	1.08	8.88681E-05
ENSMUSG00000025747	1.08	0.006968214
ENSMUSG00000015355	1.08	8.84161E-05
ENSMUSG00000006931	1.08	0.030826931
ENSMUSG00000052726	1.08	0.012844416
ENSMUSG00000056069	1.08	0.000218571
ENSMUSG00000016494	1.07	0.000106766
ENSMUSG00000024339	1.07	6.81922E-06
ENSMUSG00000009633	1.07	0.000662385
ENSMUSG00000037280	1.07	0.005677526
ENSMUSG00000004891	1.07	1.82828E-05
ENSMUSG00000057135	1.07	0.000576471
ENSMUSG00000024177	1.07	0.006763512
ENSMUSG00000041420	1.07	0.023735499
ENSMUSG00000060950	1.07	0.002488054
ENSMUSG00000026972	1.07	0.000482368
ENSMUSG00000032332	1.07	0.016148412
ENSMUSG00000066363	1.07	0.000126137
ENSMUSG00000037217	1.06	0.023504625
ENSMUSG00000039208	1.06	0.000697485

Ensembl gene id	log2FC	p-adjusted
ENSMUSG00000052270	1.06	0.005067174
ENSMUSG00000066513	1.06	0.03568801
ENSMUSG00000037321	1.06	1.58069E-08
ENSMUSG00000025732	1.06	3.60586E-08
ENSMUSG00000020810	1.06	0.000209266
ENSMUSG00000027752	1.06	0.00071552
ENSMUSG00000004100	1.06	2.10308E-06
ENSMUSG00000057322	1.05	0.001968427
ENSMUSG00000026049	1.05	0.001042325
ENSMUSG00000028549	1.05	0.005268215
ENSMUSG00000064326	1.05	0.000416834
ENSMUSG00000030577	1.05	0.000105356
ENSMUSG00000024833	1.05	5.30079E-06
ENSMUSG00000018008	1.05	6.33825E-07
ENSMUSG00000078202	1.05	0.030607514
ENSMUSG00000037847	1.05	9.78028E-07
ENSMUSG00000032245	1.05	0.000115081
ENSMUSG00000049037	1.04	0.000157114
ENSMUSG00000047250	1.04	9.50733E-08
ENSMUSG00000018774	1.04	3.91013E-07
ENSMUSG00000055184	1.04	0.029118522
ENSMUSG00000025007	1.04	0.004756032
ENSMUSG00000043931	1.04	0.04188279
ENSMUSG00000024521	1.04	0.004830843
ENSMUSG00000034675	1.04	0.004481058
ENSMUSG00000076437	1.04	0.000185541
ENSMUSG00000029363	1.04	0.003010338
ENSMUSG00000027405	1.04	0.001715791
ENSMUSG00000038034	1.04	5.53813E-06
ENSMUSG00000015745	1.04	0.000221301
ENSMUSG00000015950	1.03	1.17708E-05
ENSMUSG00000067212	1.03	0.004132791
ENSMUSG00000072235	1.03	0.000426653
ENSMUSG00000030403	1.03	1.26358E-07
ENSMUSG00000008153	1.03	0.01997717
ENSMUSG00000021423	1.03	4.51779E-05
ENSMUSG00000028555	1.03	0.000118697
ENSMUSG00000021196	1.03	0.000310631
ENSMUSG00000020895	1.03	0.027580241
ENSMUSG00000040264	1.03	0.000281622
ENSMUSG00000036777	1.03	0.027049155
ENSMUSG00000039633	1.03	0.000267717
ENSMUSG00000033685	1.03	1.09592E-08
ENSMUSG00000056501	1.02	1.74317E-07
ENSMUSG00000097188	1.02	1.51071E-05
ENSMUSG00000000317	1.02	0.000488177
ENSMUSG00000027230	1.02	0.008326399
ENSMUSG00000030170	1.02	0.000852318
ENSMUSG00000034593	1.02	0.010135932
ENSMUSG00000002111	1.02	2.67986E-06
ENSMUSG00000001056	1.02	4.5885E-07
ENSMUSG00000071035	1.02	0.002174612

Ensembl gene id	log2FC	p-adjusted
ENSMUSG00000032577	1.02	0.016406213
ENSMUSG00000029687	1.02	4.75834E-05
ENSMUSG00000020834	1.02	0.000207713
ENSMUSG00000072620	1.02	9.1712E-06
ENSMUSG00000078780	1.02	0.023277641
ENSMUSG00000049932	1.01	1.088E-05
ENSMUSG00000079555	1.01	0.000343799
ENSMUSG00000032300	1.01	7.38329E-06
ENSMUSG00000029413	1.01	0.000200666
ENSMUSG00000026360	1.01	6.08425E-06
ENSMUSG00000079363	1.01	4.68225E-06
ENSMUSG00000042606	1.01	0.03843495
ENSMUSG00000017550	1.01	0.00449758
ENSMUSG00000024673	1.01	0.001842368
ENSMUSG00000064267	1.00	0.000573079
ENSMUSG00000063193	1.00	0.000299764
ENSMUSG00000024696	1.00	0.002787815
ENSMUSG00000030047	1.00	0.000231366
ENSMUSG00000026421	1.00	6.00165E-07
ENSMUSG00000041449	1.00	5.85508E-06
ENSMUSG00000031387	0.99	0.037346895
ENSMUSG00000026211	0.96	0.005600952
ENSMUSG00000063524	0.93	2.91359E-06
ENSMUSG00000036040	0.93	0.000693818
ENSMUSG00000001750	0.91	9.63433E-07
ENSMUSG00000032997	0.91	0.002468977
ENSMUSG00000007039	0.91	0.011190263
ENSMUSG00000038005	0.90	0.000112342
ENSMUSG00000006369	0.90	0.006210608
ENSMUSG00000034636	-1.00	5.44935E-08
ENSMUSG00000020275	-1.00	0.012630901
ENSMUSG00000026579	-1.01	0.000324642
ENSMUSG00000021013	-1.01	0.008994645
ENSMUSG00000070594	-1.01	6.17501E-09
ENSMUSG00000028053	-1.01	5.50477E-08
ENSMUSG00000073664	-1.01	9.91239E-08
ENSMUSG00000022292	-1.01	0.002048145
ENSMUSG00000039976	-1.01	9.62441E-07
ENSMUSG00000035078	-1.01	9.93606E-07
ENSMUSG00000089991	-1.01	5.43647E-08
ENSMUSG00000045064	-1.02	0.004486028
ENSMUSG00000022141	-1.02	4.27817E-08
ENSMUSG00000002222	-1.02	3.3844E-09
ENSMUSG00000033396	-1.03	1.18345E-06
ENSMUSG00000042202	-1.03	0.033621473
ENSMUSG00000061544	-1.03	0.000159184
ENSMUSG00000042985	-1.03	0.021131182
ENSMUSG00000025261	-1.03	2.69101E-09
ENSMUSG00000040127	-1.03	7.03878E-05
ENSMUSG00000081920	-1.03	0.006930302
ENSMUSG00000004500	-1.03	0.02143098
ENSMUSG00000059495	-1.03	3.16064E-09

Ensembl gene id	log2FC	p-adjusted
ENSMUSG00000075470	-1.03	5.11784E-06
ENSMUSG00000020634	-1.04	0.000109087
ENSMUSG00000063663	-1.04	6.58315E-06
ENSMUSG00000021638	-1.04	0.00288968
ENSMUSG00000062949	-1.05	1.34428E-09
ENSMUSG00000018417	-1.05	1.87181E-09
ENSMUSG00000033022	-1.05	7.67855E-10
ENSMUSG00000037541	-1.05	0.011341626
ENSMUSG00000050445	-1.05	0.021966189
ENSMUSG00000000056	-1.05	1.0393E-08
ENSMUSG00000049971	-1.05	0.04791488
ENSMUSG00000063894	-1.05	3.08595E-05
ENSMUSG00000029729	-1.05	7.43782E-09
ENSMUSG00000072664	-1.05	8.26476E-05
ENSMUSG00000020640	-1.06	9.12469E-08
ENSMUSG00000038648	-1.06	0.000424639
ENSMUSG00000027243	-1.06	3.62652E-06
ENSMUSG00000030313	-1.06	2.12062E-09
ENSMUSG00000033102	-1.06	0.003310386
ENSMUSG00000033883	-1.06	0.000163835
ENSMUSG00000020091	-1.06	7.92519E-08
ENSMUSG00000021903	-1.07	0.004003121
ENSMUSG00000020620	-1.07	1.69286E-09
ENSMUSG00000071633	-1.08	1.30688E-09
ENSMUSG00000003178	-1.08	0.000110228
ENSMUSG00000055660	-1.08	0.000131011
ENSMUSG00000043924	-1.08	0.001163196
ENSMUSG00000024070	-1.08	8.57329E-05
ENSMUSG00000082534	-1.09	0.003758235
ENSMUSG00000024827	-1.09	3.16454E-10
ENSMUSG00000036155	-1.09	0.000629182
ENSMUSG00000040536	-1.09	0.01046691
ENSMUSG00000021390	-1.10	0.044872733
ENSMUSG00000095253	-1.10	9.98925E-05
ENSMUSG00000037355	-1.10	8.56889E-07
ENSMUSG00000055480	-1.10	0.026671766
ENSMUSG00000037890	-1.10	0.017272165
ENSMUSG00000030131	-1.10	0.014902944
ENSMUSG00000055228	-1.10	0.029079004
ENSMUSG00000030806	-1.11	0.000107765
ENSMUSG00000060961	-1.11	1.01197E-08
ENSMUSG00000021611	-1.11	0.000134413
ENSMUSG00000020553	-1.11	0.039873575
ENSMUSG00000022521	-1.11	1.62896E-05
ENSMUSG00000085867	-1.12	1.26326E-06
ENSMUSG00000059708	-1.12	0.020767143
ENSMUSG00000020183	-1.12	0.005646219
ENSMUSG00000025255	-1.12	4.57409E-08
ENSMUSG00000095930	-1.13	0.000639887
ENSMUSG00000018166	-1.13	0.034512999
ENSMUSG00000020154	-1.13	8.49286E-11
ENSMUSG00000041308	-1.13	2.27057E-05

Ensembl gene id	log2FC	p-adjusted
ENSMUSG00000037621	-1.13	5.10237E-05
ENSMUSG00000035948	-1.13	0.005823863
ENSMUSG00000040446	-1.13	1.62896E-05
ENSMUSG00000070368	-1.13	0.003276948
ENSMUSG00000030655	-1.13	3.17907E-10
ENSMUSG00000044676	-1.14	0.000409238
ENSMUSG00000050195	-1.14	0.040016018
ENSMUSG00000067424	-1.14	0.005921761
ENSMUSG00000021223	-1.14	0.01101429
ENSMUSG00000060012	-1.14	6.85976E-09
ENSMUSG00000031853	-1.15	4.45587E-06
ENSMUSG00000063234	-1.15	0.038813879
ENSMUSG00000074829	-1.15	6.70529E-06
ENSMUSG00000020335	-1.15	0.005181035
ENSMUSG00000025949	-1.15	1.00926E-07
ENSMUSG00000048388	-1.15	0.002986072
ENSMUSG00000031666	-1.16	8.01224E-10
ENSMUSG00000019820	-1.16	3.03834E-10
ENSMUSG00000035878	-1.16	2.39899E-11
ENSMUSG00000042589	-1.16	0.005203609
ENSMUSG00000022615	-1.16	4.2529E-10
ENSMUSG00000062519	-1.16	0.001182406
ENSMUSG00000046541	-1.17	0.000475755
ENSMUSG00000022887	-1.17	4.98537E-11
ENSMUSG00000035164	-1.17	6.72632E-06
ENSMUSG00000047907	-1.17	0.029055103
ENSMUSG00000063550	-1.17	0.001744712
ENSMUSG00000036501	-1.17	4.58069E-09
ENSMUSG00000073016	-1.17	0.013365802
ENSMUSG00000006221	-1.17	0.03551092
ENSMUSG00000085328	-1.18	4.91297E-05
ENSMUSG00000033306	-1.18	2.58972E-11
ENSMUSG00000014164	-1.18	0.001483952
ENSMUSG00000025241	-1.18	9.37497E-11
ENSMUSG00000082791	-1.18	0.005769124
ENSMUSG00000074272	-1.19	1.44411E-11
ENSMUSG00000029512	-1.19	0.030247305
ENSMUSG00000078435	-1.19	0.014701795
ENSMUSG00000044452	-1.20	2.9264E-07
ENSMUSG00000064294	-1.20	4.28464E-12
ENSMUSG00000096910	-1.20	0.012788839
ENSMUSG00000096959	-1.20	0.010416697
ENSMUSG00000033871	-1.21	0.000845653
ENSMUSG00000045410	-1.21	0.016957869
ENSMUSG00000054659	-1.21	0.005185137
ENSMUSG00000051984	-1.21	0.00106315
ENSMUSG00000060397	-1.21	0.004841196
ENSMUSG00000064372	-1.21	0.000415229
ENSMUSG00000028780	-1.22	0.016390761
ENSMUSG00000039701	-1.22	1.75185E-05
ENSMUSG00000020609	-1.22	1.34163E-06
ENSMUSG00000031596	-1.22	0.000861412

Ensembl gene id	log2FC	p-adjusted
ENSMUSG00000082588	-1.23	0.019574145
ENSMUSG00000074519	-1.23	0.002207875
ENSMUSG00000025006	-1.23	1.68133E-06
ENSMUSG00000054387	-1.23	1.65002E-08
ENSMUSG00000027014	-1.23	0.013003029
ENSMUSG00000035270	-1.23	0.003949188
ENSMUSG00000048732	-1.25	0.007559957
ENSMUSG00000041324	-1.25	0.001189027
ENSMUSG00000033458	-1.25	5.40414E-06
ENSMUSG00000031433	-1.25	0.000289505
ENSMUSG00000032715	-1.26	0.039307762
ENSMUSG00000029167	-1.26	0.00407203
ENSMUSG00000026017	-1.26	0.00389764
ENSMUSG00000037795	-1.27	3.15728E-11
ENSMUSG00000041482	-1.27	0.031475901
ENSMUSG00000020037	-1.27	2.75118E-05
ENSMUSG00000044795	-1.27	8.26476E-05
ENSMUSG00000040274	-1.27	0.000240596
ENSMUSG00000029765	-1.27	0.002126787
ENSMUSG00000016028	-1.27	0.002225602
ENSMUSG00000037003	-1.27	2.18329E-07
ENSMUSG00000021767	-1.28	9.68857E-08
ENSMUSG00000030486	-1.29	0.020327956
ENSMUSG00000032898	-1.29	0.00754109
ENSMUSG00000026004	-1.29	2.11112E-08
ENSMUSG00000028630	-1.29	4.25446E-09
ENSMUSG00000039145	-1.29	9.13422E-14
ENSMUSG00000029004	-1.30	3.91533E-12
ENSMUSG00000050954	-1.30	0.002050932
ENSMUSG00000031822	-1.31	3.18481E-06
ENSMUSG00000051452	-1.31	6.33825E-07
ENSMUSG00000039853	-1.31	5.75543E-10
ENSMUSG00000031326	-1.31	0.009549225
ENSMUSG00000037270	-1.32	1.11652E-13
ENSMUSG00000037112	-1.32	3.19932E-07
ENSMUSG00000042680	-1.32	1.32826E-11
ENSMUSG00000019726	-1.33	1.04045E-10
ENSMUSG00000032743	-1.33	3.76655E-10
ENSMUSG00000089828	-1.33	0.016807054
ENSMUSG00000064367	-1.34	1.33345E-07
ENSMUSG00000029195	-1.34	4.98257E-13
ENSMUSG00000020593	-1.34	0.005272094
ENSMUSG00000055704	-1.35	0.026114968
ENSMUSG00000056342	-1.35	7.26024E-14
ENSMUSG00000073555	-1.35	3.89068E-15
ENSMUSG00000046532	-1.35	1.46463E-07
ENSMUSG00000056019	-1.35	0.001294737
ENSMUSG00000035933	-1.36	2.95287E-09
ENSMUSG00000058921	-1.36	1.10406E-14
ENSMUSG00000027965	-1.36	0.007984979
ENSMUSG00000019368	-1.36	6.2266E-16
ENSMUSG00000088022	-1.37	0.043023463

Ensembl gene id	log2FC	p-adjusted
ENSMUSG00000040761	-1.37	6.33379E-08
ENSMUSG00000028030	-1.37	1.75309E-07
ENSMUSG00000067219	-1.38	0.001040325
ENSMUSG00000060530	-1.38	0.043676395
ENSMUSG00000032064	-1.38	3.92186E-06
ENSMUSG00000022519	-1.38	7.68326E-05
ENSMUSG00000070985	-1.39	7.27823E-14
ENSMUSG00000026398	-1.39	1.11823E-14
ENSMUSG00000033863	-1.40	3.61948E-14
ENSMUSG00000027796	-1.40	1.51374E-09
ENSMUSG00000041078	-1.40	0.021561649
ENSMUSG00000052981	-1.41	0.005842364
ENSMUSG00000070583	-1.41	0.029292266
ENSMUSG00000038751	-1.41	3.0407E-05
ENSMUSG00000093385	-1.41	0.011970701
ENSMUSG00000020604	-1.41	5.76149E-10
ENSMUSG00000043418	-1.41	0.000195291
ENSMUSG00000087368	-1.41	0.041539702
ENSMUSG00000097969	-1.41	0.045478212
ENSMUSG00000033713	-1.41	7.95068E-08
ENSMUSG00000094441	-1.41	0.00013066
ENSMUSG00000087543	-1.42	0.029280977
ENSMUSG00000032065	-1.42	0.008623491
ENSMUSG00000063047	-1.42	0.017917817
ENSMUSG00000059908	-1.42	0.007893218
ENSMUSG00000087424	-1.42	0.008662997
ENSMUSG00000049985	-1.42	1.87384E-10
ENSMUSG00000031529	-1.43	2.39899E-11
ENSMUSG00000072999	-1.43	0.047889417
ENSMUSG00000079065	-1.43	1.54481E-07
ENSMUSG00000055980	-1.44	3.7306E-12
ENSMUSG00000039004	-1.44	0.000810045
ENSMUSG00000033855	-1.45	2.69101E-09
ENSMUSG00000096463	-1.45	0.007310701
ENSMUSG00000025202	-1.45	0.004029751
ENSMUSG00000019947	-1.45	4.18013E-08
ENSMUSG00000038384	-1.45	0.000293461
ENSMUSG00000025017	-1.46	2.79278E-16
ENSMUSG00000041702	-1.46	1.69546E-08
ENSMUSG00000030424	-1.46	0.017677177
ENSMUSG00000052496	-1.46	0.006418611
ENSMUSG00000039477	-1.46	1.58431E-05
ENSMUSG00000024924	-1.46	0.024384241
ENSMUSG00000038024	-1.47	3.5384E-13
ENSMUSG00000090145	-1.47	3.7306E-12
ENSMUSG00000054385	-1.47	0.002371666
ENSMUSG00000020948	-1.48	2.55804E-05
ENSMUSG00000035133	-1.48	2.75689E-12
ENSMUSG00000074925	-1.48	0.000573079
ENSMUSG00000037259	-1.49	0.044963639
ENSMUSG00000062901	-1.49	0.004550078
ENSMUSG00000042460	-1.50	1.89814E-08

Ensembl gene id	log2FC	p-adjusted
ENSMUSG00000028864	-1.50	5.57236E-12
ENSMUSG00000015405	-1.51	0.014151732
ENSMUSG00000025764	-1.51	6.40492E-17
ENSMUSG00000032410	-1.52	1.30688E-09
ENSMUSG00000090353	-1.52	0.007624384
ENSMUSG00000064349	-1.52	0.002999636
ENSMUSG00000047036	-1.52	4.65996E-13
ENSMUSG00000052812	-1.53	2.03453E-10
ENSMUSG00000035349	-1.53	1.03017E-17
ENSMUSG00000047793	-1.53	3.11395E-06
ENSMUSG00000014547	-1.54	3.45874E-07
ENSMUSG00000097554	-1.54	6.12023E-10
ENSMUSG00000048424	-1.55	0.028002927
ENSMUSG00000005580	-1.55	1.18345E-06
ENSMUSG00000044086	-1.55	0.02742208
ENSMUSG00000020431	-1.56	0.015410297
ENSMUSG00000052595	-1.56	3.48558E-20
ENSMUSG00000022676	-1.56	9.22077E-09
ENSMUSG00000031292	-1.57	0.000521634
ENSMUSG00000026384	-1.57	4.90391E-08
ENSMUSG00000048489	-1.58	0.027128067
ENSMUSG00000031010	-1.58	2.51952E-19
ENSMUSG00000053205	-1.59	0.01540139
ENSMUSG00000006777	-1.59	0.024099827
ENSMUSG00000071083	-1.59	0.007407047
ENSMUSG00000071037	-1.59	0.000871935
ENSMUSG00000002265	-1.59	4.48821E-05
ENSMUSG00000033792	-1.61	1.43244E-07
ENSMUSG00000090659	-1.61	0.00047354
ENSMUSG00000038917	-1.61	0.020707812
ENSMUSG00000097657	-1.61	0.001910753
ENSMUSG00000060429	-1.63	4.89159E-11
ENSMUSG00000035284	-1.63	1.06474E-17
ENSMUSG00000066647	-1.64	3.35077E-05
ENSMUSG00000067653	-1.64	0.009810246
ENSMUSG00000025262	-1.66	5.4094E-09
ENSMUSG00000044749	-1.66	1.65468E-22
ENSMUSG00000090286	-1.66	0.005465501
ENSMUSG00000042390	-1.67	3.1177E-09
ENSMUSG00000022383	-1.68	2.65368E-09
ENSMUSG00000057230	-1.68	1.74317E-07
ENSMUSG00000047746	-1.69	0.040610399
ENSMUSG00000054178	-1.69	1.43516E-05
ENSMUSG00000005893	-1.70	1.1279E-15
ENSMUSG00000018581	-1.71	0.004689024
ENSMUSG00000049694	-1.72	0.046172027
ENSMUSG00000094410	-1.72	1.97045E-17
ENSMUSG00000055341	-1.72	0.020707812
ENSMUSG00000048280	-1.73	0.000149231
ENSMUSG00000023800	-1.73	0.000951906
ENSMUSG00000024600	-1.74	0.005190455
ENSMUSG00000021798	-1.74	0.008896887

Ensembl gene id	log2FC	p-adjusted
ENSMUSG00000021466	-1.75	6.36872E-09
ENSMUSG00000038894	-1.76	0.04190289
ENSMUSG00000061436	-1.77	1.32826E-11
ENSMUSG00000050556	-1.77	0.003124946
ENSMUSG00000037709	-1.78	1.39733E-15
ENSMUSG00000097131	-1.78	3.10613E-08
ENSMUSG00000023845	-1.78	1.71428E-18
ENSMUSG00000053877	-1.79	1.38457E-15
ENSMUSG00000056133	-1.80	0.04467268
ENSMUSG00000024558	-1.80	0.030563175
ENSMUSG00000038725	-1.81	0.001696899
ENSMUSG00000099032	-1.81	4.35543E-09
ENSMUSG00000085929	-1.81	0.000652197
ENSMUSG00000050919	-1.82	0.007352676
ENSMUSG00000086450	-1.83	0.010672432
ENSMUSG00000033107	-1.83	6.08142E-09
ENSMUSG00000078185	-1.83	0.026560797
ENSMUSG00000045467	-1.83	0.000570865
ENSMUSG00000059824	-1.84	1.77382E-08
ENSMUSG00000049630	-1.84	0.022099947
ENSMUSG00000038233	-1.86	9.19236E-20
ENSMUSG00000086231	-1.86	4.80512E-05
ENSMUSG00000083798	-1.88	0.036954238
ENSMUSG00000044359	-1.88	1.44885E-17
ENSMUSG00000075307	-1.89	0.023731264
ENSMUSG00000031461	-1.89	0.028953167
ENSMUSG00000094856	-1.89	0.035278676
ENSMUSG00000034342	-1.90	1.38644E-11
ENSMUSG00000025194	-1.90	1.73557E-24
ENSMUSG00000032009	-1.91	2.98826E-16
ENSMUSG00000028039	-1.91	0.004716555
ENSMUSG00000074794	-1.91	5.4248E-05
ENSMUSG00000097766	-1.92	0.03443506
ENSMUSG00000063488	-1.92	2.69101E-09
ENSMUSG00000063446	-1.94	0.005690674
ENSMUSG00000037139	-1.95	0.011831846
ENSMUSG00000056258	-1.97	0.006373888
ENSMUSG00000074283	-1.97	8.76414E-06
ENSMUSG00000023044	-1.98	0.025184108
ENSMUSG00000083116	-2.00	0.015972614
ENSMUSG00000066687	-2.01	0.001518076
ENSMUSG00000034551	-2.01	0.032975215
ENSMUSG00000033624	-2.02	7.33308E-24
ENSMUSG00000053141	-2.02	0.019434517
ENSMUSG00000046169	-2.03	0.02458561
ENSMUSG00000078486	-2.03	0.013280896
ENSMUSG00000039704	-2.04	2.05218E-21
ENSMUSG00000028834	-2.04	0.007200227
ENSMUSG00000090369	-2.04	0.002315688
ENSMUSG00000091089	-2.05	0.004076708
ENSMUSG00000055202	-2.06	0.005946761
ENSMUSG00000045094	-2.07	1.21425E-17

Ensembl gene id	log2FC	p-adjusted
ENSMUSG00000079645	-2.07	0.034260152
ENSMUSG00000069893	-2.10	0.005078121
ENSMUSG00000080223	-2.10	0.012503768
ENSMUSG00000034584	-2.11	2.87678E-17
ENSMUSG00000025019	-2.13	1.77538E-20
ENSMUSG00000024298	-2.14	4.03148E-25
ENSMUSG00000098741	-2.18	4.89625E-05
ENSMUSG00000049690	-2.18	0.004839982
ENSMUSG00000041624	-2.19	4.89625E-05
ENSMUSG00000039891	-2.19	0.010146369
ENSMUSG00000004939	-2.19	0.005293086
ENSMUSG00000071204	-2.21	3.62652E-06
ENSMUSG00000030046	-2.21	1.34677E-06
ENSMUSG00000027855	-2.22	0.003563313
ENSMUSG00000067336	-2.24	6.6102E-22
ENSMUSG00000040694	-2.27	0.029040812
ENSMUSG00000034731	-2.28	0.007734771
ENSMUSG00000038594	-2.30	1.68237E-19
ENSMUSG00000083287	-2.33	1.82828E-05
ENSMUSG00000021373	-2.37	0.006628992
ENSMUSG00000072612	-2.37	0.028910957
ENSMUSG00000064347	-2.38	0.024182653
ENSMUSG00000019787	-2.40	0.009751559
ENSMUSG00000072294	-2.40	1.30688E-09
ENSMUSG00000070385	-2.45	0.009213471
ENSMUSG00000084761	-2.48	0.002618498
ENSMUSG00000026950	-2.51	0.004411157
ENSMUSG00000054453	-2.53	1.38984E-10
ENSMUSG00000091405	-2.55	0.000403972
ENSMUSG00000061462	-2.59	0.021737658
ENSMUSG00000051497	-2.62	0.00177459
ENSMUSG00000097322	-2.63	0.014902944
ENSMUSG00000047419	-2.64	0.010484531
ENSMUSG00000040093	-2.67	0.018577358
ENSMUSG00000090942	-2.82	1.98953E-08
ENSMUSG00000052229	-2.83	0.000864192
ENSMUSG00000097452	-2.83	0.000261442
ENSMUSG00000034159	-2.90	0.035664563
ENSMUSG00000082364	-2.91	0.001467496
ENSMUSG00000045441	-2.94	2.2571E-27
ENSMUSG00000063415	-2.95	1.40391E-09
ENSMUSG00000089827	-3.04	0.011782705
ENSMUSG00000052316	-3.07	0.008915587
ENSMUSG00000087579	-3.08	3.39058E-05
ENSMUSG00000026475	-3.08	0.006537244
ENSMUSG00000038092	-3.10	0.000543004
ENSMUSG00000020889	-3.16	4.94429E-20
ENSMUSG00000087353	-3.17	0.012789511
ENSMUSG00000084834	-3.19	0.001789473
ENSMUSG00000079588	-3.43	0.020675192
ENSMUSG00000061780	-3.84	0.02685648
ENSMUSG00000056771	-4.57	0.020113606

Ensembl gene id	log2FC	p-adjusted
**Sm_Vh_46**		
ENSMUSG00000000290	10.60	2.84801E-67
ENSMUSG00000063779	9.55	7.02006E-33
ENSMUSG00000050370	8.62	1.07788E-33
ENSMUSG00000040809	8.53	1.62858E-37
ENSMUSG00000061100	8.46	1.24381E-20
ENSMUSG00000076655	8.35	0.040736036
ENSMUSG00000022157	8.23	6.66514E-05
ENSMUSG00000001131	8.16	5.33599E-11
ENSMUSG00000009185	8.13	2.99039E-06
ENSMUSG00000095285	8.04	0.005965621
ENSMUSG00000058126	7.98	0.0011094
ENSMUSG00000030111	7.97	0.014878884
ENSMUSG00000076031	7.91	0.021469086
ENSMUSG00000082976	7.84	8.04009E-14
ENSMUSG00000056399	7.79	0.003334763
ENSMUSG00000047222	7.71	5.66533E-13
ENSMUSG00000064272	7.66	3.67848E-06
ENSMUSG00000035373	7.56	5.75095E-13
ENSMUSG00000027073	7.49	0.014709334
ENSMUSG00000052234	7.32	0.003905809
ENSMUSG00000035186	7.26	0.000680725
ENSMUSG00000053338	7.14	1.18024E-13
ENSMUSG00000000805	7.12	2.97901E-66
ENSMUSG00000016498	6.92	1.67843E-29
ENSMUSG00000022651	6.90	4.73805E-20
ENSMUSG00000054196	6.85	2.98787E-20
ENSMUSG00000057729	6.75	1.26367E-06
ENSMUSG00000094749	6.74	0.031407488
ENSMUSG00000028072	6.71	6.61374E-19
ENSMUSG00000020676	6.71	1.7338E-29
ENSMUSG00000062148	6.68	2.11377E-07
ENSMUSG00000021758	6.68	2.75417E-14
ENSMUSG00000090166	6.45	8.32352E-09
ENSMUSG00000052435	6.42	1.7174E-08
ENSMUSG00000062380	6.35	5.68976E-16
ENSMUSG00000024681	6.33	0.014307608
ENSMUSG00000076614	6.32	0.012985848
ENSMUSG00000026822	6.27	7.23125E-24
ENSMUSG00000031762	6.25	4.58942E-13
ENSMUSG00000028965	6.16	1.09934E-12
ENSMUSG00000004791	6.12	1.56459E-06
ENSMUSG00000072596	6.11	2.99132E-15
ENSMUSG00000018500	6.11	7.34696E-13
ENSMUSG00000031162	6.08	0.009136608
ENSMUSG00000034634	6.02	5.81471E-07
ENSMUSG00000031780	6.01	6.85125E-07
ENSMUSG00000015652	5.99	2.06828E-06
ENSMUSG00000072601	5.96	4.39001E-10
ENSMUSG00000024029	5.95	5.93777E-06
ENSMUSG00000024529	5.94	2.73518E-30
ENSMUSG00000072599	5.94	5.82957E-07

Ensembl gene id	log2FC	p-adjusted
ENSMUSG00000024503	5.94	0.001743056
ENSMUSG00000015134	5.91	4.3963E-43
ENSMUSG00000041831	5.86	5.17485E-06
ENSMUSG00000007888	5.83	4.93093E-06
ENSMUSG00000021062	5.81	2.11535E-13
ENSMUSG00000038973	5.78	0.020044198
ENSMUSG00000051314	5.76	2.02731E-10
ENSMUSG00000062345	5.76	1.26742E-20
ENSMUSG00000020125	5.69	0.005857153
ENSMUSG00000004814	5.69	3.81961E-16
ENSMUSG00000022479	5.68	1.49373E-13
ENSMUSG00000020826	5.66	1.82121E-22
ENSMUSG00000020383	5.65	5.5747E-13
ENSMUSG00000050578	5.60	2.49861E-50
ENSMUSG00000057465	5.55	0.001367838
ENSMUSG00000030268	5.54	3.8215E-15
ENSMUSG00000062309	5.54	8.80185E-09
ENSMUSG00000047904	5.54	1.34888E-08
ENSMUSG00000098318	5.48	8.69972E-12
ENSMUSG00000043903	5.43	2.12557E-11
ENSMUSG00000031765	5.41	5.47485E-05
ENSMUSG00000021508	5.40	0.000132642
ENSMUSG00000033213	5.38	4.48555E-11
ENSMUSG00000009350	5.37	0.02595608
ENSMUSG00000018623	5.36	5.9411E-14
ENSMUSG00000004113	5.35	0.034170775
ENSMUSG00000044162	5.34	1.43807E-10
ENSMUSG00000058755	5.32	3.37647E-07
ENSMUSG00000040314	5.31	0.035612112
ENSMUSG00000001506	5.29	1.31799E-36
ENSMUSG00000052212	5.27	0.005861362
ENSMUSG00000095170	5.24	0.016096849
ENSMUSG00000038357	5.24	0.006647104
ENSMUSG00000074115	5.19	0.00039192
ENSMUSG00000037437	5.14	0.044872501
ENSMUSG00000068744	5.07	0.001912736
ENSMUSG00000037411	5.07	0.017284171
ENSMUSG00000028415	5.07	0.001733997
ENSMUSG00000076672	5.04	0.029838886
ENSMUSG00000023992	5.03	6.5154E-13
ENSMUSG00000040498	5.03	0.029026391
ENSMUSG00000054136	5.03	0.004731709
ENSMUSG00000019890	5.01	2.39659E-08
ENSMUSG00000095007	4.99	0.003821698
ENSMUSG00000025469	4.95	0.003384832
ENSMUSG00000010760	4.95	0.028655697
ENSMUSG00000040197	4.94	1.0204E-05
ENSMUSG00000032484	4.94	0.028302263
ENSMUSG00000037868	4.94	3.08761E-28
ENSMUSG00000022226	4.93	0.004454032
ENSMUSG00000096515	4.92	0.003608483
ENSMUSG00000026955	4.92	2.63755E-06

Ensembl gene id	log2FC	p-adjusted
ENSMUSG00000025473	4.91	3.53812E-43
ENSMUSG00000032487	4.91	3.10282E-08
ENSMUSG00000027656	4.91	2.74806E-06
ENSMUSG00000050761	4.85	0.004536611
ENSMUSG00000068452	4.84	0.009376013
ENSMUSG00000061540	4.83	0.000191872
ENSMUSG00000021403	4.83	2.00177E-09
ENSMUSG00000083695	4.82	0.019166165
ENSMUSG00000094509	4.82	0.024320679
ENSMUSG00000040026	4.80	5.29017E-12
ENSMUSG00000056071	4.80	0.000907672
ENSMUSG00000034226	4.79	0.008354404
ENSMUSG00000030413	4.79	1.63043E-16
ENSMUSG00000023473	4.79	0.008434504
ENSMUSG00000021268	4.79	0.011822636
ENSMUSG00000091694	4.78	0.027234519
ENSMUSG00000026285	4.77	1.74753E-05
ENSMUSG00000023067	4.76	1.2988E-07
ENSMUSG00000025804	4.76	3.92743E-34
ENSMUSG00000020241	4.75	7.16564E-36
ENSMUSG00000029811	4.73	2.81972E-20
ENSMUSG00000035352	4.72	9.53689E-05
ENSMUSG00000004267	4.70	2.50882E-08
ENSMUSG00000038037	4.70	6.33362E-16
ENSMUSG00000025161	4.69	2.55506E-22
ENSMUSG00000034394	4.69	0.000394866
ENSMUSG00000029223	4.68	0.000124223
ENSMUSG00000093861	4.67	0.028022675
ENSMUSG00000064246	4.66	2.10168E-11
ENSMUSG00000035385	4.65	1.12599E-06
ENSMUSG00000023926	4.64	0.031715426
ENSMUSG00000030162	4.64	4.71465E-10
ENSMUSG00000001119	4.64	2.11907E-64
ENSMUSG00000049723	4.63	7.08649E-57
ENSMUSG00000006574	4.63	0.029176286
ENSMUSG00000026073	4.62	2.04721E-07
ENSMUSG00000044303	4.60	3.56445E-07
ENSMUSG00000076434	4.58	0.014985562
ENSMUSG00000025400	4.58	1.36074E-06
ENSMUSG00000084796	4.58	5.94818E-13
ENSMUSG00000032496	4.58	0.00524544
ENSMUSG00000061068	4.57	0.016076023
ENSMUSG00000013766	4.56	3.73662E-09
ENSMUSG00000091345	4.54	1.37866E-11
ENSMUSG00000020702	4.54	1.50229E-06
ENSMUSG00000001555	4.54	1.42257E-19
ENSMUSG00000044313	4.54	0.010953496
ENSMUSG00000097451	4.53	0.018155312
ENSMUSG00000044071	4.53	0.022773309
ENSMUSG00000066071	4.52	0.023124692
ENSMUSG00000040152	4.51	3.42093E-16
ENSMUSG00000030054	4.51	0.005183694

Ensembl gene id	log2FC	p-adjusted
ENSMUSG00000022126	4.48	5.7577E-15
ENSMUSG00000027750	4.44	5.86487E-22
ENSMUSG00000029352	4.44	0.005164888
ENSMUSG00000050157	4.43	0.03489098
ENSMUSG00000076586	4.42	0.023463789
ENSMUSG00000022440	4.37	4.5423E-31
ENSMUSG00000053398	4.37	2.74891E-09
ENSMUSG00000035165	4.36	8.16006E-07
ENSMUSG00000097792	4.36	0.03500265
ENSMUSG00000046341	4.34	8.7509E-10
ENSMUSG00000022367	4.34	0.040086597
ENSMUSG00000023993	4.33	0.012898279
ENSMUSG00000056054	4.33	0.004213225
ENSMUSG00000070369	4.32	0.001440242
ENSMUSG00000041481	4.31	1.29164E-23
ENSMUSG00000038379	4.31	0.000216479
ENSMUSG00000021614	4.29	2.772E-25
ENSMUSG00000029272	4.27	0.009212694
ENSMUSG00000037010	4.26	0.001686539
ENSMUSG00000024989	4.26	0.001545801
ENSMUSG00000044201	4.25	0.000172994
ENSMUSG00000024791	4.24	0.000190918
ENSMUSG00000003051	4.22	1.60792E-07
ENSMUSG00000050359	4.22	0.005513633
ENSMUSG00000068129	4.21	3.07766E-11
ENSMUSG00000040950	4.21	1.6953E-14
ENSMUSG00000046223	4.20	8.82599E-29
ENSMUSG00000026011	4.20	4.03704E-07
ENSMUSG00000028217	4.20	0.002098868
ENSMUSG00000025351	4.19	5.84264E-14
ENSMUSG00000094769	4.18	0.023274293
ENSMUSG00000018930	4.18	6.9559E-17
ENSMUSG00000042757	4.17	4.5578E-18
ENSMUSG00000042265	4.17	0.004525339
ENSMUSG00000026630	4.16	1.76112E-12
ENSMUSG00000021485	4.15	6.76501E-09
ENSMUSG00000021697	4.14	5.18915E-09
ENSMUSG00000046167	4.12	0.00026095
ENSMUSG00000029915	4.12	2.27318E-05
ENSMUSG00000029373	4.11	0.009593744
ENSMUSG00000076596	4.11	0.007620885
ENSMUSG00000037568	4.09	7.52916E-05
ENSMUSG00000028644	4.07	0.018494132
ENSMUSG00000039109	4.07	8.84987E-07
ENSMUSG00000085081	4.07	0.02770407
ENSMUSG00000074676	4.06	7.23812E-07
ENSMUSG00000020787	4.05	0.000118203
ENSMUSG00000041046	4.05	2.91739E-06
ENSMUSG00000044309	4.05	2.53033E-12
ENSMUSG00000030789	4.04	1.8596E-08
ENSMUSG00000018924	4.04	2.71454E-15
ENSMUSG00000016763	4.04	1.75157E-14

Ensembl gene id	log2FC	p-adjusted
ENSMUSG00000044927	4.02	0.010371722
ENSMUSG00000040907	4.02	3.29876E-20
ENSMUSG00000032783	4.01	1.11952E-06
ENSMUSG00000001228	4.01	0.003344384
ENSMUSG00000006398	4.01	4.26349E-05
ENSMUSG00000027220	3.99	0.006688101
ENSMUSG00000029661	3.99	1.88847E-28
ENSMUSG00000030584	3.99	5.62037E-07
ENSMUSG00000019942	3.98	1.38766E-05
ENSMUSG00000036223	3.98	1.28455E-06
ENSMUSG00000062184	3.97	0.001489231
ENSMUSG00000033491	3.96	0.00712085
ENSMUSG00000030867	3.96	6.93664E-06
ENSMUSG00000000982	3.96	8.65412E-07
ENSMUSG00000031933	3.96	6.37043E-05
ENSMUSG00000003484	3.96	7.72376E-14
ENSMUSG00000022033	3.95	0.001188915
ENSMUSG00000089929	3.95	8.16635E-16
ENSMUSG00000024173	3.94	0.015535551
ENSMUSG00000035678	3.93	0.000135766
ENSMUSG00000001025	3.92	6.62671E-13
ENSMUSG00000078521	3.92	0.02245553
ENSMUSG00000020598	3.92	0.000890108
ENSMUSG00000046006	3.92	9.64113E-10
ENSMUSG00000058579	3.91	0.020413682
ENSMUSG00000027994	3.90	0.000101091
ENSMUSG00000020808	3.90	2.47373E-14
ENSMUSG00000076570	3.89	0.003727404
ENSMUSG00000044678	3.89	0.013236568
ENSMUSG00000031538	3.87	7.7619E-14
ENSMUSG00000036117	3.87	0.000370804
ENSMUSG00000060572	3.87	7.69754E-09
ENSMUSG00000030137	3.87	0.01387985
ENSMUSG00000030677	3.86	9.65636E-07
ENSMUSG00000050335	3.86	1.53257E-45
ENSMUSG00000005800	3.86	1.79409E-10
ENSMUSG00000030606	3.85	0.010289959
ENSMUSG00000028364	3.84	2.98991E-14
ENSMUSG00000022227	3.84	0.002008299
ENSMUSG00000028175	3.83	0.00233063
ENSMUSG00000079523	3.83	1.28935E-24
ENSMUSG00000079445	3.83	1.63812E-07
ENSMUSG00000020897	3.82	2.08002E-07
ENSMUSG00000022584	3.82	2.61165E-08
ENSMUSG00000048485	3.82	0.000137983
ENSMUSG00000024907	3.82	0.012711044
ENSMUSG00000001865	3.82	0.000872572
ENSMUSG00000053318	3.81	0.000313012
ENSMUSG00000063018	3.81	0.001726883
ENSMUSG00000042489	3.80	0.000254645
ENSMUSG00000064147	3.80	1.03501E-15
ENSMUSG00000096422	3.80	0.003366616

Ensembl gene id	log2FC	p-adjusted
ENSMUSG00000018927	3.79	6.56298E-15
ENSMUSG00000027966	3.79	0.018448469
ENSMUSG00000068220	3.78	4.21263E-11
ENSMUSG00000037466	3.78	7.76716E-08
ENSMUSG00000029061	3.77	2.53548E-15
ENSMUSG00000028832	3.77	6.26882E-08
ENSMUSG00000031779	3.77	1.00858E-05
ENSMUSG00000023940	3.76	0.021701967
ENSMUSG00000021298	3.76	1.61692E-10
ENSMUSG00000026204	3.76	2.12589E-06
ENSMUSG00000010830	3.75	2.18087E-13
ENSMUSG00000026043	3.75	1.06911E-28
ENSMUSG00000032556	3.75	0.022313145
ENSMUSG00000023033	3.74	2.33363E-10
ENSMUSG00000062991	3.74	0.002981523
ENSMUSG00000024402	3.74	0.00069796
ENSMUSG00000047501	3.73	6.80218E-05
ENSMUSG00000034883	3.73	0.004133863
ENSMUSG00000031740	3.72	2.49935E-12
ENSMUSG00000029307	3.70	0.001073163
ENSMUSG00000022422	3.70	0.005850285
ENSMUSG00000003541	3.69	2.03048E-10
ENSMUSG00000020695	3.68	1.26363E-08
ENSMUSG00000030114	3.67	0.003511714
ENSMUSG00000027797	3.67	5.20065E-13
ENSMUSG00000027408	3.64	1.85536E-06
ENSMUSG00000076934	3.63	0.003251411
ENSMUSG00000030346	3.63	3.61911E-05
ENSMUSG00000032374	3.63	1.29606E-23
ENSMUSG00000020914	3.63	1.47914E-05
ENSMUSG00000073400	3.63	0.003617177
ENSMUSG00000001020	3.63	9.91536E-22
ENSMUSG00000036587	3.62	3.02792E-08
ENSMUSG00000060586	3.61	4.5578E-18
ENSMUSG00000032218	3.61	7.793E-10
ENSMUSG00000027379	3.60	0.002477591
ENSMUSG00000068606	3.59	0.001463266
ENSMUSG00000076609	3.58	0.016593595
ENSMUSG00000049103	3.57	4.31846E-32
ENSMUSG00000027230	3.56	1.11079E-19
ENSMUSG00000056737	3.56	9.15118E-13
ENSMUSG00000096751	3.56	0.034828703
ENSMUSG00000023908	3.56	0.000377765
ENSMUSG00000041801	3.55	0.000629676
ENSMUSG00000051839	3.54	0.029254737
ENSMUSG00000024670	3.54	5.3068E-06
ENSMUSG00000005339	3.54	0.027252978
ENSMUSG00000021953	3.54	0.005883704
ENSMUSG00000037725	3.54	0.022969078
ENSMUSG00000024353	3.54	0.030604344
ENSMUSG00000017737	3.53	1.82631E-08
ENSMUSG00000030208	3.52	2.9372E-21

Ensembl gene id	log2FC	p-adjusted
ENSMUSG00000021822	3.52	2.95464E-23
ENSMUSG00000095771	3.52	0.003041454
ENSMUSG00000004371	3.51	0.000556122
ENSMUSG00000023905	3.51	8.19167E-09
ENSMUSG00000037161	3.51	0.001075132
ENSMUSG00000028587	3.51	1.2206E-06
ENSMUSG00000004933	3.49	3.06707E-12
ENSMUSG00000027715	3.49	9.43443E-06
ENSMUSG00000021384	3.48	3.33445E-05
ENSMUSG00000037628	3.48	0.000122635
ENSMUSG00000027469	3.48	3.09864E-05
ENSMUSG00000032322	3.48	5.70859E-16
ENSMUSG00000026683	3.47	0.000222577
ENSMUSG00000031506	3.47	3.35349E-18
ENSMUSG00000026039	3.46	0.007236284
ENSMUSG00000027368	3.46	1.35553E-10

Ensembl gene id	log2FC	p-adjusted
ENSMUSG00000000290	2.83	0.00392108
ENSMUSG00000063779	2.82	0.00391613
ENSMUSG00000050370	2.81	0.00391118
ENSMUSG00000040809	2.79	0.003906229
ENSMUSG00000061100	2.78	0.003901279
ENSMUSG00000076655	2.77	0.003896329
ENSMUSG00000022157	2.76	0.003891378
ENSMUSG00000001131	2.75	0.003886428
ENSMUSG00000009185	2.74	0.003881478
ENSMUSG00000095285	2.72	0.003876527
ENSMUSG00000058126	2.71	0.003871577
ENSMUSG00000030111	2.70	0.003866627
ENSMUSG00000076031	2.69	0.003861676
ENSMUSG00000082976	2.68	0.003856726
ENSMUSG00000056399	2.67	0.003851776
ENSMUSG00000047222	2.65	0.003846826
ENSMUSG00000064272	2.64	0.003841875
ENSMUSG00000035373	2.63	0.003836925
ENSMUSG00000027073	2.62	0.003831975
ENSMUSG00000052234	2.61	0.003827024
ENSMUSG00000035186	2.60	0.003822074
ENSMUSG00000053338	2.58	0.003817124
ENSMUSG00000000805	2.57	0.003812173
ENSMUSG00000016498	2.56	0.003807223
ENSMUSG00000022651	2.55	0.003802273
ENSMUSG00000054196	2.54	0.003797323
ENSMUSG00000057729	2.53	0.003792372
ENSMUSG00000094749	2.51	0.003787422
ENSMUSG00000028072	2.50	0.003782472
ENSMUSG00000020676	2.49	0.003777521
ENSMUSG00000062148	2.48	0.003772571
ENSMUSG00000021758	2.47	0.003767621
ENSMUSG00000090166	2.45	0.00376267
ENSMUSG00000052435	2.44	0.00375772
ENSMUSG00000062380	2.43	0.00375277

Ensembl gene id	log2FC	p-adjusted
ENSMUSG00000024681	2.42	0.00374782
ENSMUSG00000076614	2.41	0.003742869
ENSMUSG00000026822	2.40	0.003737919
ENSMUSG00000031762	2.38	0.003732969
ENSMUSG00000028965	2.37	0.003728018
ENSMUSG00000004791	2.36	0.003723068
ENSMUSG00000072596	2.35	0.003718118
ENSMUSG00000018500	2.34	0.003713167
ENSMUSG00000031162	2.33	0.003708217
ENSMUSG00000034634	2.31	0.003703267
ENSMUSG00000031780	2.30	0.003698316
ENSMUSG00000015652	2.29	0.003693366
ENSMUSG00000072601	2.28	0.003688416
ENSMUSG00000024029	2.27	0.003683466
ENSMUSG00000024529	2.26	0.003678515
ENSMUSG00000072599	2.24	0.003673565
ENSMUSG00000024503	2.23	0.003668615
ENSMUSG00000015134	2.22	0.003663664
ENSMUSG00000042808	3.29	0.032628176
ENSMUSG00000049420	3.29	0.02316201
ENSMUSG00000069910	3.29	2.75754E-06
ENSMUSG00000052271	3.28	0.008011446
ENSMUSG00000046714	3.28	0.007156907
ENSMUSG00000079547	3.28	9.40614E-05
ENSMUSG00000024806	3.27	0.005750328
ENSMUSG00000023132	3.26	2.01234E-12
ENSMUSG00000031262	3.26	0.009447989
ENSMUSG00000022034	3.26	0.007605652
ENSMUSG00000006403	3.25	0.000508984
ENSMUSG00000041064	3.25	1.18266E-06
ENSMUSG00000036322	3.25	0.011293429
ENSMUSG00000076617	3.25	0.004966713
ENSMUSG00000027848	3.23	2.46404E-12
ENSMUSG00000008136	3.22	8.07382E-05
ENSMUSG00000067149	3.22	0.003130867
ENSMUSG00000055235	3.22	1.7707E-13
ENSMUSG00000038963	3.22	0.000258501
ENSMUSG00000028678	3.22	0.001223176
ENSMUSG00000022225	3.22	0.008835014
ENSMUSG00000094694	3.22	0.004124902
ENSMUSG00000041605	3.21	0.000281793
ENSMUSG00000021728	3.21	5.03204E-11
ENSMUSG00000038943	3.21	0.007271548
ENSMUSG00000019982	3.21	0.007878855
ENSMUSG00000094335	3.20	0.010976713
ENSMUSG00000017716	3.20	2.45785E-05
ENSMUSG00000032334	3.19	1.95704E-09
ENSMUSG00000024598	3.19	0.002425314
ENSMUSG00000026728	3.19	3.23087E-35
ENSMUSG00000039013	3.18	0.000760189
ENSMUSG00000086291	3.18	0.009634613
ENSMUSG00000029322	3.18	3.75825E-24

Ensembl gene id	log2FC	p-adjusted
ENSMUSG00000079553	3.17	0.000279288
ENSMUSG00000028602	3.17	0.009634396
ENSMUSG00000029378	3.17	0.01048551
ENSMUSG00000079120	3.17	0.00997016
ENSMUSG00000087362	3.17	0.003802524
ENSMUSG00000050105	3.17	0.010505054
ENSMUSG00000025912	3.17	0.003162399
ENSMUSG00000027654	3.16	0.002612871
ENSMUSG00000024672	3.16	0.000669603
ENSMUSG00000026196	3.15	0.001138763
ENSMUSG00000021464	3.15	0.024228296
ENSMUSG00000031410	3.15	0.039401068
ENSMUSG00000095630	3.15	0.02151138
ENSMUSG00000055629	3.14	0.003853718
ENSMUSG00000024056	3.14	3.78226E-05
ENSMUSG00000068696	3.14	0.01084273
ENSMUSG00000022032	3.13	6.13601E-06
ENSMUSG00000030742	3.13	6.56298E-15
ENSMUSG00000042345	3.13	8.18159E-05
ENSMUSG00000022494	3.13	0.014093983
ENSMUSG00000071714	3.13	1.61836E-11
ENSMUSG00000002100	3.12	0.013835901
ENSMUSG00000005696	3.12	0.004420432
ENSMUSG00000097487	3.12	0.000634931
ENSMUSG00000032231	3.12	2.96178E-33
ENSMUSG00000079597	3.12	0.016061752
ENSMUSG00000059900	3.12	0.013236568
ENSMUSG00000046591	3.12	2.40377E-06
ENSMUSG00000054342	3.12	1.38245E-13
ENSMUSG00000033453	3.12	1.05998E-09
ENSMUSG00000000983	3.12	0.003972638
ENSMUSG00000025330	3.12	0.000323398
ENSMUSG00000073421	3.12	2.13989E-10
ENSMUSG00000048040	3.11	0.004646923
ENSMUSG00000030793	3.11	2.17144E-14
ENSMUSG00000032294	3.11	1.15124E-32
ENSMUSG00000000318	3.10	2.0839E-12
ENSMUSG00000025746	3.10	0.041102348
ENSMUSG00000015316	3.10	0.000146763
ENSMUSG00000022322	3.10	0.027592157
ENSMUSG00000032011	3.09	5.10563E-09
ENSMUSG00000031562	3.09	0.001750111
ENSMUSG00000020810	3.09	1.06911E-28
ENSMUSG00000056481	3.08	2.32307E-18
ENSMUSG00000028873	3.08	1.2839E-06
ENSMUSG00000020635	3.07	7.98501E-05
ENSMUSG00000028068	3.07	0.000250919
ENSMUSG00000095180	3.07	5.47102E-05
ENSMUSG00000005470	3.07	1.72965E-10
ENSMUSG00000037166	3.07	4.18107E-05
ENSMUSG00000036768	3.07	3.24897E-11
ENSMUSG00000027004	3.07	2.3297E-17

Ensembl gene id	log2FC	p-adjusted
ENSMUSG00000063646	3.07	3.62499E-05
ENSMUSG00000040751	3.07	1.28013E-14
ENSMUSG00000028111	3.06	1.55225E-11
ENSMUSG00000024909	3.06	9.60745E-11
ENSMUSG00000038523	3.06	0.010708048
ENSMUSG00000027323	3.06	0.002054642
ENSMUSG00000023015	3.06	2.66428E-07
ENSMUSG00000054200	3.06	0.003931006
ENSMUSG00000024968	3.06	0.000238699
ENSMUSG00000031934	3.06	3.15769E-06
ENSMUSG00000024388	3.06	0.004394185
ENSMUSG00000054675	3.05	2.25046E-15
ENSMUSG00000000869	3.05	0.002569781
ENSMUSG00000034833	3.04	0.003225244
ENSMUSG00000017652	3.04	1.87627E-15
ENSMUSG00000037337	3.03	3.04857E-15
ENSMUSG00000030124	3.03	2.46106E-08
ENSMUSG00000004612	3.03	1.91643E-16
ENSMUSG00000037649	3.03	6.11433E-06
ENSMUSG00000051811	3.02	8.08026E-06
ENSMUSG00000045502	3.02	1.32339E-09
ENSMUSG00000037095	3.02	0.001000499
ENSMUSG00000022203	3.02	1.15673E-05
ENSMUSG00000030745	3.02	2.4396E-08
ENSMUSG00000026390	3.01	0.000582072
ENSMUSG00000038059	3.01	8.18246E-08
ENSMUSG00000058290	3.01	2.84785E-11
ENSMUSG00000034612	3.01	5.59501E-11
ENSMUSG00000034855	3.01	0.008356552
ENSMUSG00000026837	3.01	1.43267E-30
ENSMUSG00000027907	3.00	1.73494E-17
ENSMUSG00000006179	3.00	0.016958104
ENSMUSG00000020323	3.00	0.020956557
ENSMUSG00000054999	3.00	0.006128636
ENSMUSG00000019992	2.99	4.74869E-05
ENSMUSG00000045763	2.99	2.73632E-17
ENSMUSG00000093726	2.98	0.023904213
ENSMUSG00000003352	2.98	9.6626E-12
ENSMUSG00000030717	2.97	2.53237E-06
ENSMUSG00000095488	2.97	0.00420513
ENSMUSG00000032323	2.97	0.000544952
ENSMUSG00000027316	2.97	0.025466394
ENSMUSG00000046841	2.97	1.58056E-11
ENSMUSG00000006519	2.96	7.91851E-20
ENSMUSG00000079019	2.96	0.007053627
ENSMUSG00000029752	2.96	2.23104E-06
ENSMUSG00000046295	2.96	4.39628E-06
ENSMUSG00000051727	2.96	0.015865889
ENSMUSG00000031595	2.95	2.49803E-05
ENSMUSG00000058427	2.95	0.002054642
ENSMUSG00000042804	2.95	8.54433E-18
ENSMUSG00000034570	2.95	0.000333375

Ensembl gene id	log2FC	p-adjusted
ENSMUSG00000000791	2.94	1.28462E-05
ENSMUSG00000075602	2.94	0.004924221
ENSMUSG00000005640	2.94	0.022906805
ENSMUSG00000086742	2.94	0.020696635
ENSMUSG00000050014	2.94	0.001073072
ENSMUSG00000030154	2.94	0.002237527
ENSMUSG00000083679	2.94	3.26835E-05
ENSMUSG00000046160	2.94	0.026396294
ENSMUSG00000024669	2.93	2.51351E-05
ENSMUSG00000048922	2.93	8.16542E-05
ENSMUSG00000076523	2.92	0.004532419
ENSMUSG00000002228	2.92	1.94727E-06
ENSMUSG00000038775	2.92	0.003337438
ENSMUSG00000023903	2.92	0.006788454
ENSMUSG00000027331	2.91	1.73769E-06
ENSMUSG00000023505	2.91	0.010522552
ENSMUSG00000022021	2.91	3.16454E-05
ENSMUSG00000039055	2.91	0.003716293
ENSMUSG00000022947	2.90	0.007206327
ENSMUSG00000040016	2.90	0.000891684
ENSMUSG00000031150	2.90	4.10502E-06
ENSMUSG00000026358	2.90	0.018130825
ENSMUSG00000026271	2.90	7.27779E-09
ENSMUSG00000018339	2.89	9.64872E-10
ENSMUSG00000097113	2.89	0.006688101
ENSMUSG00000020649	2.89	0.004876748
ENSMUSG00000020325	2.89	0.029082155
ENSMUSG00000005553	2.88	0.025496575
ENSMUSG00000035042	2.88	2.91156E-20
ENSMUSG00000026547	2.88	1.25284E-16
ENSMUSG00000044748	2.87	8.00102E-05
ENSMUSG00000029371	2.87	0.024753636
ENSMUSG00000055298	2.87	0.023416035
ENSMUSG00000006360	2.87	6.11433E-06
ENSMUSG00000029283	2.87	1.63283E-05
ENSMUSG00000024610	2.87	4.59006E-15
ENSMUSG00000029581	2.86	1.59065E-23
ENSMUSG00000040204	2.86	3.62936E-06
ENSMUSG00000022385	2.86	0.014311252
ENSMUSG00000026875	2.86	5.03502E-12
ENSMUSG00000022018	2.86	0.002817199
ENSMUSG00000024028	2.86	0.003738612
ENSMUSG00000068758	2.85	8.52067E-06
ENSMUSG00000050071	2.85	0.004405503
ENSMUSG00000020077	2.85	4.8965E-27
ENSMUSG00000017002	2.85	0.018838139
ENSMUSG00000026069	2.84	1.32797E-09
ENSMUSG00000059089	2.84	1.08976E-24
ENSMUSG00000024011	2.84	4.57992E-09
ENSMUSG00000051517	2.83	0.000119606
ENSMUSG00000045273	2.83	3.0406E-06
ENSMUSG00000016283	2.83	6.42744E-09

Ensembl gene id	log2FC	p-adjusted
ENSMUSG00000037544	2.83	0.00610015
ENSMUSG00000021596	2.83	0.000129569
ENSMUSG00000029718	2.82	2.26138E-13
ENSMUSG00000035279	2.82	2.16073E-11
ENSMUSG00000027962	2.82	4.56253E-05
ENSMUSG00000031289	2.81	2.27489E-16
ENSMUSG00000096140	2.81	0.029426768
ENSMUSG00000030142	2.81	0.000930139
ENSMUSG00000064109	2.80	0.000839409
ENSMUSG00000007805	2.80	0.002811683
ENSMUSG00000021453	2.79	1.3041E-24
ENSMUSG00000021263	2.79	8.31106E-09
ENSMUSG00000076522	2.79	0.003408067
ENSMUSG00000048534	2.79	0.00026594
ENSMUSG00000043496	2.79	2.60083E-07
ENSMUSG00000035683	2.78	0.00736976
ENSMUSG00000047139	2.78	0.007698672
ENSMUSG00000065999	2.78	0.0053781
ENSMUSG00000045328	2.78	0.000381822
ENSMUSG00000000693	2.77	3.68132E-06
ENSMUSG00000035365	2.77	0.018389438
ENSMUSG00000014813	2.77	0.03220902
ENSMUSG00000028041	2.77	8.5242E-22
ENSMUSG00000031906	2.76	0.018411174
ENSMUSG00000042821	2.76	1.09148E-09
ENSMUSG00000045826	2.76	8.77954E-15
ENSMUSG00000066861	2.76	0.008932765
ENSMUSG00000024675	2.76	0.008172589
ENSMUSG00000037887	2.76	1.53646E-10
ENSMUSG00000076490	2.75	2.99691E-06
ENSMUSG00000079293	2.75	5.11112E-20
ENSMUSG00000005667	2.75	1.47668E-14
ENSMUSG00000022659	2.75	0.012388494
ENSMUSG00000030707	2.75	7.10639E-25
ENSMUSG00000040035	2.75	0.000144874
ENSMUSG00000070436	2.75	1.5093E-07
ENSMUSG00000092035	2.75	1.54858E-05
ENSMUSG00000032717	2.75	1.16828E-07
ENSMUSG00000031838	2.74	4.1901E-19
ENSMUSG00000022015	2.74	0.041102348
ENSMUSG00000071470	2.74	0.034170775
ENSMUSG00000096768	2.74	0.000234399
ENSMUSG00000022102	2.74	1.14082E-21
ENSMUSG00000031662	2.74	8.65652E-13
ENSMUSG00000021965	2.74	0.017706397
ENSMUSG00000020388	2.74	6.41648E-17
ENSMUSG00000006219	2.73	1.21514E-19
ENSMUSG00000001517	2.73	0.018842663
ENSMUSG00000098090	2.73	0.000490686
ENSMUSG00000043421	2.73	0.001115465
ENSMUSG00000037991	2.73	0.008775222
ENSMUSG00000023972	2.73	1.03174E-15

Ensembl gene id	log2FC	p-adjusted
ENSMUSG00000044707	2.73	0.000439158
ENSMUSG00000070645	2.72	0.016359219
ENSMUSG00000087187	2.72	0.005834374
ENSMUSG00000026928	2.72	9.24752E-10
ENSMUSG00000002257	2.72	5.03724E-10
ENSMUSG00000037548	2.72	1.45221E-11
ENSMUSG00000031497	2.72	0.000176599
ENSMUSG00000020415	2.72	8.58291E-08
ENSMUSG00000022665	2.71	2.05305E-17
ENSMUSG00000044811	2.71	1.24303E-15
ENSMUSG00000032094	2.71	4.01382E-07
ENSMUSG00000037913	2.71	0.002914694
ENSMUSG00000026628	2.71	1.73022E-17
ENSMUSG00000059830	2.71	0.00404873
ENSMUSG00000034317	2.71	1.75456E-12
ENSMUSG00000001270	2.71	8.48301E-10
ENSMUSG00000084845	2.70	0.019674355
ENSMUSG00000036594	2.70	1.1057E-13
ENSMUSG00000015619	2.70	1.06486E-06
ENSMUSG00000019539	2.69	2.44911E-10
ENSMUSG00000031290	2.68	0.005655241
ENSMUSG00000026442	2.68	3.52391E-08
ENSMUSG00000072980	2.68	0.025880376
ENSMUSG00000021760	2.68	2.95691E-06
ENSMUSG00000037313	2.68	6.15077E-06
ENSMUSG00000051022	2.68	1.30173E-06
ENSMUSG00000026429	2.67	0.000378811
ENSMUSG00000041774	2.67	5.03718E-05
ENSMUSG00000024660	2.67	9.81183E-08
ENSMUSG00000021675	2.67	0.009981237
ENSMUSG00000006931	2.67	5.56185E-12
ENSMUSG00000032436	2.67	3.51186E-19
ENSMUSG00000022876	2.67	5.57252E-07
ENSMUSG00000050010	2.66	0.014133778
ENSMUSG00000034266	2.66	3.65919E-10
ENSMUSG00000035692	2.66	0.015124475
ENSMUSG00000022099	2.66	0.020322622
ENSMUSG00000050107	2.65	2.88256E-07
ENSMUSG00000016494	2.65	1.74002E-09
ENSMUSG00000051439	2.65	6.63806E-21
ENSMUSG00000097432	2.65	0.022938398
ENSMUSG00000034786	2.65	6.30948E-17
ENSMUSG00000005824	2.64	0.000204622
ENSMUSG00000074570	2.64	0.000704085
ENSMUSG00000027221	2.64	0.000196586
ENSMUSG00000095562	2.64	3.19666E-08
ENSMUSG00000018819	2.64	9.0618E-21
ENSMUSG00000012889	2.64	1.73503E-07
ENSMUSG00000028541	2.64	4.43479E-07
ENSMUSG00000034765	2.64	1.49847E-11
ENSMUSG00000053862	2.64	0.003813696
ENSMUSG00000039942	2.64	2.92351E-06

Ensembl gene id	log2FC	p-adjusted
ENSMUSG00000071068	2.63	6.52475E-11
ENSMUSG00000003153	2.63	1.96768E-07
ENSMUSG00000000204	2.63	0.004525646
ENSMUSG00000039542	2.63	0.000685302
ENSMUSG00000085786	2.63	0.0084495
ENSMUSG00000030220	2.63	3.16922E-23
ENSMUSG00000038264	2.63	1.12333E-05
ENSMUSG00000050212	2.63	1.02094E-05
ENSMUSG00000005410	2.63	0.001384402
ENSMUSG00000039787	2.62	6.52878E-06
ENSMUSG00000032098	2.62	0.007306949
ENSMUSG00000019214	2.62	0.000268724
ENSMUSG00000048458	2.62	0.011796876
ENSMUSG00000026956	2.62	2.53548E-15
ENSMUSG00000025930	2.62	0.030863057
ENSMUSG00000050022	2.62	5.97489E-11
ENSMUSG00000004709	2.62	0.000221704
ENSMUSG00000048402	2.61	0.029350403
ENSMUSG00000056413	2.61	2.88231E-14
ENSMUSG00000052087	2.61	5.08064E-08
ENSMUSG00000023216	2.61	0.004760979
ENSMUSG00000082292	2.61	0.011634
ENSMUSG00000022439	2.61	4.31281E-19
ENSMUSG00000030148	2.61	8.79711E-15
ENSMUSG00000041754	2.60	0.00364724
ENSMUSG00000013707	2.60	2.03012E-15
ENSMUSG00000085058	2.60	0.030159764
ENSMUSG00000025701	2.60	0.000261829
ENSMUSG00000034906	2.60	0.001008417
ENSMUSG00000002233	2.60	1.81838E-21
ENSMUSG00000062007	2.60	0.004593318
ENSMUSG00000029096	2.60	3.03968E-16
ENSMUSG00000051279	2.59	1.77261E-07
ENSMUSG00000092920	2.59	0.006047983
ENSMUSG00000048126	2.59	0.000503329
ENSMUSG00000039153	2.59	3.39694E-06
ENSMUSG00000070000	2.59	3.82999E-08
ENSMUSG00000034311	2.59	0.00182727
ENSMUSG00000031253	2.59	1.98363E-11
ENSMUSG00000029923	2.59	4.44392E-06
ENSMUSG00000056812	2.59	0.025268537
ENSMUSG00000032221	2.58	0.018550308
ENSMUSG00000057058	2.58	6.64108E-08
ENSMUSG00000085580	2.58	0.00486253
ENSMUSG00000031390	2.58	0.035708212
ENSMUSG00000002835	2.58	0.001437073
ENSMUSG00000026211	2.58	9.25187E-12
ENSMUSG00000063838	2.58	1.40619E-11
ENSMUSG00000024349	2.58	1.89675E-18
ENSMUSG00000034591	2.57	1.9107E-09
ENSMUSG00000022816	2.57	1.9906E-12
ENSMUSG00000076498	2.57	4.93093E-06

Ensembl gene id	log2FC	p-adjusted
ENSMUSG00000029204	2.57	3.76883E-13
ENSMUSG00000019139	2.57	3.07395E-19
ENSMUSG00000062661	2.56	2.35656E-05
ENSMUSG00000046908	2.56	2.16414E-07
ENSMUSG00000075224	2.56	0.017467877
ENSMUSG00000036777	2.56	0.001014183
ENSMUSG00000025007	2.56	7.85715E-15
ENSMUSG00000061356	2.56	1.18725E-07
ENSMUSG00000000627	2.56	0.003788378
ENSMUSG00000030200	2.56	0.011173076
ENSMUSG00000087006	2.56	1.43664E-06
ENSMUSG00000023411	2.55	2.10479E-06
ENSMUSG00000036040	2.55	2.10509E-06
ENSMUSG00000009687	2.55	1.53844E-20
ENSMUSG00000030587	2.55	2.78232E-05
ENSMUSG00000026605	2.55	0.007849487
ENSMUSG00000074469	2.55	0.031549339
ENSMUSG00000040212	2.54	2.47048E-17
ENSMUSG00000076564	2.54	0.018375049
ENSMUSG00000037820	2.54	3.02344E-24
ENSMUSG00000020429	2.54	0.003562347
ENSMUSG00000036764	2.54	0.019116638
ENSMUSG00000025489	2.53	0.026868391
ENSMUSG00000032815	2.53	0.00040817
ENSMUSG00000033220	2.53	5.86487E-22
ENSMUSG00000010142	2.53	1.03912E-08
ENSMUSG00000089672	2.53	6.80979E-15
ENSMUSG00000032446	2.53	0.00022362
ENSMUSG00000096336	2.53	0.003957936
ENSMUSG00000031778	2.53	1.47532E-05
ENSMUSG00000024912	2.53	0.011507944
ENSMUSG00000040084	2.52	0.002242361
ENSMUSG00000030144	2.52	0.004234474
ENSMUSG00000002204	2.52	1.47708E-11
ENSMUSG00000052125	2.52	1.95478E-05
ENSMUSG00000020142	2.52	0.016634452
ENSMUSG00000024910	2.52	2.53237E-06
ENSMUSG00000023886	2.51	0.000956303
ENSMUSG00000062524	2.51	8.46052E-09
ENSMUSG00000049775	2.51	6.21559E-12
ENSMUSG00000073412	2.51	8.84543E-11
ENSMUSG00000082902	2.51	0.021814384
ENSMUSG00000085156	2.51	7.21152E-10
ENSMUSG00000001444	2.51	3.4853E-07
ENSMUSG00000022048	2.51	7.53072E-12
ENSMUSG00000000028	2.51	0.000825101
ENSMUSG00000031880	2.51	0.002363902
ENSMUSG00000000486	2.50	1.91318E-13
ENSMUSG00000060568	2.50	0.000585965
ENSMUSG00000026622	2.50	0.008920265
ENSMUSG00000020099	2.50	7.761E-19
ENSMUSG00000087366	2.49	0.002394675

Ensembl gene id	log2FC	p-adjusted
ENSMUSG00000061577	2.49	0.000522777
ENSMUSG00000021280	2.49	3.00125E-08
ENSMUSG00000031495	2.49	0.023212943
ENSMUSG00000024399	2.49	1.59029E-14
ENSMUSG00000075702	2.49	7.19347E-06
ENSMUSG00000039774	2.49	0.001338118
ENSMUSG00000056427	2.49	3.73053E-07
ENSMUSG00000047497	2.48	0.007222216
ENSMUSG00000071637	2.48	7.06874E-16
ENSMUSG00000048779	2.48	9.32931E-17
ENSMUSG00000042485	2.48	3.68221E-11
ENSMUSG00000026587	2.48	0.035112917
ENSMUSG00000027490	2.48	0.001075598
ENSMUSG00000045573	2.48	0.000597422
ENSMUSG00000024397	2.48	0.000101299
ENSMUSG00000076937	2.48	4.27987E-09
ENSMUSG00000034675	2.47	1.66958E-13
ENSMUSG00000038508	2.47	0.029784153
ENSMUSG00000032997	2.47	1.08009E-16
ENSMUSG00000042684	2.47	2.44209E-17
ENSMUSG00000091938	2.47	0.007578716
ENSMUSG00000034205	2.47	6.80626E-13
ENSMUSG00000036067	2.47	1.48413E-07
ENSMUSG00000097296	2.47	2.42573E-05
ENSMUSG00000054717	2.46	8.06406E-09
ENSMUSG00000028702	2.46	0.002352946
ENSMUSG00000019467	2.46	4.55152E-13
ENSMUSG00000087365	2.46	0.001588557
ENSMUSG00000087107	2.46	2.62615E-16
ENSMUSG00000094796	2.45	0.014830614
ENSMUSG00000087060	2.45	0.007129069
ENSMUSG00000029380	2.44	5.86086E-05
ENSMUSG00000026649	2.44	0.000208267
ENSMUSG00000056529	2.44	1.35637E-08
ENSMUSG00000060044	2.44	5.89153E-17
ENSMUSG00000073599	2.44	5.28583E-15
ENSMUSG00000039748	2.44	0.006763133
ENSMUSG00000043681	2.44	0.01759558
ENSMUSG00000034023	2.44	0.003690567
ENSMUSG00000043931	2.43	1.1098E-09
ENSMUSG00000040592	2.43	0.003444209
ENSMUSG00000022456	2.43	0.00964693
ENSMUSG00000036446	2.43	5.58773E-08
ENSMUSG00000032135	2.43	2.08202E-15
ENSMUSG00000060063	2.43	6.80979E-15
ENSMUSG00000028843	2.43	1.92199E-13
ENSMUSG00000025150	2.43	0.035836581
ENSMUSG00000020303	2.43	0.000872554
ENSMUSG00000062753	2.43	3.63819E-05
ENSMUSG00000001918	2.42	1.97418E-14
ENSMUSG00000035455	2.42	0.002486571
ENSMUSG00000020407	2.42	9.46155E-10

Ensembl gene id	log2FC	p-adjusted
ENSMUSG00000097136	2.42	0.003644042
ENSMUSG00000040473	2.42	0.013271288
ENSMUSG00000020057	2.41	3.57468E-13
ENSMUSG00000071715	2.41	2.69965E-17
ENSMUSG00000022474	2.41	6.63397E-13
ENSMUSG00000070691	2.41	8.99166E-08
ENSMUSG00000020493	2.40	0.011877954
ENSMUSG00000029075	2.40	1.36969E-05
ENSMUSG00000027134	2.40	8.99166E-08
ENSMUSG00000024538	2.40	1.52455E-16
ENSMUSG00000031142	2.40	0.002242794
ENSMUSG00000029641	2.40	0.001086131
ENSMUSG00000031904	2.40	3.20094E-07
ENSMUSG00000052776	2.40	0.005085566
ENSMUSG00000043017	2.40	1.82958E-05
ENSMUSG00000028931	2.40	1.15581E-11
ENSMUSG00000079018	2.40	0.012299639
ENSMUSG00000015568	2.39	4.03115E-21
ENSMUSG00000034987	2.39	0.009895673
ENSMUSG00000050410	2.39	0.000702761
ENSMUSG00000026546	2.39	0.018461601
ENSMUSG00000072844	2.39	0.009512972
ENSMUSG00000021423	2.39	2.51052E-05
ENSMUSG00000061322	2.39	0.006417717
ENSMUSG00000089762	2.39	0.003915788
ENSMUSG00000078794	2.39	9.80504E-07
ENSMUSG00000040675	2.38	5.5168E-12
ENSMUSG00000052142	2.38	3.60768E-12
ENSMUSG00000019303	2.38	6.87406E-05
ENSMUSG00000008318	2.38	1.12915E-07
ENSMUSG00000020363	2.38	2.08634E-10
ENSMUSG00000034773	2.38	0.044411134
ENSMUSG00000006585	2.37	0.003824569
ENSMUSG00000047534	2.37	0.000370477
ENSMUSG00000001864	2.37	0.002252115
ENSMUSG00000024501	2.37	3.77937E-16
ENSMUSG00000024965	2.37	2.20694E-18
ENSMUSG00000053746	2.37	0.012604322
ENSMUSG00000074480	2.37	0.000683006
ENSMUSG00000024379	2.37	0.043968283
ENSMUSG00000029417	2.36	0.003898781
ENSMUSG00000032577	2.36	6.77177E-11
ENSMUSG00000082163	2.36	0.037181262
ENSMUSG00000047945	2.36	1.84896E-18
ENSMUSG00000031101	2.36	7.01465E-11
ENSMUSG00000045382	2.36	1.22277E-05
ENSMUSG00000087658	2.36	0.010411543
ENSMUSG00000000562	2.36	0.041307084
ENSMUSG00000079017	2.36	0.004578752
ENSMUSG00000036887	2.36	0.000144205
ENSMUSG00000031304	2.35	1.49639E-14
ENSMUSG00000003355	2.35	4.44353E-09

Ensembl gene id	log2FC	p-adjusted
ENSMUSG00000027306	2.35	7.38982E-05
ENSMUSG00000038034	2.35	4.21857E-14
ENSMUSG00000025165	2.35	1.00036E-05
ENSMUSG00000026480	2.35	3.44487E-17
ENSMUSG00000029675	2.35	3.50788E-12
ENSMUSG00000040247	2.34	1.04649E-10
ENSMUSG00000026520	2.34	5.57903E-12
ENSMUSG00000021262	2.34	2.00177E-09
ENSMUSG00000031827	2.34	1.83408E-18
ENSMUSG00000090733	2.34	0.019074443
ENSMUSG00000034792	2.34	1.57231E-07
ENSMUSG00000059791	2.34	4.24364E-09
ENSMUSG00000068105	2.34	0.000364393
ENSMUSG00000034164	2.34	0.000303916
ENSMUSG00000041420	2.34	5.0665E-05
ENSMUSG00000066170	2.34	0.000901317
ENSMUSG00000017724	2.34	0.001675337
ENSMUSG00000095788	2.34	0.008261069
ENSMUSG00000038642	2.33	3.29051E-17
ENSMUSG00000029359	2.33	0.003220766
ENSMUSG00000026357	2.33	0.024840465
ENSMUSG00000026580	2.33	3.38566E-12
ENSMUSG00000069792	2.33	8.77693E-06
ENSMUSG00000011256	2.33	1.10981E-09
ENSMUSG00000031387	2.32	1.64398E-12
ENSMUSG00000020437	2.32	3.44489E-14
ENSMUSG00000062210	2.32	3.08396E-15
ENSMUSG00000024486	2.32	4.86195E-11
ENSMUSG00000029552	2.32	1.58375E-11
ENSMUSG00000046031	2.32	6.99837E-11
ENSMUSG00000031503	2.32	9.37733E-20
ENSMUSG00000030147	2.32	0.031715426
ENSMUSG00000004891	2.31	8.18724E-13
ENSMUSG00000000244	2.31	9.59269E-07
ENSMUSG00000056394	2.31	5.96785E-06
ENSMUSG00000039396	2.31	2.36366E-05
ENSMUSG00000029163	2.31	5.36997E-20
ENSMUSG00000056708	2.31	9.35583E-17
ENSMUSG00000029108	2.31	3.71864E-06
ENSMUSG00000046417	2.31	5.02726E-11
ENSMUSG00000037169	2.31	9.20586E-08
ENSMUSG00000063388	2.30	0.006445446
ENSMUSG00000076508	2.30	0.024571471
ENSMUSG00000031821	2.30	0.000752283
ENSMUSG00000072620	2.30	6.9559E-17
ENSMUSG00000057191	2.30	4.32862E-17
ENSMUSG00000037813	2.30	0.000883542
ENSMUSG00000029484	2.30	1.68541E-15
ENSMUSG00000029414	2.30	0.005750328
ENSMUSG00000022096	2.29	0.027502574
ENSMUSG00000047557	2.29	1.40212E-06
ENSMUSG00000064023	2.29	0.000323331

Ensembl gene id	log2FC	p-adjusted
ENSMUSG00000029778	2.29	3.97755E-08
ENSMUSG00000052477	2.29	0.000958527
ENSMUSG00000032184	2.29	1.3369E-07
ENSMUSG00000028862	2.29	6.07181E-05
ENSMUSG00000047798	2.28	1.53033E-12
ENSMUSG00000037239	2.28	0.035112917
ENSMUSG00000068335	2.28	9.69079E-13
ENSMUSG00000053044	2.28	0.006643592
ENSMUSG00000039699	2.28	2.68044E-06
ENSMUSG00000096727	2.28	0.000351948
ENSMUSG00000024640	2.28	2.23271E-07
ENSMUSG00000022346	2.27	1.90421E-10
ENSMUSG00000015314	2.27	5.52025E-05
ENSMUSG00000030165	2.27	0.000229154
ENSMUSG00000024696	2.27	1.84392E-10
ENSMUSG00000042246	2.26	0.005810619
ENSMUSG00000044827	2.26	6.31418E-10
ENSMUSG00000056888	2.26	9.4887E-07
ENSMUSG00000028071	2.26	1.80129E-05
ENSMUSG00000000359	2.26	1.58778E-07
ENSMUSG00000051225	2.26	3.84871E-08
ENSMUSG00000031146	2.26	0.00010153
ENSMUSG00000043157	2.26	5.95586E-06
ENSMUSG00000078922	2.26	0.036695999
ENSMUSG00000067377	2.25	2.63546E-05
ENSMUSG00000019853	2.25	0.007779761
ENSMUSG00000060550	2.25	1.8196E-18
ENSMUSG00000085761	2.25	0.004467566
ENSMUSG00000021196	2.25	3.16029E-12
ENSMUSG00000062510	2.25	3.76988E-06
ENSMUSG00000027611	2.25	2.22686E-10
ENSMUSG00000058715	2.24	9.49255E-14
ENSMUSG00000026981	2.24	2.53504E-13
ENSMUSG00000078763	2.24	0.012237574
ENSMUSG00000036896	2.24	8.20396E-08
ENSMUSG00000056671	2.24	3.34578E-05
ENSMUSG00000026355	2.24	4.51848E-06
ENSMUSG00000040690	2.24	2.92264E-14
ENSMUSG00000079442	2.24	6.51424E-08
ENSMUSG00000012443	2.24	0.000905116
ENSMUSG00000027456	2.24	0.004769237
ENSMUSG00000054871	2.24	2.49815E-05
ENSMUSG00000027204	2.24	8.87512E-16
ENSMUSG00000020423	2.24	4.02848E-06
ENSMUSG00000003779	2.24	0.042632437
ENSMUSG00000004098	2.23	3.11852E-10
ENSMUSG00000038530	2.23	0.01592584
ENSMUSG00000015947	2.23	1.06575E-05
ENSMUSG00000039208	2.23	3.35711E-14
ENSMUSG00000024036	2.22	1.3018E-11
ENSMUSG00000032093	2.22	0.016983315
ENSMUSG00000030107	2.22	0.026050576

Ensembl gene id	log2FC	p-adjusted
ENSMUSG00000050288	2.22	0.013138168
ENSMUSG00000061878	2.22	7.9564E-06
ENSMUSG00000020178	2.22	1.80366E-10
ENSMUSG00000033847	2.22	0.016132909
ENSMUSG00000053113	2.22	1.31056E-15
ENSMUSG00000038086	2.22	0.004185368
ENSMUSG00000001281	2.21	2.15335E-13
ENSMUSG00000036634	2.21	0.001297709
ENSMUSG00000045999	2.21	0.006146233
ENSMUSG00000037060	2.21	2.40449E-06
ENSMUSG00000037995	2.21	0.014199289
ENSMUSG00000034041	2.21	0.000240536
ENSMUSG00000097910	2.21	0.034903589
ENSMUSG00000061048	2.21	0.00019123
ENSMUSG00000023827	2.21	1.47943E-06
ENSMUSG00000044199	2.20	1.14243E-05
ENSMUSG00000032053	2.20	1.28724E-08
ENSMUSG00000048521	2.20	0.000180069
ENSMUSG00000020689	2.20	1.07013E-06
ENSMUSG00000026566	2.20	6.79203E-16
ENSMUSG00000006538	2.20	0.000133256
ENSMUSG00000033952	2.20	0.018098279
ENSMUSG00000015437	2.20	0.006459975
ENSMUSG00000020330	2.20	0.008550047
ENSMUSG00000033287	2.20	8.1062E-13
ENSMUSG00000014030	2.20	0.00035368
ENSMUSG00000027239	2.20	0.006296635
ENSMUSG00000094526	2.20	0.006791321
ENSMUSG00000042759	2.20	3.81271E-12
ENSMUSG00000026748	2.20	1.24347E-11
ENSMUSG00000026126	2.20	9.83241E-10
ENSMUSG00000087213	2.19	0.008466036
ENSMUSG00000000409	2.19	1.65864E-08
ENSMUSG00000050357	2.19	2.79846E-05
ENSMUSG00000000903	2.19	0.005455945
ENSMUSG00000038400	2.19	6.09303E-05
ENSMUSG00000032254	2.19	0.000576952
ENSMUSG00000021569	2.19	0.025970541
ENSMUSG00000042918	2.19	0.006959125
ENSMUSG00000000386	2.19	0.00322535
ENSMUSG00000070501	2.19	0.00833809
ENSMUSG00000034116	2.18	6.29884E-15
ENSMUSG00000013921	2.18	4.32511E-05
ENSMUSG00000029553	2.18	1.54133E-13
ENSMUSG00000048442	2.18	0.034655682
ENSMUSG00000024885	2.18	3.11179E-11
ENSMUSG00000021493	2.18	9.0471E-14
ENSMUSG00000029275	2.18	0.005894191
ENSMUSG00000043263	2.18	0.015687187
ENSMUSG00000020798	2.18	0.009500077
ENSMUSG00000021253	2.18	3.64088E-10
ENSMUSG00000048163	2.18	9.59962E-15

Ensembl gene id	log2FC	p-adjusted
ENSMUSG00000030790	2.18	1.82631E-08
ENSMUSG00000022436	2.18	2.75112E-12
ENSMUSG00000032643	2.17	1.24033E-06
ENSMUSG00000041859	2.17	0.001995858
ENSMUSG00000051212	2.17	5.03718E-05
ENSMUSG00000045725	2.17	0.005357126
ENSMUSG00000029844	2.17	0.006246104
ENSMUSG00000031502	2.17	4.78053E-18
ENSMUSG00000039903	2.17	0.043445085
ENSMUSG00000026278	2.17	1.18024E-13
ENSMUSG00000031825	2.17	0.039995799
ENSMUSG00000002033	2.16	0.000292117
ENSMUSG00000047675	2.16	0.00067959
ENSMUSG00000015745	2.16	3.06171E-11
ENSMUSG00000032085	2.16	0.00221969
ENSMUSG00000025877	2.16	1.30201E-10
ENSMUSG00000024030	2.16	1.50546E-15
ENSMUSG00000011751	2.16	0.003745722
ENSMUSG00000035232	2.16	4.82994E-08
ENSMUSG00000021318	2.16	0.000405907
ENSMUSG00000007039	2.16	2.76913E-06
ENSMUSG00000034413	2.16	0.011320835
ENSMUSG00000050241	2.16	0.030990896
ENSMUSG00000022489	2.16	8.64084E-08
ENSMUSG00000042109	2.16	0.000164147
ENSMUSG00000073409	2.16	3.86024E-17
ENSMUSG00000022831	2.16	5.4415E-16
ENSMUSG00000074578	2.15	3.75126E-10
ENSMUSG00000049892	2.15	0.000134809
ENSMUSG00000024338	2.15	2.59912E-05
ENSMUSG00000050953	2.15	4.36839E-15
ENSMUSG00000029436	2.15	2.44776E-05
ENSMUSG00000007041	2.15	4.66701E-17
ENSMUSG00000028927	2.15	2.9221E-06
ENSMUSG00000062939	2.15	2.02444E-06
ENSMUSG00000015396	2.15	6.51757E-13
ENSMUSG00000030156	2.15	0.000652946
ENSMUSG00000072082	2.15	0.000715805
ENSMUSG00000033436	2.14	1.09934E-12
ENSMUSG00000046402	2.14	0.00169734
ENSMUSG00000048191	2.14	0.000174765
ENSMUSG00000024737	2.14	2.17879E-15
ENSMUSG00000052248	2.14	0.001466236
ENSMUSG00000094162	2.14	0.009523953
ENSMUSG00000089944	2.14	0.011616034
ENSMUSG00000051457	2.14	1.20764E-05
ENSMUSG00000003411	2.14	0.00026855
ENSMUSG00000011008	2.14	8.26032E-05
ENSMUSG00000022221	2.14	1.08331E-10
ENSMUSG00000028460	2.14	0.041936702
ENSMUSG00000024459	2.14	0.005576644
ENSMUSG00000030796	2.14	8.73344E-08

Ensembl gene id	log2FC	p-adjusted
ENSMUSG00000001473	2.13	5.26377E-07
ENSMUSG00000028597	2.13	0.000109277
ENSMUSG00000020728	2.13	0.008682568
ENSMUSG00000030325	2.13	0.020477291
ENSMUSG00000048807	2.13	5.33192E-05
ENSMUSG00000009876	2.13	0.005360468
ENSMUSG00000026421	2.13	1.6211E-07
ENSMUSG00000047953	2.13	0.021201334
ENSMUSG00000072694	2.12	7.94237E-07
ENSMUSG00000051735	2.12	2.93887E-11
ENSMUSG00000024334	2.12	0.003840743
ENSMUSG00000002058	2.12	2.25609E-12
ENSMUSG00000044258	2.12	4.81955E-10
ENSMUSG00000022667	2.12	3.2278E-08
ENSMUSG00000018008	2.12	1.21462E-15
ENSMUSG00000038811	2.12	2.3881E-06
ENSMUSG00000004665	2.12	9.19111E-15
ENSMUSG00000022863	2.12	0.018381008
ENSMUSG00000024803	2.12	0.001552391
ENSMUSG00000063193	2.11	8.1062E-13
ENSMUSG00000030605	2.11	2.75279E-15
ENSMUSG00000028874	2.11	5.05064E-13
ENSMUSG00000020722	2.11	0.017663404
ENSMUSG00000053101	2.11	0.043875686
ENSMUSG00000036931	2.11	9.4581E-05
ENSMUSG00000035274	2.11	0.041736896
ENSMUSG00000022103	2.11	3.5774E-09
ENSMUSG00000014543	2.11	0.008577118
ENSMUSG00000037202	2.11	0.001459802
ENSMUSG00000028044	2.10	1.26906E-10
ENSMUSG00000021175	2.10	0.006809403
ENSMUSG00000097415	2.10	5.77078E-08
ENSMUSG00000016458	2.10	6.68227E-06
ENSMUSG00000030579	2.10	1.02799E-15
ENSMUSG00000040276	2.10	0.005740554
ENSMUSG00000059336	2.10	0.021334256
ENSMUSG00000097364	2.10	0.024408873
ENSMUSG00000026796	2.09	3.36591E-14
ENSMUSG00000024679	2.09	6.5477E-09
ENSMUSG00000027398	2.09	4.2233E-13
ENSMUSG00000016529	2.09	0.0053772
ENSMUSG00000025140	2.09	0.003158386
ENSMUSG00000057135	2.09	4.24856E-11
ENSMUSG00000067341	2.09	0.021412115
ENSMUSG00000053977	2.09	0.017639659
ENSMUSG00000036905	2.09	6.63079E-16
ENSMUSG00000031264	2.09	1.19837E-10
ENSMUSG00000001493	2.09	0.003389304
ENSMUSG00000041449	2.09	3.03926E-12
ENSMUSG00000002983	2.08	6.6368E-10
ENSMUSG00000028885	2.08	0.010325062
ENSMUSG00000034686	2.08	0.005831621

Ensembl gene id	log2FC	p-adjusted
ENSMUSG00000086591	2.08	0.035362878
ENSMUSG00000001082	2.08	4.39052E-11
ENSMUSG00000027347	2.08	6.55473E-12
ENSMUSG00000034156	2.08	0.014274159
ENSMUSG00000040415	2.08	4.23621E-12
ENSMUSG00000038418	2.08	9.43847E-05
ENSMUSG00000020846	2.07	5.24801E-08
ENSMUSG00000028270	2.07	1.59821E-05
ENSMUSG00000049625	2.07	1.6139E-13
ENSMUSG00000066363	2.07	1.86119E-15
ENSMUSG00000029925	2.07	1.59228E-07
ENSMUSG00000036672	2.07	1.16005E-06
ENSMUSG00000015981	2.06	0.000907672
ENSMUSG00000008193	2.06	8.33485E-08
ENSMUSG00000039264	2.06	9.03325E-08
ENSMUSG00000068227	2.06	3.39772E-05
ENSMUSG00000043122	2.06	9.83165E-06
ENSMUSG00000093565	2.06	0.001488978
ENSMUSG00000025128	2.05	0.042447214
ENSMUSG00000074364	2.05	7.07875E-13
ENSMUSG00000039131	2.05	0.01454365
ENSMUSG00000029304	2.05	2.75136E-16
ENSMUSG00000003500	2.05	4.47993E-07
ENSMUSG00000092486	2.05	0.00015756
ENSMUSG00000066456	2.05	0.002054642
ENSMUSG00000040618	2.05	1.02559E-09
ENSMUSG00000026825	2.05	0.010825662
ENSMUSG00000093916	2.05	0.000514119
ENSMUSG00000048965	2.04	0.000157913
ENSMUSG00000028010	2.04	5.33599E-11
ENSMUSG00000057933	2.04	0.005099841
ENSMUSG00000022372	2.04	1.8201E-07
ENSMUSG00000004952	2.04	8.76029E-11
ENSMUSG00000047592	2.03	0.011109829
ENSMUSG00000010021	2.03	0.000485606
ENSMUSG00000003873	2.03	2.75417E-14
ENSMUSG00000030878	2.03	0.001445906
ENSMUSG00000070003	2.03	1.03381E-11
ENSMUSG00000028581	2.03	6.69948E-16
ENSMUSG00000037280	2.03	7.14032E-09
ENSMUSG00000074813	2.03	0.000587927
ENSMUSG00000033685	2.03	5.23221E-16
ENSMUSG00000030798	2.03	3.2519E-06
ENSMUSG00000024301	2.03	0.042292125
ENSMUSG00000000861	2.03	0.008548543
ENSMUSG00000018920	2.03	2.02147E-14
ENSMUSG00000036599	2.03	3.27304E-09
ENSMUSG00000032245	2.03	1.53454E-11
ENSMUSG00000076928	2.03	0.007814408
ENSMUSG00000021702	2.03	0.008361377
ENSMUSG00000042379	2.02	0.014532908
ENSMUSG00000052160	2.02	4.19655E-15

Ensembl gene id	log2FC	p-adjusted
ENSMUSG00000040711	2.02	2.41935E-10
ENSMUSG00000049866	2.02	1.18809E-12
ENSMUSG00000002111	2.02	7.5149E-14
ENSMUSG00000049130	2.02	0.001195585
ENSMUSG00000006589	2.02	7.32108E-07
ENSMUSG00000031756	2.02	0.004325729
ENSMUSG00000024663	2.02	6.83006E-13
ENSMUSG00000047810	2.02	6.9882E-12
ENSMUSG00000041515	2.02	2.93706E-14
ENSMUSG00000092550	2.02	0.017912987
ENSMUSG00000020108	2.02	1.57231E-07
ENSMUSG00000033467	2.02	6.78919E-10
ENSMUSG00000052760	2.02	1.14957E-06
ENSMUSG00000074141	2.02	1.56932E-08
ENSMUSG00000040345	2.02	2.25291E-11
ENSMUSG00000019823	2.02	1.80117E-08
ENSMUSG00000048450	2.01	0.032393305
ENSMUSG00000030047	2.01	8.07062E-12
ENSMUSG00000035439	2.01	3.21085E-05
ENSMUSG00000076437	2.01	0.002130545
ENSMUSG00000037321	2.01	5.82635E-05
ENSMUSG00000017861	2.01	0.001028943
ENSMUSG00000056612	2.01	1.3907E-10
ENSMUSG00000031995	2.01	0.004063061
ENSMUSG00000034059	2.01	0.008225015
ENSMUSG00000018774	2.01	2.7175E-14
ENSMUSG00000037613	2.01	3.35718E-06
ENSMUSG00000034959	2.01	0.000810631
ENSMUSG00000090877	2.00	0.017275733
ENSMUSG00000078122	2.00	6.71274E-06
ENSMUSG00000041219	2.00	0.012007912
ENSMUSG00000024300	2.00	1.96808E-13
ENSMUSG00000018507	2.00	2.33441E-10
ENSMUSG00000029449	2.00	3.75665E-05
ENSMUSG00000044337	2.00	5.42332E-08
ENSMUSG00000002297	2.00	0.000100185
ENSMUSG00000022952	1.99	5.18614E-08
ENSMUSG00000026821	1.99	7.46502E-11
ENSMUSG00000043079	1.99	1.93789E-13
ENSMUSG00000021795	1.99	0.006514891
ENSMUSG00000090698	1.99	0.027794143
ENSMUSG00000023004	1.99	2.55798E-09
ENSMUSG00000058794	1.99	0.000699485
ENSMUSG00000031722	1.99	0.011552938
ENSMUSG00000053310	1.99	0.005301743
ENSMUSG00000032491	1.99	0.000696772
ENSMUSG00000059901	1.99	5.85824E-08
ENSMUSG00000029561	1.99	0.016961134
ENSMUSG00000016526	1.99	0.008696785
ENSMUSG00000023078	1.99	0.016810285
ENSMUSG00000022586	1.98	0.015316421
ENSMUSG00000010660	1.98	0.000514295

Ensembl gene id	log2FC	p-adjusted
ENSMUSG00000002603	1.98	2.72445E-13
ENSMUSG00000030403	1.98	2.3592E-14
ENSMUSG00000027298	1.98	0.000149786
ENSMUSG00000071552	1.98	0.012772917
ENSMUSG00000079363	1.98	0.038508026
ENSMUSG00000013155	1.98	0.002921307
ENSMUSG00000027955	1.97	8.54922E-08
ENSMUSG00000078439	1.97	0.00288351
ENSMUSG00000021880	1.97	0.000149127
ENSMUSG00000036545	1.97	1.26702E-12
ENSMUSG00000063458	1.97	0.000295295
ENSMUSG00000023009	1.97	6.14341E-07
ENSMUSG00000015312	1.97	7.68937E-05
ENSMUSG00000056069	1.97	1.57332E-10
ENSMUSG00000059326	1.97	2.37507E-11
ENSMUSG00000032246	1.97	8.84225E-09
ENSMUSG00000031971	1.97	0.003437393
ENSMUSG00000046058	1.97	0.001331935
ENSMUSG00000006344	1.96	5.00089E-10
ENSMUSG00000086763	1.96	0.000606183
ENSMUSG00000096472	1.96	3.68494E-08
ENSMUSG00000041707	1.96	6.09216E-07
ENSMUSG00000031004	1.96	0.000503058
ENSMUSG00000062593	1.96	2.41488E-10
ENSMUSG00000034039	1.96	0.006687157
ENSMUSG00000048583	1.96	0.003608874
ENSMUSG00000072941	1.96	1.28013E-14
ENSMUSG00000021451	1.96	0.000164759
ENSMUSG00000092517	1.96	0.004042538
ENSMUSG00000079014	1.96	1.79162E-10
ENSMUSG00000070526	1.96	0.009893763
ENSMUSG00000020886	1.95	5.76257E-07
ENSMUSG00000059108	1.95	0.04276398
ENSMUSG00000033450	1.95	0.004545429
ENSMUSG00000097180	1.95	0.009945831
ENSMUSG00000013584	1.95	0.000303916
ENSMUSG00000059498	1.95	4.7059E-13
ENSMUSG00000033059	1.95	8.65652E-13
ENSMUSG00000072235	1.95	1.63562E-08
ENSMUSG00000021391	1.95	0.002106043
ENSMUSG00000073489	1.95	0.010822772
ENSMUSG00000022613	1.95	0.001926459
ENSMUSG00000032640	1.95	2.88613E-09
ENSMUSG00000020901	1.95	4.37964E-09
ENSMUSG00000025008	1.94	1.12502E-05
ENSMUSG00000069917	1.94	0.003213192
ENSMUSG00000049988	1.94	9.20907E-11
ENSMUSG00000002997	1.94	0.02101308
ENSMUSG00000034427	1.94	0.002128707
ENSMUSG00000032271	1.94	0.005833713
ENSMUSG00000034520	1.94	0.00049055
ENSMUSG00000037902	1.94	1.42845E-14

Ensembl gene id	log2FC	p-adjusted
ENSMUSG00000045211	1.93	1.40809E-07
ENSMUSG00000000628	1.93	3.58889E-09
ENSMUSG00000038155	1.93	0.002739401
ENSMUSG00000052270	1.93	0.000497604
ENSMUSG00000019773	1.93	5.17485E-06
ENSMUSG00000071005	1.93	0.003463853
ENSMUSG00000024659	1.93	6.95923E-12
ENSMUSG00000056498	1.93	0.000100996
ENSMUSG00000020737	1.92	6.44487E-13
ENSMUSG00000000901	1.92	0.000469641
ENSMUSG00000030695	1.92	2.79844E-14
ENSMUSG00000043557	1.92	0.003242585
ENSMUSG00000034457	1.92	0.005390856
ENSMUSG00000054555	1.92	4.93088E-05
ENSMUSG00000074874	1.92	5.31448E-11
ENSMUSG00000000489	1.92	1.01515E-07
ENSMUSG00000034595	1.92	1.29027E-12
ENSMUSG00000045664	1.92	1.04393E-09
ENSMUSG00000037594	1.92	0.000116485
ENSMUSG00000037759	1.92	5.3465E-06
ENSMUSG00000023277	1.91	1.05345E-10
ENSMUSG00000021250	1.91	0.023084845
ENSMUSG00000030365	1.91	0.001713404
ENSMUSG00000028883	1.91	0.000848634
ENSMUSG00000046491	1.91	0.006746068
ENSMUSG00000026785	1.91	3.65577E-06
ENSMUSG00000025582	1.91	0.009333201
ENSMUSG00000030217	1.91	1.50992E-07
ENSMUSG00000070427	1.91	7.17134E-14
ENSMUSG00000032332	1.91	2.30028E-09
ENSMUSG00000003379	1.90	0.012802377
ENSMUSG00000002458	1.90	1.40691E-09
ENSMUSG00000019194	1.90	9.84981E-11
ENSMUSG00000008734	1.90	0.000339736
ENSMUSG00000028268	1.90	0.011690805
ENSMUSG00000022639	1.90	0.01002603
ENSMUSG00000042106	1.90	0.000546756
ENSMUSG00000022090	1.90	9.36892E-07
ENSMUSG00000091649	1.90	0.027674417
ENSMUSG00000018983	1.90	1.13277E-05
ENSMUSG00000060791	1.90	1.83265E-09
ENSMUSG00000020331	1.89	0.014710651
ENSMUSG00000022945	1.89	0.0014142
ENSMUSG00000030641	1.89	0.008243687
ENSMUSG00000023349	1.89	5.26278E-10
ENSMUSG00000097993	1.89	0.007504693
ENSMUSG00000005087	1.89	9.73844E-12
ENSMUSG00000021886	1.89	0.001061358
ENSMUSG00000049556	1.89	0.008816839
ENSMUSG00000042766	1.89	0.015519388
ENSMUSG00000041323	1.89	0.003319896
ENSMUSG00000030724	1.89	0.035375316

Ensembl gene id	log2FC	p-adjusted
ENSMUSG00000030218	1.89	0.038829437
ENSMUSG00000026972	1.89	1.7971E-08
ENSMUSG00000036894	1.89	4.31257E-09
ENSMUSG00000029674	1.88	6.8847E-06
ENSMUSG00000024401	1.88	0.002545927
ENSMUSG00000027326	1.88	0.007487484
ENSMUSG00000040264	1.88	0.01562403
ENSMUSG00000036596	1.88	0.008462767
ENSMUSG00000039187	1.88	0.036269862
ENSMUSG00000052821	1.88	2.123E-07
ENSMUSG00000030774	1.88	2.73183E-09
ENSMUSG00000020473	1.88	6.46579E-10
ENSMUSG00000076576	1.88	0.004941532
ENSMUSG00000057948	1.87	1.93082E-07
ENSMUSG00000025232	1.87	1.55802E-13
ENSMUSG00000021773	1.87	0.00019123
ENSMUSG00000025650	1.87	0.024432751
ENSMUSG00000014786	1.87	0.000817405
ENSMUSG00000055865	1.87	0.038957316
ENSMUSG00000061086	1.87	0.006297228
ENSMUSG00000025498	1.87	0.009522168
ENSMUSG00000091228	1.86	0.032622134
ENSMUSG00000024177	1.86	0.000138338
ENSMUSG00000062157	1.86	0.024330556
ENSMUSG00000084274	1.85	0.00035247
ENSMUSG00000032281	1.85	0.017288756
ENSMUSG00000063605	1.84	6.46133E-05
ENSMUSG00000024579	1.84	0.000330795
ENSMUSG00000045679	1.84	0.000514119
ENSMUSG00000040663	1.84	0.001050878
ENSMUSG00000029330	1.84	0.000115514
ENSMUSG00000003283	1.84	1.47013E-11
ENSMUSG00000053063	1.84	3.75059E-11
ENSMUSG00000020086	1.84	0.000742091
ENSMUSG00000026413	1.84	0.017414121
ENSMUSG00000000732	1.84	0.012857103
ENSMUSG00000094420	1.84	0.000515104
ENSMUSG00000034593	1.84	8.84987E-07
ENSMUSG00000050232	1.83	0.020534588
ENSMUSG00000026542	1.83	0.005050071
ENSMUSG00000050910	1.83	9.41893E-09
ENSMUSG00000037148	1.83	6.08206E-07
ENSMUSG00000049804	1.83	6.09835E-07
ENSMUSG00000038252	1.83	0.000215156
ENSMUSG00000035783	1.83	0.000978168
ENSMUSG00000027646	1.83	8.60859E-10
ENSMUSG00000097906	1.83	0.035703736
ENSMUSG00000012126	1.83	0.012777774
ENSMUSG00000049932	1.83	4.49544E-11
ENSMUSG00000033031	1.83	0.004524981
ENSMUSG00000030468	1.83	4.93057E-05
ENSMUSG00000030167	1.83	0.04276398

Ensembl gene id	log2FC	p-adjusted
ENSMUSG00000074743	1.83	1.22277E-05
ENSMUSG00000030022	1.83	5.83467E-07
ENSMUSG00000021948	1.82	4.06716E-12
ENSMUSG00000038860	1.82	0.02214649
ENSMUSG00000000093	1.82	1.69447E-09
ENSMUSG00000026581	1.82	3.14497E-08
ENSMUSG00000050075	1.82	0.004137577
ENSMUSG00000092203	1.82	2.99223E-10
ENSMUSG00000042436	1.82	0.001132377
ENSMUSG00000026072	1.82	2.83603E-07
ENSMUSG00000036459	1.82	6.02099E-08
ENSMUSG00000049037	1.82	2.15349E-09
ENSMUSG00000025889	1.82	0.004484673
ENSMUSG00000035458	1.82	0.016306094
ENSMUSG00000008384	1.82	4.53391E-10
ENSMUSG00000055541	1.82	4.20395E-05
ENSMUSG00000078853	1.82	0.005979944
ENSMUSG00000037474	1.82	0.032179833
ENSMUSG00000027330	1.81	8.51591E-08
ENSMUSG00000024013	1.81	8.25835E-10
ENSMUSG00000022324	1.81	1.66733E-06
ENSMUSG00000026676	1.81	0.00182727
ENSMUSG00000060803	1.81	0.005174544
ENSMUSG00000028613	1.81	0.004609754
ENSMUSG00000018593	1.81	3.0403E-13
ENSMUSG00000082809	1.81	0.025267147
ENSMUSG00000020641	1.81	0.027911527
ENSMUSG00000044339	1.81	5.37865E-08
ENSMUSG00000002870	1.81	0.006903094
ENSMUSG00000057322	1.80	0.001175035
ENSMUSG00000002900	1.80	1.40097E-11
ENSMUSG00000040219	1.80	0.018196304
ENSMUSG00000045404	1.80	1.30028E-05
ENSMUSG00000007783	1.80	0.001856342
ENSMUSG00000039232	1.80	0.000111388
ENSMUSG00000031375	1.80	2.41849E-10
ENSMUSG00000020120	1.80	6.97771E-11
ENSMUSG00000049744	1.80	4.80481E-05
ENSMUSG00000005763	1.80	0.012234884
ENSMUSG00000071713	1.80	7.75776E-11
ENSMUSG00000025058	1.80	0.003941104
ENSMUSG00000043832	1.79	1.60479E-08
ENSMUSG00000096956	1.79	0.000469387
ENSMUSG00000038390	1.79	0.004591261
ENSMUSG00000036036	1.79	0.000962788
ENSMUSG00000070802	1.79	0.024808535
ENSMUSG00000025648	1.79	6.01458E-06
ENSMUSG00000068086	1.79	0.015027875
ENSMUSG00000053158	1.79	2.41935E-10
ENSMUSG00000004864	1.79	0.00675508
ENSMUSG00000015027	1.79	1.10974E-08
ENSMUSG00000035105	1.78	0.003873186

Ensembl gene id	log2FC	p-adjusted
ENSMUSG00000046432	1.78	3.64363E-05
ENSMUSG00000021190	1.78	7.34696E-13
ENSMUSG00000027496	1.78	4.87918E-06
ENSMUSG00000062488	1.78	0.003765937
ENSMUSG00000020101	1.78	4.54478E-11
ENSMUSG00000021214	1.78	0.008699512
ENSMUSG00000049107	1.78	0.021300179
ENSMUSG00000021356	1.78	0.009266586
ENSMUSG00000086841	1.78	3.65242E-10
ENSMUSG00000043807	1.78	0.011963309
ENSMUSG00000020814	1.78	0.000388197
ENSMUSG00000004707	1.78	5.33998E-05
ENSMUSG00000052384	1.78	4.18486E-11
ENSMUSG00000029177	1.78	4.72477E-06
ENSMUSG00000031616	1.77	2.16071E-10
ENSMUSG00000035929	1.77	2.25606E-06
ENSMUSG00000049999	1.77	0.002344596
ENSMUSG00000000530	1.77	9.46155E-10
ENSMUSG00000052837	1.77	6.49289E-12
ENSMUSG00000027635	1.77	0.008726553
ENSMUSG00000026068	1.77	0.000615018
ENSMUSG00000028378	1.77	5.45795E-08
ENSMUSG00000061111	1.77	0.000151703
ENSMUSG00000037447	1.77	4.59203E-07
ENSMUSG00000027863	1.77	5.12046E-05
ENSMUSG00000027360	1.76	0.001163645
ENSMUSG00000055805	1.76	1.81002E-07
ENSMUSG00000034192	1.76	6.6978E-06
ENSMUSG00000055809	1.76	0.014709334
ENSMUSG00000029570	1.76	2.00177E-09
ENSMUSG00000095677	1.76	0.000435203
ENSMUSG00000068522	1.76	5.20705E-05
ENSMUSG00000032657	1.76	6.66514E-05
ENSMUSG00000026600	1.76	6.98542E-11
ENSMUSG00000027199	1.76	3.46902E-07
ENSMUSG00000041954	1.76	0.00119114
ENSMUSG00000040552	1.76	1.4468E-09
ENSMUSG00000039693	1.76	0.001504349
ENSMUSG00000086813	1.76	0.011512163
ENSMUSG00000031776	1.76	1.34639E-08
ENSMUSG00000020185	1.76	0.000637075
ENSMUSG00000020788	1.76	3.0967E-06
ENSMUSG00000078963	1.76	0.004292065
ENSMUSG00000037206	1.75	8.95451E-08
ENSMUSG00000019278	1.75	0.000201327
ENSMUSG00000001588	1.75	1.58575E-08
ENSMUSG00000037661	1.75	0.001420454
ENSMUSG00000026223	1.75	3.19432E-10
ENSMUSG00000050382	1.75	0.001206546
ENSMUSG00000026288	1.75	8.84543E-11
ENSMUSG00000006651	1.75	0.006084809
ENSMUSG00000026548	1.75	0.005028995

Ensembl gene id	log2FC	p-adjusted
ENSMUSG00000023274	1.75	0.000168868
ENSMUSG00000006356	1.74	5.48483E-12
ENSMUSG00000027544	1.74	0.001350431
ENSMUSG00000034206	1.74	0.005217807
ENSMUSG00000032400	1.74	0.009672143
ENSMUSG00000027514	1.74	0.013818969
ENSMUSG00000000317	1.74	4.92296E-08
ENSMUSG00000003534	1.74	4.46399E-05
ENSMUSG00000029723	1.74	2.85036E-10
ENSMUSG00000009013	1.74	6.50664E-11
ENSMUSG00000029413	1.74	8.24843E-09
ENSMUSG00000053656	1.74	2.39357E-05
ENSMUSG00000044548	1.73	0.000592797
ENSMUSG00000038352	1.73	0.00030947
ENSMUSG00000030329	1.73	0.005489252
ENSMUSG00000071547	1.73	4.29313E-08
ENSMUSG00000019235	1.73	0.000567099
ENSMUSG00000073411	1.73	3.83126E-12
ENSMUSG00000096255	1.73	0.001286257
ENSMUSG00000078780	1.73	3.25168E-05
ENSMUSG00000052151	1.73	1.46645E-10
ENSMUSG00000010307	1.73	8.33212E-08
ENSMUSG00000019997	1.72	0.004861464
ENSMUSG00000047250	1.72	4.10215E-11
ENSMUSG00000047415	1.72	0.004620361
ENSMUSG00000073982	1.72	9.6747E-11
ENSMUSG00000030772	1.72	8.24041E-09
ENSMUSG00000060216	1.72	4.82367E-11
ENSMUSG00000026009	1.71	8.55136E-05
ENSMUSG00000091971	1.71	0.019590256
ENSMUSG00000029082	1.71	0.007826895
ENSMUSG00000036172	1.71	0.010114743
ENSMUSG00000040441	1.71	0.028896728
ENSMUSG00000056367	1.71	0.030990896
ENSMUSG00000005958	1.71	0.001673
ENSMUSG00000051855	1.71	0.001878633
ENSMUSG00000039936	1.70	3.64088E-10
ENSMUSG00000031897	1.70	0.002275872
ENSMUSG00000020732	1.70	0.007156833
ENSMUSG00000034353	1.70	1.01845E-06
ENSMUSG00000074579	1.70	0.011336748
ENSMUSG00000054400	1.70	0.00745143
ENSMUSG00000043448	1.69	0.003722817
ENSMUSG00000025290	1.69	0.018373499
ENSMUSG00000026832	1.69	8.66953E-05
ENSMUSG00000064043	1.69	0.001915678
ENSMUSG00000064264	1.69	0.000150162
ENSMUSG00000024736	1.69	1.58128E-05
ENSMUSG00000007892	1.69	3.1358E-05
ENSMUSG00000078771	1.69	4.75207E-07
ENSMUSG00000050989	1.69	1.75929E-08
ENSMUSG00000000740	1.69	0.000318798

Ensembl gene id	log2FC	p-adjusted
ENSMUSG00000035493	1.69	1.34313E-11
ENSMUSG00000025875	1.68	3.82233E-05
ENSMUSG00000071078	1.68	0.000106585
ENSMUSG00000051343	1.68	1.92307E-06
ENSMUSG00000005397	1.68	1.2839E-06
ENSMUSG00000034652	1.68	5.61127E-10
ENSMUSG00000038151	1.68	0.000111399
ENSMUSG00000078247	1.68	0.005661915
ENSMUSG00000032816	1.68	0.005336545
ENSMUSG00000028159	1.68	3.91869E-06
ENSMUSG00000024851	1.68	6.71678E-09
ENSMUSG00000015950	1.67	3.68427E-09
ENSMUSG00000022014	1.67	0.002701095
ENSMUSG00000030782	1.67	4.31501E-07
ENSMUSG00000038156	1.67	2.14416E-05
ENSMUSG00000048537	1.67	3.86201E-08
ENSMUSG00000004044	1.67	6.10709E-10
ENSMUSG00000035863	1.67	5.25823E-05
ENSMUSG00000023911	1.67	0.017384322
ENSMUSG00000046157	1.67	2.65083E-07
ENSMUSG00000001227	1.67	0.000238101
ENSMUSG00000039899	1.67	0.00073142
ENSMUSG00000040990	1.67	5.21663E-08
ENSMUSG00000006930	1.67	1.43927E-06
ENSMUSG00000026274	1.67	0.006113804
ENSMUSG00000074896	1.67	0.027077907
ENSMUSG00000060131	1.66	7.01097E-05
ENSMUSG00000026817	1.66	0.000585965
ENSMUSG00000089665	1.66	0.021532758
ENSMUSG00000062545	1.66	8.9517E-08
ENSMUSG00000025185	1.66	0.010099315
ENSMUSG00000018169	1.66	0.000147744
ENSMUSG00000040528	1.66	0.00012552
ENSMUSG00000064326	1.66	0.000189993
ENSMUSG00000022057	1.66	1.47146E-08
ENSMUSG00000021696	1.66	0.011485513
ENSMUSG00000030428	1.66	0.008882756
ENSMUSG00000032661	1.66	0.011351962
ENSMUSG00000024940	1.66	2.84231E-07
ENSMUSG00000032344	1.66	0.017736015
ENSMUSG00000025163	1.66	0.000120807
ENSMUSG00000029007	1.66	1.20005E-08
ENSMUSG00000019850	1.66	0.019664391
ENSMUSG00000031903	1.66	1.76848E-10
ENSMUSG00000001948	1.65	0.005003069
ENSMUSG00000063146	1.65	3.64854E-08
ENSMUSG00000069662	1.65	6.3973E-11
ENSMUSG00000039252	1.65	0.040600153
ENSMUSG00000028019	1.65	5.771E-08
ENSMUSG00000028859	1.65	1.13647E-06
ENSMUSG00000037318	1.65	2.56138E-05
ENSMUSG00000027293	1.65	3.19267E-07

Ensembl gene id	log2FC	p-adjusted
ENSMUSG00000060019	1.65	4.05211E-06
ENSMUSG00000032802	1.65	0.003622267
ENSMUSG00000032300	1.65	2.66067E-08
ENSMUSG00000030844	1.65	6.15725E-07
ENSMUSG00000048376	1.65	0.000605486
ENSMUSG00000051220	1.64	0.025945698
ENSMUSG00000091021	1.64	0.021684193
ENSMUSG00000033066	1.64	4.43066E-07
ENSMUSG00000015355	1.64	1.03201E-07
ENSMUSG00000092074	1.64	0.002435025
ENSMUSG00000090213	1.64	3.76343E-08
ENSMUSG00000027843	1.64	0.000275206
ENSMUSG00000078453	1.64	2.17446E-08
ENSMUSG00000016206	1.64	1.70881E-07
ENSMUSG00000044562	1.64	3.25514E-05
ENSMUSG00000051998	1.64	0.001514168
ENSMUSG00000027530	1.64	0.016774243
ENSMUSG00000036718	1.64	8.98743E-07
ENSMUSG00000070476	1.64	0.000288667
ENSMUSG00000027859	1.63	8.85935E-05
ENSMUSG00000035711	1.63	6.05124E-05
ENSMUSG00000026180	1.63	0.001082121
ENSMUSG00000097769	1.63	0.002080996
ENSMUSG00000028645	1.63	2.99523E-07
ENSMUSG00000001128	1.63	1.08294E-10
ENSMUSG00000047676	1.63	0.026027121
ENSMUSG00000024063	1.63	1.43571E-09
ENSMUSG00000043004	1.63	1.49096E-07
ENSMUSG00000097188	1.63	9.38295E-09
ENSMUSG00000037972	1.63	5.7633E-07
ENSMUSG00000026121	1.63	4.66414E-08
ENSMUSG00000026249	1.63	0.001261384
ENSMUSG00000032091	1.63	0.010612855
ENSMUSG00000067608	1.63	0.000872471
ENSMUSG00000031565	1.63	1.36544E-07
ENSMUSG00000032089	1.63	3.53249E-09
ENSMUSG00000002059	1.62	2.03517E-06
ENSMUSG00000005447	1.62	1.97546E-05
ENSMUSG00000030786	1.62	3.23759E-07
ENSMUSG00000028634	1.62	1.21744E-05
ENSMUSG00000059195	1.62	0.009443641
ENSMUSG00000095098	1.62	0.021576992
ENSMUSG00000020656	1.62	2.8024E-05
ENSMUSG00000037012	1.62	1.22508E-05
ENSMUSG00000031504	1.62	1.17372E-07
ENSMUSG00000012017	1.62	9.97353E-08
ENSMUSG00000049517	1.62	0.00255345
ENSMUSG00000025324	1.62	0.040775687
ENSMUSG00000023034	1.61	0.010491445
ENSMUSG00000063160	1.61	0.000536245
ENSMUSG00000011884	1.61	1.04063E-08
ENSMUSG00000028197	1.61	0.013204137

Ensembl gene id	log2FC	p-adjusted
ENSMUSG00000035121	1.61	0.008656518
ENSMUSG00000031342	1.61	0.001300599
ENSMUSG00000037601	1.61	9.99761E-07
ENSMUSG00000085295	1.61	0.020174921
ENSMUSG00000020395	1.61	0.024526776
ENSMUSG00000006342	1.61	1.24033E-06
ENSMUSG00000075266	1.61	0.000577441
ENSMUSG00000024352	1.61	0.005489252
ENSMUSG00000027297	1.61	0.005155419
ENSMUSG00000018459	1.61	0.000481675
ENSMUSG00000030651	1.61	0.041062217
ENSMUSG00000094344	1.60	0.03022004
ENSMUSG00000030263	1.60	4.26822E-08
ENSMUSG00000051504	1.60	0.01914001
ENSMUSG00000087273	1.60	0.009660827
ENSMUSG00000040891	1.60	3.90095E-10
ENSMUSG00000034892	1.60	0.000982909
ENSMUSG00000041313	1.60	0.000183986
ENSMUSG00000025508	1.60	0.000762386
ENSMUSG00000057092	1.60	0.013952355
ENSMUSG00000059049	1.60	0.036478805
ENSMUSG00000052684	1.60	0.003879459
ENSMUSG00000031799	1.60	4.4869E-10
ENSMUSG00000035713	1.60	0.00169734
ENSMUSG00000043099	1.60	1.95226E-07
ENSMUSG00000061578	1.59	0.006977638
ENSMUSG00000056501	1.59	1.78011E-09
ENSMUSG00000042041	1.59	2.1643E-09
ENSMUSG00000050350	1.59	0.018170298
ENSMUSG00000027712	1.59	3.13102E-10
ENSMUSG00000028369	1.59	0.000232153
ENSMUSG00000020186	1.59	0.000535184
ENSMUSG00000089726	1.59	5.14234E-05
ENSMUSG00000035891	1.59	2.20095E-08
ENSMUSG00000090084	1.58	0.004749704
ENSMUSG00000025491	1.58	6.79916E-05
ENSMUSG00000057604	1.58	0.001297943
ENSMUSG00000069919	1.58	0.003060723
ENSMUSG00000046711	1.58	0.000862009
ENSMUSG00000042510	1.58	0.004173706
ENSMUSG00000031103	1.58	6.68671E-08
ENSMUSG00000068245	1.58	0.014400991
ENSMUSG00000031548	1.58	3.92002E-05
ENSMUSG00000020773	1.58	9.35963E-08
ENSMUSG00000033307	1.58	0.003220766
ENSMUSG00000023947	1.58	1.01602E-06
ENSMUSG00000036526	1.58	0.006656063
ENSMUSG00000031875	1.58	9.15372E-09
ENSMUSG00000027636	1.58	0.027697566
ENSMUSG00000021943	1.57	0.007236284
ENSMUSG00000093880	1.57	0.006933239
ENSMUSG00000039316	1.57	3.40556E-07

Ensembl gene id	log2FC	p-adjusted
ENSMUSG00000015053	1.57	0.00353603
ENSMUSG00000032020	1.57	2.34825E-05
ENSMUSG00000037103	1.57	9.81808E-08
ENSMUSG00000031848	1.57	7.63517E-07
ENSMUSG00000024556	1.57	8.55495E-07
ENSMUSG00000002006	1.57	0.023148379
ENSMUSG00000054428	1.57	0.000207379
ENSMUSG00000029580	1.57	1.45994E-10
ENSMUSG00000004100	1.57	2.17762E-08
ENSMUSG00000058818	1.57	4.85907E-08
ENSMUSG00000039001	1.56	0.002172399
ENSMUSG00000020895	1.56	0.000533643
ENSMUSG00000050721	1.56	2.74891E-09
ENSMUSG00000049687	1.56	0.034971455
ENSMUSG00000034845	1.56	4.29681E-10
ENSMUSG00000017734	1.56	0.007126473
ENSMUSG00000010067	1.56	4.89193E-06
ENSMUSG00000038421	1.56	0.004929516
ENSMUSG00000023755	1.56	0.008829991
ENSMUSG00000002844	1.56	4.17527E-08
ENSMUSG00000025352	1.56	0.013271288
ENSMUSG00000038193	1.56	1.8463E-07
ENSMUSG00000023043	1.56	7.28757E-08
ENSMUSG00000034664	1.56	0.00957216
ENSMUSG00000028884	1.55	0.000931249
ENSMUSG00000038147	1.55	2.24846E-08
ENSMUSG00000054364	1.55	1.1102E-09
ENSMUSG00000076137	1.55	0.019945101
ENSMUSG00000015094	1.55	2.65875E-07
ENSMUSG00000029070	1.55	6.36524E-08
ENSMUSG00000052146	1.55	1.43222E-05
ENSMUSG00000031494	1.55	0.006545303
ENSMUSG00000028923	1.55	2.75649E-08
ENSMUSG00000009585	1.55	3.43936E-07
ENSMUSG00000028687	1.55	0.005556583
ENSMUSG00000025758	1.55	0.005654467
ENSMUSG00000086859	1.55	0.00743266
ENSMUSG00000074063	1.55	5.80671E-10
ENSMUSG00000012519	1.55	0.005686447
ENSMUSG00000050234	1.54	3.86104E-06
ENSMUSG00000015854	1.54	5.79182E-10
ENSMUSG00000074873	1.54	2.27305E-05
ENSMUSG00000038644	1.54	1.01475E-06
ENSMUSG00000027670	1.54	0.012096461
ENSMUSG00000026586	1.54	0.001314445
ENSMUSG00000026979	1.54	7.41615E-08
ENSMUSG00000020806	1.54	5.31378E-09
ENSMUSG00000046229	1.54	0.005378526
ENSMUSG00000059479	1.54	0.003567977
ENSMUSG00000006369	1.53	1.85013E-05
ENSMUSG00000025855	1.53	0.014054635
ENSMUSG00000043740	1.53	9.21067E-08

Ensembl gene id	log2FC	p-adjusted
ENSMUSG00000042842	1.53	4.3257E-05
ENSMUSG00000047721	1.53	0.000438848
ENSMUSG00000029135	1.53	4.97649E-08
ENSMUSG00000024590	1.53	0.002473189
ENSMUSG00000079227	1.53	5.14995E-09
ENSMUSG00000040502	1.53	0.0420101
ENSMUSG00000072812	1.53	0.007282903
ENSMUSG00000005054	1.53	4.66529E-07
ENSMUSG00000027555	1.53	0.001752872
ENSMUSG00000050921	1.53	0.002416093
ENSMUSG00000019874	1.53	6.66551E-08
ENSMUSG00000045362	1.53	0.001426629
ENSMUSG00000029762	1.52	6.11971E-05
ENSMUSG00000030245	1.52	1.31183E-07
ENSMUSG00000018648	1.52	0.008909422
ENSMUSG00000073940	1.52	0.004379173
ENSMUSG00000020018	1.52	1.85826E-05
ENSMUSG00000041827	1.52	0.00327874
ENSMUSG00000024843	1.52	0.001426878
ENSMUSG00000028337	1.52	0.000436998
ENSMUSG00000003206	1.52	0.012686075
ENSMUSG00000024053	1.52	9.97353E-08
ENSMUSG00000078249	1.52	0.004981298
ENSMUSG00000037731	1.51	3.2991E-08
ENSMUSG00000097039	1.51	0.01392837
ENSMUSG00000006586	1.51	0.00489364
ENSMUSG00000001525	1.51	1.1883E-09
ENSMUSG00000047832	1.51	1.09121E-06
ENSMUSG00000023909	1.51	1.77066E-05
ENSMUSG00000023021	1.51	2.17227E-06
ENSMUSG00000034220	1.51	7.27756E-06
ENSMUSG00000037499	1.51	0.007046004
ENSMUSG00000079003	1.51	1.791E-07
ENSMUSG00000025402	1.51	8.85058E-08
ENSMUSG00000016496	1.50	0.006971689
ENSMUSG00000031594	1.50	0.004618451
ENSMUSG00000001521	1.50	3.17768E-05
ENSMUSG00000025212	1.50	1.71674E-05
ENSMUSG00000031165	1.50	4.17209E-06
ENSMUSG00000049580	1.50	0.003832424
ENSMUSG00000019916	1.50	1.06389E-08
ENSMUSG00000024824	1.50	6.98165E-06
ENSMUSG00000086869	1.50	0.001550488
ENSMUSG00000038304	1.50	0.017178396
ENSMUSG00000034438	1.50	0.01648795
ENSMUSG00000038976	1.50	1.43158E-08
ENSMUSG00000049382	1.50	1.48979E-09
ENSMUSG00000020974	1.50	0.00485399
ENSMUSG00000041189	1.50	0.03007137
ENSMUSG00000028643	1.49	0.001735415
ENSMUSG00000041120	1.49	0.000222577
ENSMUSG00000030681	1.49	3.97577E-06

Ensembl gene id	log2FC	p-adjusted
ENSMUSG00000024937	1.49	2.41895E-08
ENSMUSG00000024620	1.49	3.04024E-08
ENSMUSG00000091705	1.49	0.026651543
ENSMUSG00000040747	1.49	1.7732E-08
ENSMUSG00000029730	1.49	0.000602566
ENSMUSG00000074170	1.49	1.57716E-06
ENSMUSG00000022074	1.49	0.005448134
ENSMUSG00000020681	1.49	0.000752283
ENSMUSG00000030839	1.49	4.14639E-05
ENSMUSG00000022978	1.49	2.303E-05
ENSMUSG00000097187	1.49	0.029762048
ENSMUSG00000027510	1.49	0.010429788
ENSMUSG00000029126	1.49	0.002450445
ENSMUSG00000024871	1.49	0.032782521
ENSMUSG00000032939	1.48	1.84221E-05
ENSMUSG00000037405	1.48	0.006156689
ENSMUSG00000037946	1.48	2.30165E-05
ENSMUSG00000021367	1.48	0.017506278
ENSMUSG00000025647	1.48	2.43487E-06
ENSMUSG00000020607	1.48	0.000155103
ENSMUSG00000019461	1.48	1.56955E-07
ENSMUSG00000052726	1.48	0.037987271
ENSMUSG00000033722	1.48	0.034394182
ENSMUSG00000022180	1.48	3.86201E-08
ENSMUSG00000026930	1.48	0.000221292
ENSMUSG00000074671	1.48	0.034719258
ENSMUSG00000046818	1.48	0.013415837
ENSMUSG00000029659	1.48	0.015330514
ENSMUSG00000020230	1.48	0.000381609
ENSMUSG00000040659	1.47	4.56461E-09
ENSMUSG00000097328	1.47	2.09799E-05
ENSMUSG00000020439	1.47	2.00469E-07
ENSMUSG00000089715	1.47	3.17223E-07
ENSMUSG00000001029	1.47	1.79382E-05
ENSMUSG00000068101	1.47	1.83731E-05
ENSMUSG00000067370	1.47	0.010623002
ENSMUSG00000098112	1.47	2.5236E-07
ENSMUSG00000042712	1.47	2.6791E-08
ENSMUSG00000002732	1.47	0.000117187
ENSMUSG00000028459	1.47	1.12636E-05
ENSMUSG00000062591	1.47	0.006283657
ENSMUSG00000061451	1.47	0.031826242
ENSMUSG00000055401	1.47	6.81111E-08
ENSMUSG00000045165	1.47	0.000374134
ENSMUSG00000040483	1.47	0.01160074
ENSMUSG00000022534	1.46	0.000468121
ENSMUSG00000052336	1.46	0.001199444
ENSMUSG00000038543	1.46	0.002711661
ENSMUSG00000027459	1.46	0.000252986
ENSMUSG00000022587	1.46	0.003854974
ENSMUSG00000098557	1.46	7.07753E-08
ENSMUSG00000037035	1.46	3.45548E-05

Ensembl gene id	log2FC	p-adjusted
ENSMUSG00000027995	1.46	4.21548E-06
ENSMUSG00000025888	1.46	2.25606E-06
ENSMUSG00000030577	1.46	0.000121447
ENSMUSG00000020424	1.46	0.000889322
ENSMUSG00000043415	1.46	0.028453351
ENSMUSG00000026110	1.46	0.001293137
ENSMUSG00000005947	1.46	0.030210208
ENSMUSG00000050592	1.46	2.07104E-05
ENSMUSG00000060860	1.46	1.79401E-05
ENSMUSG00000022969	1.46	2.10312E-07
ENSMUSG00000034255	1.46	2.21924E-05
ENSMUSG00000001240	1.46	2.39079E-07
ENSMUSG00000026321	1.46	0.001456187
ENSMUSG00000038260	1.45	0.002791903
ENSMUSG00000037217	1.45	0.00169734
ENSMUSG00000034684	1.45	4.71255E-07
ENSMUSG00000044703	1.45	0.024796436
ENSMUSG00000027777	1.45	0.001265564
ENSMUSG00000039452	1.45	0.00228375
ENSMUSG00000085887	1.44	0.004654615
ENSMUSG00000033768	1.44	0.002693522
ENSMUSG00000024521	1.44	0.000182971
ENSMUSG00000055184	1.44	0.005274561
ENSMUSG00000000817	1.44	0.044396995
ENSMUSG00000033538	1.44	6.16705E-05
ENSMUSG00000028633	1.44	0.013508364
ENSMUSG00000022285	1.44	1.69766E-08
ENSMUSG00000032034	1.44	0.041102348
ENSMUSG00000022146	1.44	3.58934E-06
ENSMUSG00000041378	1.44	0.001550637
ENSMUSG00000046718	1.44	0.043458455
ENSMUSG00000029715	1.44	4.04739E-06
ENSMUSG00000041117	1.44	0.000112764
ENSMUSG00000078190	1.43	0.008864299
ENSMUSG00000048497	1.43	6.8409E-05
ENSMUSG00000046731	1.43	2.31862E-05
ENSMUSG00000078773	1.43	0.014136802
ENSMUSG00000025154	1.43	4.66529E-07
ENSMUSG00000024270	1.43	1.30254E-05
ENSMUSG00000042254	1.43	0.005451008
ENSMUSG00000030159	1.43	1.965E-07
ENSMUSG00000073684	1.43	2.15473E-05
ENSMUSG00000026773	1.43	0.00036122
ENSMUSG00000022965	1.43	4.78767E-08
ENSMUSG00000042817	1.43	0.025237699
ENSMUSG00000056665	1.42	2.30758E-05
ENSMUSG00000055148	1.42	7.66661E-07
ENSMUSG00000029059	1.42	0.013419778
ENSMUSG00000045680	1.42	4.45642E-06
ENSMUSG00000021806	1.42	0.001972487
ENSMUSG00000090164	1.42	0.001082698
ENSMUSG00000025856	1.42	4.30418E-06

Ensembl gene id	log2FC	p-adjusted
ENSMUSG00000086316	1.42	6.66514E-05
ENSMUSG00000075122	1.42	0.009394602
ENSMUSG00000023885	1.42	2.53234E-06
ENSMUSG00000022438	1.42	1.17655E-06
ENSMUSG00000060950	1.42	5.98765E-06
ENSMUSG00000024742	1.42	0.008570264
ENSMUSG00000024245	1.42	0.006725851
ENSMUSG00000021457	1.42	1.57714E-07
ENSMUSG00000062995	1.42	0.000192577
ENSMUSG00000020277	1.42	0.037471308
ENSMUSG00000087150	1.42	0.005583202
ENSMUSG00000035215	1.41	0.007931713
ENSMUSG00000063439	1.41	0.000847073
ENSMUSG00000041592	1.41	0.022454918
ENSMUSG00000023571	1.41	0.00618318
ENSMUSG00000022504	1.41	5.24717E-06
ENSMUSG00000003032	1.41	0.010175797
ENSMUSG00000073802	1.41	0.00676285
ENSMUSG00000049307	1.41	0.019117613
ENSMUSG00000025225	1.41	4.78483E-07
ENSMUSG00000037563	1.41	0.000256198
ENSMUSG00000034330	1.41	3.15881E-06
ENSMUSG00000022881	1.41	0.003488079
ENSMUSG00000039221	1.41	0.001938257
ENSMUSG00000060671	1.41	3.22435E-06
ENSMUSG00000066000	1.40	0.000284643
ENSMUSG00000021725	1.40	0.006836014
ENSMUSG00000032324	1.40	2.43979E-06
ENSMUSG00000028059	1.40	5.60974E-07
ENSMUSG00000030499	1.40	0.017706397
ENSMUSG00000035239	1.40	0.019557566
ENSMUSG00000052305	1.40	0.004403197
ENSMUSG00000025902	1.40	0.000273648
ENSMUSG00000018293	1.40	2.33907E-08
ENSMUSG00000060938	1.40	0.042018926
ENSMUSG00000029231	1.40	2.90283E-07
ENSMUSG00000027160	1.40	0.000427775
ENSMUSG00000043953	1.39	0.000382049
ENSMUSG00000051790	1.39	1.05031E-05
ENSMUSG00000057789	1.39	2.5425E-07
ENSMUSG00000021765	1.39	0.038044797
ENSMUSG00000031805	1.39	1.07148E-06
ENSMUSG00000032966	1.39	8.33212E-08
ENSMUSG00000014602	1.39	0.004516489
ENSMUSG00000005465	1.39	5.12046E-05
ENSMUSG00000082433	1.38	0.044411134
ENSMUSG00000029622	1.38	8.33362E-08
ENSMUSG00000022378	1.38	2.23117E-07
ENSMUSG00000025132	1.38	4.16015E-08
ENSMUSG00000028978	1.38	3.40552E-05
ENSMUSG00000070034	1.38	3.13947E-05
ENSMUSG00000040428	1.38	0.00282308

Ensembl gene id	log2FC	p-adjusted
ENSMUSG00000031609	1.38	9.4581E-05
ENSMUSG00000024014	1.38	0.000309462
ENSMUSG00000004837	1.38	1.34496E-05
ENSMUSG00000071076	1.38	9.30049E-08
ENSMUSG00000033122	1.38	0.009435882
ENSMUSG00000019966	1.38	0.00518285
ENSMUSG00000030800	1.38	0.01484767
ENSMUSG00000029094	1.38	0.000370202
ENSMUSG00000060260	1.38	3.69841E-05
ENSMUSG00000045751	1.38	0.006143336
ENSMUSG00000027203	1.38	2.37488E-06
ENSMUSG00000036943	1.38	4.09563E-07
ENSMUSG00000059430	1.38	0.003326071
ENSMUSG00000003573	1.38	0.000580094
ENSMUSG00000033589	1.37	0.000250097
ENSMUSG00000089832	1.37	8.73949E-06
ENSMUSG00000040952	1.37	9.4581E-05
ENSMUSG00000074793	1.37	0.001214164
ENSMUSG00000029605	1.37	0.000526151
ENSMUSG00000038900	1.37	6.10995E-07
ENSMUSG00000030223	1.37	0.000922264
ENSMUSG00000032691	1.37	0.000737159
ENSMUSG00000041488	1.37	4.49024E-06
ENSMUSG00000020486	1.37	1.95285E-05
ENSMUSG00000000253	1.36	0.017802999
ENSMUSG00000089669	1.36	0.034104343
ENSMUSG00000001750	1.36	5.32821E-05
ENSMUSG00000030188	1.36	0.00107441
ENSMUSG00000071379	1.36	2.63755E-06
ENSMUSG00000020176	1.36	1.79659E-05
ENSMUSG00000002699	1.36	6.09337E-07
ENSMUSG00000021714	1.36	0.014399932
ENSMUSG00000032518	1.36	0.00012671
ENSMUSG00000054000	1.36	0.006023963
ENSMUSG00000041736	1.36	3.50272E-07
ENSMUSG00000048644	1.36	0.008910701
ENSMUSG00000025408	1.36	4.67193E-05
ENSMUSG00000074476	1.36	0.017063269
ENSMUSG00000050666	1.35	8.31787E-05
ENSMUSG00000026360	1.35	2.30832E-05
ENSMUSG00000047414	1.35	0.000786504
ENSMUSG00000085923	1.35	0.039766515
ENSMUSG00000055675	1.35	0.01852348
ENSMUSG00000052727	1.35	0.014490753
ENSMUSG00000085972	1.35	0.001926683
ENSMUSG00000054091	1.35	0.00015006
ENSMUSG00000067924	1.35	0.005573033
ENSMUSG00000024339	1.35	0.000325645
ENSMUSG00000037379	1.35	0.005749727
ENSMUSG00000032431	1.35	1.50965E-06
ENSMUSG00000060380	1.35	0.004877887
ENSMUSG00000018217	1.35	3.50429E-05

Ensembl gene id	log2FC	p-adjusted
ENSMUSG00000030342	1.35	6.40571E-07
ENSMUSG00000028796	1.35	1.92763E-07
ENSMUSG00000090019	1.35	0.000980016
ENSMUSG00000094685	1.35	0.001935514
ENSMUSG00000056116	1.34	8.54485E-05
ENSMUSG00000015013	1.34	0.000221292
ENSMUSG00000019558	1.34	3.16454E-05
ENSMUSG00000005360	1.34	0.014729299
ENSMUSG00000018537	1.34	0.007708707
ENSMUSG00000030247	1.34	0.011495258
ENSMUSG00000025268	1.34	2.30744E-06
ENSMUSG00000022673	1.34	0.043445085
ENSMUSG00000055612	1.33	0.0008768
ENSMUSG00000030659	1.33	7.24809E-05
ENSMUSG00000028551	1.33	0.000500555
ENSMUSG00000034459	1.33	0.005152422
ENSMUSG00000039126	1.33	0.000769947
ENSMUSG00000067818	1.33	2.4935E-05
ENSMUSG00000021365	1.33	2.0203E-06
ENSMUSG00000018654	1.33	3.53811E-05
ENSMUSG00000073002	1.33	0.00577311
ENSMUSG00000097081	1.33	0.012694368
ENSMUSG00000030814	1.33	0.00169629
ENSMUSG00000027318	1.33	0.006830674
ENSMUSG00000032280	1.33	1.6327E-06
ENSMUSG00000028214	1.33	0.000737252
ENSMUSG00000036908	1.32	2.11356E-07
ENSMUSG00000029725	1.32	0.011908862
ENSMUSG00000001248	1.32	1.73188E-06
ENSMUSG00000037139	1.32	0.01262338
ENSMUSG00000035314	1.32	0.002610738
ENSMUSG00000029591	1.32	3.83501E-06
ENSMUSG00000052429	1.32	1.69936E-06
ENSMUSG00000061758	1.32	0.001052139
ENSMUSG00000024608	1.32	0.001278851
ENSMUSG00000063316	1.32	0.001128441
ENSMUSG00000027452	1.32	0.002079919
ENSMUSG00000017400	1.32	0.040966705
ENSMUSG00000041889	1.32	0.013112122
ENSMUSG00000073702	1.32	0.00014983
ENSMUSG00000005973	1.31	1.16527E-06
ENSMUSG00000026177	1.31	1.12516E-06
ENSMUSG00000061718	1.31	0.021247634
ENSMUSG00000030336	1.31	0.022841092
ENSMUSG00000020778	1.31	0.001018053
ENSMUSG00000053801	1.31	3.77304E-06
ENSMUSG00000034614	1.31	0.03433234
ENSMUSG00000037816	1.31	0.001056434
ENSMUSG00000048612	1.31	6.64703E-05
ENSMUSG00000023367	1.31	0.0114236
ENSMUSG00000025203	1.31	0.0006037
ENSMUSG00000024795	1.31	0.023670035

Ensembl gene id	log2FC	p-adjusted
ENSMUSG00000025362	1.31	0.008519172
ENSMUSG00000060509	1.31	0.031715982
ENSMUSG00000041538	1.30	0.002212799
ENSMUSG00000040945	1.30	6.28714E-07
ENSMUSG00000046330	1.30	0.007153067
ENSMUSG00000044328	1.30	0.000734253
ENSMUSG00000026939	1.30	0.005327968
ENSMUSG00000097052	1.30	0.001123035
ENSMUSG00000014329	1.30	1.48165E-05
ENSMUSG00000079641	1.30	2.1584E-06
ENSMUSG00000040767	1.30	0.014902248
ENSMUSG00000031709	1.30	0.000180914
ENSMUSG00000048865	1.30	0.002994717
ENSMUSG00000027669	1.30	2.14812E-05
ENSMUSG00000037860	1.30	0.000752242
ENSMUSG00000025876	1.30	0.034170775
ENSMUSG00000037306	1.29	1.92534E-05
ENSMUSG00000018322	1.29	2.37808E-05
ENSMUSG00000063457	1.29	0.000607186
ENSMUSG00000032690	1.29	0.014007003
ENSMUSG00000006715	1.29	0.016825739
ENSMUSG00000029822	1.29	0.02754839
ENSMUSG00000035268	1.29	2.44781E-06
ENSMUSG00000021608	1.29	2.14461E-05
ENSMUSG00000041406	1.29	0.030022832
ENSMUSG00000061983	1.29	0.025159576
ENSMUSG00000019975	1.29	5.10034E-05
ENSMUSG00000046865	1.29	1.51369E-05
ENSMUSG00000059022	1.29	0.00105205
ENSMUSG00000027752	1.29	9.91294E-06
ENSMUSG00000027447	1.29	3.50161E-07
ENSMUSG00000019817	1.28	0.004629071
ENSMUSG00000026921	1.28	0.000102868
ENSMUSG00000041841	1.28	0.00019108
ENSMUSG00000028312	1.28	0.043725491
ENSMUSG00000035778	1.28	5.28226E-06
ENSMUSG00000024299	1.28	2.21301E-05
ENSMUSG00000034947	1.28	3.00364E-05
ENSMUSG00000023913	1.28	0.000278149
ENSMUSG00000039981	1.28	0.005650566
ENSMUSG00000022856	1.28	1.87427E-05
ENSMUSG00000033987	1.28	0.007401915
ENSMUSG00000026077	1.28	0.006639988
ENSMUSG00000063727	1.28	0.003767483
ENSMUSG00000006333	1.28	0.000560305
ENSMUSG00000080316	1.28	0.007236917
ENSMUSG00000005233	1.27	0.000134851
ENSMUSG00000003882	1.27	0.014686622
ENSMUSG00000067925	1.27	0.012740261
ENSMUSG00000021242	1.27	0.000316303
ENSMUSG00000074129	1.27	0.000481108
ENSMUSG00000059775	1.27	4.50628E-05

Ensembl gene id	log2FC	p-adjusted
ENSMUSG00000085795	1.27	1.29673E-05
ENSMUSG00000000552	1.27	3.85504E-06
ENSMUSG00000021115	1.27	3.79331E-05
ENSMUSG00000027953	1.27	9.15043E-06
ENSMUSG00000094910	1.27	0.002258331
ENSMUSG00000013419	1.27	0.005586353
ENSMUSG00000035697	1.27	4.17767E-06
ENSMUSG00000031750	1.27	0.000750027
ENSMUSG00000071041	1.27	0.009327412
ENSMUSG00000056220	1.27	0.000167631
ENSMUSG00000032766	1.27	0.00120591
ENSMUSG00000015476	1.27	0.014141723
ENSMUSG00000022754	1.26	0.000126679
ENSMUSG00000001166	1.26	0.010086524
ENSMUSG00000038387	1.26	0.00055997
ENSMUSG00000032477	1.26	3.51264E-05
ENSMUSG00000023191	1.26	4.5339E-05
ENSMUSG00000042677	1.26	0.000219705
ENSMUSG00000036944	1.26	0.003722743
ENSMUSG00000091509	1.26	0.003767483
ENSMUSG00000020638	1.26	0.004384822
ENSMUSG00000026456	1.26	5.34101E-05
ENSMUSG00000044092	1.26	0.007465118
ENSMUSG00000019876	1.26	0.000499329
ENSMUSG00000038668	1.26	0.006494593
ENSMUSG00000020282	1.25	0.00254201
ENSMUSG00000036381	1.25	0.000123653
ENSMUSG00000040613	1.25	0.00024417
ENSMUSG00000001056	1.25	0.000223094
ENSMUSG00000047420	1.25	0.004500087
ENSMUSG00000039457	1.25	0.003044177
ENSMUSG00000002602	1.25	2.30075E-05
ENSMUSG00000056025	1.25	2.8136E-05
ENSMUSG00000006273	1.25	1.06599E-06
ENSMUSG00000056515	1.25	9.81928E-06
ENSMUSG00000000440	1.25	0.007776415
ENSMUSG00000022488	1.25	9.48972E-05
ENSMUSG00000027613	1.25	1.00017E-06
ENSMUSG00000027386	1.25	0.027252978
ENSMUSG00000033420	1.25	0.017511246
ENSMUSG00000044341	1.25	0.012060965
ENSMUSG00000079419	1.25	0.022228045
ENSMUSG00000026627	1.25	0.00034024
ENSMUSG00000039377	1.25	4.58178E-05
ENSMUSG00000049600	1.25	0.00028399
ENSMUSG00000001034	1.25	0.000136342
ENSMUSG00000004207	1.24	5.25224E-07
ENSMUSG00000079555	1.24	0.000519582
ENSMUSG00000062997	1.24	0.00893944
ENSMUSG00000030917	1.24	0.00267001
ENSMUSG00000050379	1.24	0.000628059
ENSMUSG00000043015	1.24	0.007330138

Ensembl gene id	log2FC	p-adjusted
ENSMUSG00000085241	1.24	0.012430964
ENSMUSG00000003363	1.24	3.71864E-06
ENSMUSG00000061666	1.24	0.002045827
ENSMUSG00000062580	1.24	5.88268E-06
ENSMUSG00000060147	1.24	4.0245E-06
ENSMUSG00000022620	1.24	1.39487E-05
ENSMUSG00000029153	1.24	0.025535262
ENSMUSG00000037020	1.24	0.005140395
ENSMUSG00000078202	1.23	0.005268477
ENSMUSG00000031697	1.23	0.000130839
ENSMUSG00000004849	1.23	6.89909E-05
ENSMUSG00000021811	1.23	0.000881534
ENSMUSG00000047181	1.23	0.013147327
ENSMUSG00000061062	1.23	0.031942621
ENSMUSG00000090626	1.23	0.014195093
ENSMUSG00000053137	1.23	0.002152401
ENSMUSG00000064254	1.23	0.006914342
ENSMUSG00000035735	1.23	0.002990528
ENSMUSG00000064080	1.23	0.009982082
ENSMUSG00000026123	1.23	0.006194026
ENSMUSG00000028717	1.23	0.021426788
ENSMUSG00000055839	1.23	0.007813913
ENSMUSG00000021998	1.23	4.40113E-06
ENSMUSG00000070462	1.23	4.2361E-05
ENSMUSG00000061132	1.23	0.026468737
ENSMUSG00000059323	1.22	0.001073738
ENSMUSG00000030095	1.22	1.0534E-05
ENSMUSG00000060675	1.22	0.001351603
ENSMUSG00000025287	1.22	0.000169292
ENSMUSG00000049313	1.22	0.000430651
ENSMUSG00000054793	1.22	0.028185742
ENSMUSG00000049588	1.22	0.026275082
ENSMUSG00000039328	1.22	0.015370153
ENSMUSG00000065037	1.22	0.00510211
ENSMUSG00000054720	1.22	5.14083E-05
ENSMUSG00000053553	1.22	0.001816753
ENSMUSG00000059070	1.22	0.004408672
ENSMUSG00000047109	1.22	1.08176E-05
ENSMUSG00000036246	1.22	5.1901E-05
ENSMUSG00000019158	1.22	0.031824511
ENSMUSG00000029145	1.22	2.87607E-06
ENSMUSG00000030536	1.21	2.91739E-06
ENSMUSG00000004473	1.21	0.007160521
ENSMUSG00000039294	1.21	8.18179E-06
ENSMUSG00000059291	1.21	0.004154838
ENSMUSG00000061232	1.21	0.036767936
ENSMUSG00000005873	1.21	0.000124278
ENSMUSG00000008036	1.21	0.002010399
ENSMUSG00000046768	1.21	0.000135399
ENSMUSG00000009927	1.21	0.000773782
ENSMUSG00000067276	1.21	0.021925772
ENSMUSG00000042029	1.21	0.010076505

Ensembl gene id	log2FC	p-adjusted
ENSMUSG00000022500	1.21	1.82669E-06
ENSMUSG00000027254	1.21	0.019448893
ENSMUSG00000027834	1.21	0.025798442
ENSMUSG00000040616	1.21	7.59239E-05
ENSMUSG00000090958	1.21	1.09102E-05
ENSMUSG00000030978	1.20	0.012117111
ENSMUSG00000031465	1.20	0.00910006
ENSMUSG00000054932	1.20	0.005525711
ENSMUSG00000017631	1.20	0.000577488
ENSMUSG00000037031	1.20	5.65384E-05
ENSMUSG00000028234	1.20	3.18643E-05
ENSMUSG00000041361	1.20	0.027184039
ENSMUSG00000046688	1.20	9.81928E-06
ENSMUSG00000027562	1.20	0.005672478
ENSMUSG00000039196	1.20	0.00576884
ENSMUSG00000022186	1.20	1.18461E-05
ENSMUSG00000029538	1.20	1.06006E-05
ENSMUSG00000078348	1.20	0.002671614
ENSMUSG00000019659	1.20	0.001008357
ENSMUSG00000045917	1.20	8.36164E-06
ENSMUSG00000002477	1.20	0.00049055
ENSMUSG00000028266	1.20	5.56735E-05
ENSMUSG00000052033	1.20	0.000214806
ENSMUSG00000071451	1.20	0.000752045
ENSMUSG00000010358	1.19	0.000465572
ENSMUSG00000071662	1.19	1.0326E-05
ENSMUSG00000039747	1.19	0.019283478
ENSMUSG00000042115	1.19	0.000299516
ENSMUSG00000032796	1.19	3.56173E-05
ENSMUSG00000040699	1.19	1.32019E-05
ENSMUSG00000026786	1.19	0.000120211
ENSMUSG00000039347	1.19	0.000154133
ENSMUSG00000019261	1.19	0.000263651
ENSMUSG00000008683	1.18	1.8423E-05
ENSMUSG00000028453	1.18	0.015004972
ENSMUSG00000031633	1.18	1.60681E-05
ENSMUSG00000038141	1.18	0.02214649
ENSMUSG00000031328	1.18	3.42461E-06
ENSMUSG00000019590	1.18	0.008344345
ENSMUSG00000045176	1.18	0.004548742
ENSMUSG00000064267	1.18	0.000828434
ENSMUSG00000043895	1.18	0.000149127
ENSMUSG00000024525	1.18	0.010304573
ENSMUSG00000024621	1.18	3.09643E-06
ENSMUSG00000041926	1.18	8.25657E-06
ENSMUSG00000066877	1.18	0.001787196
ENSMUSG00000063229	1.17	0.017295932
ENSMUSG00000031785	1.17	0.001011839
ENSMUSG00000062175	1.17	0.029177734
ENSMUSG00000050106	1.17	0.000344532
ENSMUSG00000008845	1.17	0.018196304
ENSMUSG00000034271	1.17	0.002484434

Ensembl gene id	log2FC	p-adjusted
ENSMUSG00000058546	1.17	0.003805931
ENSMUSG00000048200	1.17	0.016946549
ENSMUSG00000025283	1.17	7.29614E-06
ENSMUSG00000001627	1.17	0.010143264
ENSMUSG00000027954	1.17	0.004600272
ENSMUSG00000041734	1.17	0.001233663
ENSMUSG00000039405	1.17	0.002659251
ENSMUSG00000035397	1.17	0.000736985
ENSMUSG00000083161	1.17	0.008080461
ENSMUSG00000056201	1.17	4.2446E-06
ENSMUSG00000045377	1.16	0.006745729
ENSMUSG00000049950	1.16	0.001139475
ENSMUSG00000063236	1.16	0.001075132
ENSMUSG00000032965	1.16	0.029993196
ENSMUSG00000090862	1.16	0.000400624
ENSMUSG00000074227	1.16	0.026469204
ENSMUSG00000054065	1.16	0.009292882
ENSMUSG00000052688	1.16	0.00956177
ENSMUSG00000039911	1.16	0.002685235
ENSMUSG00000074607	1.16	0.011963309
ENSMUSG00000024381	1.16	3.77774E-05
ENSMUSG00000029161	1.16	0.016115014
ENSMUSG00000031149	1.16	0.004255424
ENSMUSG00000053175	1.16	0.001742078
ENSMUSG00000040010	1.15	0.001650232
ENSMUSG00000043445	1.15	0.000209573
ENSMUSG00000098098	1.15	0.007123079
ENSMUSG00000057863	1.15	0.003803566
ENSMUSG00000079491	1.15	0.023540467
ENSMUSG00000029771	1.15	2.23527E-05
ENSMUSG00000048440	1.15	0.000182964
ENSMUSG00000005481	1.15	2.25113E-05
ENSMUSG00000020399	1.15	0.016827557
ENSMUSG00000035208	1.15	0.017665076
ENSMUSG00000019528	1.15	9.03292E-05
ENSMUSG00000031458	1.15	0.006019716
ENSMUSG00000029401	1.15	0.000265294
ENSMUSG00000001039	1.15	0.012604683
ENSMUSG00000031398	1.15	0.016847977
ENSMUSG00000030830	1.15	7.1033E-06
ENSMUSG00000005732	1.15	1.33599E-05
ENSMUSG00000021932	1.15	0.001123035
ENSMUSG00000027018	1.15	0.001751576
ENSMUSG00000039959	1.15	2.24146E-05
ENSMUSG00000021838	1.15	0.008015325
ENSMUSG00000037960	1.14	0.000506113
ENSMUSG00000035357	1.14	0.001610285
ENSMUSG00000028333	1.14	1.19576E-05
ENSMUSG00000016256	1.14	1.06272E-05
ENSMUSG00000029022	1.14	0.000313933
ENSMUSG00000026737	1.14	5.60472E-05
ENSMUSG00000028028	1.14	0.002484571

Ensembl gene id	log2FC	p-adjusted
ENSMUSG00000030432	1.14	0.005028682
ENSMUSG00000026426	1.14	5.09037E-05
ENSMUSG00000006728	1.14	1.47158E-05
ENSMUSG00000019810	1.14	7.77218E-05
ENSMUSG00000004508	1.14	0.000305885
ENSMUSG00000029616	1.14	0.012420307
ENSMUSG00000029003	1.14	8.42049E-05
ENSMUSG00000097971	1.14	0.004857382
ENSMUSG00000048677	1.14	0.000447136
ENSMUSG00000066191	1.14	0.039284949
ENSMUSG00000020717	1.14	1.68747E-05
ENSMUSG00000024725	1.14	1.865E-05
ENSMUSG00000079435	1.13	0.000997238
ENSMUSG00000023927	1.13	0.011347376
ENSMUSG00000004359	1.13	0.001892189
ENSMUSG00000034509	1.13	0.000592145
ENSMUSG00000020844	1.13	0.000526107
ENSMUSG00000058173	1.13	0.000679176
ENSMUSG00000046207	1.13	0.007810931
ENSMUSG00000025790	1.13	0.00014983
ENSMUSG00000000567	1.13	0.003352053
ENSMUSG00000033970	1.13	0.000992632
ENSMUSG00000005413	1.13	1.58145E-05
ENSMUSG00000024691	1.13	0.001040877
ENSMUSG00000031226	1.13	6.51833E-05
ENSMUSG00000032698	1.13	0.000145667
ENSMUSG00000026770	1.13	0.036463151
ENSMUSG00000022360	1.13	0.013871449
ENSMUSG00000026134	1.13	0.001376886
ENSMUSG00000025743	1.13	6.13388E-06
ENSMUSG00000021556	1.13	0.014054648
ENSMUSG00000028641	1.12	3.77697E-05
ENSMUSG00000016087	1.12	0.000889322
ENSMUSG00000057841	1.12	1.30767E-05
ENSMUSG00000073490	1.12	0.002304086
ENSMUSG00000015468	1.12	0.000806428
ENSMUSG00000026222	1.12	0.002026111
ENSMUSG00000014075	1.12	0.007409459
ENSMUSG00000024087	1.12	0.009430347
ENSMUSG00000027276	1.12	0.004764843
ENSMUSG00000025134	1.12	9.24822E-05
ENSMUSG00000024944	1.12	0.001214492
ENSMUSG00000021306	1.12	0.005294042
ENSMUSG00000006442	1.12	4.99335E-05
ENSMUSG00000078183	1.12	0.014580963
ENSMUSG00000007867	1.12	0.020604992
ENSMUSG00000039217	1.12	0.000175861
ENSMUSG00000015474	1.12	3.90924E-05
ENSMUSG00000059714	1.12	8.26482E-05
ENSMUSG00000037572	1.11	0.002628941
ENSMUSG00000097195	1.11	0.000907672
ENSMUSG00000034932	1.11	0.013147327

Ensembl gene id	log2FC	p-adjusted
ENSMUSG00000000295	1.11	0.010132955
ENSMUSG00000062382	1.11	0.03335403
ENSMUSG00000032562	1.11	1.38766E-05
ENSMUSG00000035448	1.11	0.007326021
ENSMUSG00000036655	1.11	2.77043E-05
ENSMUSG00000000078	1.11	0.000197255
ENSMUSG00000064210	1.11	3.51354E-05
ENSMUSG00000093674	1.11	0.002596091
ENSMUSG00000038467	1.10	1.67809E-05
ENSMUSG00000091337	1.10	5.12046E-05
ENSMUSG00000029810	1.10	0.001899508
ENSMUSG00000020009	1.10	0.000519635
ENSMUSG00000018378	1.10	0.002447054
ENSMUSG00000031400	1.10	0.0001735
ENSMUSG00000075284	1.10	7.65076E-05
ENSMUSG00000032353	1.10	0.017730974
ENSMUSG00000079197	1.10	0.021980102
ENSMUSG00000023805	1.10	0.036695999
ENSMUSG00000028896	1.10	0.000160339
ENSMUSG00000074577	1.10	0.00781912
ENSMUSG00000022575	1.10	2.303E-05
ENSMUSG00000015575	1.10	4.16157E-05
ENSMUSG00000061411	1.09	0.011895855
ENSMUSG00000007891	1.09	0.00093709
ENSMUSG00000029298	1.09	0.016049034
ENSMUSG00000033313	1.09	0.003863268
ENSMUSG00000019979	1.09	0.000296185
ENSMUSG00000033788	1.09	6.39879E-05
ENSMUSG00000073771	1.09	0.011306538
ENSMUSG00000040322	1.09	0.002323403
ENSMUSG00000024429	1.09	7.52916E-05
ENSMUSG00000031207	1.09	1.67805E-05
ENSMUSG00000033020	1.09	0.003894379
ENSMUSG00000044155	1.09	0.000448294
ENSMUSG00000020801	1.09	0.035591184
ENSMUSG00000028849	1.09	3.75665E-05
ENSMUSG00000026074	1.09	7.63838E-05
ENSMUSG00000022432	1.09	0.035616449
ENSMUSG00000062312	1.08	0.014384302
ENSMUSG00000045503	1.08	0.000292674
ENSMUSG00000058013	1.08	0.003592624
ENSMUSG00000020227	1.08	0.016360972
ENSMUSG00000031557	1.08	0.000276891
ENSMUSG00000047822	1.08	0.0039525
ENSMUSG00000042419	1.08	0.00092519
ENSMUSG00000039231	1.08	0.000573443
ENSMUSG00000069833	1.08	5.07405E-05
ENSMUSG00000022579	1.08	0.016093031
ENSMUSG00000001986	1.08	0.002626396
ENSMUSG00000026239	1.08	0.001040877
ENSMUSG00000039199	1.08	0.006496621
ENSMUSG00000024844	1.08	5.68401E-05

Ensembl gene id	log2FC	p-adjusted
ENSMUSG00000041930	1.08	0.023516116
ENSMUSG00000017715	1.07	0.000133528
ENSMUSG00000032369	1.07	0.002547318
ENSMUSG00000009292	1.07	0.000141132
ENSMUSG00000027327	1.07	0.002254175
ENSMUSG00000020441	1.07	0.003046957
ENSMUSG00000034708	1.07	1.97992E-05
ENSMUSG00000036820	1.07	0.040119584
ENSMUSG00000027999	1.07	0.000220363
ENSMUSG00000025511	1.07	4.87896E-05
ENSMUSG00000024247	1.07	4.22492E-05
ENSMUSG00000021958	1.07	0.003206548
ENSMUSG00000002980	1.07	0.000411341
ENSMUSG00000035835	1.07	0.022206072
ENSMUSG00000040061	1.07	0.002622335
ENSMUSG00000022114	1.07	0.008697067
ENSMUSG00000026020	1.07	0.024506309
ENSMUSG00000042492	1.07	0.000152838
ENSMUSG00000028943	1.07	0.01299527
ENSMUSG00000058099	1.06	0.00030542
ENSMUSG00000038005	1.06	0.00075634
ENSMUSG00000024308	1.06	0.001653071
ENSMUSG00000090553	1.06	0.000102628
ENSMUSG00000027506	1.06	0.00019771
ENSMUSG00000020440	1.06	0.004756979
ENSMUSG00000036114	1.06	0.016116603
ENSMUSG00000020492	1.06	0.007668169
ENSMUSG00000040435	1.06	0.021089657
ENSMUSG00000038524	1.06	0.021081403
ENSMUSG00000048332	1.06	0.000257117
ENSMUSG00000022043	1.06	0.000275746
ENSMUSG00000018585	1.06	0.011654608
ENSMUSG00000034127	1.06	0.018225111
ENSMUSG00000034993	1.06	0.000102963
ENSMUSG00000042190	1.06	0.004171103
ENSMUSG00000035199	1.06	0.005159569
ENSMUSG00000023348	1.06	0.00033774
ENSMUSG00000026107	1.06	0.005970312
ENSMUSG00000035273	1.06	0.000156617
ENSMUSG00000046546	1.05	0.000403753
ENSMUSG00000000958	1.05	0.000277577
ENSMUSG00000058600	1.05	0.003651347
ENSMUSG00000045665	1.05	0.000120999
ENSMUSG00000032725	1.05	0.027209534
ENSMUSG00000022894	1.05	0.003935162
ENSMUSG00000030528	1.05	0.008486067
ENSMUSG00000038718	1.05	0.011821011
ENSMUSG00000095115	1.05	0.000109623
ENSMUSG00000039384	1.05	0.000845113
ENSMUSG00000053040	1.05	0.005377641
ENSMUSG00000001930	1.05	0.029408401
ENSMUSG00000013698	1.05	6.13363E-05

Ensembl gene id	log2FC	p-adjusted
ENSMUSG00000050628	1.05	0.038794081
ENSMUSG00000068735	1.05	0.000538426
ENSMUSG00000063511	1.05	8.42696E-05
ENSMUSG00000046245	1.05	0.000753119
ENSMUSG00000091553	1.05	0.004889205
ENSMUSG00000056124	1.04	0.001750787
ENSMUSG00000003970	1.04	0.002809158
ENSMUSG00000074916	1.04	0.007518183
ENSMUSG00000029366	1.04	0.00472904
ENSMUSG00000012848	1.04	0.001246335
ENSMUSG00000024258	1.04	0.001056217
ENSMUSG00000029156	1.04	0.002997757
ENSMUSG00000040722	1.04	0.034801117
ENSMUSG00000068566	1.04	6.4827E-05
ENSMUSG00000049686	1.04	0.000189174
ENSMUSG00000074088	1.04	0.000387448
ENSMUSG00000036678	1.04	0.010978874
ENSMUSG00000028850	1.04	0.002315936
ENSMUSG00000002825	1.04	0.013856956
ENSMUSG00000072214	1.04	0.01667556
ENSMUSG00000027423	1.04	5.15722E-05
ENSMUSG00000038422	1.04	0.00013141
ENSMUSG00000052889	1.04	0.002412883
ENSMUSG00000024816	1.04	0.000220913
ENSMUSG00000041642	1.03	0.000831898
ENSMUSG00000045128	1.03	0.000104315
ENSMUSG00000031659	1.03	0.023497467
ENSMUSG00000048668	1.03	0.001243846
ENSMUSG00000045752	1.03	0.006508845
ENSMUSG00000049225	1.03	0.00424257
ENSMUSG00000040605	1.03	0.000510163
ENSMUSG00000038274	1.03	0.000994254
ENSMUSG00000038930	1.03	0.013204137
ENSMUSG00000025422	1.03	0.009974679
ENSMUSG00000030060	1.03	0.000737957
ENSMUSG00000004631	1.03	0.005011943
ENSMUSG00000003808	1.03	0.000191369
ENSMUSG00000033880	1.03	0.005327968
ENSMUSG00000016495	1.03	0.000544009
ENSMUSG00000026208	1.03	0.005497802
ENSMUSG00000042541	1.03	0.001075598
ENSMUSG00000057278	1.03	0.002944699
ENSMUSG00000041571	1.02	0.01777329
ENSMUSG00000091625	1.02	0.03193121
ENSMUSG00000014303	1.02	0.005308538
ENSMUSG00000026094	1.02	0.000237644
ENSMUSG00000035283	1.02	0.004766575
ENSMUSG00000020823	1.02	0.000178505
ENSMUSG00000062328	1.02	0.009103594
ENSMUSG00000063019	1.02	0.000888259
ENSMUSG00000003812	1.02	0.005254541
ENSMUSG00000090841	1.02	0.018302744

Ensembl gene id	log2FC	p-adjusted
ENSMUSG00000022181	1.02	0.00014991
ENSMUSG00000061046	1.02	0.008145464
ENSMUSG00000046722	1.02	9.27217E-05
ENSMUSG00000067586	1.02	0.001297888
ENSMUSG00000067367	1.02	0.000667354
ENSMUSG00000035967	1.02	0.012778689
ENSMUSG00000034685	1.02	0.02540477
ENSMUSG00000047712	1.02	0.017722387
ENSMUSG00000074457	1.02	0.015477134
ENSMUSG00000024644	1.02	0.000103785
ENSMUSG00000031843	1.02	0.005572224
ENSMUSG00000047767	1.02	0.002693522
ENSMUSG00000053332	1.02	8.86598E-05
ENSMUSG00000028152	1.01	0.040758036
ENSMUSG00000034254	1.01	0.000326921
ENSMUSG00000039697	1.01	0.016299073
ENSMUSG00000032366	1.01	6.66514E-05
ENSMUSG00000028081	1.01	0.000184138
ENSMUSG00000030409	1.01	9.13046E-05
ENSMUSG00000039646	1.01	0.001211039
ENSMUSG00000003848	1.01	0.00058128
ENSMUSG00000035547	1.01	0.004770794
ENSMUSG00000046434	1.01	0.00682317
ENSMUSG00000025083	1.01	0.005714388
ENSMUSG00000024732	1.01	0.000503898
ENSMUSG00000024213	1.01	0.000160471
ENSMUSG00000020476	1.01	0.00012795
ENSMUSG00000035031	1.01	0.016298987
ENSMUSG00000027639	1.01	0.00023532
ENSMUSG00000027935	1.01	0.001933087
ENSMUSG00000000957	1.01	0.003794846
ENSMUSG00000078350	1.01	0.015707292
ENSMUSG00000021951	1.00	0.005126776
ENSMUSG00000028063	1.00	0.000164147
ENSMUSG00000033350	1.00	0.002990528
ENSMUSG00000041375	1.00	0.000477286
ENSMUSG00000042638	1.00	0.009981237
ENSMUSG00000006456	1.00	0.000518702
ENSMUSG00000031367	1.00	0.002252562
ENSMUSG00000028532	1.00	0.025251687
ENSMUSG00000062352	1.00	0.00049055
ENSMUSG00000039158	1.00	0.001028004
ENSMUSG00000020467	1.00	0.000557344
ENSMUSG00000086290	0.99	0.002447489
ENSMUSG00000007029	0.98	0.000251665
ENSMUSG00000063268	0.97	0.010310433
ENSMUSG00000043505	0.97	0.004663989
ENSMUSG00000047215	0.96	0.000522805
ENSMUSG00000028339	0.95	0.006591331
ENSMUSG00000029446	0.95	0.00182727
ENSMUSG00000030032	0.94	0.007705036
ENSMUSG00000026478	0.93	0.000464414

Ensembl gene id	log2FC	p-adjusted
ENSMUSG00000032946	0.93	0.002154157
ENSMUSG00000044702	0.92	0.010587622
ENSMUSG00000039621	0.91	0.001111959
ENSMUSG00000055491	0.90	0.001115154
ENSMUSG00000025572	0.90	0.00279921
ENSMUSG00000034799	0.89	0.018167409
ENSMUSG00000031093	0.89	0.005240684
ENSMUSG00000030680	0.88	0.004959325
ENSMUSG00000021288	0.88	0.002377133
ENSMUSG00000051435	0.87	0.034069301
ENSMUSG00000062542	0.87	0.010289418
ENSMUSG00000007035	0.87	0.02456423
ENSMUSG00000022901	0.83	0.009305194
ENSMUSG00000014542	0.81	0.001416398
ENSMUSG00000046080	0.80	0.035205977
ENSMUSG00000023032	0.79	0.017142739
ENSMUSG00000066894	0.78	0.005335975
ENSMUSG00000035704	0.77	0.021953647
ENSMUSG00000020143	0.77	0.008350272
ENSMUSG00000030649	0.73	0.037064325
ENSMUSG00000089847	0.73	0.031527342
ENSMUSG00000028763	0.73	0.014182448
ENSMUSG00000025188	0.72	0.040148547
ENSMUSG00000081251	0.72	0.021088167
ENSMUSG00000079478	0.71	0.041062217
ENSMUSG00000024170	0.69	0.040258058
ENSMUSG00000054728	0.67	0.022381831
ENSMUSG00000010755	0.63	0.0076106
ENSMUSG00000026958	0.62	0.004503019
ENSMUSG00000034673	0.62	0.004551332
ENSMUSG00000038695	0.61	0.011318286
ENSMUSG00000020898	0.60	0.006285232
ENSMUSG00000069874	0.59	0.004188006
ENSMUSG00000037197	0.59	0.037544357
ENSMUSG00000009741	-1.00	0.000273648
ENSMUSG00000025037	-1.00	0.000597422
ENSMUSG00000042305	-1.00	0.000135447
ENSMUSG00000025200	-1.00	0.000336772
ENSMUSG00000022214	-1.00	9.44658E-05
ENSMUSG00000020072	-1.00	4.39352E-05
ENSMUSG00000042659	-1.00	0.000414164
ENSMUSG00000031012	-1.00	0.000653238
ENSMUSG00000020534	-1.00	8.16862E-05
ENSMUSG00000061755	-1.00	0.000400928
ENSMUSG00000002769	-1.01	4.18326E-05
ENSMUSG00000041912	-1.01	0.012965386
ENSMUSG00000031889	-1.01	0.001063353
ENSMUSG00000015305	-1.01	0.000167859
ENSMUSG00000004187	-1.01	0.007165733
ENSMUSG00000006262	-1.01	0.000198725
ENSMUSG00000045205	-1.01	0.000511782
ENSMUSG00000038217	-1.01	0.030860839

Ensembl gene id	log2FC	p-adjusted
ENSMUSG00000016534	-1.01	5.50155E-05
ENSMUSG00000021270	-1.01	0.033075714
ENSMUSG00000037119	-1.01	0.000119727
ENSMUSG00000014932	-1.01	0.000266432
ENSMUSG00000021733	-1.01	0.001620765
ENSMUSG00000000804	-1.01	0.000242471
ENSMUSG00000031540	-1.01	0.000319845
ENSMUSG00000059434	-1.01	3.41999E-05
ENSMUSG00000034171	-1.01	6.02581E-05
ENSMUSG00000032827	-1.01	0.005183694
ENSMUSG00000046318	-1.01	0.00538066
ENSMUSG00000029311	-1.01	5.79632E-05
ENSMUSG00000029759	-1.01	8.77156E-05
ENSMUSG00000085438	-1.02	0.000128513
ENSMUSG00000058152	-1.02	0.010289959
ENSMUSG00000026715	-1.02	2.27027E-05
ENSMUSG00000034075	-1.02	0.000124884
ENSMUSG00000044060	-1.02	0.007339196
ENSMUSG00000038250	-1.02	0.000321727
ENSMUSG00000055322	-1.02	0.00018425
ENSMUSG00000023031	-1.02	0.021256574
ENSMUSG00000021846	-1.02	0.000351613
ENSMUSG00000042942	-1.02	0.028171809
ENSMUSG00000035941	-1.02	8.17416E-05
ENSMUSG00000047492	-1.02	0.000402417
ENSMUSG00000005362	-1.02	0.000350794
ENSMUSG00000041935	-1.02	0.000282112
ENSMUSG00000025486	-1.02	9.27217E-05
ENSMUSG00000004233	-1.02	0.00165075
ENSMUSG00000035875	-1.02	6.68899E-05
ENSMUSG00000039182	-1.02	0.005711325
ENSMUSG00000009112	-1.02	0.000187717
ENSMUSG00000070939	-1.02	0.000120661
ENSMUSG00000027180	-1.02	0.000130064
ENSMUSG00000025899	-1.03	0.004255424
ENSMUSG00000025207	-1.03	6.09303E-05
ENSMUSG00000026775	-1.03	6.51003E-05
ENSMUSG00000060224	-1.03	8.92388E-05
ENSMUSG00000022629	-1.03	0.000141556
ENSMUSG00000039841	-1.03	0.017611031
ENSMUSG00000044617	-1.03	0.000415562
ENSMUSG00000031672	-1.03	4.54931E-05
ENSMUSG00000038764	-1.03	0.000130443
ENSMUSG00000021709	-1.03	0.000179953
ENSMUSG00000021033	-1.03	4.5413E-05
ENSMUSG00000040548	-1.03	0.000102777
ENSMUSG00000020623	-1.03	0.006188981
ENSMUSG00000038010	-1.03	0.032276772
ENSMUSG00000025369	-1.03	6.23991E-05
ENSMUSG00000031299	-1.03	6.68141E-05
ENSMUSG00000066441	-1.03	3.96256E-05
ENSMUSG00000045312	-1.03	0.000276439

Ensembl gene id	log2FC	p-adjusted
ENSMUSG00000021336	-1.03	0.000128755
ENSMUSG00000031229	-1.04	0.000108654
ENSMUSG00000054150	-1.04	0.006530454
ENSMUSG00000037475	-1.04	0.00016289
ENSMUSG00000068742	-1.04	0.001228159
ENSMUSG00000042790	-1.04	0.000662944
ENSMUSG00000008226	-1.04	9.3173E-05
ENSMUSG00000023861	-1.04	5.03749E-05
ENSMUSG00000034525	-1.04	0.000257983
ENSMUSG00000032216	-1.04	5.48479E-05
ENSMUSG00000033991	-1.04	0.00030947
ENSMUSG00000030762	-1.04	0.002093587
ENSMUSG00000005871	-1.04	0.00011192
ENSMUSG00000035161	-1.04	0.00060027
ENSMUSG00000025059	-1.04	5.20947E-05
ENSMUSG00000035572	-1.04	0.000100698
ENSMUSG00000068083	-1.04	0.005567325
ENSMUSG00000018425	-1.04	0.015957623
ENSMUSG00000019877	-1.05	4.99335E-05
ENSMUSG00000030747	-1.05	3.52958E-05
ENSMUSG00000078861	-1.05	0.010736363
ENSMUSG00000066440	-1.05	0.000135447
ENSMUSG00000057778	-1.05	0.0004384
ENSMUSG00000037416	-1.05	0.00019812
ENSMUSG00000026986	-1.05	0.001104934
ENSMUSG00000049624	-1.05	6.37402E-05
ENSMUSG00000071281	-1.05	0.0023558
ENSMUSG00000053347	-1.05	0.000753253
ENSMUSG00000030088	-1.05	1.89138E-05
ENSMUSG00000037533	-1.05	0.00026493
ENSMUSG00000027698	-1.05	8.88005E-05
ENSMUSG00000021993	-1.05	9.65089E-05
ENSMUSG00000026565	-1.05	0.000780244
ENSMUSG00000057069	-1.05	0.016832008
ENSMUSG00000021690	-1.05	0.00019481
ENSMUSG00000075273	-1.05	0.006774377
ENSMUSG00000057068	-1.05	0.003783752
ENSMUSG00000022664	-1.05	0.000477344
ENSMUSG00000022672	-1.05	0.000380491
ENSMUSG00000074582	-1.05	8.77156E-05
ENSMUSG00000043279	-1.05	0.000113134
ENSMUSG00000060397	-1.05	0.020054883
ENSMUSG00000003119	-1.05	0.000333338
ENSMUSG00000012640	-1.05	0.00037044
ENSMUSG00000044252	-1.06	7.46176E-05
ENSMUSG00000091474	-1.06	0.003412448
ENSMUSG00000000296	-1.06	0.005336545
ENSMUSG00000087370	-1.06	0.000146605
ENSMUSG00000071266	-1.06	0.000903537
ENSMUSG00000085995	-1.06	0.022319084
ENSMUSG00000031555	-1.06	5.14083E-05
ENSMUSG00000036792	-1.06	0.001233376

Ensembl gene id	log2FC	p-adjusted
ENSMUSG00000061778	-1.06	0.00012337
ENSMUSG00000045896	-1.06	0.002703087
ENSMUSG00000029735	-1.06	0.000247736
ENSMUSG00000055866	-1.06	0.00024417
ENSMUSG00000033487	-1.06	5.13349E-05
ENSMUSG00000004591	-1.06	0.000102425
ENSMUSG00000036368	-1.06	0.001333423
ENSMUSG00000036879	-1.06	0.000121615
ENSMUSG00000026991	-1.07	6.87828E-05
ENSMUSG00000028607	-1.07	4.32443E-05
ENSMUSG00000079108	-1.07	0.000503898
ENSMUSG00000058331	-1.07	0.0366579
ENSMUSG00000075703	-1.07	5.07271E-05
ENSMUSG00000037621	-1.07	0.003572307
ENSMUSG00000032531	-1.07	0.001742078
ENSMUSG00000053641	-1.07	9.13046E-05
ENSMUSG00000026259	-1.07	0.000284139
ENSMUSG00000056698	-1.07	0.000251642
ENSMUSG00000031232	-1.07	2.06103E-05
ENSMUSG00000059027	-1.07	0.001750551
ENSMUSG00000006529	-1.07	0.000238058
ENSMUSG00000029575	-1.07	0.001857336
ENSMUSG00000026781	-1.07	4.27959E-05
ENSMUSG00000040943	-1.07	0.001038873
ENSMUSG00000025558	-1.07	0.000195354
ENSMUSG00000024695	-1.07	6.92514E-05
ENSMUSG00000036585	-1.07	0.025612212
ENSMUSG00000031561	-1.07	0.01174926
ENSMUSG00000024283	-1.07	0.00011192
ENSMUSG00000028080	-1.07	8.63128E-05
ENSMUSG00000047141	-1.08	0.000278047
ENSMUSG00000024122	-1.08	8.45741E-05
ENSMUSG00000028082	-1.08	6.31719E-05
ENSMUSG00000018995	-1.08	0.000298316
ENSMUSG00000036779	-1.08	0.000591421
ENSMUSG00000005774	-1.08	0.001040877
ENSMUSG00000052914	-1.08	3.32613E-05
ENSMUSG00000027439	-1.08	5.59907E-05
ENSMUSG00000037254	-1.08	0.000550058
ENSMUSG00000032548	-1.08	0.000200367
ENSMUSG00000027189	-1.08	6.09303E-05
ENSMUSG00000020137	-1.08	0.00091418
ENSMUSG00000017831	-1.08	8.11334E-05
ENSMUSG00000051977	-1.08	0.001399245
ENSMUSG00000020590	-1.08	5.0665E-05
ENSMUSG00000085615	-1.08	0.016322451
ENSMUSG00000041852	-1.08	0.000125704
ENSMUSG00000025968	-1.08	2.7464E-05
ENSMUSG00000030172	-1.08	6.77491E-05
ENSMUSG00000025246	-1.08	7.28821E-05
ENSMUSG00000030302	-1.08	0.003929718
ENSMUSG00000048264	-1.09	0.000248853

Ensembl gene id	log2FC	p-adjusted
ENSMUSG00000062980	-1.09	4.6705E-05
ENSMUSG00000034957	-1.09	1.0997E-05
ENSMUSG00000021470	-1.09	0.04403846
ENSMUSG00000001942	-1.09	9.43847E-05
ENSMUSG00000020189	-1.09	4.20142E-05
ENSMUSG00000016128	-1.09	7.86575E-05
ENSMUSG00000054469	-1.09	4.59539E-05
ENSMUSG00000042744	-1.09	0.000426767
ENSMUSG00000028085	-1.09	5.13349E-05
ENSMUSG00000028991	-1.09	2.28845E-05
ENSMUSG00000054226	-1.09	1.40736E-05
ENSMUSG00000039599	-1.09	0.000169452
ENSMUSG00000024975	-1.09	1.52948E-05
ENSMUSG00000034557	-1.09	0.000197887
ENSMUSG00000021704	-1.09	0.000563809
ENSMUSG00000063558	-1.09	7.40201E-05
ENSMUSG00000040022	-1.09	0.001802114
ENSMUSG00000014498	-1.09	0.000224672
ENSMUSG00000024304	-1.09	3.9089E-05
ENSMUSG00000000876	-1.09	2.08759E-05
ENSMUSG00000066880	-1.09	0.004282587
ENSMUSG00000029090	-1.09	6.87816E-05
ENSMUSG00000032735	-1.09	9.35679E-05
ENSMUSG00000039653	-1.10	1.40599E-05
ENSMUSG00000074994	-1.10	0.000177932
ENSMUSG00000021000	-1.10	1.86394E-05
ENSMUSG00000033014	-1.10	0.000159217
ENSMUSG00000023055	-1.10	0.039020541
ENSMUSG00000022353	-1.10	1.72141E-05
ENSMUSG00000030671	-1.10	2.415E-05
ENSMUSG00000040209	-1.10	0.000159217
ENSMUSG00000039519	-1.10	0.000121915
ENSMUSG00000029563	-1.10	0.036783745
ENSMUSG00000032261	-1.10	7.59935E-05
ENSMUSG00000074071	-1.10	0.000144335
ENSMUSG00000029455	-1.10	7.6584E-05
ENSMUSG00000030660	-1.10	5.7545E-05
ENSMUSG00000040929	-1.10	0.005939341
ENSMUSG00000027340	-1.11	0.000240663
ENSMUSG00000030213	-1.11	0.000139875
ENSMUSG00000045973	-1.11	0.000732297
ENSMUSG00000039782	-1.11	8.56063E-05
ENSMUSG00000092260	-1.11	0.008096576
ENSMUSG00000072762	-1.11	0.005293786
ENSMUSG00000061455	-1.11	3.81907E-05
ENSMUSG00000036155	-1.11	0.00545723
ENSMUSG00000053460	-1.11	1.10249E-05
ENSMUSG00000036452	-1.11	6.87816E-05
ENSMUSG00000040225	-1.11	4.64449E-05
ENSMUSG00000027243	-1.11	0.000176758
ENSMUSG00000066319	-1.11	8.99648E-05
ENSMUSG00000020364	-1.11	0.019134333

Ensembl gene id	log2FC	p-adjusted
ENSMUSG00000072680	-1.11	0.002041458
ENSMUSG00000041915	-1.11	3.84723E-05
ENSMUSG00000021245	-1.11	0.000164746
ENSMUSG00000025396	-1.11	0.035818441
ENSMUSG00000029269	-1.11	0.000155196
ENSMUSG00000026614	-1.12	0.006570763
ENSMUSG00000037674	-1.12	0.000189993
ENSMUSG00000016382	-1.12	2.0438E-05
ENSMUSG00000068959	-1.12	0.035360847
ENSMUSG00000034245	-1.12	1.10249E-05
ENSMUSG00000035473	-1.12	1.43222E-05
ENSMUSG00000046985	-1.12	1.6221E-05
ENSMUSG00000045975	-1.12	1.85013E-05
ENSMUSG00000039652	-1.12	0.00031817
ENSMUSG00000011831	-1.12	1.54361E-05
ENSMUSG00000022309	-1.12	0.044925468
ENSMUSG00000022136	-1.12	0.002579344
ENSMUSG00000022292	-1.13	0.004449408
ENSMUSG00000048186	-1.13	0.000477335
ENSMUSG00000039220	-1.13	0.004492415
ENSMUSG00000029238	-1.13	0.004604243
ENSMUSG00000005907	-1.13	3.65655E-05
ENSMUSG00000021048	-1.13	7.45526E-06
ENSMUSG00000035173	-1.13	7.41283E-05
ENSMUSG00000067369	-1.13	4.66339E-05
ENSMUSG00000050240	-1.13	0.016972956
ENSMUSG00000074377	-1.13	0.012193631
ENSMUSG00000041782	-1.13	0.003935162
ENSMUSG00000018363	-1.13	1.66888E-05
ENSMUSG00000040370	-1.13	1.68179E-05
ENSMUSG00000018042	-1.13	3.64065E-06
ENSMUSG00000046352	-1.13	8.1851E-06
ENSMUSG00000008976	-1.13	1.85451E-05
ENSMUSG00000020715	-1.13	0.004242388
ENSMUSG00000032018	-1.14	2.93715E-06
ENSMUSG00000028838	-1.14	7.16494E-05
ENSMUSG00000031517	-1.14	0.013448818
ENSMUSG00000018387	-1.14	2.83424E-05
ENSMUSG00000024276	-1.14	5.54955E-05
ENSMUSG00000036026	-1.14	6.23991E-05
ENSMUSG00000035898	-1.14	0.000325837
ENSMUSG00000021193	-1.14	1.94312E-05
ENSMUSG00000025969	-1.14	2.19975E-05
ENSMUSG00000031986	-1.14	0.042292125
ENSMUSG00000010064	-1.14	4.03139E-06
ENSMUSG00000020546	-1.14	0.001596591
ENSMUSG00000019907	-1.14	0.000211835
ENSMUSG00000054843	-1.14	1.66937E-05
ENSMUSG00000015882	-1.14	0.00018265
ENSMUSG00000029049	-1.14	0.025274604
ENSMUSG00000033634	-1.15	6.02537E-06
ENSMUSG00000041303	-1.15	0.000444941

Ensembl gene id	log2FC	p-adjusted
ENSMUSG00000010601	-1.15	0.001044255
ENSMUSG00000026827	-1.15	0.000522324
ENSMUSG00000069206	-1.15	0.000331296
ENSMUSG00000001435	-1.15	0.000617099
ENSMUSG00000037486	-1.15	4.34933E-05
ENSMUSG00000033499	-1.15	0.000292443
ENSMUSG00000056999	-1.15	5.89472E-06
ENSMUSG00000020859	-1.15	1.21744E-05
ENSMUSG00000026638	-1.15	8.87111E-06
ENSMUSG00000027956	-1.15	0.00096481
ENSMUSG00000021982	-1.15	1.61686E-05
ENSMUSG00000028228	-1.15	2.46899E-05
ENSMUSG00000068686	-1.15	0.018255463
ENSMUSG00000027288	-1.15	1.23971E-05
ENSMUSG00000028327	-1.15	6.67616E-06
ENSMUSG00000023912	-1.15	0.029111862
ENSMUSG00000043019	-1.15	1.08176E-05
ENSMUSG00000022325	-1.15	0.001286712
ENSMUSG00000095990	-1.15	0.031820172
ENSMUSG00000029669	-1.15	6.07397E-06
ENSMUSG00000040134	-1.16	1.91202E-06
ENSMUSG00000031596	-1.16	0.022779148
ENSMUSG00000027589	-1.16	1.3846E-05
ENSMUSG00000023206	-1.16	0.001178715
ENSMUSG00000020986	-1.16	8.84557E-06
ENSMUSG00000025326	-1.16	2.00056E-05
ENSMUSG00000005803	-1.16	5.69752E-06
ENSMUSG00000050312	-1.16	0.000138345
ENSMUSG00000038023	-1.16	6.66514E-05
ENSMUSG00000023243	-1.16	8.50772E-05
ENSMUSG00000040524	-1.16	0.000613587
ENSMUSG00000075324	-1.16	0.000295578
ENSMUSG00000017453	-1.16	1.46079E-06
ENSMUSG00000050270	-1.16	5.10797E-05
ENSMUSG00000015112	-1.16	3.63386E-06
ENSMUSG00000020300	-1.16	9.86707E-06
ENSMUSG00000024354	-1.16	1.01846E-05
ENSMUSG00000003226	-1.16	7.53929E-06
ENSMUSG00000067928	-1.16	0.000641901
ENSMUSG00000026203	-1.17	2.40657E-05
ENSMUSG00000027249	-1.17	1.46611E-05
ENSMUSG00000062960	-1.17	5.73266E-06
ENSMUSG00000097119	-1.17	0.000201963
ENSMUSG00000047603	-1.17	0.014235889
ENSMUSG00000041797	-1.17	0.000279532
ENSMUSG00000020427	-1.17	1.37406E-05
ENSMUSG00000029369	-1.17	1.35351E-06
ENSMUSG00000052539	-1.17	3.29054E-05
ENSMUSG00000030972	-1.17	1.52264E-05
ENSMUSG00000021474	-1.17	2.47213E-05
ENSMUSG00000001542	-1.17	1.67809E-05
ENSMUSG00000019996	-1.17	2.6188E-05

Ensembl gene id	log2FC	p-adjusted
ENSMUSG00000034522	-1.17	0.003517328
ENSMUSG00000016942	-1.17	9.65636E-07
ENSMUSG00000025862	-1.17	9.27881E-06
ENSMUSG00000030451	-1.17	0.000833396
ENSMUSG00000046785	-1.18	5.62359E-05
ENSMUSG00000030498	-1.18	0.011491887
ENSMUSG00000090112	-1.18	4.29012E-05
ENSMUSG00000050541	-1.18	0.00014114
ENSMUSG00000063362	-1.18	1.04152E-05
ENSMUSG00000062646	-1.18	1.29679E-05
ENSMUSG00000024588	-1.18	1.85767E-06
ENSMUSG00000020170	-1.18	7.39791E-05
ENSMUSG00000031549	-1.18	0.001443976
ENSMUSG00000030934	-1.18	0.017158376
ENSMUSG00000039218	-1.18	2.10032E-05
ENSMUSG00000037580	-1.18	1.05779E-05
ENSMUSG00000032536	-1.18	9.77872E-06
ENSMUSG00000052656	-1.18	5.73671E-06
ENSMUSG00000028414	-1.18	5.14104E-05
ENSMUSG00000047193	-1.18	0.0014142
ENSMUSG00000040359	-1.18	0.001238853
ENSMUSG00000025355	-1.18	9.30035E-06
ENSMUSG00000036492	-1.18	0.001888893
ENSMUSG00000029787	-1.18	3.2694E-05
ENSMUSG00000039234	-1.18	8.92388E-05
ENSMUSG00000054648	-1.18	0.000646063
ENSMUSG00000036391	-1.18	4.04739E-06
ENSMUSG00000095432	-1.18	0.000632334
ENSMUSG00000049672	-1.18	0.000377765
ENSMUSG00000031095	-1.19	1.54858E-05
ENSMUSG00000003847	-1.19	1.17729E-05
ENSMUSG00000029833	-1.19	4.83994E-06
ENSMUSG00000032527	-1.19	8.2653E-07
ENSMUSG00000041328	-1.19	2.03606E-05
ENSMUSG00000037316	-1.19	2.42253E-05
ENSMUSG00000040848	-1.19	3.42283E-06
ENSMUSG00000000708	-1.19	3.93364E-06
ENSMUSG00000074219	-1.19	0.004136119
ENSMUSG00000038745	-1.19	2.00837E-06
ENSMUSG00000031842	-1.19	0.000986835
ENSMUSG00000026640	-1.19	8.4624E-06
ENSMUSG00000049164	-1.19	5.14083E-05
ENSMUSG00000051339	-1.19	2.71003E-06
ENSMUSG00000018401	-1.20	3.47725E-06
ENSMUSG00000036503	-1.20	2.28719E-06
ENSMUSG00000021577	-1.20	1.25446E-06
ENSMUSG00000052062	-1.20	0.003234975
ENSMUSG00000042320	-1.20	0.022357201
ENSMUSG00000021745	-1.20	1.12254E-05
ENSMUSG00000025531	-1.20	3.62098E-05
ENSMUSG00000041258	-1.20	1.56049E-05
ENSMUSG00000024781	-1.20	1.98536E-06

Ensembl gene id	log2FC	p-adjusted
ENSMUSG00000038591	-1.20	2.89505E-06
ENSMUSG00000073424	-1.20	2.91091E-05
ENSMUSG00000049470	-1.20	6.46078E-06
ENSMUSG00000069208	-1.20	0.000248723
ENSMUSG00000028572	-1.20	1.88589E-06
ENSMUSG00000067889	-1.20	0.005489252
ENSMUSG00000028402	-1.20	1.22146E-05
ENSMUSG00000039988	-1.20	3.13947E-05
ENSMUSG00000035954	-1.20	1.83633E-05
ENSMUSG00000041849	-1.20	2.61193E-05
ENSMUSG00000087385	-1.20	0.000843248
ENSMUSG00000033943	-1.21	6.6978E-06
ENSMUSG00000022066	-1.21	0.003511714
ENSMUSG00000045954	-1.21	1.45633E-05
ENSMUSG00000038774	-1.21	9.80012E-06
ENSMUSG00000032702	-1.21	3.98788E-06
ENSMUSG00000043991	-1.21	3.31603E-06
ENSMUSG00000056429	-1.21	1.96292E-06
ENSMUSG00000039976	-1.21	7.86552E-06
ENSMUSG00000040520	-1.21	1.84221E-05
ENSMUSG00000043252	-1.21	3.55051E-06
ENSMUSG00000087141	-1.21	2.76416E-06
ENSMUSG00000026721	-1.21	7.35048E-06
ENSMUSG00000032006	-1.21	0.019330205
ENSMUSG00000076435	-1.21	0.010557173
ENSMUSG00000033478	-1.21	4.9689E-06
ENSMUSG00000040029	-1.21	2.63755E-06
ENSMUSG00000036985	-1.21	1.75418E-06
ENSMUSG00000038582	-1.21	0.000102277
ENSMUSG00000026621	-1.22	1.48376E-06
ENSMUSG00000025702	-1.22	2.25406E-06
ENSMUSG00000035847	-1.22	7.35512E-06
ENSMUSG00000091586	-1.22	0.000326921
ENSMUSG00000010175	-1.22	1.91629E-06
ENSMUSG00000078234	-1.22	2.57407E-06
ENSMUSG00000026788	-1.22	1.67809E-05
ENSMUSG00000032420	-1.22	0.000217762
ENSMUSG00000082791	-1.22	0.01755651
ENSMUSG00000032607	-1.22	7.2609E-06
ENSMUSG00000060733	-1.22	4.17209E-06
ENSMUSG00000074093	-1.22	1.3179E-05
ENSMUSG00000033720	-1.22	9.90988E-06
ENSMUSG00000024580	-1.22	9.93544E-06
ENSMUSG00000039831	-1.22	1.64243E-06
ENSMUSG00000034973	-1.22	5.59796E-05
ENSMUSG00000013663	-1.22	2.66432E-06
ENSMUSG00000071226	-1.23	0.001147633
ENSMUSG00000042476	-1.23	7.43033E-07
ENSMUSG00000035441	-1.23	8.88431E-06
ENSMUSG00000057400	-1.23	1.62E-07
ENSMUSG00000030494	-1.23	1.21765E-05
ENSMUSG00000033382	-1.23	4.27046E-06

Ensembl gene id	log2FC	p-adjusted
ENSMUSG00000033533	-1.23	5.14025E-07
ENSMUSG00000032252	-1.23	4.48861E-06
ENSMUSG00000066361	-1.23	1.10734E-05
ENSMUSG00000062908	-1.23	5.21573E-07
ENSMUSG00000069727	-1.23	0.012757842
ENSMUSG00000038371	-1.23	6.94246E-06
ENSMUSG00000000056	-1.23	2.02444E-06
ENSMUSG00000021699	-1.23	0.006023963
ENSMUSG00000019873	-1.24	9.31066E-07
ENSMUSG00000059890	-1.24	1.69043E-06
ENSMUSG00000031924	-1.24	6.60955E-07
ENSMUSG00000028011	-1.24	1.32357E-06
ENSMUSG00000063428	-1.24	5.36067E-06
ENSMUSG00000046959	-1.24	7.43033E-07
ENSMUSG00000070565	-1.24	5.14083E-05
ENSMUSG00000064341	-1.24	3.54625E-07
ENSMUSG00000027761	-1.24	6.16968E-07
ENSMUSG00000039063	-1.24	7.83821E-07
ENSMUSG00000043789	-1.24	5.22023E-06
ENSMUSG00000062785	-1.24	0.000124843
ENSMUSG00000054619	-1.24	1.33514E-06
ENSMUSG00000073910	-1.24	3.39477E-06
ENSMUSG00000025176	-1.24	5.82307E-07
ENSMUSG00000032528	-1.25	1.23121E-06
ENSMUSG00000019944	-1.25	0.000582402
ENSMUSG00000037876	-1.25	3.58876E-06
ENSMUSG00000032079	-1.25	5.56735E-05
ENSMUSG00000056091	-1.25	1.10554E-06
ENSMUSG00000054052	-1.25	0.001050655
ENSMUSG00000038914	-1.25	2.95032E-06
ENSMUSG00000045095	-1.25	4.13384E-06
ENSMUSG00000041750	-1.25	0.000114481
ENSMUSG00000036782	-1.25	7.43597E-05
ENSMUSG00000032500	-1.25	0.00071789
ENSMUSG00000031628	-1.25	3.9077E-05
ENSMUSG00000038664	-1.25	3.19429E-06
ENSMUSG00000060807	-1.25	0.003088865
ENSMUSG00000036339	-1.25	1.73433E-05
ENSMUSG00000027365	-1.25	3.21913E-06
ENSMUSG00000041757	-1.25	1.8817E-06
ENSMUSG00000020640	-1.25	6.85314E-06
ENSMUSG00000086010	-1.26	9.12023E-05
ENSMUSG00000069805	-1.26	1.69873E-07
ENSMUSG00000019951	-1.26	1.74582E-06
ENSMUSG00000039480	-1.26	1.89138E-05
ENSMUSG00000053411	-1.26	0.002914694
ENSMUSG00000021712	-1.26	2.09335E-05
ENSMUSG00000040651	-1.26	2.0832E-06
ENSMUSG00000021775	-1.26	1.58809E-06
ENSMUSG00000040596	-1.26	0.000126454
ENSMUSG00000003809	-1.26	2.4863E-07
ENSMUSG00000001998	-1.26	0.00031822

Ensembl gene id	log2FC	p-adjusted
ENSMUSG00000037053	-1.26	1.14916E-06
ENSMUSG00000078435	-1.26	0.026509283
ENSMUSG00000028199	-1.26	6.23298E-07
ENSMUSG00000036019	-1.27	0.000252297
ENSMUSG00000020988	-1.27	3.73446E-06
ENSMUSG00000027263	-1.27	9.13046E-05
ENSMUSG00000034903	-1.27	0.001639822
ENSMUSG00000028514	-1.27	6.74332E-07
ENSMUSG00000032679	-1.27	0.010147386
ENSMUSG00000016028	-1.27	0.000124307
ENSMUSG00000026492	-1.27	9.13134E-05
ENSMUSG00000029776	-1.27	4.74563E-07
ENSMUSG00000048720	-1.27	6.84422E-05
ENSMUSG00000032393	-1.27	1.44172E-06
ENSMUSG00000024987	-1.28	0.006271357
ENSMUSG00000029273	-1.28	1.29069E-06
ENSMUSG00000070044	-1.28	3.41604E-06
ENSMUSG00000030161	-1.28	5.24032E-07
ENSMUSG00000031138	-1.28	1.91492E-07
ENSMUSG00000050199	-1.28	2.63987E-06
ENSMUSG00000025014	-1.28	0.018130825
ENSMUSG00000041058	-1.28	9.31525E-07
ENSMUSG00000046982	-1.28	1.91612E-05
ENSMUSG00000034574	-1.28	7.78485E-07
ENSMUSG00000041891	-1.28	2.55544E-07
ENSMUSG00000048047	-1.28	0.000178022
ENSMUSG00000041540	-1.28	0.000134809
ENSMUSG00000042647	-1.29	9.77184E-05
ENSMUSG00000057637	-1.29	2.7257E-06
ENSMUSG00000028906	-1.29	8.79205E-07
ENSMUSG00000025813	-1.29	1.15864E-06
ENSMUSG00000024431	-1.29	6.33357E-07
ENSMUSG00000063730	-1.29	0.029736938
ENSMUSG00000056492	-1.29	1.14262E-06
ENSMUSG00000073565	-1.29	2.00711E-06
ENSMUSG00000060961	-1.29	0.000191895
ENSMUSG00000014496	-1.29	3.22719E-06
ENSMUSG00000006373	-1.29	1.72693E-07
ENSMUSG00000052928	-1.29	1.03265E-06
ENSMUSG00000023070	-1.29	9.55524E-08
ENSMUSG00000027332	-1.29	2.61539E-07
ENSMUSG00000024664	-1.29	6.77491E-05
ENSMUSG00000022514	-1.29	2.27781E-07
ENSMUSG00000046798	-1.29	3.0734E-07
ENSMUSG00000045005	-1.29	4.81998E-05
ENSMUSG00000045294	-1.30	0.004399674
ENSMUSG00000028654	-1.30	0.009623626
ENSMUSG00000026024	-1.30	7.3349E-07
ENSMUSG00000005951	-1.30	4.10874E-07
ENSMUSG00000027597	-1.30	1.94345E-07
ENSMUSG00000021311	-1.30	0.000342426
ENSMUSG00000027710	-1.30	7.19378E-07

Ensembl gene id	log2FC	p-adjusted
ENSMUSG00000048271	-1.30	1.34992E-06
ENSMUSG00000046949	-1.30	7.13116E-07
ENSMUSG00000036093	-1.30	0.003949169
ENSMUSG00000022679	-1.30	5.84906E-06
ENSMUSG00000049091	-1.30	2.99525E-07
ENSMUSG00000003949	-1.30	9.2434E-07
ENSMUSG00000000753	-1.30	1.00481E-05
ENSMUSG00000021366	-1.30	1.00858E-05
ENSMUSG00000030834	-1.30	0.000112681
ENSMUSG00000030315	-1.30	2.62599E-06
ENSMUSG00000036499	-1.31	1.07518E-06
ENSMUSG00000032741	-1.31	2.96092E-07
ENSMUSG00000047153	-1.31	0.005667937
ENSMUSG00000034308	-1.31	1.12642E-07
ENSMUSG00000036550	-1.31	6.1745E-07
ENSMUSG00000006423	-1.31	3.9811E-06
ENSMUSG00000045467	-1.31	0.01189039
ENSMUSG00000046404	-1.31	0.002416542
ENSMUSG00000097554	-1.31	0.000302343
ENSMUSG00000061544	-1.32	0.00010548
ENSMUSG00000041134	-1.32	0.000111899
ENSMUSG00000069922	-1.32	2.41433E-08
ENSMUSG00000048960	-1.32	1.4021E-05
ENSMUSG00000024921	-1.32	4.18936E-07
ENSMUSG00000032086	-1.32	1.13861E-06
ENSMUSG00000069825	-1.32	0.000777056
ENSMUSG00000030109	-1.32	1.56528E-07
ENSMUSG00000031731	-1.32	1.43909E-06
ENSMUSG00000078515	-1.32	1.98622E-07
ENSMUSG00000061288	-1.32	4.32252E-07
ENSMUSG00000063550	-1.32	0.000525938
ENSMUSG00000030655	-1.32	8.03078E-07
ENSMUSG00000041426	-1.32	6.40775E-07
ENSMUSG00000090290	-1.32	0.007810931
ENSMUSG00000031574	-1.32	0.014529531
ENSMUSG00000042595	-1.33	1.54593E-05
ENSMUSG00000039840	-1.33	1.34043E-06
ENSMUSG00000035992	-1.33	8.54944E-07
ENSMUSG00000078799	-1.33	0.018117683
ENSMUSG00000009376	-1.33	5.20268E-07
ENSMUSG00000097695	-1.33	2.47853E-05
ENSMUSG00000037415	-1.33	3.48346E-07
ENSMUSG00000048218	-1.33	2.89583E-06
ENSMUSG00000060002	-1.33	2.58641E-07
ENSMUSG00000002588	-1.34	7.60055E-08
ENSMUSG00000001663	-1.34	1.50992E-07
ENSMUSG00000017418	-1.34	0.001258301
ENSMUSG00000032652	-1.34	2.29484E-07
ENSMUSG00000021069	-1.34	3.73246E-08
ENSMUSG00000054630	-1.34	8.18246E-08
ENSMUSG00000086448	-1.34	0.000143917
ENSMUSG00000024507	-1.34	4.84222E-08

Ensembl gene id	log2FC	p-adjusted
ENSMUSG00000005107	-1.34	5.20268E-07
ENSMUSG00000024425	-1.34	7.2221E-08
ENSMUSG00000047454	-1.34	2.60676E-07
ENSMUSG00000031938	-1.35	1.17978E-06
ENSMUSG00000055301	-1.35	4.53478E-06
ENSMUSG00000032050	-1.35	1.35771E-07
ENSMUSG00000006567	-1.35	3.2026E-07
ENSMUSG00000021514	-1.35	2.86201E-05
ENSMUSG00000021770	-1.35	2.77597E-07
ENSMUSG00000030030	-1.35	0.026650836
ENSMUSG00000001670	-1.35	0.008145464
ENSMUSG00000056493	-1.35	0.016920437
ENSMUSG00000012609	-1.36	5.75078E-06
ENSMUSG00000037798	-1.36	4.07783E-08
ENSMUSG00000026272	-1.36	1.0534E-05
ENSMUSG00000039967	-1.36	3.67346E-07
ENSMUSG00000070495	-1.36	0.038214228
ENSMUSG00000050144	-1.36	1.06616E-07
ENSMUSG00000070704	-1.36	3.652E-08
ENSMUSG00000040127	-1.36	1.79679E-07
ENSMUSG00000021646	-1.37	6.44225E-08
ENSMUSG00000058056	-1.37	3.84822E-05
ENSMUSG00000022772	-1.37	2.3467E-05
ENSMUSG00000024697	-1.37	0.000525938
ENSMUSG00000030465	-1.37	2.76419E-07
ENSMUSG00000025314	-1.37	1.87784E-07
ENSMUSG00000095930	-1.37	0.000283522
ENSMUSG00000051674	-1.37	0.000178903
ENSMUSG00000047466	-1.37	0.000122874
ENSMUSG00000024477	-1.37	1.70246E-05
ENSMUSG00000097493	-1.37	0.044244529
ENSMUSG00000022843	-1.37	1.66338E-07
ENSMUSG00000006134	-1.37	3.31803E-05
ENSMUSG00000025190	-1.37	1.8393E-07
ENSMUSG00000026812	-1.38	5.50754E-07
ENSMUSG00000064370	-1.38	8.62095E-09
ENSMUSG00000042213	-1.38	5.26441E-05
ENSMUSG00000035864	-1.38	4.8238E-06
ENSMUSG00000034910	-1.38	0.000777056
ENSMUSG00000033883	-1.38	2.7084E-05
ENSMUSG00000033400	-1.38	7.93533E-08
ENSMUSG00000046329	-1.38	2.27125E-08
ENSMUSG00000094786	-1.38	0.000338107
ENSMUSG00000003279	-1.39	0.000193427
ENSMUSG00000075517	-1.39	5.2668E-06
ENSMUSG00000032293	-1.39	1.25029E-07
ENSMUSG00000028842	-1.39	7.60389E-06
ENSMUSG00000036904	-1.39	1.54126E-06
ENSMUSG00000062519	-1.39	0.001174684
ENSMUSG00000082588	-1.39	0.020379804
ENSMUSG00000076441	-1.39	5.24801E-08
ENSMUSG00000038128	-1.39	0.001848089

Ensembl gene id	log2FC	p-adjusted
ENSMUSG00000035757	-1.39	2.20767E-07
ENSMUSG00000030737	-1.39	3.41442E-08
ENSMUSG00000066232	-1.39	1.49708E-07
ENSMUSG00000043467	-1.39	0.000175417
ENSMUSG00000042680	-1.39	3.89598E-07
ENSMUSG00000027358	-1.39	1.2839E-06
ENSMUSG00000058230	-1.40	5.80618E-08
ENSMUSG00000035150	-1.40	6.70647E-07
ENSMUSG00000030096	-1.40	9.32832E-08
ENSMUSG00000059811	-1.40	1.03201E-07
ENSMUSG00000050440	-1.40	0.014822849
ENSMUSG00000087361	-1.40	0.001597006
ENSMUSG00000020553	-1.40	0.002708298
ENSMUSG00000028536	-1.40	4.95173E-06
ENSMUSG00000000340	-1.40	8.18246E-08
ENSMUSG00000040855	-1.40	5.14313E-08
ENSMUSG00000009614	-1.40	1.04063E-08
ENSMUSG00000030231	-1.40	1.0546E-06
ENSMUSG00000082100	-1.41	7.2216E-07
ENSMUSG00000073664	-1.41	1.19308E-07
ENSMUSG00000037541	-1.41	0.000301916
ENSMUSG00000024548	-1.41	0.001924084
ENSMUSG00000002032	-1.41	8.18246E-08
ENSMUSG00000026866	-1.41	8.85058E-08
ENSMUSG00000018415	-1.41	3.42673E-08
ENSMUSG00000093577	-1.41	0.006077221
ENSMUSG00000025175	-1.41	4.92543E-06
ENSMUSG00000041298	-1.42	2.17769E-05
ENSMUSG00000035504	-1.42	1.06389E-08
ENSMUSG00000023977	-1.42	3.41022E-08
ENSMUSG00000013878	-1.42	1.40207E-06
ENSMUSG00000042350	-1.42	6.15077E-06
ENSMUSG00000051098	-1.42	3.5485E-06
ENSMUSG00000028517	-1.42	7.6121E-09
ENSMUSG00000039087	-1.42	8.90874E-08
ENSMUSG00000022141	-1.42	1.22114E-07
ENSMUSG00000040505	-1.42	0.017716065
ENSMUSG00000065952	-1.42	0.032420106
ENSMUSG00000031075	-1.42	0.006062172
ENSMUSG00000055312	-1.42	1.72389E-05
ENSMUSG00000052155	-1.42	1.55332E-07
ENSMUSG00000002346	-1.43	0.000124278
ENSMUSG00000021786	-1.43	8.74408E-08
ENSMUSG00000095253	-1.43	0.000128339
ENSMUSG00000026692	-1.43	0.00852211
ENSMUSG00000000711	-1.43	6.45486E-08
ENSMUSG00000086389	-1.43	0.004980994
ENSMUSG00000031853	-1.43	3.84522E-06
ENSMUSG00000042271	-1.43	4.89113E-07
ENSMUSG00000005886	-1.43	3.99013E-07
ENSMUSG00000046541	-1.43	0.000316505
ENSMUSG00000022389	-1.43	0.000101428

Ensembl gene id	log2FC	p-adjusted
ENSMUSG00000030909	-1.43	4.82994E-08
ENSMUSG00000021638	-1.43	7.29329E-05
ENSMUSG00000067653	-1.43	0.006226113
ENSMUSG00000062624	-1.43	1.04513E-08
ENSMUSG00000027598	-1.43	4.13512E-08
ENSMUSG00000020258	-1.44	1.05467E-08
ENSMUSG00000021559	-1.44	1.05334E-08
ENSMUSG00000036898	-1.44	4.69852E-06
ENSMUSG00000067851	-1.44	1.84494E-08
ENSMUSG00000063894	-1.44	1.19294E-06
ENSMUSG00000030087	-1.44	5.90372E-08
ENSMUSG00000056234	-1.44	4.52768E-06
ENSMUSG00000021779	-1.44	3.80446E-08
ENSMUSG00000057181	-1.44	4.89934E-07
ENSMUSG00000050730	-1.44	3.28663E-08
ENSMUSG00000053897	-1.44	5.9043E-08
ENSMUSG00000007827	-1.45	9.04594E-05
ENSMUSG00000058022	-1.45	2.58961E-08
ENSMUSG00000030672	-1.45	0.008212941
ENSMUSG00000086628	-1.45	7.69006E-05
ENSMUSG00000029512	-1.45	0.007383831
ENSMUSG00000022235	-1.45	5.03809E-08
ENSMUSG00000052102	-1.45	3.10909E-07
ENSMUSG00000055660	-1.45	7.20265E-05
ENSMUSG00000041977	-1.45	1.00688E-07
ENSMUSG00000028383	-1.46	1.76254E-07
ENSMUSG00000083563	-1.46	6.06479E-06
ENSMUSG00000087153	-1.46	0.003283496
ENSMUSG00000040407	-1.46	4.92296E-08
ENSMUSG00000028053	-1.46	4.38002E-08
ENSMUSG00000038949	-1.46	0.000397321
ENSMUSG00000021390	-1.46	0.006833421
ENSMUSG00000087413	-1.47	0.001531978
ENSMUSG00000063108	-1.47	1.67857E-05
ENSMUSG00000022445	-1.47	2.06587E-09
ENSMUSG00000024065	-1.47	7.43882E-09
ENSMUSG00000021708	-1.47	0.017405864
ENSMUSG00000034636	-1.47	3.57631E-08
ENSMUSG00000041698	-1.47	5.68506E-10
ENSMUSG00000018501	-1.47	1.01755E-08
ENSMUSG00000036775	-1.47	9.59269E-07
ENSMUSG00000062949	-1.47	2.54833E-09
ENSMUSG00000040181	-1.47	8.31106E-09
ENSMUSG00000037458	-1.47	1.13132E-08
ENSMUSG00000038056	-1.47	2.91519E-08
ENSMUSG00000036257	-1.47	9.63005E-09
ENSMUSG00000031274	-1.48	7.80186E-05
ENSMUSG00000024759	-1.48	9.76429E-09
ENSMUSG00000026675	-1.48	4.10182E-06
ENSMUSG00000027690	-1.48	6.49203E-09
ENSMUSG00000034066	-1.48	6.32747E-08
ENSMUSG00000071793	-1.48	0.000354669

Ensembl gene id	log2FC	p-adjusted
ENSMUSG00000037542	-1.48	4.537E-09
ENSMUSG00000041559	-1.48	0.004904789
ENSMUSG00000022788	-1.48	1.09006E-07
ENSMUSG00000044566	-1.48	0.002748285
ENSMUSG00000061371	-1.48	0.000869001
ENSMUSG00000025757	-1.48	3.04024E-08
ENSMUSG00000003233	-1.48	2.82623E-06
ENSMUSG00000003746	-1.48	2.06587E-09
ENSMUSG00000026639	-1.49	0.028165264
ENSMUSG00000034480	-1.49	1.11516E-07
ENSMUSG00000026944	-1.49	2.03342E-08
ENSMUSG00000038768	-1.49	3.07952E-06
ENSMUSG00000029263	-1.49	2.61539E-07
ENSMUSG00000055296	-1.49	5.89179E-05
ENSMUSG00000035759	-1.49	2.08489E-05
ENSMUSG00000036278	-1.49	6.66514E-05
ENSMUSG00000024052	-1.49	7.04089E-05
ENSMUSG00000022304	-1.49	6.35738E-07
ENSMUSG00000025453	-1.50	2.00089E-08
ENSMUSG00000032372	-1.50	1.974E-06
ENSMUSG00000073609	-1.50	1.43664E-06
ENSMUSG00000029167	-1.50	0.005290207
ENSMUSG00000073600	-1.50	0.000376514
ENSMUSG00000025317	-1.50	2.2565E-06
ENSMUSG00000038084	-1.50	4.94237E-09
ENSMUSG00000028976	-1.50	0.006855991
ENSMUSG00000023809	-1.50	0.000219178
ENSMUSG00000035834	-1.50	2.32412E-07
ENSMUSG00000019841	-1.50	4.20533E-08
ENSMUSG00000028307	-1.51	5.70131E-10
ENSMUSG00000018846	-1.51	2.10234E-09
ENSMUSG00000029597	-1.51	0.002291248
ENSMUSG00000017978	-1.51	1.5417E-05
ENSMUSG00000064357	-1.51	0.0005893
ENSMUSG00000096188	-1.52	6.01212E-09
ENSMUSG00000032310	-1.52	9.07056E-05
ENSMUSG00000026313	-1.52	0.000282718
ENSMUSG00000033898	-1.52	3.2612E-10
ENSMUSG00000051721	-1.52	5.38112E-05
ENSMUSG00000055493	-1.52	1.76565E-06
ENSMUSG00000020865	-1.52	5.07685E-09
ENSMUSG00000079065	-1.52	5.96107E-06
ENSMUSG00000052562	-1.53	3.5774E-09
ENSMUSG00000028518	-1.53	4.01209E-09
ENSMUSG00000022265	-1.53	4.08018E-09
ENSMUSG00000046794	-1.53	1.57231E-07
ENSMUSG00000020850	-1.53	1.16606E-09
ENSMUSG00000063077	-1.53	1.17118E-09
ENSMUSG00000022253	-1.53	1.37178E-09
ENSMUSG00000035258	-1.53	3.04206E-06
ENSMUSG00000026368	-1.53	4.74438E-10
ENSMUSG00000029729	-1.53	2.4133E-09

Ensembl gene id	log2FC	p-adjusted
ENSMUSG00000066477	-1.53	1.79885E-05
ENSMUSG00000026589	-1.53	1.30436E-09
ENSMUSG00000039100	-1.54	1.18568E-09
ENSMUSG00000022533	-1.54	1.97276E-09
ENSMUSG00000020961	-1.54	4.83304E-05
ENSMUSG00000041333	-1.54	0.005711325
ENSMUSG00000030446	-1.54	0.017802999
ENSMUSG00000037686	-1.54	4.28166E-05
ENSMUSG00000041567	-1.54	0.018363782
ENSMUSG00000031327	-1.54	2.79865E-08
ENSMUSG00000022237	-1.54	5.00199E-06
ENSMUSG00000029291	-1.55	1.13297E-07
ENSMUSG00000036501	-1.55	3.40618E-08
ENSMUSG00000059495	-1.55	8.07669E-10
ENSMUSG00000019990	-1.55	0.007608246
ENSMUSG00000056476	-1.55	7.29231E-06
ENSMUSG00000059897	-1.55	0.002604216
ENSMUSG00000070811	-1.55	1.12502E-05
ENSMUSG00000029695	-1.55	1.29431E-07
ENSMUSG00000071253	-1.55	7.90181E-10
ENSMUSG00000071037	-1.56	0.005936376
ENSMUSG00000028344	-1.56	1.89911E-07
ENSMUSG00000000686	-1.56	1.10825E-07
ENSMUSG00000035811	-1.56	0.000524656
ENSMUSG00000025903	-1.56	8.2536E-10
ENSMUSG00000033458	-1.56	1.06484E-05
ENSMUSG00000054422	-1.56	0.000174839
ENSMUSG00000041261	-1.56	9.91388E-10
ENSMUSG00000027312	-1.56	5.80771E-10
ENSMUSG00000074292	-1.56	0.038792867
ENSMUSG00000058883	-1.56	0.000642564
ENSMUSG00000024782	-1.56	1.52948E-10
ENSMUSG00000046213	-1.57	0.004609754
ENSMUSG00000020335	-1.57	0.00080934
ENSMUSG00000020053	-1.57	1.04782E-10
ENSMUSG00000059423	-1.57	4.62847E-06
ENSMUSG00000030313	-1.57	1.12956E-09
ENSMUSG00000040213	-1.57	7.84919E-11
ENSMUSG00000035840	-1.57	1.33437E-08
ENSMUSG00000038641	-1.57	1.0717E-09
ENSMUSG00000067424	-1.58	0.000903198
ENSMUSG00000057103	-1.58	8.68547E-10
ENSMUSG00000022521	-1.58	1.25904E-06
ENSMUSG00000024694	-1.58	9.83241E-10
ENSMUSG00000029556	-1.58	2.30941E-08
ENSMUSG00000052605	-1.58	6.8175E-05
ENSMUSG00000032898	-1.58	0.000538388
ENSMUSG00000061906	-1.58	4.51861E-05
ENSMUSG00000001323	-1.59	3.37386E-08
ENSMUSG00000028182	-1.59	0.00846378
ENSMUSG00000089857	-1.59	0.00135911
ENSMUSG00000038172	-1.59	2.77861E-09

Ensembl gene id	log2FC	p-adjusted
ENSMUSG00000002475	-1.59	2.03048E-10
ENSMUSG00000049152	-1.59	2.05468E-11
ENSMUSG00000054477	-1.60	5.62547E-09
ENSMUSG00000074971	-1.60	0.000692478
ENSMUSG00000045691	-1.60	5.22409E-07
ENSMUSG00000075391	-1.60	0.011180225
ENSMUSG00000021338	-1.60	0.004396612
ENSMUSG00000030055	-1.60	2.84903E-07
ENSMUSG00000042978	-1.60	4.5766E-10
ENSMUSG00000053205	-1.60	0.02591243
ENSMUSG00000030168	-1.60	1.3907E-10
ENSMUSG00000030513	-1.60	9.45995E-09
ENSMUSG00000027253	-1.60	0.000487871
ENSMUSG00000095463	-1.60	7.95143E-05
ENSMUSG00000014164	-1.60	0.000442925
ENSMUSG00000042202	-1.60	0.00131381
ENSMUSG00000035247	-1.60	1.60093E-10
ENSMUSG00000085566	-1.60	2.36907E-08
ENSMUSG00000051285	-1.60	4.39001E-10
ENSMUSG00000040536	-1.60	0.000239485
ENSMUSG00000003948	-1.61	1.92967E-05
ENSMUSG00000061838	-1.61	1.23737E-10
ENSMUSG00000079057	-1.61	1.09256E-10
ENSMUSG00000004815	-1.61	4.12743E-08
ENSMUSG00000081920	-1.61	0.00026263
ENSMUSG00000073016	-1.61	0.003722817
ENSMUSG00000056383	-1.61	1.34992E-06
ENSMUSG00000071856	-1.61	2.07012E-09
ENSMUSG00000027875	-1.61	3.88854E-09
ENSMUSG00000030340	-1.61	2.46981E-08
ENSMUSG00000090394	-1.61	7.78328E-07
ENSMUSG00000010122	-1.61	1.19837E-10
ENSMUSG00000038122	-1.61	9.17693E-07
ENSMUSG00000029202	-1.62	4.31257E-09
ENSMUSG00000041992	-1.62	2.70703E-06
ENSMUSG00000032601	-1.62	6.2801E-10
ENSMUSG00000082586	-1.62	0.000135877
ENSMUSG00000063450	-1.62	6.04139E-05
ENSMUSG00000032041	-1.62	1.3595E-09
ENSMUSG00000022323	-1.62	2.41935E-10
ENSMUSG00000035868	-1.62	0.000268646
ENSMUSG00000022519	-1.62	5.88268E-06
ENSMUSG00000029178	-1.62	7.77657E-09
ENSMUSG00000046138	-1.62	5.78881E-09
ENSMUSG00000056978	-1.62	0.031826298
ENSMUSG00000045410	-1.62	0.00606279
ENSMUSG00000020275	-1.62	0.001147633
ENSMUSG00000048647	-1.62	2.0203E-06
ENSMUSG00000032743	-1.62	6.48911E-09
ENSMUSG00000028132	-1.62	2.9996E-10
ENSMUSG00000058258	-1.63	4.80825E-05
ENSMUSG00000024241	-1.63	1.01212E-09

Ensembl gene id	log2FC	p-adjusted
ENSMUSG00000073678	-1.63	6.56427E-10
ENSMUSG00000040446	-1.63	2.61758E-06
ENSMUSG00000074882	-1.63	2.83199E-11
ENSMUSG00000045284	-1.63	1.28568E-05
ENSMUSG00000052957	-1.63	2.88155E-05
ENSMUSG00000057924	-1.63	0.006153174
ENSMUSG00000005089	-1.64	7.64736E-11
ENSMUSG00000085867	-1.64	2.08246E-08
ENSMUSG00000057396	-1.64	5.5165E-06
ENSMUSG00000073988	-1.64	6.77177E-11
ENSMUSG00000034456	-1.64	6.09521E-11
ENSMUSG00000022040	-1.64	1.1392E-07
ENSMUSG00000026380	-1.64	1.41244E-06
ENSMUSG00000050556	-1.64	0.009711978
ENSMUSG00000030236	-1.64	2.84785E-11
ENSMUSG00000035164	-1.64	1.05266E-06
ENSMUSG00000070810	-1.64	8.46052E-09
ENSMUSG00000024378	-1.64	1.40097E-11
ENSMUSG00000021573	-1.64	0.000260651
ENSMUSG00000003585	-1.64	2.60529E-11
ENSMUSG00000043154	-1.64	5.53469E-09
ENSMUSG00000039496	-1.65	0.036832808
ENSMUSG00000079487	-1.65	0.000265358
ENSMUSG00000021676	-1.65	4.07155E-11
ENSMUSG00000026417	-1.65	2.61776E-12
ENSMUSG00000042743	-1.65	4.01254E-05
ENSMUSG00000027809	-1.65	1.18675E-11
ENSMUSG00000044086	-1.65	0.01411478
ENSMUSG00000075044	-1.66	0.000821028
ENSMUSG00000044033	-1.66	1.24147E-08
ENSMUSG00000050103	-1.66	3.35951E-09
ENSMUSG00000010663	-1.66	1.599E-11
ENSMUSG00000030259	-1.66	2.85288E-09
ENSMUSG00000055782	-1.66	2.25291E-11
ENSMUSG00000074925	-1.66	0.000983873
ENSMUSG00000034563	-1.66	0.003392635
ENSMUSG00000087241	-1.67	9.55835E-05
ENSMUSG00000005534	-1.67	2.17372E-11
ENSMUSG00000042834	-1.67	3.81684E-10
ENSMUSG00000037234	-1.67	4.41757E-11
ENSMUSG00000085396	-1.67	2.40445E-07
ENSMUSG00000025764	-1.67	2.68178E-10
ENSMUSG00000035078	-1.68	5.37778E-09
ENSMUSG00000019856	-1.68	0.021490744
ENSMUSG00000029173	-1.68	4.68955E-11
ENSMUSG00000038751	-1.68	1.97311E-05
ENSMUSG00000044308	-1.68	3.24897E-11
ENSMUSG00000054387	-1.68	2.81177E-08
ENSMUSG00000044393	-1.68	4.58751E-11
ENSMUSG00000021482	-1.68	5.54882E-10
ENSMUSG00000030257	-1.68	4.80681E-08
ENSMUSG00000037940	-1.68	0.008041597

Ensembl gene id	log2FC	p-adjusted
ENSMUSG00000078689	-1.68	0.040895453
ENSMUSG00000027016	-1.68	1.95321E-08
ENSMUSG00000033715	-1.68	2.58523E-11
ENSMUSG00000020220	-1.68	1.65644E-10
ENSMUSG00000039853	-1.68	1.13568E-08
ENSMUSG00000069184	-1.68	0.009691388
ENSMUSG00000031445	-1.69	5.80717E-12
ENSMUSG00000067219	-1.69	0.000198003
ENSMUSG00000044340	-1.69	4.37238E-10
ENSMUSG00000026623	-1.69	2.25143E-11
ENSMUSG00000037355	-1.69	4.78965E-09
ENSMUSG00000033295	-1.69	3.02218E-11
ENSMUSG00000039952	-1.70	4.81887E-11
ENSMUSG00000046070	-1.70	3.12553E-12
ENSMUSG00000094472	-1.70	0.002356034
ENSMUSG00000025261	-1.70	9.9313E-12
ENSMUSG00000018800	-1.70	1.63218E-08
ENSMUSG00000025255	-1.70	8.41028E-10
ENSMUSG00000038145	-1.70	1.01752E-10
ENSMUSG00000097357	-1.70	0.000597316
ENSMUSG00000053279	-1.71	0.008011892
ENSMUSG00000042473	-1.71	5.29668E-09
ENSMUSG00000084883	-1.71	3.10342E-05
ENSMUSG00000052676	-1.71	1.36544E-07
ENSMUSG00000031767	-1.71	0.003484887
ENSMUSG00000030244	-1.71	0.00231543
ENSMUSG00000025479	-1.71	9.59834E-13
ENSMUSG00000068587	-1.71	5.02837E-12
ENSMUSG00000024292	-1.71	2.07467E-12
ENSMUSG00000021767	-1.72	4.94274E-09
ENSMUSG00000040583	-1.72	5.47768E-12
ENSMUSG00000021622	-1.72	0.010680508
ENSMUSG00000032313	-1.72	0.002383033
ENSMUSG00000039202	-1.72	0.006469827
ENSMUSG00000024413	-1.72	4.67157E-07
ENSMUSG00000041660	-1.72	7.34382E-12
ENSMUSG00000048087	-1.72	1.92673E-11
ENSMUSG00000054827	-1.73	2.62679E-05
ENSMUSG00000006494	-1.73	5.50754E-07
ENSMUSG00000086782	-1.73	0.000644839
ENSMUSG00000020532	-1.73	4.12162E-09
ENSMUSG00000011382	-1.73	7E-12
ENSMUSG00000024601	-1.73	1.50023E-11
ENSMUSG00000021798	-1.73	0.029762048
ENSMUSG00000049971	-1.73	0.001867876
ENSMUSG00000031665	-1.73	2.91162E-11
ENSMUSG00000021903	-1.73	3.88992E-05
ENSMUSG00000085687	-1.73	0.002641042
ENSMUSG00000025588	-1.73	0.005683275
ENSMUSG00000050931	-1.73	2.65784E-10
ENSMUSG00000033396	-1.73	3.08642E-09
ENSMUSG00000003178	-1.73	9.37424E-07

Ensembl gene id	log2FC	p-adjusted
ENSMUSG00000057551	-1.74	2.26536E-07
ENSMUSG00000024228	-1.74	3.06171E-11
ENSMUSG00000070997	-1.74	0.0124096
ENSMUSG00000021135	-1.74	5.14067E-13
ENSMUSG00000038286	-1.75	1.05083E-12
ENSMUSG00000029195	-1.75	5.29037E-11
ENSMUSG00000042851	-1.75	0.001640083
ENSMUSG00000024471	-1.75	0.038277508
ENSMUSG00000036863	-1.75	4.43066E-07
ENSMUSG00000086054	-1.75	0.000752853
ENSMUSG00000025241	-1.75	9.6626E-12
ENSMUSG00000089828	-1.76	0.003901407
ENSMUSG00000037646	-1.76	9.44675E-12
ENSMUSG00000042251	-1.76	9.90023E-08
ENSMUSG00000037826	-1.76	0.022686051
ENSMUSG00000027784	-1.77	6.63816E-11
ENSMUSG00000033871	-1.77	6.7473E-06
ENSMUSG00000087382	-1.77	4.43808E-06
ENSMUSG00000021952	-1.77	5.91141E-07
ENSMUSG00000024887	-1.77	4.9532E-11
ENSMUSG00000085328	-1.77	6.77319E-07
ENSMUSG00000019768	-1.77	3.87747E-06
ENSMUSG00000097284	-1.78	0.000358086
ENSMUSG00000019820	-1.78	7.38665E-12
ENSMUSG00000026687	-1.78	5.4788E-13
ENSMUSG00000079164	-1.78	0.002435025
ENSMUSG00000041650	-1.78	2.54981E-12
ENSMUSG00000021620	-1.78	1.05717E-12
ENSMUSG00000084329	-1.78	3.45168E-06
ENSMUSG00000087478	-1.78	0.005750328
ENSMUSG00000026185	-1.79	5.94418E-11
ENSMUSG00000099146	-1.79	2.97742E-11
ENSMUSG00000034785	-1.79	0.000282024
ENSMUSG00000042460	-1.79	4.81955E-10
ENSMUSG00000033102	-1.79	7.92214E-06
ENSMUSG00000040147	-1.79	5.20065E-13
ENSMUSG00000039477	-1.80	1.80866E-06
ENSMUSG00000038384	-1.80	4.6688E-05
ENSMUSG00000075551	-1.80	4.09686E-09
ENSMUSG00000053219	-1.80	0.044244529
ENSMUSG00000097207	-1.80	0.005251744
ENSMUSG00000016756	-1.80	3.88148E-13
ENSMUSG00000055228	-1.80	0.002056713
ENSMUSG00000034949	-1.80	0.017249965
ENSMUSG00000049044	-1.80	3.60995E-12
ENSMUSG00000032065	-1.80	0.031527342
ENSMUSG00000035000	-1.81	3.61178E-13
ENSMUSG00000034342	-1.81	5.18237E-07
ENSMUSG00000021223	-1.81	0.000237882
ENSMUSG00000033863	-1.81	3.43356E-11
ENSMUSG00000020634	-1.81	3.89067E-11
ENSMUSG00000064351	-1.81	1.32453E-13

Ensembl gene id	log2FC	p-adjusted
ENSMUSG00000003623	-1.82	1.13072E-06
ENSMUSG00000024900	-1.82	5.20065E-13
ENSMUSG00000042496	-1.82	9.21501E-06
ENSMUSG00000027048	-1.82	7.27058E-13
ENSMUSG00000089991	-1.82	1.07932E-12
ENSMUSG00000024827	-1.82	2.14906E-12
ENSMUSG00000054008	-1.82	1.23476E-12
ENSMUSG00000046782	-1.82	0.012116824
ENSMUSG00000068284	-1.82	2.53961E-12
ENSMUSG00000083798	-1.83	0.0420101
ENSMUSG00000026489	-1.83	1.49152E-13
ENSMUSG00000075307	-1.83	0.015772005
ENSMUSG00000055480	-1.83	0.000871007
ENSMUSG00000037818	-1.84	8.44488E-12
ENSMUSG00000030701	-1.84	1.62675E-11
ENSMUSG00000037110	-1.84	6.87207E-12
ENSMUSG00000026579	-1.84	1.03889E-06
ENSMUSG00000028051	-1.84	2.61838E-08
ENSMUSG00000056019	-1.85	0.000218498
ENSMUSG00000054263	-1.85	3.09261E-13
ENSMUSG00000041828	-1.85	9.81589E-05
ENSMUSG00000025429	-1.85	2.49347E-09
ENSMUSG00000071633	-1.85	3.16737E-13
ENSMUSG00000029456	-1.85	3.60802E-12
ENSMUSG00000028780	-1.85	0.000960184
ENSMUSG00000070985	-1.85	8.02849E-12
ENSMUSG00000025197	-1.85	4.73394E-14
ENSMUSG00000020948	-1.85	1.55632E-05
ENSMUSG00000074829	-1.86	4.67157E-07
ENSMUSG00000054545	-1.86	8.59772E-11
ENSMUSG00000057228	-1.86	3.98861E-13
ENSMUSG00000016194	-1.86	8.57262E-15
ENSMUSG00000074519	-1.86	0.000103607
ENSMUSG00000009633	-1.86	0.002207086
ENSMUSG00000086868	-1.87	4.37148E-06
ENSMUSG00000015127	-1.87	8.67237E-05
ENSMUSG00000071796	-1.87	4.96207E-05
ENSMUSG00000032808	-1.87	4.50303E-05
ENSMUSG00000027359	-1.87	2.37337E-14
ENSMUSG00000056342	-1.87	9.40765E-13
ENSMUSG00000042985	-1.87	3.6864E-05
ENSMUSG00000064294	-1.87	3.53091E-14
ENSMUSG00000029004	-1.87	2.62506E-12
ENSMUSG00000040761	-1.87	1.1116E-10
ENSMUSG00000055737	-1.88	4.13672E-14
ENSMUSG00000037440	-1.88	0.003429604
ENSMUSG00000064345	-1.88	7.45735E-15
ENSMUSG00000019232	-1.88	0.003840743
ENSMUSG00000072949	-1.88	0.043725491
ENSMUSG00000029571	-1.88	5.26079E-14
ENSMUSG00000015970	-1.88	4.65359E-14
ENSMUSG00000086502	-1.89	0.025658252

Ensembl gene id	log2FC	p-adjusted
ENSMUSG00000075552	-1.89	1.46901E-09
ENSMUSG00000086450	-1.89	0.008792349
ENSMUSG00000022887	-1.90	3.16737E-13
ENSMUSG00000097908	-1.90	2.02929E-08
ENSMUSG00000026004	-1.90	3.13796E-10
ENSMUSG00000028713	-1.90	8.7509E-10
ENSMUSG00000039116	-1.90	2.2144E-05
ENSMUSG00000041132	-1.90	0.000869355
ENSMUSG00000040740	-1.91	2.14978E-10
ENSMUSG00000021611	-1.91	5.42604E-08
ENSMUSG00000026610	-1.91	1.34496E-05
ENSMUSG00000033306	-1.91	1.0525E-13
ENSMUSG00000026495	-1.91	2.59101E-05
ENSMUSG00000015890	-1.91	1.16435E-13
ENSMUSG00000028167	-1.91	0.001022833
ENSMUSG00000031433	-1.91	4.08299E-06
ENSMUSG00000028008	-1.92	2.54435E-06
ENSMUSG00000024254	-1.92	0.0153343
ENSMUSG00000048856	-1.92	7.65076E-05
ENSMUSG00000020709	-1.92	1.20292E-11
ENSMUSG00000097662	-1.92	0.000407669
ENSMUSG00000090286	-1.92	0.002941818
ENSMUSG00000031666	-1.92	5.06706E-13
ENSMUSG00000018166	-1.92	0.000426257
ENSMUSG00000030643	-1.92	1.70984E-06
ENSMUSG00000025323	-1.92	6.07272E-09
ENSMUSG00000020091	-1.93	1.11828E-13
ENSMUSG00000035686	-1.93	5.56463E-05
ENSMUSG00000030861	-1.93	1.14901E-14
ENSMUSG00000025003	-1.94	3.68852E-15
ENSMUSG00000078798	-1.94	0.030539682
ENSMUSG00000090150	-1.94	6.39602E-15
ENSMUSG00000046480	-1.94	0.011860846
ENSMUSG00000037270	-1.94	2.67621E-14
ENSMUSG00000033965	-1.94	5.38685E-07
ENSMUSG00000028957	-1.94	0.000781551
ENSMUSG00000033196	-1.95	0.012927166
ENSMUSG00000045875	-1.95	1.65721E-06
ENSMUSG00000026384	-1.95	9.02146E-08
ENSMUSG00000047496	-1.95	8.8472E-07
ENSMUSG00000090799	-1.95	0.008456417
ENSMUSG00000082519	-1.95	5.9043E-08
ENSMUSG00000030424	-1.95	0.003826402
ENSMUSG00000026017	-1.96	0.000204946
ENSMUSG00000056328	-1.96	0.014819375
ENSMUSG00000021364	-1.96	4.54022E-15
ENSMUSG00000026398	-1.96	1.85523E-13
ENSMUSG00000068130	-1.96	0.010471178
ENSMUSG00000070594	-1.96	5.04403E-16
ENSMUSG00000028127	-1.96	1.41741E-08
ENSMUSG00000061292	-1.96	8.10236E-13
ENSMUSG00000005373	-1.97	1.7561E-12

Ensembl gene id	log2FC	p-adjusted
ENSMUSG00000025153	-1.97	7.34696E-13
ENSMUSG00000049630	-1.97	0.027252978
ENSMUSG00000038174	-1.97	3.028E-13
ENSMUSG00000038121	-1.97	8.62163E-14
ENSMUSG00000079469	-1.98	1.24652E-06
ENSMUSG00000041702	-1.98	3.62089E-10
ENSMUSG00000046230	-1.98	1.95004E-12
ENSMUSG00000042102	-1.98	8.85097E-16
ENSMUSG00000074272	-1.98	3.90188E-15
ENSMUSG00000021884	-1.98	4.84162E-16
ENSMUSG00000020051	-1.99	3.69272E-15
ENSMUSG00000025991	-1.99	1.6604E-11
ENSMUSG00000090623	-1.99	1.42809E-05
ENSMUSG00000032410	-1.99	1.04183E-09
ENSMUSG00000096401	-1.99	8.00138E-05
ENSMUSG00000070461	-1.99	1.30352E-05
ENSMUSG00000094856	-1.99	0.033440202
ENSMUSG00000028542	-1.99	1.89498E-07
ENSMUSG00000029260	-1.99	3.04857E-15
ENSMUSG00000021057	-1.99	0.005876535
ENSMUSG00000010051	-1.99	6.62671E-13
ENSMUSG00000029630	-2.00	2.43221E-06
ENSMUSG00000028399	-2.00	3.04857E-15
ENSMUSG00000054031	-2.00	5.53718E-07
ENSMUSG00000055725	-2.00	3.2452E-09
ENSMUSG00000019906	-2.00	9.41327E-14
ENSMUSG00000030486	-2.00	0.001335968
ENSMUSG00000060012	-2.00	2.12203E-12
ENSMUSG00000023845	-2.01	3.03684E-12
ENSMUSG00000086507	-2.01	0.000161903
ENSMUSG00000046840	-2.01	0.001056434
ENSMUSG00000002222	-2.01	1.05845E-15
ENSMUSG00000032009	-2.01	1.62126E-10
ENSMUSG00000093385	-2.01	0.001700867
ENSMUSG00000039701	-2.01	2.4142E-08
ENSMUSG00000086141	-2.01	4.58751E-11
ENSMUSG00000031196	-2.01	1.28239E-13
ENSMUSG00000046532	-2.02	2.51138E-12
ENSMUSG00000092335	-2.02	0.004980388
ENSMUSG00000045106	-2.02	0.002464506
ENSMUSG00000018900	-2.02	9.01042E-07
ENSMUSG00000027950	-2.03	0.004924221
ENSMUSG00000030935	-2.03	3.24391E-14
ENSMUSG00000034687	-2.03	5.3465E-06
ENSMUSG00000058997	-2.04	2.98606E-05
ENSMUSG00000036110	-2.04	7.09027E-17
ENSMUSG00000025195	-2.04	7.37549E-09
ENSMUSG00000041324	-2.04	2.04843E-07
ENSMUSG00000051910	-2.04	2.54255E-12
ENSMUSG00000069892	-2.04	1.62503E-06
ENSMUSG00000044452	-2.04	3.51271E-11
ENSMUSG00000091089	-2.04	0.005165784

Ensembl gene id	log2FC	p-adjusted
ENSMUSG00000053825	-2.05	0.007549801
ENSMUSG00000030592	-2.05	0.011671455
ENSMUSG00000024665	-2.05	5.17531E-16
ENSMUSG00000090171	-2.05	0.000522324
ENSMUSG00000022041	-2.05	8.92388E-05
ENSMUSG00000020889	-2.06	9.10221E-12
ENSMUSG00000086245	-2.06	0.005291064
ENSMUSG00000050321	-2.06	0.044543443
ENSMUSG00000041372	-2.06	0.004799747
ENSMUSG00000020021	-2.06	1.38563E-15
ENSMUSG00000087368	-2.06	0.006112024
ENSMUSG00000042390	-2.06	3.66273E-08
ENSMUSG00000052520	-2.07	1.23984E-16
ENSMUSG00000023122	-2.07	8.94714E-08
ENSMUSG00000090659	-2.07	0.000129988
ENSMUSG00000064340	-2.07	0.000107166
ENSMUSG00000025017	-2.07	2.83188E-14
ENSMUSG00000040170	-2.07	1.04641E-14
ENSMUSG00000071669	-2.07	2.57263E-06
ENSMUSG00000048915	-2.08	0.000886524
ENSMUSG00000024302	-2.08	0.026360662
ENSMUSG00000032349	-2.08	6.07013E-17
ENSMUSG00000078650	-2.08	0.019091267
ENSMUSG00000028630	-2.08	3.33849E-12
ENSMUSG00000038763	-2.08	0.00931747
ENSMUSG00000086430	-2.09	0.013630471
ENSMUSG00000033174	-2.09	1.48655E-08
ENSMUSG00000042010	-2.09	1.32802E-07
ENSMUSG00000090353	-2.09	0.000526151
ENSMUSG00000057132	-2.09	0.00019123
ENSMUSG00000078486	-2.09	0.000190918
ENSMUSG00000033792	-2.09	3.13014E-08
ENSMUSG00000090145	-2.09	1.96808E-13
ENSMUSG00000037112	-2.09	1.17353E-09
ENSMUSG00000032418	-2.09	1.1473E-08
ENSMUSG00000028030	-2.09	1.01839E-09
ENSMUSG00000048732	-2.10	0.000107471
ENSMUSG00000039145	-2.10	1.01442E-16
ENSMUSG00000020672	-2.10	3.33734E-13
ENSMUSG00000092008	-2.10	2.62128E-10
ENSMUSG00000036027	-2.10	4.38722E-05
ENSMUSG00000035270	-2.10	1.65034E-05
ENSMUSG00000038522	-2.10	1.1851E-15
ENSMUSG00000038670	-2.11	0.011969062
ENSMUSG00000027071	-2.11	0.010506547
ENSMUSG00000048280	-2.11	1.48848E-05
ENSMUSG00000091764	-2.11	0.009231571
ENSMUSG00000001985	-2.12	0.02181929
ENSMUSG00000090165	-2.12	7.90171E-07
ENSMUSG00000033610	-2.12	1.12972E-17
ENSMUSG00000063047	-2.12	0.002074559
ENSMUSG00000073555	-2.13	1.08009E-16

Ensembl gene id	log2FC	p-adjusted
ENSMUSG00000028194	-2.13	7.23457E-14
ENSMUSG00000043013	-2.13	0.00337377
ENSMUSG00000024437	-2.13	0.001073135
ENSMUSG00000082534	-2.13	4.28378E-06
ENSMUSG00000024485	-2.13	0.005437463
ENSMUSG00000035133	-2.13	4.05365E-15
ENSMUSG00000056258	-2.13	0.00566046
ENSMUSG00000031173	-2.14	7.62815E-18
ENSMUSG00000075470	-2.14	5.31233E-14
ENSMUSG00000037709	-2.14	9.27673E-14
ENSMUSG00000035780	-2.14	1.15164E-17
ENSMUSG00000006784	-2.14	0.005346075
ENSMUSG00000020777	-2.15	1.24382E-07
ENSMUSG00000032064	-2.15	1.70329E-11
ENSMUSG00000061175	-2.15	1.10562E-06
ENSMUSG00000002265	-2.15	1.61024E-07
ENSMUSG00000086296	-2.15	0.000394866
ENSMUSG00000059149	-2.16	1.45892E-06
ENSMUSG00000027792	-2.16	1.84896E-18
ENSMUSG00000020609	-2.16	1.7103E-12
ENSMUSG00000040998	-2.16	0.000278149
ENSMUSG00000027346	-2.16	1.36972E-13
ENSMUSG00000039891	-2.16	0.039449115
ENSMUSG00000022401	-2.16	1.32679E-14
ENSMUSG00000096463	-2.16	0.000472205
ENSMUSG00000037795	-2.16	9.95443E-17
ENSMUSG00000065987	-2.16	0.004681459
ENSMUSG00000038024	-2.16	9.33291E-14
ENSMUSG00000066944	-2.17	3.10342E-05
ENSMUSG00000086796	-2.18	0.028574707
ENSMUSG00000031292	-2.18	4.52244E-05
ENSMUSG00000030731	-2.18	6.52002E-05
ENSMUSG00000029206	-2.18	0.005092164
ENSMUSG00000066571	-2.18	4.2182E-10
ENSMUSG00000071691	-2.18	0.032449274
ENSMUSG00000033618	-2.18	1.61255E-07
ENSMUSG00000063929	-2.18	5.33872E-06
ENSMUSG00000085400	-2.18	0.025169174
ENSMUSG00000022708	-2.19	2.27004E-13
ENSMUSG00000071551	-2.19	1.32202E-09
ENSMUSG00000035936	-2.19	2.22274E-17
ENSMUSG00000023921	-2.19	1.95458E-18
ENSMUSG00000084788	-2.19	0.034294309
ENSMUSG00000067225	-2.19	2.51261E-05
ENSMUSG00000035284	-2.20	8.6921E-17
ENSMUSG00000033855	-2.20	5.96495E-11
ENSMUSG00000024130	-2.20	1.06334E-18
ENSMUSG00000005268	-2.20	3.09556E-19
ENSMUSG00000032207	-2.21	1.07744E-19
ENSMUSG00000074283	-2.21	2.34907E-05
ENSMUSG00000021238	-2.21	1.55354E-19
ENSMUSG00000039879	-2.22	1.6121E-15

Ensembl gene id	log2FC	p-adjusted
ENSMUSG00000097811	-2.22	8.42696E-05
ENSMUSG00000049791	-2.22	1.65283E-14
ENSMUSG00000021097	-2.22	7.52557E-08
ENSMUSG00000041237	-2.22	5.36997E-20
ENSMUSG00000020017	-2.22	1.09712E-17
ENSMUSG00000038656	-2.23	1.54687E-14
ENSMUSG00000025949	-2.23	1.61595E-13
ENSMUSG00000031529	-2.23	1.96995E-13
ENSMUSG00000019726	-2.23	1.00695E-15
ENSMUSG00000041044	-2.23	2.67528E-12
ENSMUSG00000055704	-2.23	0.000448364
ENSMUSG00000074794	-2.24	1.3341E-06
ENSMUSG00000024978	-2.24	9.84976E-19
ENSMUSG00000034218	-2.24	1.77288E-12
ENSMUSG00000006587	-2.24	5.74066E-05
ENSMUSG00000072774	-2.24	0.04433207
ENSMUSG00000072999	-2.24	0.002604216
ENSMUSG00000074824	-2.25	5.21196E-06
ENSMUSG00000057037	-2.26	1.42257E-19
ENSMUSG00000060429	-2.26	1.43236E-15
ENSMUSG00000027015	-2.26	0.023497467
ENSMUSG00000073647	-2.26	0.00924906
ENSMUSG00000043753	-2.26	0.006909833
ENSMUSG00000046169	-2.26	0.009793116
ENSMUSG00000027965	-2.27	9.13046E-05
ENSMUSG00000037406	-2.27	0.016547989
ENSMUSG00000029254	-2.27	1.94312E-05
ENSMUSG00000094613	-2.27	0.000457273
ENSMUSG00000062901	-2.27	5.96608E-05
ENSMUSG00000047394	-2.27	9.7248E-06
ENSMUSG00000058523	-2.27	8.00965E-05
ENSMUSG00000019947	-2.28	2.07467E-12
ENSMUSG00000019368	-2.28	3.17439E-19
ENSMUSG00000063590	-2.28	9.13216E-12
ENSMUSG00000023737	-2.28	0.017706397
ENSMUSG00000079645	-2.28	0.033063463
ENSMUSG00000021259	-2.29	0.007834075
ENSMUSG00000024558	-2.29	0.011081293
ENSMUSG00000049858	-2.29	2.1049E-19
ENSMUSG00000027187	-2.29	1.8222E-20
ENSMUSG00000057230	-2.30	1.70511E-08
ENSMUSG00000052812	-2.30	1.4319E-12
ENSMUSG00000074254	-2.30	2.99717E-10
ENSMUSG00000014547	-2.30	6.75641E-09
ENSMUSG00000029765	-2.31	6.16098E-06
ENSMUSG00000044296	-2.32	0.001061182
ENSMUSG00000044676	-2.32	8.32352E-09
ENSMUSG00000097470	-2.32	0.001868992
ENSMUSG00000044378	-2.32	0.033126626
ENSMUSG00000049427	-2.32	0.025360808
ENSMUSG00000034858	-2.32	4.69699E-19
ENSMUSG00000028834	-2.33	0.013149622

Ensembl gene id	log2FC	p-adjusted
ENSMUSG00000078680	-2.33	1.51928E-05
ENSMUSG00000022464	-2.33	9.01062E-21
ENSMUSG00000025937	-2.33	3.04687E-20
ENSMUSG00000019787	-2.34	0.005465453
ENSMUSG00000055980	-2.34	5.03357E-11
ENSMUSG00000031461	-2.34	0.012604643
ENSMUSG00000059824	-2.35	2.48117E-09
ENSMUSG00000025004	-2.35	1.89071E-21
ENSMUSG00000040270	-2.35	9.59834E-13
ENSMUSG00000041476	-2.35	0.011661654
ENSMUSG00000020154	-2.36	3.72267E-20
ENSMUSG00000031010	-2.36	2.90196E-20
ENSMUSG00000027559	-2.37	1.99776E-05
ENSMUSG00000099032	-2.37	4.2182E-10
ENSMUSG00000061331	-2.37	2.56065E-11
ENSMUSG00000035933	-2.37	3.12283E-14
ENSMUSG00000097969	-2.37	0.000810926
ENSMUSG00000005580	-2.38	3.27669E-11
ENSMUSG00000084761	-2.38	0.004387425
ENSMUSG00000087613	-2.38	0.000115502
ENSMUSG00000025262	-2.39	6.77177E-11
ENSMUSG00000052544	-2.39	0.015643912
ENSMUSG00000025006	-2.40	4.47585E-13
ENSMUSG00000041653	-2.40	0.000120104
ENSMUSG00000062496	-2.41	0.019718198
ENSMUSG00000017718	-2.41	6.51757E-13
ENSMUSG00000038725	-2.41	0.000148885
ENSMUSG00000033713	-2.41	4.48037E-13
ENSMUSG00000048371	-2.41	1.04736E-11
ENSMUSG00000053846	-2.42	6.26219E-18
ENSMUSG00000033308	-2.42	7.86958E-23
ENSMUSG00000086265	-2.42	0.025470537
ENSMUSG00000027871	-2.43	0.038656604
ENSMUSG00000054659	-2.43	2.04669E-06
ENSMUSG00000021466	-2.44	3.34275E-12
ENSMUSG00000034295	-2.44	0.001014679
ENSMUSG00000044795	-2.44	2.35349E-09
ENSMUSG00000089960	-2.44	8.47624E-22
ENSMUSG00000052496	-2.44	1.52264E-05
ENSMUSG00000097730	-2.44	1.81425E-12
ENSMUSG00000027887	-2.45	0.005179993
ENSMUSG00000055341	-2.45	0.003941104
ENSMUSG00000010651	-2.45	0.002579517
ENSMUSG00000086043	-2.45	0.018372469
ENSMUSG00000097458	-2.46	0.042250124
ENSMUSG00000084923	-2.46	0.013615402
ENSMUSG00000035836	-2.46	7.63503E-23
ENSMUSG00000024600	-2.46	0.00510705
ENSMUSG00000064372	-2.47	1.55646E-10
ENSMUSG00000010025	-2.48	3.71864E-06
ENSMUSG00000093625	-2.48	0.008348561
ENSMUSG00000057003	-2.48	0.022711337

Ensembl gene id	log2FC	p-adjusted
ENSMUSG00000053303	-2.48	3.02344E-24
ENSMUSG00000055730	-2.48	2.33333E-23
ENSMUSG00000056666	-2.49	5.23547E-07
ENSMUSG00000019883	-2.49	8.11468E-19
ENSMUSG00000057722	-2.49	0.006268335
ENSMUSG00000020673	-2.49	0.018175017
ENSMUSG00000051984	-2.49	1.58153E-10
ENSMUSG00000038092	-2.49	0.001156793
ENSMUSG00000038894	-2.49	0.005386825
ENSMUSG00000018796	-2.50	5.45351E-24
ENSMUSG00000094743	-2.50	0.019357366
ENSMUSG00000024899	-2.50	6.61374E-19
ENSMUSG00000041624	-2.50	1.91234E-05
ENSMUSG00000052392	-2.51	3.65919E-10
ENSMUSG00000064367	-2.51	1.08009E-16
ENSMUSG00000089704	-2.51	0.002900998
ENSMUSG00000082179	-2.51	0.000525938
ENSMUSG00000087030	-2.51	0.021619704
ENSMUSG00000015405	-2.51	0.000164759
ENSMUSG00000085929	-2.51	5.79632E-05
ENSMUSG00000064347	-2.51	0.011038907
ENSMUSG00000022853	-2.51	1.78218E-06
ENSMUSG00000044749	-2.52	2.54372E-23
ENSMUSG00000061436	-2.52	5.34647E-20
ENSMUSG00000039763	-2.52	1.12708E-16
ENSMUSG00000028024	-2.52	6.54598E-24
ENSMUSG00000043461	-2.53	0.021541284
ENSMUSG00000061816	-2.53	0.005132201
ENSMUSG00000018868	-2.54	0.005232521
ENSMUSG00000029268	-2.54	0.000380464
ENSMUSG00000025815	-2.54	5.61107E-23
ENSMUSG00000035606	-2.54	0.035095084
ENSMUSG00000096674	-2.55	7.49122E-07
ENSMUSG00000044005	-2.55	7.79418E-22
ENSMUSG00000022419	-2.56	3.7061E-24
ENSMUSG00000066647	-2.57	1.42922E-07
ENSMUSG00000094030	-2.57	1.47921E-06
ENSMUSG00000044365	-2.57	0.025374468
ENSMUSG00000035878	-2.57	9.99314E-24
ENSMUSG00000054385	-2.57	9.75431E-06
ENSMUSG00000030046	-2.58	2.63742E-06
ENSMUSG00000087695	-2.58	0.014452027
ENSMUSG00000085595	-2.59	0.004732006
ENSMUSG00000061462	-2.59	0.013261365
ENSMUSG00000086231	-2.59	4.77334E-08
ENSMUSG00000089873	-2.60	3.15567E-06
ENSMUSG00000039004	-2.61	1.23928E-08
ENSMUSG00000096910	-2.62	1.0464E-05
ENSMUSG00000078687	-2.62	1.15657E-06
ENSMUSG00000054514	-2.62	0.025274604
ENSMUSG00000096688	-2.63	4.73585E-06
ENSMUSG00000078683	-2.63	8.98819E-06

Ensembl gene id	log2FC	p-adjusted
ENSMUSG00000064349	-2.63	3.51097E-06
ENSMUSG00000089675	-2.63	1.10649E-05
ENSMUSG00000063488	-2.63	1.28305E-10
ENSMUSG00000054417	-2.64	9.84051E-05
ENSMUSG00000047036	-2.64	5.37344E-18
ENSMUSG00000040978	-2.65	0.008192626
ENSMUSG00000057880	-2.65	3.24631E-25
ENSMUSG00000029797	-2.65	5.00249E-07
ENSMUSG00000051262	-2.65	0.042556542
ENSMUSG00000021198	-2.66	0.001412293
ENSMUSG00000028655	-2.66	1.76112E-12
ENSMUSG00000070385	-2.66	0.040020687
ENSMUSG00000041220	-2.67	2.09863E-26
ENSMUSG00000050195	-2.67	2.53976E-05
ENSMUSG00000022615	-2.67	1.62434E-23
ENSMUSG00000042540	-2.68	0.005403512
ENSMUSG00000094793	-2.68	4.92807E-07
ENSMUSG00000047419	-2.68	0.00487548
ENSMUSG00000043398	-2.68	0.000207863
ENSMUSG00000022003	-2.69	1.24225E-20
ENSMUSG00000094410	-2.69	3.97064E-22
ENSMUSG00000074375	-2.70	8.52053E-05
ENSMUSG00000064344	-2.72	3.16564E-07
ENSMUSG00000073842	-2.72	3.22435E-06
ENSMUSG00000034071	-2.73	0.00177103
ENSMUSG00000024924	-2.73	0.004852862
ENSMUSG00000029862	-2.74	0.0084324
ENSMUSG00000096449	-2.74	0.010405594
ENSMUSG00000037071	-2.75	1.07001E-06
ENSMUSG00000055202	-2.75	0.001504146
ENSMUSG00000070419	-2.76	0.000770208
ENSMUSG00000043418	-2.76	3.69512E-09
ENSMUSG00000072963	-2.76	0.000355604
ENSMUSG00000050919	-2.78	0.000361046
ENSMUSG00000073834	-2.79	3.94367E-07
ENSMUSG00000044820	-2.79	0.041639665
ENSMUSG00000053877	-2.80	1.65945E-19
ENSMUSG00000043924	-2.80	7.22198E-12
ENSMUSG00000098290	-2.80	0.001116731
ENSMUSG00000085962	-2.80	0.026026394
ENSMUSG00000078673	-2.81	5.60391E-07
ENSMUSG00000078674	-2.81	4.64386E-07
ENSMUSG00000070368	-2.81	1.93614E-09
ENSMUSG00000078675	-2.81	2.09769E-07
ENSMUSG00000082065	-2.81	0.006250763
ENSMUSG00000028039	-2.81	8.26606E-05
ENSMUSG00000049985	-2.82	8.95114E-25
ENSMUSG00000078688	-2.82	2.37373E-08
ENSMUSG00000057425	-2.82	2.0661E-05
ENSMUSG00000073830	-2.82	2.59331E-06
ENSMUSG00000098678	-2.82	0.005705528
ENSMUSG00000063415	-2.83	1.70228E-10

Ensembl gene id	log2FC	p-adjusted
ENSMUSG00000038201	-2.83	0.03198928
ENSMUSG00000021590	-2.83	0.007302476
ENSMUSG00000042589	-2.83	1.20265E-05
ENSMUSG00000053541	-2.84	0.04361329
ENSMUSG00000023800	-2.84	1.59148E-06
ENSMUSG00000097657	-2.86	4.56759E-06
ENSMUSG00000064356	-2.86	0.004316431
ENSMUSG00000090175	-2.87	2.92119E-05
ENSMUSG00000097766	-2.87	0.001448668
ENSMUSG00000043556	-2.87	0.042206751
ENSMUSG00000089943	-2.88	8.65652E-13
ENSMUSG00000067336	-2.89	4.43858E-20
ENSMUSG00000025202	-2.90	1.74078E-06
ENSMUSG00000022025	-2.90	3.80167E-06
ENSMUSG00000084839	-2.90	0.000876554
ENSMUSG00000078686	-2.91	4.54412E-06
ENSMUSG00000028396	-2.91	0.005654467
ENSMUSG00000040694	-2.91	0.013641779
ENSMUSG00000089827	-2.92	0.006957772
ENSMUSG00000003053	-2.92	1.46278E-08
ENSMUSG00000004939	-2.93	0.000735086
ENSMUSG00000040724	-2.93	0.012729111
ENSMUSG00000079055	-2.93	0.003718422
ENSMUSG00000026475	-2.94	0.004698905
ENSMUSG00000081053	-2.95	1.4493E-05
ENSMUSG00000091867	-2.96	0.008225015
ENSMUSG00000025019	-2.97	1.53844E-20
ENSMUSG00000040660	-2.97	1.47932E-24
ENSMUSG00000027022	-2.97	0.025466394
ENSMUSG00000054178	-2.97	7.60717E-10
ENSMUSG00000042248	-2.97	4.03733E-12
ENSMUSG00000045094	-2.98	5.81595E-18
ENSMUSG00000051747	-2.99	0.009763661
ENSMUSG00000034872	-2.99	0.013195864
ENSMUSG00000026839	-3.02	2.27305E-05
ENSMUSG00000031725	-3.02	3.13102E-10
ENSMUSG00000055137	-3.03	7.21152E-10
ENSMUSG00000062181	-3.03	4.23481E-33
ENSMUSG00000027796	-3.04	3.89635E-20
ENSMUSG00000005893	-3.04	1.76168E-22
ENSMUSG00000087638	-3.05	0.021993123
ENSMUSG00000097131	-3.05	1.52894E-15
ENSMUSG00000078185	-3.06	0.001046963
ENSMUSG00000067736	-3.06	0.002078307
ENSMUSG00000056133	-3.06	0.003908678
ENSMUSG00000001095	-3.07	0.011286555
ENSMUSG00000038594	-3.07	7.10746E-21
ENSMUSG00000090369	-3.07	6.12606E-06
ENSMUSG00000098354	-3.08	0.000413839
ENSMUSG00000052229	-3.08	0.000207739
ENSMUSG00000061959	-3.09	1.85841E-08
ENSMUSG00000035349	-3.10	2.81037E-30

Ensembl gene id	log2FC	p-adjusted
ENSMUSG00000053141	-3.10	0.001857336
ENSMUSG00000069456	-3.11	3.86249E-07
ENSMUSG00000026950	-3.11	0.000222793
ENSMUSG00000097762	-3.12	3.73246E-08
ENSMUSG00000084781	-3.14	0.021906522
ENSMUSG00000054453	-3.15	9.78177E-12
ENSMUSG00000005952	-3.16	0.022434663
ENSMUSG00000024298	-3.16	5.72085E-26
ENSMUSG00000006777	-3.17	0.032268046
ENSMUSG00000086392	-3.18	0.001399245
ENSMUSG00000051452	-3.20	1.28907E-18
ENSMUSG00000091562	-3.21	8.52042E-05
ENSMUSG00000058921	-3.21	1.72542E-31
ENSMUSG00000094441	-3.23	6.1022E-11
ENSMUSG00000039704	-3.23	2.33115E-30
ENSMUSG00000090942	-3.23	1.12159E-09
ENSMUSG00000052974	-3.24	9.93267E-07
ENSMUSG00000025194	-3.24	1.96679E-35
ENSMUSG00000022878	-3.28	0.019764822
ENSMUSG00000071204	-3.28	6.79112E-11
ENSMUSG00000030378	-3.28	4.83481E-37
ENSMUSG00000058360	-3.29	0.015693962
ENSMUSG00000082364	-3.31	0.001142494
ENSMUSG00000044359	-3.34	8.07155E-25
ENSMUSG00000040093	-3.34	0.008356552
ENSMUSG00000031326	-3.37	1.27272E-09
ENSMUSG00000060530	-3.37	5.07405E-05
ENSMUSG00000027855	-3.38	4.09746E-05
ENSMUSG00000052595	-3.38	3.411E-37
ENSMUSG00000097417	-3.39	0.005149562
ENSMUSG00000087151	-3.39	0.000432579
ENSMUSG00000027762	-3.40	5.9242E-05
ENSMUSG00000087353	-3.41	0.011783305
ENSMUSG00000022129	-3.42	3.24391E-14
ENSMUSG00000066154	-3.43	4.23481E-33
ENSMUSG00000072294	-3.44	4.49012E-14
ENSMUSG00000003477	-3.45	1.24901E-07
ENSMUSG00000033107	-3.45	5.23538E-23
ENSMUSG00000050663	-3.46	0.000222418
ENSMUSG00000041078	-3.46	2.41078E-09
ENSMUSG00000022383	-3.46	4.34431E-30
ENSMUSG00000021228	-3.48	1.95437E-09
ENSMUSG00000085439	-3.51	0.003522409
ENSMUSG00000034584	-3.52	4.6853E-26
ENSMUSG00000006711	-3.52	4.01272E-32
ENSMUSG00000093772	-3.53	0.039618697
ENSMUSG00000083287	-3.54	3.09573E-07
ENSMUSG00000097380	-3.55	0.024738238
ENSMUSG00000039809	-3.55	3.99254E-25
ENSMUSG00000027870	-3.59	4.976E-22
ENSMUSG00000042717	-3.60	0.041434599
ENSMUSG00000083478	-3.60	2.39291E-05

Ensembl gene id	log2FC	p-adjusted
ENSMUSG00000086253	-3.61	6.84312E-06
ENSMUSG00000038233	-3.64	3.2926E-33
ENSMUSG00000045149	-3.65	0.006832929
ENSMUSG00000005220	-3.71	0.015747626
ENSMUSG00000021416	-3.71	3.73877E-10
ENSMUSG00000082363	-3.73	4.24973E-10
ENSMUSG00000089694	-3.74	8.36449E-11
ENSMUSG00000090284	-3.74	0.000838931
ENSMUSG00000035296	-3.75	0.013332894
ENSMUSG00000085095	-3.77	0.001773052
ENSMUSG00000045441	-3.83	9.63551E-26
ENSMUSG00000018581	-3.88	6.33781E-07
ENSMUSG00000047793	-3.89	6.94507E-20
ENSMUSG00000096493	-3.90	0.031946828
ENSMUSG00000097724	-3.91	0.020871193
ENSMUSG00000030483	-3.91	1.67144E-13
ENSMUSG00000035948	-3.92	1.17774E-17
ENSMUSG00000049694	-3.94	0.000216322
ENSMUSG00000079588	-3.94	0.000271451
ENSMUSG00000033624	-3.95	7.19688E-38
ENSMUSG00000087579	-3.98	1.2469E-06
ENSMUSG00000078685	-3.98	0.008274168
ENSMUSG00000024411	-4.03	0.005326747
ENSMUSG00000043165	-4.05	0.03848994
ENSMUSG00000097452	-4.07	1.18141E-05
ENSMUSG00000099242	-4.07	1.48269E-05
ENSMUSG00000026691	-4.08	2.56441E-09
ENSMUSG00000097691	-4.14	2.53011E-14
ENSMUSG00000091405	-4.16	2.52118E-06
ENSMUSG00000072664	-4.21	2.13789E-30
ENSMUSG00000041596	-4.22	0.000211222
ENSMUSG00000026567	-4.26	0.00093709
ENSMUSG00000055775	-4.30	0.036453261
ENSMUSG00000083116	-4.31	9.12023E-05
ENSMUSG00000090399	-4.32	0.031654209
ENSMUSG00000073840	-4.37	0.040779443
ENSMUSG00000027820	-4.39	3.70488E-10
ENSMUSG00000095532	-4.51	1.92428E-20
ENSMUSG00000020037	-4.51	1.42884E-23
ENSMUSG00000056018	-4.51	0.039018637
ENSMUSG00000081207	-4.62	0.000208214
ENSMUSG00000097322	-4.65	0.000807753
ENSMUSG00000084834	-4.66	0.000112716
ENSMUSG00000073835	-4.67	3.16737E-13
ENSMUSG00000050423	-4.72	0.01540191
ENSMUSG00000094222	-4.80	0.004905214
ENSMUSG00000082173	-4.81	0.000303503
ENSMUSG00000024211	-4.82	0.022783365
ENSMUSG00000088022	-4.89	2.14661E-07
ENSMUSG00000096652	-4.90	0.032287297
ENSMUSG00000085132	-5.49	0.001767215
ENSMUSG00000020052	-5.88	0.012604322

Ensembl gene id	log2FC	p-adjusted
ENSMUSG00000059668	-5.92	0.034223282
ENSMUSG00000098741	-6.12	1.91649E-12
ENSMUSG00000022347	-6.78	4.27664E-21
ENSMUSG00000048424	-6.82	4.46867E-08
ENSMUSG00000000001	-11.71	1.048E-158
